# Quantitative In Silico Evaluation of Allergenic Proteins from *Anacardium occidentale*, *Carya illinoinensis*, *Juglans regia* and *Pistacia vera* and Their Epitopes as Precursors of Bioactive Peptides †

**DOI:** 10.3390/cimb44070214

**Published:** 2022-07-06

**Authors:** Piotr Minkiewicz, Christopher P. Mattison, Małgorzata Darewicz

**Affiliations:** 1 Chair of Food Biochemistry, University of Warmia and Mazury in Olsztyn, Plac Cieszyński 1, 10-726 Olsztyn-Kortowo, Poland; darewicz@uwm.edu.pl; 2 Southern Regional Research Center, FPSQ, ARS, U.S. Department of Agriculture, New Orleans, LA 70124, USA

**Keywords:** food allergy, tree nut, bioactive, peptide, bioinformatics, cupin, vicilin, legumin, albumin, epitope

## Abstract

The aim of the study presented here was to determine if there is a correlation between the presence of specific protein domains within tree nut allergens or tree nut allergen epitopes and the frequency of bioactive fragments and the predicted susceptibility to enzymatic digestion in allergenic proteins from tree nuts of cashew (*Anacardium occidentale*), pecan (*Carya illinoinensis*), English walnut (*Juglans regia*) and pistachio (*Pistacia vera*) plants. These bioactive peptides are distributed along the length of the protein and are not enriched in IgE epitope sequences. Classification of proteins as bioactive peptide precursors based on the presence of specific protein domains may be a promising approach. Proteins possessing a vicilin, N-terminal family domain, or napin domain contain a relatively low occurrence of bioactive fragments. In contrast, proteins possessing the cupin 1 domain without the vicilin N-terminal family domain contain a relatively high total frequency of bioactive fragments and predicted release of bioactive fragments by the joint action of pepsin, trypsin, and chymotrypsin. This approach could be utilized in food science to simplify the selection of protein domains enriched for bioactive peptides.

## 1. Introduction

Food proteins contain the precursors of biologically active peptides. The most extensively studied activities of food derived peptides are (1) inhibition of angiotensin-converting enzyme (ACE) (EC 3.4.15.1), (2) inhibition of dipeptidyl peptidase IV (EC 3.4.14.5) and (3) antioxidative activity [1,2].

ACE activity regulates blood pressure by causing blood vessels to constrict and is a target for the rational design of bioactive peptides [3]. This enzyme hydrolyzes inactive peptide Angiotensin I to vasoconstrictor Angiotensin II. The second peptide causes vasoconstriction and thus increase of blood pressure. Its inhibitors serve as antihypertensive drugs. Many peptides from foods reveals similar activity [1,2]. The role of dipeptidyl peptidases (e.g., dipeptidyl peptidase III and IV) has been reviewed by Sato and Ogita [4]. Dipeptidyl peptidase III (EC 3.4.14.4) degrades the vasoconstrictor angiotensin II, among other substrates. Dipeptidyl peptidase III inhibitors may therefore also be factors affecting blood pressure [5]. Dipeptidyl peptidase IV is a target for antidiabetic drugs inhibiting this enzyme [1,2,4]. Polyphenols and other antioxidants from diverse foods have beneficial anti-cancer and protective cardiovascular health activities [6]. Tree nuts are an excellent source of antioxidant activity and phytochemicals that are beneficial to human health [7]. 

Bioactive peptides can be found in diverse foods including those from animal and plant sources [8,9]. For example, peptides from wheat, oat, barley, and rice harbor sequences correlated with ACE and dipeptidyl peptidase inhibition, while peptides from rice and wheat storage proteins contain anti-cancer sequences [10]. Tree nut proteins are also sources of peptides with bioactive potential. For example, proteins from walnut (*Juglans regia*) are known as precursors of angiotensin-converting enzyme (ACE) inhibitors [11,12,13], dipeptidyl peptidase IV inhibitors [14], peptides with antioxidant activity [15,16,17,18,19,20,21], peptides with anticancer activity [16], tyrosinase (EC 1.14.18.1) inhibitor [22], and anti-fatigue peptides [23]. Peptides with sequences YEP [11] and YVPHWEL [12] may serve as examples of ACE inhibitors from walnut. Kong et al. [14] has measured DPPIV inhibitory activity of hydrolysate of walnut proteins. Nine peptides have been identified using mass spectrometry. PPPIV inhibition by peptides was predicted using molecular docking. The antioxidative activity of walnut hydrolyzates and peptides was measured using 2,2-diphenyl-1-picrylhydrazyl (DPPH), 2,2′-azino-bis (3-ethylbenzothiazoline-6-sulphonic acid (ABTS), and superoxide radical scavenging assays. Peptide with sequence ADAF is an example of antioxidative peptide from walnut. Liu et al. [19] and Wang et al. [20,21] highlighted neuroprotective and anti-inflammatory effects of antioxidative peptides. The peptide with sequence FPY has been found as tyrosinase inhibitor [22]. Detailed review about biologically active peptides from walnut has been recently published [13]. Cashew (*Anacardium occidentale*), pecan (*Carya illinoinensis*), and pistachio (*Pistacia vera*) also are known as sources of peptides possessing various biological activities [24,25,26]. Known peptides isolated form cashew and pistachio revealed ACE inhibitory activity [24,25]. Peptides identified in the pecan hydrolysates represent diverse biological activities including ACE and DPPIV inhibition, antioxidative, anticancerogenic and other activities [26].

Food allergies are a worldwide health problem affecting millions of people. Food allergies are the result of a misguided immune response and are usually mediated by immunoglobulin-E (IgE) antibodies binding to otherwise innocuous food proteins. Several reasons, including environmental contributions, skin barrier dysfunction, nutritional deficiency, and microbiome alterations are thought to contribute to the apparent increase in food allergy incidence over the past three decades [27,28]. Food allergic reaction symptoms may include itching, hives, swelling, wheezing, abdominal pain, diarrhea, dizziness, fainting, or anaphylaxis in extreme cases. Peanuts and tree nuts (walnut, pecan, cashew, pistachio) are included in a group of eight foods that commonly cause food allergy [29]. Three seed storage proteins from peanuts and tree nuts commonly act as immuno-dominant food allergens, including 2S albumin, 7S vicilin, and 11S legumin proteins, and the peptides derived from these proteins have been shown to bind IgE from tree nut allergic volunteers [30]. 

The biological, molecular, immunological, and physico-chemical properties of 2S albumin, 7S vicilin, and 11S legumin proteins from peanuts and tree nuts that contribute to their propensity to commonly act as allergens are not fully understood. A number of the 7S vicilin and 11S legumin proteins in the cupin family have been identified as plant food allergens. Several factors including protein stability, resistance to physical processing and enzymatic digestion, amino acid sequence, and protein conformation are thought to be important factors [31,32,33]. In addition, the amino acid sequence and conformational similarities of these proteins contribute to their potential to cross-react with IgE cognate to allergens from other tree nuts or peanuts [34,35,36]. Contemporary strategies of epitope prediction assume that they can be discriminated from the entire protein chain due to amino acid composition, amino acid order, or oligopeptide composition [37,38,39,40]. Alternatively, it may be that that epitopes differ from entire protein chains in frequency of occurrence of bioactive fragments. The most simple measure of this type of inspection would be the sum of fragment frequencies with particular activities [41]. 

The aim of the study presented here was to determine if there is a correlation between the presence of specific protein domains within tree nut allergens or tree nut allergen epitopes and the frequency of bioactive fragments and predicted susceptibility to enzymatic digestion in allergenic proteins from tree nuts of cashew (*Anacardium occidentale*), pecan (*Carya illinoinensis*), English walnut (*Juglans regia*) and pistachio (*Pistacia vera*) plants.

## 2. Materials and Methods

### 2.1. Protein Sequences 

Protein sequences were obtained from the UniProt database https://www.uniprot.org/ [42] (accessed on 30 August 2020). The list of allergenic proteins is provided in Table 1. Sequences of linear epitopes were taken from the Immune Epitope Database (IEDB) https://www.iedb.org/ [43] (accessed on 30 August 2020). Search options included: name of plant, both linear and discontinuous epitopes, all types of assays, positive assay results, no MHC restriction, any host and any disease. We have found only linear epitopes attributed to walnut, cashew, pecan or pistachio. Epitopes were aligned to protein sequences. BLAST program https://www.ebi.ac.uk/Tools/sss/ncbiblast/ [44] (accessed on 30 August 2020) was used to find fragments overlapping partially or wholly with epitopes in precursor proteins belonging to various species. Due to fact that default parameters of BLAST program often produce false negative results with short peptide sequences used as a query the following search parameters were used: PAM 30 matrix and word size 3. Other parameters were default. Overlapping and adjacent epitopes were merged into longer fragments in agreement with the peptide scanning strategy, commonly used for epitope mapping [45]. 

### 2.2. BIOPEP-UWM Database of Bioactive Peptides

Both entire protein sequences and epitopic regions were submitted to analysis via the BIOPEP-UWM database of bioactive peptides https://biochemia.uwm.edu.pl/biopep-uwm/ [46] (accessed on 30 September 2020). The BIOPEP-UWM output included profiles and computation of the frequency of bioactive fragments occurrence in protein sequence (A) [47] calculated according to Equation (1).

A = a/N(1)

where:

a—the number of fragments with given activity in a protein sequence,
N—the number of amino acid residues of protein chain.

The total frequency of occurrence of bioactive fragments (∑A) introduced by Minkiewicz et al. [41] was used as a parameter characterizing entire protein sequences and epitopes as precursors of bioactive fragments. 

Simulated proteolysis was performed using the BIOPEP-UWM database. Theoretical degree of hydrolysis (DH_T_) and total frequency of release of bioactive fragments by proteolytic enzymes (∑A_E_) defined as sum of A_E_ values for all peptide activities, were used as a scores characterizing entire protein sequences and epitopes as precursors of bioactive fragments.

Theoretical degree of hydrolysis (DH_T_) was calculated according to the Equation (2) [48].

DH_t_ = d/D × 100% (2)

d—number of hydrolyzed peptide bonds in a protein/peptide chain,
D—total number of peptide bonds in a protein/peptide chain.

Frequency of release of bioactive fragments by proteolytic enzymes was calculated according to the Equation (3) [41].

A_E_ = d/N (3)

d—the number of peptides with a given activity (e.g., ACE inhibitors) released by a given enzyme (e.g., trypsin),
N—the number of amino acid residues in protein.

DH_t_ and ∑A_E_ were calculated for simulated hydrolysis by enzymes of digestive tract: pepsin at pH > 2 (EC 3.4.23.1, BIOPEP-UWM ID 39), trypsin (EC 3.4.21.4, BIOPEP-UWM ID 12) and chymotrypsin A (EC 3.4.21.1, BIOPEP-UWM ID 11), in agreement with the recommendation of Brodkorb and co-workers [49]. 

Protein were classified according to the InterPro database http://www.ebi.ac.uk/interpro/ [50] (accessed on 30 August 2020).

The following domain sets were defined:

(1) IPR036312, IPR016140, IPR000617;
(2) IPR006045, IPR014710, IPR011051, IPR006792;
(3a) IPR006045, IPR014710, IPR011051;
(3b) IPR022379, IPR006044, IPR006045, IPR014710, IPR011051.

Set 3 is defined as the merged sets of 3a and 3b.

For sets of protein sequences and epitope sequences arithmetic means of ∑A, DH_t_ and ∑A_E_ and standard deviations (SD) were calculated. 

### 2.3. Statistical Analysis 

Statistical significance of differences was calculated using *t*-test. Pairwise comparison was performed between features of group of entire proteins possessing given set of domains and epitopes from these proteins. Differences in features of proteins and in features of epitopes between particular groups of proteins were also calculated. Differences were considered as statistically significant if *p* value was below 0.05.

Program Heatmapper: http://www.heatmapper.ca/ (accessed on 30 August 2020) [51] was used for visualization of results of calculations.

**Figure 1:**
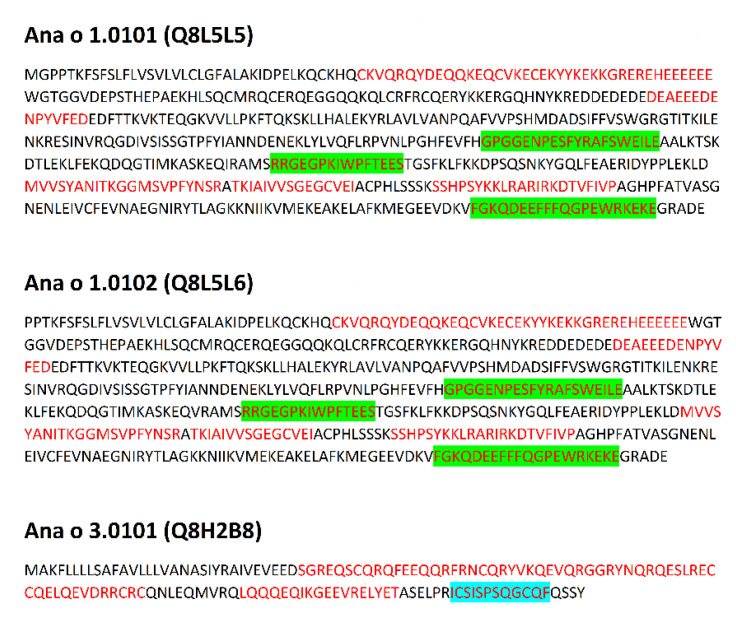
Sequences of allergens from *Anacardium occidentale*. Location of epitopes is indicated using red font. Green background indicates epitopes with highest ∑A (range 1.6000–2.1000), blue background indicates epitopes with lowest ∑A (range 0.1000–0.5999).

**Figure 2:**
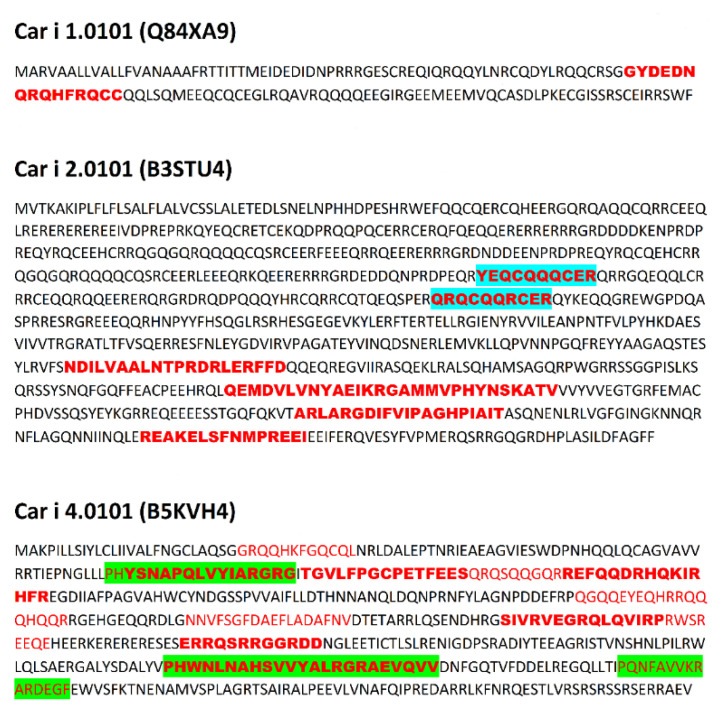
Sequences of allergens from *Carya illinoinensis*. Location of epitopes is indicated using red font. Green background indicates epitopes with highest ∑A (range 1.6000–2.1000), blue background indicates epitopes with lowest ∑A (range 0.1000–0.5999). Bold font indicates epitopes and their fragments occurring both in *Carya illinoinensis* and *Juglans regia* proteins.

**Figure 3:**
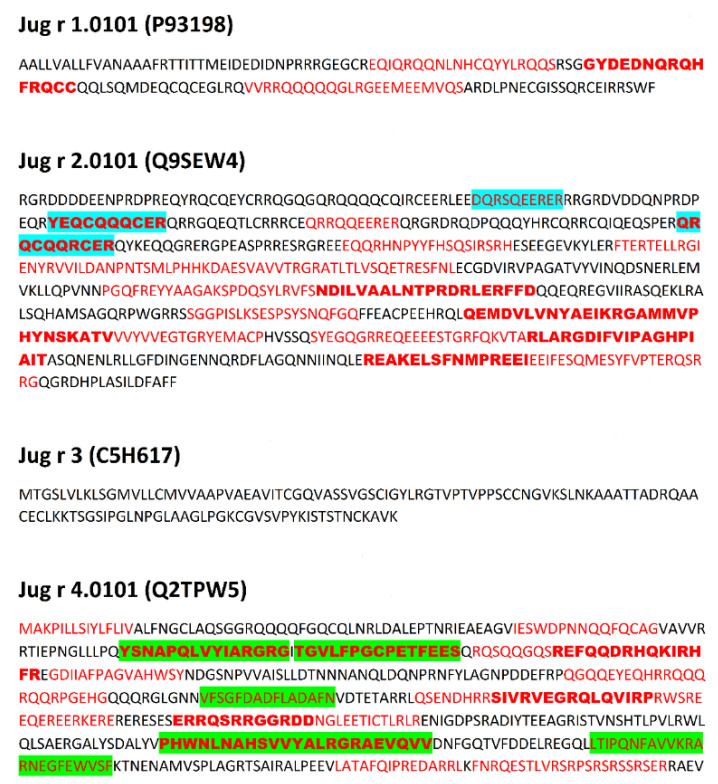
Sequences of allergens from *Juglans regia*. Location of epitopes is indicated using red font. Green background indicates epitopes with highest ∑A (range 1.6000–2.1000), blue background indicates epitopes with lowest ∑A (range 0.1000–0.5999). Bold font indicates epitopes and their fragments occurring both in *Carya illinoinensis* and *Juglans regia* proteins.

**Figure 4:**
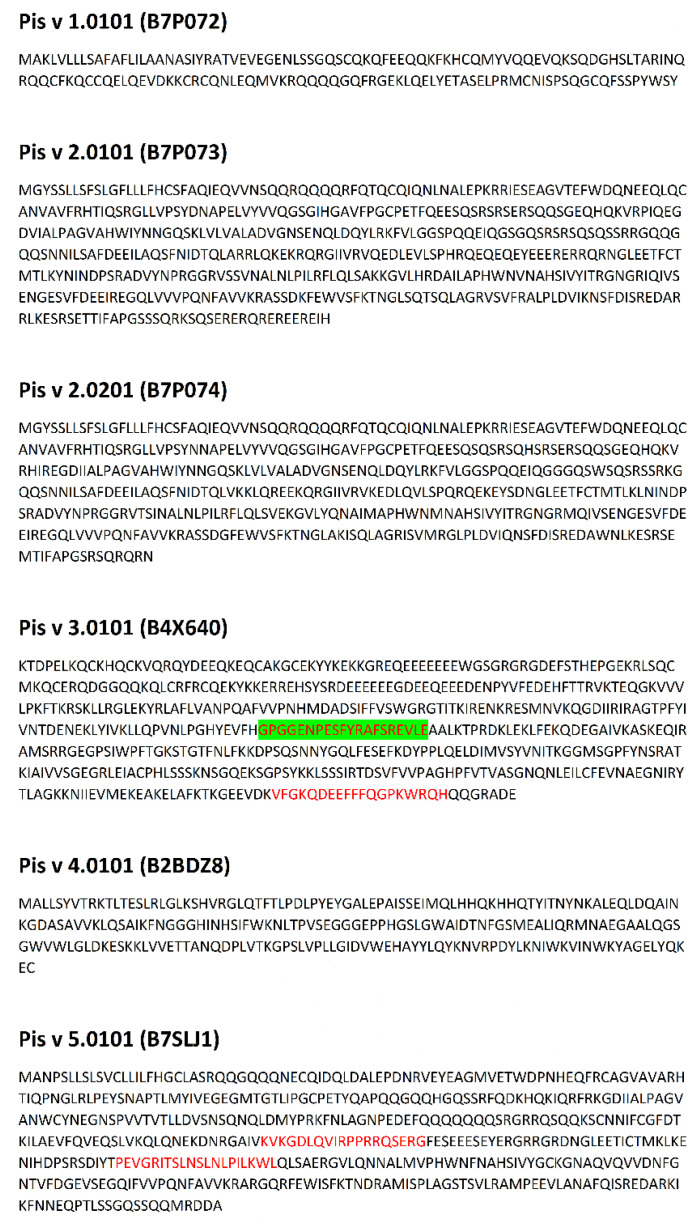
Sequences of allergens from *Pistacia vera*. Location of epitopes is indicated using red font. Green background indicates epitopes with highest ∑A (range 1.6000–2.1000).

Data concerning proteins submitted for analysis is presented in Table 1. Sequences of proteins with epitopes are shown in Figure 1, Figure 2, Figure 3 and Figure 4 and in Table S1 in Supplementary Materials.

### 2.4. Protein Modeling

Protein homology models were created and visualized using MOE software (version 2020.09, Chemical Computing Group, Montreal, QC, Canada, https://www.chemcomp.com/ (accessed on 30 August 2020)). Ana o 1.0101 and Ana o 2.0101 allergen models were created using the crystal structures of the major peanut allergens Ara h 1 (pdb 3SMH) [52] and Ara h 3 (pdb 3C3V) [53] as templates downloaded from the pdb website (https://www.rcsb.org/pages/policies (accessed on 30 August 2020)) [54]. 

## 3. Results and Discussion

### 3.1. Tree Nut Epitopes

The Immune Epitope Database (IEDB) contains 150 sequential epitopes within the tree nut allergens examined and sequences of their linear epitopes, retrieved from the IEDB, are summarized in Table S2 in Supplementary Materials. Three homologous and commonly immuno-dominant tree nut allergens, including the 2S albumin, 7S vicilin, and 11S legumin, from each species were examined along with a few other allergens included in the IUIS list for walnut and pistachio. 

Due to the relatively high level of conservation among the immuno-dominant peanut and tree nut allergens, they commonly cross-react [34]. For example, identity among tree nut allergens, such as those from pecan and walnut, can reach as high as 95% [34]. Clinical reactivity occurs when someone has allergic symptoms to a closely related food due to IgE recognition of a similar amino acid sequence or structural similarity among proteins in the food. For example, pecan and walnut allergens often cross-react, and recent studies indicate pecan epitopes represent a subset of walnut allergen epitopes [55]. Therefore, the presence of identical linear epitopes in allergens of *C. illinoinensis* and *J. regia* are summarized in Table 2. These epitopes were included in preprocessing analysis of epitopes of both species. Data preprocessing as described in the Methods section (aligning common epitopes to all proteins, merging adjacent and partially overlapping peptides) allowed the reduction of the epitope set to 64 non-redundant sequences. This preprocessing mimics the final step of experimental epitope mapping. Publication of Zhang et al. [56] concerning epitopes of tropomyosin from prawn *Exopalaemon modestus* many serve as a representative example of such work. The above Authors have found five epitopes with the length up to 38 amino acid residues on the basis of analysis performed using shorter overlapping or adjacent peptides. Discussion of the results included presence of the epitopes in sequences of other proteins with the same family. The sequences of epitopes after preprocessing and their location within proteins is presented in Figure 1, Figure 2, Figure 3 and Figure 4 and Table S1 in Supplementary Materials. 

### 3.2. Tree Nut Allergen Protein Domains

Proteins are classified into families based on the presence of characteristic domains [57,58,59]. A protein domain is considered a conserved part of a sequence and tertiary structure that is attributed to protein function, and exists independently of the rest of the protein chain [60]. The presence of domains may be the basis of classification of proteins as potential precursors of bioactive peptides [61]. Domains present in particular tree nut allergens are summarized in Table 1 and Table S1 (Supplementary Materials). 

The most abundant domains occurring in the tree nut allergens include IPR006045 (Cupin 1), IPR011051 (RmlC-like cupin domain superfamily), IPR011051 (RmlC-like cupin domain superfamily) and IPR014710 (RmlC-like jelly roll fold). The above domains occur in 11 proteins. For example, the IPR006045 and IPR011051 cupin domains are found in each of the 7S vicilin and 11S legumin allergens from each tree nut (Table 1). Two proteins, Car i 2 and Jug r 2, contain domain IPR006792 (Vicilin, N-terminal). A family of proteins containing the four domains mentioned above is cited as Cupin 1/Vicilin, N-terminal. Alternatively, a family of proteins with Cupin 1 domain, but without Vicilin, N-terminal domain can be named Cupin 1 family. Nine proteins investigated within this work belong to the above Cupin 1 family. A few of them also contain the IPR006044 (11-S seed storage protein, plant) and IPR022379 (11-S seed storage protein, conserved site) domains. Another group of proteins, including the 2S albumins Ana o 3, Car i 1, Jug r 1, and Pis v 1 and the non-specific lipid transfer protein type 1 (nsLTP1) Jug r 3 contain domains associated with lipid transfer: IPR016140 (Bifunctional inhibitor/plant lipid transfer protein/seed storage helical domain) and IPR036312 (Bifunctional inhibitor/plant lipid transfer protein/seed storage helical domain superfamily). One of these five proteins (Jug r 3) contains domain IPR000528 (Plant lipid transfer protein/Par allergen), whereas the four 2S albumins contain domain IPR000617 (Napin/Bra allergen). 

### 3.3. Correlation of Bioactive Fragments between Allergen Protein Domains and Epitopes

Profiles of the potential biological functions of specific allergen fragments are presented in Supplementary Materials (Tables S6–S21). One characteristic feature of these profiles is the presence of a large number of dipeptides as dominant bioactive fragment. There are also several bioactive tripeptides. Fragments revealing biological activity and longer than three amino acid residues are exceptions. We can thus suppose than dipeptide composition is crucial factor affecting frequency of occurrence of bioactive fragments in protein chain. Bioactive dipeptides may occur with high probability in any protein sequence. Longer fragments, such as epitopes may be common fragments of homologous proteins revealing a relatively high similarity of sequences [62]. Inhibitors of dipeptidyl peptidase IV (EC 3.4.14.5), angiotensin I-converting enzyme (EC 3.4.15.1), dipeptidyl peptidase III (EC 3.4.14.4) and antioxidative peptides are the most abundant bioactive peptides encrypted in the full-length allergen sequences and distinct epitopes.

**Figure 5:**
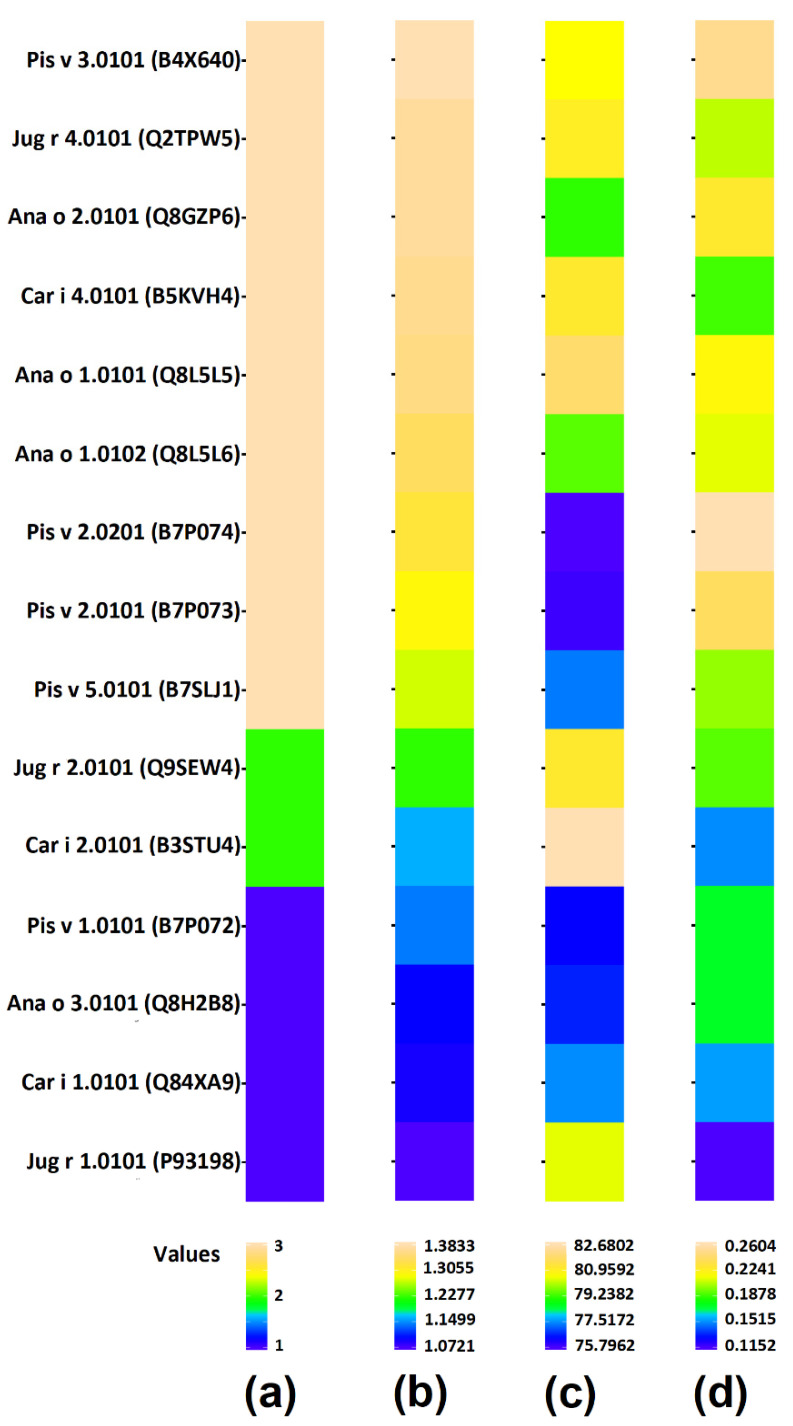
Scores describing entire proteins as potential precursors of bioactive peptides. (**a**) domain sets present in proteins: 1 (blue)—Set 1; 2 (green)—set 2; 3 (yellow)—set 3 (3a + 3b); (**b**) ∑A; (**c**) DH_t_ [%]; (**d**) ∑A_E_. Proteins are sorted from highest to lowest ∑A value (column (**b**)).

**Figure 6:**
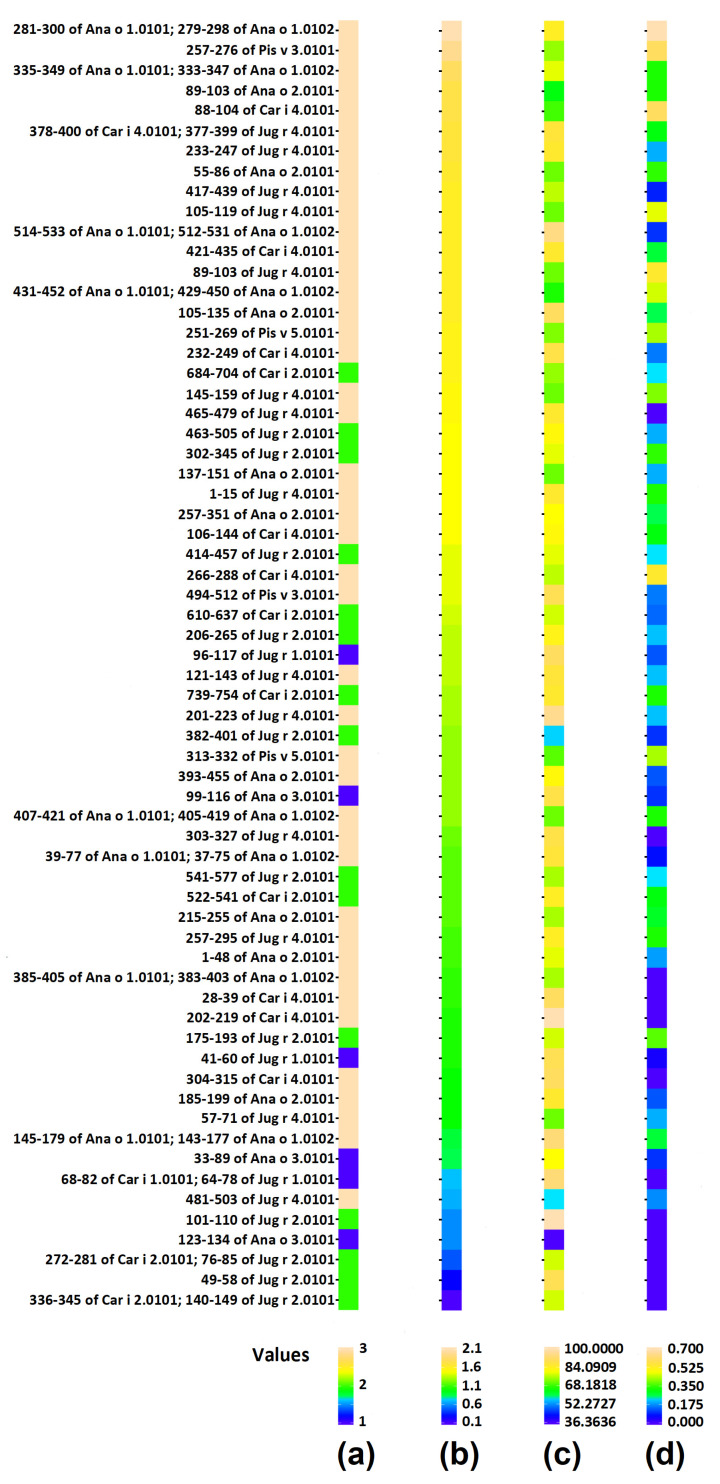
Scores concerning potential release of bioactive peptides from epitopes. (**a**) domain sets present in proteins: 1 (blue)—Set 1; 2 (green)—set 2; 3 (yellow)—set 3 (3a + 3b); (**b**) ∑A; (**c**) DH_t_ [%]; (**d**) ∑A_E_. Epitopes are sorted from highest to lowest ∑A value (column (**b**)).

**Figure 7:**
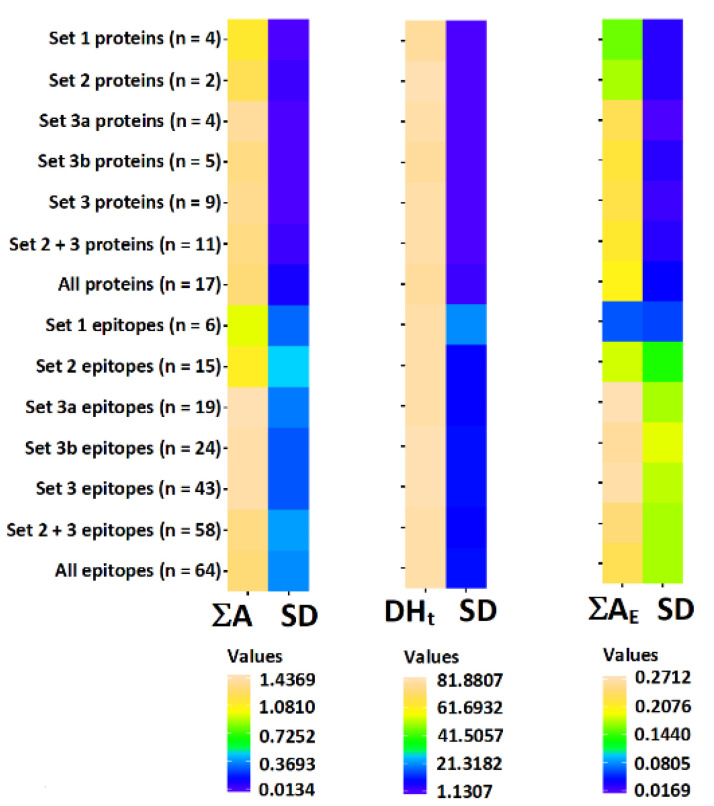
Scores attributed to particular set of domains. ∑A—total frequency of bioactive fragments occurrence in a protein sequence (Equation (1)); DH_t_—theoretical degree of hydrolysis (Equation (2)) expressed in %; ∑A_E_—total frequency of release of bioactive fragments by proteolytic enzymes (Equation (3)), SD—standard deviation.

**Figure 8:**
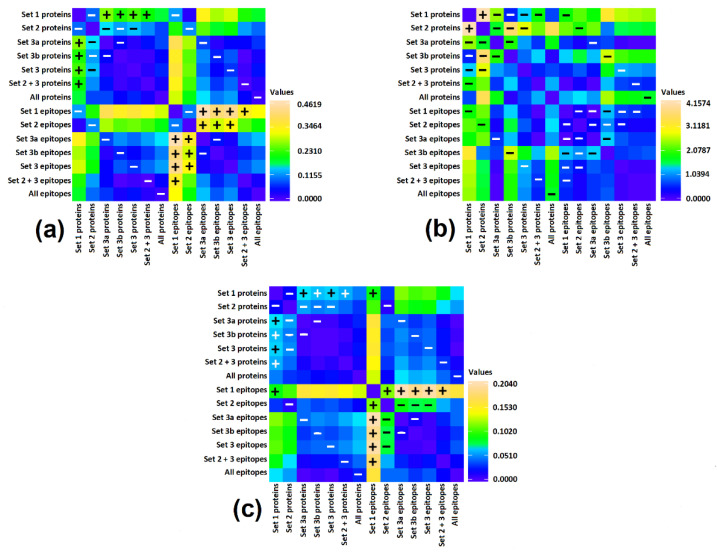
Differences in scores between groups of proteins containing particular sets of domains, expressed as matrices of Euclidean distances: (**a**) ∑A; (**b**) DH_t_ [%]; (**c**) ∑A_E_ Differences subjected to *t*-test are labeled. Symbol “+” indicates differences that are statistically significant at *p* < 0.05. Symbol “−“ indicates a lack of statistically significant difference.

All three scores describing occurrence and possibility of release of bioactive peptides from proteins and epitopes are presented in Figure 5 and Figure 6 respectively. The scores of individual proteins and individual epitopes are also summarized in Tables S3–S4 in Supplementary Materials. The mean scores of groups of proteins possessing particular sets of domains are presented in Figure 7 and in Table S5 in Supplementary Materials. The statistical significance of differences between particular groups of proteins is displayed in Figure 8 and in Table S5 in Supplementary Materials. 

Two proteins are not included in Figure 5: Jug r 3 (Non-specific lipid transfer protein) and Pis v 4.0101 (Superoxide dismutase). Their families are represented only by single proteins. Jug r 3 possesses the following scores: ∑A = 1.4369; DH_t_ = 66.1017% and ∑A_E_ = 0.2520. The ∑A and ∑A_E_ scores of Jug r 3 are the highest among all proteins subjected to analysis, whereas DH_t_ is lower than for all other proteins. Sequence analysis of Pis v 4.0101 reveals the following scores: ∑A = 1.4345; DH_t_ = 80.3493% and ∑A_E_ = 0.1867. The ∑A of Pis v 4.0101 is only slightly lower than for Jug r 3 and much higher than for all other proteins. The Pis v 4.0101 DH_t_ and ∑A_E_ values are not unique from scores of other proteins. No information about epitopes of the above two allergens is available in the IEDB database. Their scores were included for calculation of mean values for all proteins. 

Based on the results summarized in Figure 7 and Figure 8 as well as Table S3 we can conclude that scores describing the content and potential for release of bioactive peptides in the whole set of protein sequences and in the whole set of epitopes are nearly identical. Only epitopes from the Napin family (Set 1) reveal a lower ∑A_E_ value than entire proteins belonging to the same family (Figure 7 and Figure 8, Table S5). The standard deviation of scores for epitopes is larger than in this of proteins (Figure 7). The frequency of bioactive peptide occurrence varies between families. Proteins possessing the Cupin 1 domain (domain sets 3a and 3b) reveal a relatively higher ∑A score. Proteins containing Vicilin N-terminal family domain apart from Cupin 1 (set 2), have a much lower ∑A than proteins from the previous family. Proteins containing a Napin domain have the lowest ∑A among the proteins investigated. One protein containing plant lipid transfer protein/Par allergen domain instead of Napin possess a relatively higher total frequency of occurrence of bioactive fragments. Replacement of one of three domains leads to significant change in the of bioactive fragment within the protein sequence. Also, the presence or absence of the Vicilin N-terminal family domain markedly affects the biological activity of protein fragments. There is no difference in any score between sets 3a and 3b (Figure 8). These sets are therefore included in Figure 5 and Figure 6 as one ‘set 3’ including all proteins containing domains attributed to the Cupin 1 family, but without Vicilin N-terminal family domain. 

Epitopes within the proteins evaluated here reveal a broad range of frequency correlating with the occurrence of bioactive fragments (0.1–2.1). This range can be divided into four intervals: 0.1–0.5999; 0.6000–1.0999; 1.1000–1.5999 and 1.6000–2.1000 in agreement with a classification used by the Heatmapper program [51] (Table S4 in Supplementary Materials). Epitopes from the intervals with the highest and lowest ∑A are highlighted in Figure 1, Figure 2, Figure 3 and Figure 4. Epitopes from the interval with highest ∑A primarily contain fragments of proteins from the Cupin 1 family. 

Predicted products of enzymatic hydrolysis of proteins are presented in Table S23 in Supplementary Materials. Peptides predicted to be released should be preceded and followed by bonds susceptible to one of enzymes used. Peptides may be theoretically released if peptide bonds within peptide are resistant to proteolysis. Program simulating proteolysis assumes that all bonds theoretically susceptible to enzyme action are hydrolysed. This assumption is oversimplification as compared with experimental hydrolysis. Hydrolysis by three enzymes should theoretically lead to release of free amino acids and short peptides, containing two or three amino acid residues. Peptides longer than three amino acid residues are exceptions (Table S23 in Supplementary Materials). Bioactive dipeptides are among predicted products of hydrolysis. The same peptide sequences may be found both in Tables S6–S22 and in Table S23. 

There is no significant difference in the theoretical degree of hydrolysis between proteins and epitopes representing particular sets of domains with one exception. The difference between DH_t_ of set 2 (the highest DH_t_ among all groups of proteins) and DH_T_ of set 1 (lowest DH_t_) is statistically significant (Figure 8). The theoretical degree of hydrolysis of all proteins and almost all epitopes exceeds 50%. This indicates that the predicted products are short peptides and free amino acids. The degree of hydrolysis is calculated assuming that all bonds theoretically susceptible to the enzymes applied are hydrolyzed. In actual protein digestion experiments however, this assumption is usually not fulfilled. 

The frequency of bioactive fragment release by proteolytic enzyme reveals a high variability at the level of individual proteins and especially within epitope sequences (expressed as a large standard deviation—see Figure 7). Proteins and their epitopes with domains belonging to set 1 have significantly lower ∑A_E_ scores compared to proteins within set 3. In the case of epitopes, there is also significant difference between epitopes attributed to set 1 and 2 (i.e., the ∑A_E_ score for domain set 1 is lower than for set 2). On the other hand, in some instances there are epitopes with ∑A_E_ = 0 attributed to proteins containing N-terminal vicilin domain (set 2).

The possible influence of individual domains on scores characterizing individual proteins and epitopes and their potential as precursors of bioactive peptides is presented in Table 3. The plant lipid transfer protein/Par allergen domain is associated with high ∑A and ∑A_E_ scores, but this information is based on calculation results obtained for only one protein. The same finding is applicable to ∑A score in general for domains associated with manganese/iron superoxide dismutase. Domains possessing this label are not included in the conclusions presented here, due to the single sample analyzed. Among domains present in at least two proteins, the Napin/Bra allergen and Vicilin, N-terminal are associated with low ∑A and ∑A_E_ scores. In contrast, the other domains appear to have no defined influence on scores characterizing proteins as potential precursors of bioactive peptides.

**Figure 9:**
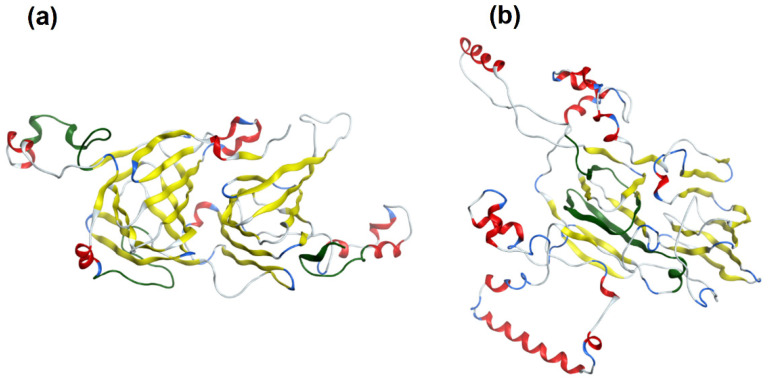
Cupin domain proteins associated with relatively high ∑A scores. Ana o 1.0101 or (**a**) and Ana o 2.0101 (**b**) ribbon models indicating the relative placement of segments within the two allergens having high ∑A scores. Loop sections are colored red, cupin domains yellow, and segments with high ∑A scores in green.

The presence of epitopes with highest frequency of occurrence correlating with bioactive fragments is consistent with the findings of Nardo et al. [61], who have drawn the conclusion that there are fragments within protein sequences considered as ‘hotspots’ rich in bioactive fragments. Epitopes from the interval with highest ∑A are fragments of proteins from the Cupin 1 family. For example, two segments with high ∑A scores in Ana o 2 overlap with epitopes and reside in the first cupin domain within the protein (Table 3 and Figure 1). Similarly, three segments with high ∑A scores in the Ana o 1 protein overlap with epitopes, but lie in loop regions in between and adjacent to the cupin domains (Table 3 and Figure 1). Two of the high scoring ∑A segments in Ana o 1 lie in between the two cupin domains, while the third resides in the carboxy terminal end of the protein (Figure 9). In contrast four of five epitopes from interval with lowest ∑A are fragments of proteins containing Vicilin, N-terminal family domain. However, other proteins from this family contain epitopes with relatively high ∑A values (e.g., fragment 684–704 of Car i 2.0101 or fragments 302–345 and 463–505 of Jug r. 2.0101), belonging to the interval 1.1000–1.5999. Lower scoring intervals 0.6000–1.0999 and 1.1000–1.5999 contain fragments of proteins belonging to all families possessing epitopes annotated in Immune Epitope Database.

The observed differences in bioactive fragment frequency between particular protein domains may provide an interesting approach for preliminary selection of food proteins as potential precursors of specific bioactive peptides. Early research works concerning classification of food proteins as precursors of bioactive peptides [63,64] were concentrated on the division of proteins into families based upon of differences in “A” scores corresponding to the particular activities. More recently, bioactive peptide research has included protein domain classifications [61,65]. However, there has been no association between the presence of certain domains and the incidence of bioactive fragments within those domains and their possible release by proteolytic enzymes. Successful implementation of this type of approach may allow the selection of plant or animal food varieties characterized by the presence of protein with domains harboring relatively rich sources of bioactive fragments.

## 4. Conclusions

In conclusion, tree nut allergens possessing a Cupin 1 (without Vicilin, N-terminal family) domain reveal a relatively high overall occurrence of bioactive fragments and predicted frequency of bioactive fragment release by the joint action of pepsin, trypsin, and chymotrypsin along the length of the protein and within epitopes. In a few cases, there are epitopes harboring an exceptionally high occurrence of bioactive fragments, and they are primarily present in Cupin 1 family proteins. Overall, there is no significant difference in total frequency of bioactive fragment occurrence or theoretical degree of hydrolysis in the protein sequences and linear epitopes of the tree nut allergens evaluated here. The frequency of bioactive fragment occurrence and predicted frequency of bioactive fragment release by the joint action of pepsin, trypsin and chymotrypsin varies between different protein domains commonly found in tree nut allergens. Proteins possessing a Vicilin, N-terminal family domain or Napin domain reveal a relatively low total frequency of bioactive fragments and predicted frequency of release by the joint action of pepsin, trypsin, and chymotrypsin both along the length of the protein and in epitopes. IgE epitopes within Vicilin, N-terminal family allergens have an exceptionally low frequency of bioactive fragments occurrence and predicted frequency of release by the joint action of pepsin, trypsin, and chymotrypsin.

## Figures and Tables

**Table 1:** List of allergenic proteins submitted to in silico analysis.

No	Species	Protein Type	Allergen Name	UniProt Access. No	InterPro Domain IDs ^1^	Set of Domains
1	*Anacardium occidentale*	7S vicilin	Ana o 1.0101	Q8L5L5	IPR006045, IPR014710, IPR011051	3a
2	*Anacardium occidentale*	7S vicilin	Ana o 1.0102	Q8L5L6	IPR006045, IPR014710, IPR011051	3a
3	*Anacardium occidentale*	11S legumin	Ana o 2.0101	Q8GZP6	IPR022379, IPR006044, IPR006045, IPR014710, IPR011051	3b
4	*Anacardium occidentale*	2S albumin	Ana o 3.0101	Q8H2B8	IPR036312, IPR016140, IPR000617	1
5	*Carya illinoinensis*	2S albumin	Car i 1.0101	Q84XA9	IPR036312, IPR016140, IPR000617	1
6	*Carya illinoinensis*	7S viclin	Car i 2.0101	B3STU4	IPR006045, IPR014710, IPR011051, IPR006792	2
7	*Carya illinoinensis*	11S legumin	Car i 4.0101	B5KVH4	IPR022379, IPR006044, IPR006045, IPR014710, IPR011051	3b
8	*Juglans regia*	2S albumin	Jug r 1.0101	P93198	IPR036312, IPR016140, IPR000617	1
9	*Juglans regia*	7S vicilin	Jug r 2.0101	Q9SEW4	IPR006045, IPR014710, IPR011051, IPR006792	2
10	*Juglans regia*	non-specific lipid transfer protein type 1 (nsLTP1)	Jug r 3	C5H617	IPR036312, IPR016140, IPR000528	-
11	*Juglans regia*	11S legumin	Jug r 4.0101	Q2TPW5	IPR022379, IPR006044, IPR006045, IPR014710, IPR011051	3b
12	*Pistacia vera*	2S albumin	Pis v 1.0101	B7P072	IPR036312, IPR016140, IPR000617	1
13	*Pistacia vera*	11S legumin	Pis v 2.0101	B7P073	IPR022379, IPR006044, IPR006045, IPR014710, IPR011051	3b
14	*Pistacia vera*	11S legumin	Pis v 2.0201	B7P074	IPR022379, IPR006044, IPR006045, IPR014710, IPR011051	3b
15	*Pistacia vera*	7S vicilin	Pis v 3.0101	B4X640	IPR006045, IPR014710, IPR011051	3a
16	*Pistacia vera*	manganese superoxide dismutase	Pis v 4.0101	B2BDZ8	IPR001189, IPR019833, IPR019832, IPR019831, IPR036324, IPR036314	-
17	*Pistacia vera*	11S legumin	Pis v 5.0101	B7SLJ1	IPR006044, IPR006045, IPR014710, IPR011051	3b

^1^ IDs of domains according to the InterPro database.

**Table 2:** Interspecies share of linear epitopes between proteins from *C. illinoinensis* and *J. regia*.

Epitopes (ID According to the Immune Epitope Database) ^1^	Allergen Containing Epitopes, Annotated in Immune Epitope Database	Allergen Containing Epitopes, Found in the UniProt Database Using BLAST
157220, 157381, 157603, 157808, 157835	Car i 4.0101	Jug r 4.0101
174135	Jug r 1.0101	Car i 1.0101
157811, 158509, 241161, 241344, 241355, 241557	Jug r 2.0101	Car i 2.0101
114508, 114569, 114610, 114627, 114695	Jug r 4.0101	Car i 4.0101

^1^ Sequences of epitopes retrieved from IEDB are presented in Table S2 (Supplementary Materials).

**Table 3:** Summary of domains present in allergenic proteins.

ID	Name	Score for Entire Proteins			Score for Epitopes		
		∑A	DH_t_	∑A_E_	∑A	DH_t_	∑A_E_
IPR000528	Plant lipid transfer protein/Par allergen	+ (s)	− (s)	+ (s)	nd	nd	nd
IPR000617	Napin/Bra allergen	−	−	−	−	0	−
IPR001189	Manganese/iron superoxide dismutase	+ (s)	0 (s)	0 (s)	nd	nd	nd
IPR006044	11-S seed storage protein, plant	0	0	0	0	0	0
IPR006045	Cupin 1	+	0	+	+	0	+
IPR006792	Vicilin, N-terminal	−	+	−	−	0	0
IPR011051	RmlC-like cupin domain superfamily	+	0	+	+	0	+
IPR014710	RmlC-like jelly roll fold	+	0	+	+	0	+
IPR016140	Bifunctional inhibitor/plant lipid transfer protein/seed storage helical domain	0	0	0	−	0	−
IPR019831	Manganese/iron superoxide dismutase, N-terminal	+ (s)	0 (s)	0 (s)	nd	nd	nd
IPR019832	Manganese/iron superoxide dismutase, C-terminal	+ (s)	0 (s)	0 (s)	nd	nd	nd
IPR019833	Manganese/iron superoxide dismutase, binding site	+ (s)	0 (s)	0 (s)	nd	nd	nd
IPR022379	11-S seed storage protein, conserved site	0	0	0	0	0	0
IPR036312	Bifunctional inhibitor/plant lipid transfer protein/seed storage helical domain superfamily	0	0	0	−	0	−
IPR036314	Manganese/iron superoxide dismutase, C-terminal domain superfamily	+ (s)	0 (s)	0 (s)	nd	nd	nd
IPR036324	Manganese/iron superoxide dismutase, N-terminal domain superfamily	+ (s)	0 (s)	0 (s)	nd	nd	nd

“+”—presence of domain associated with high score; “−“—presence of domain associated with low score; “0”—presence of domain has no defined influence on score; “nd”—no data; (s)—score for single protein.

## Data Availability

Not applicable.

## Supplementary Materials

The following supporting information can be downloaded at: https://www.mdpi.com/article/10.3390/cimb44070214/s1. Supplementary Materials contain as follows: a list of allergens described in this work with the location of epitopes (Table S1), a list of epitopes retrieved from the Immune Epitope Database (Table S2), values of ∑A, DHt and ∑AE parameters for entire proteins and their epitopes (Table S3), values of ∑A, DHt and ∑AE parameters for individual epitopes (Table S4), values of ∑A, DHt and ∑AE scores of entire protein sequences and epitopes classified according to presence of sets of domains defined according to the InterPro database (Table S5), profiles of potential biological activity of fragments of particular allergens (Tables S6–S22) and results of proteolysis simulation, obtained using the BIOPEP-UWM program (Table S23).
