# Supplementary material for: Quantitative In Silico Evaluation of Allergenic Proteins from Anacardium occidentale, Carya illinoinensis, Juglans regia and Pistacia vera and Their Epitopes as Precursors of Bioactive Peptides
Source: Curr Issues Mol Biol. 2022 Jul 6;44(7):3100–17. doi: 10.3390/cimb44070214 (PMC9317212; doi:10.3390/cimb44070214)
Supplement: Supplementary file 1 [file cimb-44-00214-s001.zip › cimb-1776659-supplementary.pdf]

Quantitative *in silico* evaluation of allergenic proteins from *Anacardium occidentale*, *Carya illinoinensis*, *Juglans regia* and *Pistacia vera* and their epitopes as precursors of bioactive epitopes.

Piotr Minkiewicz, Christopher P. Mattison, Małgorzata Darewicz

Supplement

Table S1. Allergens and their epitopes analyzed in this work

No	Allergen	UniProt / InterPro	Sequence[1, 2]	Epitopes[2]
1	Ana o 1.0101	Q8L5L5 / IPR006045, IPR014710, IPR011051	MGPPTKFSFSLFLVSVLVLCLGFALAKIDPELKQCKHQCKVQRQYDEQQKEQCVKECEKYYKEKKGRER<br>EHEEEEEEWGTGGVDEPSTHEPAEKHLSQCMRQCERQEGGQQKQLCRFRCQERYKKERGQHNYKR<br>EDDEDEDEDEAEEEDENPYVFEDEDFTTKVKTEQGKVVLLPKFTQKSKLLHALEKYRLAVLVANPQAFV<br>VPSHMDADSIFFVSWGRGTITKILENKRESINVRQGDIVSISSGTPFYIANNDENEKLYLVQFLRPVNLP<br>GHFEVFHGPGGENPESFYRAFSWEILEAALKTSKDTLEKLFEKQDQGTIMKASKEQIRAMSRRGEGPKI<br>WPFTEESTGSFKLFKKDPSQSNKYGQLFEAERIDYPPLEKLDMVVSYANITKGGMSVPFYNSRATKIAIV<br>VSGEGCVEIACPHLSSSKSSHPSYKKLRARIRKDTVFIVPAGHPFATVASGNENLEIVCFEVNAEGNIRYT<br>LAGKKNIIKVMEKEAKELAFKMEGEEVDKVFGKQDEEFFFQGPEWRKEKEGRADE	Epitope,protein,start,end<br>CKVQRQYDEQQKEQCVKECEKYYKEKKGREREHEEEEEE,Q8L5L5,39,77<br>DEAEEEDENPYVFED, Q8L5L5,145,179<br>GPGGENPESFYRAFSWEILE, Q8L5L5,281,300<br>RRGEGPKIWPFTEES,Q8L5L5,335,349<br>MVVSYANITKGGMSVPFYNSR,Q8L5L5,385,405<br>TKIAIVVSGEGCVEI,Q8L5L5,407,421<br>SSHPSYKKLRARIRKDTVFIVP,Q8L5L5,431,452<br>FGKQDEEFFFQGPEWRKEKE,Q8L5L5,514,533
2	Ana o 1.0102	Q8L5L6 / IPR006045, IPR014710, IPR011051	PPTKFSFSLFLVSVLVLCLGFALAKIDPELKQCKHQCKVQRQYDEQQKEQCVKECEKYYKEKKGREREH<br>EEEEEEWGTGGVDEPSTHEPAEKHLSQCMRQCERQEGGQQKQLCRFRCQERYKKERGQHNYKRED<br>DEDEDEDEAEEEDENPYVFEDEDFTTKVKTEQGKVVLLPKFTQKSKLLHALEKYRLAVLVANPQAFVVP<br>SHMDADSIFFVSWGRGTITKILENKRESINVRQGDIVSISSGTPFYIANNDENEKLYLVQFLRPVNLPGH<br>FEVFHGPGGENPESFYRAFSWEILEAALKTSKDTLEKLFEKQDQGTIMKASKEQVRAMSRRGEGPKIW<br>PFTEESTGSFKLFKKDPSQSNKYGQLFEAERIDYPPLEKLDMVVSYANITKGGMSVPFYNSRATKIAIVV<br>SGEGCVEIACPHLSSSKSSHPSYKKLRARIRKDTVFIVPAGHPFATVASGNENLEIVCFEVNAEGNIRYTL<br>AGKKNIIKVMEKEAKELAFKMEGEEVDKVFGKQDEEFFFQGPEWRKEKEGRADE	Epitope,protein,start,end<br>CKVQRQYDEQQKEQCVKECEKYYKEKKGREREHEEEEEE,Q8L5L6,37,75<br>DEAEEEDENPYVFED,Q8L5L6,143,177<br>GPGGENPESFYRAFSWEILE,Q8L5L6,279,298<br>RRGEGPKIWPFTEES,Q8L5L6,333,347<br>MVVSYANITKGGMSVPFYNSR,Q8L5L6,383,403<br>TKIAIVVSGEGCVEI,Q8L5L6,407,419<br>SSHPSYKKLRARIRKDTVFIVP,Q8L5L6,429,450<br>FGKQDEEFFFQGPEWRKEKE,Q8L5L6,512,531

3	Ana o 2.0101	Q8GZP6 / IPR022379, IPR006044, IPR006045, IPR014710, IPR011051	LSVCFLILFHGCLASRQEWQQQDECQIDRLDALEPDNRVEYEAGTVEAWDPNHEQFRCAGVALVRH<br>TIQPNGLLLPQYSNAPQLIYVVQGEGMTGISYPGCPETYQAPQQGRQQGQSGRFQDRHQKIRRFRR<br>GDIIAIPAGVAHWCYNEGNSPVVTVTLLDVSNSQNQLDRTPRKFHLAGNPKDVFQQQQQHQSRGR<br>NLFSGFDTELLAEAFQVDERLIKQLKSEDNRGGIVKVKDDELRVIRPSRSQSERGSESEEESEDEKRRWG<br>QRDNGIEETICTMRLKENINDPARADIYTPEVGRLTTLNSLNLPILKWLQLSVEKGVLYKNALVLPHWN<br>LNSHSIIYGCKGKGQVQVVDNFGNRVFDGEVREGQMLVVPQNFAVVKRAREERFEWISFKTNDRAM<br>TSPLAGRTSVLGGMPEEVLANAFQISREDARKIKFNNQQTTLTSGESSHHMRDDA	Epitope,protein,start,end<br>LSVCFLILFHGCLASRQEWQQQDECQIDRLDALEPDNRVEYEAGTVEA,Q8GZP6,1,48<br>QFRCAGVALVRHTIQPNGLLLPQYSNAPQLIY,Q8GZP6,55,86<br>QGEGMTGISYPGCPE,Q8GZP6,89,103<br>YQAPQQGRQQGQSGRFQDRHQKIRRFRRGDI,Q8GZP6,105,135<br>AIPAGVAHWCYNEGN,Q8GZP6,137,151<br>VFQQQQQHQSRGRNL,Q8GZP6,185,199<br>DERLIKQLKSEDNRGGIVKVKDDELRVIRPSRSQSERGSES,Q8GZP6,215,255<br>EESEDEKRRWGQRDNGIEETICTMRLKENINDPARADIYTPEVGRLTTLNSLNLPILKWLQLSVEKGVLYK<br>NALVLPHWNLNSHSIIYGCKGKGQ,Q8GZP6,257,351<br>QNFAVVKRAREERFEWISFKTNDRAMTSPLAGRTSVLGGMPEEVLANAFQISREDARKIKFN,<br>Q8GZP6,393,455
4	Ana o 3.0101	Q8H2B8 / IPR036312, IPR016140, IPR000617	MAKFLLLLSAFAVLLLVANASIYRAIVEVEEDSGREQSCQRQFEEQQRFRNCQRYVKQEVQRGGRYNQ<br>RQESLRECCQELQEVDRRCRCQNLEQMVRQLQQQEQIKGEEVRELYETASELPRICSISPSQGCQFQS<br>SY	Epitope,protein,start,end<br>SGREQSCQRQFEEQQRFRNCQRYVKQEVQRGGRYNQRQESLRECCQELQEVDRRCRC,Q8H2B8,33,8<br>9<br>LQQQEQIKGEEVRELYET,Q8H2B8,99,116<br>ICSISPSQGCQF,Q8H2B8,123,134
5	Car i 1.0101	Q84XA9 / IPR036312, IPR016140, IPR000617	MARVAALLVALLFVANAAAFRTTITTMEIDEDIDNPRRRGESCREQIQRQQYLNRCQDYLRQQCRSG<br>**GYDEDNQRQHFRQCC**QQLSQMEEQCQCEGLRQAVRQQQQEEGIRGEEMEEMVQCASDLPKECGI<br>SSRSCEIRRSWF	Epitope,protein,start,end<br>**GYDEDNQRQHFRQCC**,Q84XA9,68,82
6	Car i 2.0101	B3STU4 / IPR006045, IPR014710, IPR011051, IPR006792	MVTKAKIPLFLFLSALFLALVCSSLALETEDLSNELNPHHDPESHRWEFQQCQERCQHEERGQRQAQQ<br>CQRRCEEQLRERERERERE EIVDPREPRKQYEQCRETCEKQDPRQQPQCERRCERQFQEQQERERRE<br>RRRGRDDDDKENPRDPREQYRQCEEHCRRQGQGQRQQQQCQSRCEERFEEEQRRQEERERRRGR<br>DNDDEENPRDPREQYRQCQEHCRRQGQGQRQQQQCQSRCEERLEEEQRKQEERERRRGRDEDDQ<br>NPRDPEQR**YEQCQQQCER**QRRGQEQQLCRRRCEQQRQQEERERQRGRDRQDPQQQYHRCQRRC<br>QTQEQSPER**QRQCQQRCER**QYKEQQGREWGPDQASPRRESRGREEEQQRHNPYYFHSQGLRSRH<br>ESGEGEVKYLERFTERTELLRGIENYRVVILEANPNTFVLPYHKDAESVIVVTRGRATLTFVSQERRESFN<br>LEYGDVIRVPAGATEYVINQDSNERLEMVKLLQPVNNPGQFREYYAAGAQSTESYLRVFS**NDILVAAL**<br>**NTPRDRLERFFD**QQEQREGVIIRASQEKLRALSQHAMSAGQRPWGRRSSGGPISLKSQRSSYSNQFG<br>QFFEACPEEHRQL**QEMDVLVNYAEIKRGAMMVPHYNSKATV**VVYVVEGTGRFEMACPHDVSSQS<br>YEYKGRREQEEEESSTGQFQKVT**ARLARGDIFVIPAGHPIAIT**ASQNENLRLVGFGINGKNNQRNFLA<br>GQNNIINQLE**REAKELSFNMPREEI**EEIFERQVESYFVPMERQSRRGQGRDHPLASILDFAGFF	Epitope,protein,start,end<br>**YEQCQQQCER**,B3STU4,272,281<br>**QRQCQQRCER**,B3STU4,336,345<br>**NDILVAALNTPRDRLERFFD**,B3STU4,522,541<br>**QEMDVLVNYAEIKRGAMMVPHYNSKATV**,B3STU4,610,637<br>**ARLARGDIFVIPAGHPIAIT**,B3STU4,684,704<br>**REAKELSFNMPREEI**,B3STU4,739,754
7	Car i 4.0101	B5KVH4 / IPR022379, IPR006044, IPR006045, IPR014710, IPR011051	MAKPILLSIYLCLIIVALFNGCLAQSGGRQQHKFGQCQLNRLDALEPTNRIEAEAGVIESWDPNHQQLQ<br>CAGVAVVRRTIEPNGLLLPH**YSNAPQLVYIARGRG**I**TGVLFPGCPETFEES**QRQSQQGQR**REFQQDR**<br>**HQKIRHFR**EGDIIAFPAGVAHWCYNDGSSPVVAIFLLDTHNNANQLDQNPRNFYLAGNPDDEFRPQ<br>GQQEYEQHRRQQQHQQRRGEHGEQQRDLGNNVFSGFDAEFLADAFNVDTETARRLQSENDHRG**S**<br>**IVRVEGRQLQVIRP**RWSREEQEHEERKERERERESES**ERRQSRRGGRDD**NGLEETICTLSLRENIGDPS<br>RADIYTEEAGRISTVNSHNLPILRWLQLSAERGALYSDALYV**PHWNLNAHSVVYALRGRAEVQVV**DN<br>FGQTVFDDELREGQLLTIPQNFAVVKRARDEGFEWVSFKTNENAMVSPLAGRTSAIRALPEEVLVNAF<br>QIPREDARRLKFNRQESTLVRSRSRSSRSERRAEV	Epitope,protein,start,end<br>GRQQHKFGQCQL,B5KVH4,28,39<br>**PHYSNAPQLVYIARGRG**,B5KVH4,88,104<br>**TGVLFPGCPETFEES**QRQSQQGQR**REFQQDRHQKIRHFR**,B5KVH4,106,144<br>QGQQEYEQHRRQQQHQQR,B5KVH4,202,219<br>NNVFSGFDAEFLADAFNV,B5KVH4,232,249<br>**SIVRVEGRQLQVIRP**RWSREEQE,B5KVH4,266,288<br>**ERRQSRRGGRDD**,B5KVH4,304,315<br>**PHWNLNAHSVVYALRGRAEVQVV**,B5KVH4,378,400<br>PQNFAVVKRARDEGF,B5KVH4,421,435
8	Jug r 1.0101	P93198 / IPR036312, IPR016140, IPR000617	AALLVALLFVANAAAFRTTITTMEIDEDIDNPRRRGEGCREQIQRQQNLNHCQYYLRQQSRSG**GYDE**<br>**DNQRQHFRQCC**QQLSQMDEQCQCEGLRQVVRRQQQQQGLRGEEMEEMVQSARDLPNECGISSQ<br>RCEIRRSWF	Epitope,protein,start,end<br>EQIQRQQNLNHCQYYLRQQS,P93198,41,60<br>**GYDEDNQRQHFRQCC**,P93198,64,78<br>VVRRQQQQQGLRGEEMEEMVQS,P93198,96,117

9	Jug r 2.0101	Q9SEW4 / IPR006045, IPR014710, IPR011051, IPR006792	RGRDDDDEENPRDPREQYRQCQEYCRRQGQGQRQQQQCQIRCEERLEEDQRSQEERERRRGRDVD<br>DQNPRDPEQR**YEQCQQQCER**QRRGQEQTLCRRRCEQRRQQEERERQRGRDRQDPQQQYHRCQR<br>RCQIQEQSPER**QRQCQQRCER**QYKEQQGRERGPEASPRRESRGREEEQQRHNPYYFHSQSIRSRHES<br>EEGEVKYLERFTERTELLRGIENYRVVILDANPNTSMLPHHKDAESVAVVTRGRATLTLVSQETRESFNL<br>ECGDVIRVPAGATVYVINQDSNERLEMVKLLQPVNNPGQFREYYAAGAKSPDQSYLRVFS**NDILVAAL**<br>**NTPRDRLERFFD**QQEQREGVIIRASQEKLRALSQHAMSAGQRPWGRRSSGGPISLKSESPSYSNQFG<br>QFFEACPEEHRQL**QEMDVLVNYAEIKRGAMMVPHYNSKATV**VVYVVEGTGRYEMACPHVSSQSYE<br>GQGRREQEEEESTGRFQKVTA**RLARGDIFVIPAGHPIAIT**ASQNENLRLLGFDINGENNQRDFLAGQN<br>NIINQLE**REAKELSFNMPREEI**EEIFESQMESYFVPTERQSRRGQGRDHPLASILDFAFF	Epitope,protein,start,end<br>DQRSQEERER,Q9SEW4,49,58<br>**YEQCQQQCER**,Q9SEW4,76,85<br>QRRQQEERER,Q9SEW4,101,110<br>**QRQCQQRCER**,Q9SEW4,140,149<br>EQQRHNPYYFHSQSIRSRH,Q9SEW4,175,193<br>FTERTELLRGIENYRVVILDANPNTSMLPHHKDAESVAVVTRGRATLTLVSQETRESFNL,Q9SEW4,206,<br>265<br>PGQFREYYAAGAKSPDQSYLRVFS**NDILVAALNTPRDRLERFFD**,Q9SEW4,302,345<br>SGGPISLKSESPSYSNQFGQ,Q9SEW4,382,401<br>**QEMDVLVNYAEIKRGAMMVPHYNSKATV**VVYVVEGTGRYEMACP,Q9SEW4,414,457<br>SYEGQGRREQEEEESTGRFQKVT**ARLARGDIFVIPAGHPIAIT**,Q9SEW4,463,505<br>**REAKELSFNMPREEI**EEIFESQMESYFVPTERQSRRG,Q9SEW4,541,577
10	Jug r 3	C5H617 / IPR036312, IPR016140, IPR000528	MTGSLVLKLSGMVLLCMVVAAPVAEAVITCGQVASSVGSCIGYLRGTVPTVPPSCCNGVKSLNKAAAT<br>TADRQAACECLKKTSGSIPGLNPGLAAGLPGKCGVSVPYKISTSTNCKAVK	
11	Jug r 4.0101	Q2TPW5 / IPR022379, IPR006044, IPR006045, IPR014710, IPR011051	MAKPILLSIYLFLIVALFNGCLAQSGGRQQQQFGQCQLNRLDALEPTNRIEAEAGVIESWDPNNQQFQ<br>CAGVAVVRRTIEPNGLLLPQ**YSNAPQLVYIARGRG**I**TGVLFPGCPETFEES**QRQSQQGQS**REFQQDR**<br>**HQKIRHFR**EGDIIAFPAGVAHWSYNDGSNPVVAISLLDTNNNANQLDQNPRNFYLAGNPDDEFRPQ<br>GQQEYEQHRRQQQRQQRPGEHGQQQRGLGNNVFSGFDADFLADAFNVDTETARRLQSENDHRR**S**<br>**IVRVEGRQLQVIRP**RWSREEQEREERKERERERESES**ERRQSRRGGRDD**NGLEETICTLRLRENIGDPS<br>RADIYTEEAGRISTVNSHTLPVLRWLQLSAERGALYSDALYV**PHWNLNAHSVVYALRGRAEVQVV**DN<br>FGQTVFDDELREGQLLTIPQNFAVVKRARNEGFEWVSFKTNENAMVSPLAGRTSAIRALPEEVLATAF<br>QIPREDARRLKFNRQESTLVRSRPSRSRSSRSERRAEV	Epitope,protein,start,end<br>MAKPILLSIYLFLIV,Q2TPW5,1,15<br>IESWDPNNQQFQCAG,Q2TPW5,57,71<br>**YSNAPQLVYIARGRG**,Q2TPW5,89,103<br>**TGVLFPGCPETFEES**,Q2TPW5,105,119<br>RQSQQGQS**REFQQDRHQKIRHFR**,Q2TPW5,121,143<br>GDIIAFPAGVAHWSY,Q2TPW5,145,159<br>QGQQEYEQHRRQQQRQQRPGEHG,Q2TPW5,201,223<br>**VFSGFDADFLADAFN**,Q2TPW5,233,247<br>QSENDHRR**SIVRVEGRQLQVIRP**RWSREEQEREERKERE,Q2TPW5,257,295<br>**ERRQSRRGGRDD**NGLEETICTLRLR,Q2TPW5,303,327<br>**PHWNLNAHSVVYALRGRAEVQVV**,Q2TPW5,377,399<br>LTIPQNFAVVKRARNEGFEWVSF,Q2TPW5,417,439<br>LATAFQIPREDARRL,Q2TPW5,465,479<br>FNRQESTLVRSRPSRSRSSRSER,481,503
12	Pis v 1.0101	B7P072 / IPR036312, IPR016140, IPR000617	MAKLVLLLSAFAFLILAANASIYRATVEVEGENLSSGQSCQKQFEEQQKFKHCQMYVQQEVQKSQDG<br>HSLTARINQRQQCFKQCCQELQEVDKKCRCQNLEQMVKRQQQQGQFRGEKLQELYETASELPRMC<br>NISPSQGCQFSSPYWSY	
13	Pis v 2.0101	B7P073 / IPR022379, IPR006044, IPR006045, IPR014710, IPR011051	MGYSSLLSFSLGFLLLFHCSFAQIEQVVNSQQRQQQQRFQTQCQIQNLNALEPKRRIESEAGVTEFWD<br>QNEEQLQCANVAVFRHTIQSRGLLVPSYDNAPELVYVVQGSGIHGAVFPGCPETFQEESQSRSRSERS<br>QQSGEQHQKVRPIQEGDVIALPAGVAHWIYNNGQSKLVLVALADVGNSENQLDQYLRKFVLGGSPQ<br>QEIQGSGQSRSRSQSQSSRRGQQGQQSNNILSAFDEEILAQSFNIDTQLARRLQKEKRQRGIIVRVQE<br>DLEVLSPHRQEQEQEYEEERERRQRNGLEETFCTMTLKYNINDPSRADVYNPRGGRVSSVNALNLPIL<br>RFLQLSAKKGVLHRDAILAPHWNVNAHSIVYITRGNGRIQIVSENGESVFDEEIREGQLVVVPQNFAVV<br>KRASSDKFEWVSFKTNGLSQTSQLAGRVSVFRALPLDVIKNSFDISREDARRLKESRSETTIFAPGSSSQR<br>KSQSERERQREREEREIH	

14	Pis v 2.0201	B7P074 / IPR022379, IPR006044, IPR006045, IPR014710, IPR011051	MGYSSLLSFSLGFLLLFHCSFAQIEQVVNSQQRQQQQRFQTQCQIQNLNALEPKRRIESEAGVTEFWD QNEEQLQCANVAVFRHTIQSRGLLVPSYNNAPELVYVVQGSGIHGAVFPGCPETFQEESQSQSRSQH SRSERSQQSGEQHQKVRHIREGDIIALPAGVAHWIYNNGQSKLVLVALADVGNSENQLDQYLRKFVL GGSPQQEIQGGGQSWSQSRSSRKGQQSNNILSAFDEEILAQSFNIDTQLVKKLQREEKQRGIIVRVKE DLQVLSPQRQEKEYSDNGLEETFCTMTLKLNINDPSRADVYNPRGGRVTSINALNLPILRFLQLSVEKG VLYQNAIMAPHWNMNAHSIVYITRGNGRMQIVSENGESVFDEEIREGQLVVVPQNFAVVKRASSDG FEWVSFKTNGLAKISQLAGRISVMRGLPLDVIQNSFDISREDAWNLKESRSEMTIFAPGSRSQRQRN	
15	Pis v 3.0101	B4X640 / IPR006045, IPR014710, IPR011051	KTDPELKQCKHQCKVQRQYDEEQKEQCAKGCEKYYKEKKGREQEEEEEEEWGSGRGRGDEFSTHEP GEKRLSQCMKQCERQDGGQQKQLCRFRCQEKYKKERREHSYSRDEEEEEEGDEEQEEEDENPYVFED EHFTTRVKTEQGKVVVLPKFTKRSKLLRGLEKYRLAFLVANPQAFVVPNHMDADSIFFVSWGRGTITKI RENKRESMNVKQGDIIRIRAGTPFYIVNTDENEKLYIVKLLQPVNLPGHYEVFHGPGGENPESFYRAFS REVLEAALKTPRDKLEKLFEKQDEGAIVKASKEQIRAMSRRGEGPSIWPFTGKSTGTFNLFKKDPSQSN NYGQLFESEFKDYPPLQELDIMVSYVNITKGGMSGPFYNSRATKIAIVVSGEGRLEIACPHLSSSKNSGQ EKSGPSYKKLSSSIRTDSVFVVPAGHPFVTVASGNQNLEILCFEVNAEGNIRYTLAGKKNIIEVMEKEAKE LAFKTKGEEVDKVFGKQDEEFFFQGPKWRQHQQGRADE	Epitope,protein,start,end<br>GPGGENPESFYRAFSREVLE,B4X640,257,276<br>VFGKQDEEFFFQGPKWRQH,B4X640,494,512
16	Pis v 4.0101	B2BDZ8 / IPR001189, IPR019833, IPR019832, IPR019831, IPR036324, IPR036314	MALLSYVTRKTLTESLRLGLKSHVRGLQTFTLPDLPYEYGALEPAISSEIMQLHHQKHHQTYITNYNKAL EQLDQAINKGDASAVVKLQSAIKFNGGGHINHSIFWKNLTPVSEGGGEPPHGSLGWAIDTNFGSMEA LIQRMNAEGAALQGSGWVWLGLDKESKKLVVETTANQDPLVTKGPSLVPLLGIDVWEHAYYLQYKN VRPDYLKNIWKVINWKYAGELYQKECP	
17	Pis v 5.0101	B7SLJ1 / IPR006044, IPR006045, IPR014710, IPR011051	MANPSLLSLSVCLLILFHGCLASRQQGQQQNECQIDQLDALEPDNRVEYEAGMVETWDPNHEQFRC AGVAVARHTIQPNGLRLPEYSNAPTLMYIVEGEGMTGTLIPGCPETYQAPQQGQQHGQSSRFQDKH QKIQRFRKGDIIALPAGVANWCYNEGNSPVVTVTLLDVSNSQNQLDMYPRKFNLAGNPEDEFQQQQ QQQSRGRRQSQQKSCNNIFCGFDTKILAEVFQVEQSLVKQLQNEKDNRGAIVKVKGDLQVIRPPRRQ SERGFESEEESEYERGRRGRDNGLEETICTMKLKENIHDPSRSDIYTPEVGRITSLNSLNLPILKWLQLSA ERGVLQNNALMVPHWNFNAHSIVYGCKGNAQVQVVDNFGNTVFDGEVSEGQIFVVPQNFAVVKR ARGQRFEWISFKTNDRAMISPLAGSTSVLRAMPEEVLANAFQISREDARKIKFNNEQPTLSSGQSSQQ MRDDA	Epitope,protein,start,end<br>KVKGDLQVIRPPRRQSERG,B7SLJ1,251,269<br>PEVGRITSLNSLNLPILKWL,B7SLJ1,313,332

1. Epitopes indicated using red font.
2. Green background indicates epitopes with highest ΣA (range 1.6000-2.1000), blue background indicates epitopes with lowest ΣA (range 0.1000-0.5999), bold font – fragments occurring in proteins from *Juglans regia* and their homologs from *Carya illinoinensis*

Table S2. Sequences of epitopes, retrieved from the IEDB database and taken into account in this work

ID	Epitope	Antigen	Organism
137395	EESEDEKRRWGQRDN	Ana o 2	Anacardium occidentale (maranon)
2023	AIMGPPTKFSFSLFL	Ana o 1	Anacardium occidentale (maranon)
6532	CKVQRQYDEQQKEQC	Ana o 1	Anacardium occidentale (maranon)
6897	CQRQFEEQQRFR	Ana o 3	Anacardium occidentale (maranon)
7902	DEAEEEDENPYVFED	Ana o 1	Anacardium occidentale (maranon)
11234	ECCQELQEVDRR	Ana o 3	Anacardium occidentale (maranon)
11615	EEFFFQGPEWRKEKE	Ana o 1	Anacardium occidentale (maranon)
12729	EKKGREREHEEEEEE	Ana o 1	Anacardium occidentale (maranon)
13908	EQQKEQCVKECEKYY	Ana o 1	Anacardium occidentale (maranon)
13911	EQQRFRNCQRYV	Ana o 3	Anacardium occidentale (maranon)
25525	ICSISPSQGCQF	Ana o 3	Anacardium occidentale (maranon)
26794	IKGEEVRELYET	Ana o 3	Anacardium occidentale (maranon)
30318	KECEKYYKEKKGRER	Ana o 1	Anacardium occidentale (maranon)
32975	KQEVQRGGRYNQ	Ana o 3	Anacardium occidentale (maranon)
38962	LQQQEQIKGEEV	Ana o 3	Anacardium occidentale (maranon)
44369	NITKGGMSVPFYNSR	Ana o 1	Anacardium occidentale (maranon)
50648	QELQEVDRRCRC	Ana o 3	Anacardium occidentale (maranon)
50677	QEQIKGEEVREL	Ana o 3	Anacardium occidentale (maranon)
50752	QFEEQQRFRNCQ	Ana o 3	Anacardium occidentale (maranon)
53773	RFRNCQRYVKQE	Ana o 3	Anacardium occidentale (maranon)
55412	RQESLRECCQEL	Ana o 3	Anacardium occidentale (maranon)
55567	RRGEGPKIWPFTEES	Ana o 1	Anacardium occidentale (maranon)
56675	RYVKQEVQRGGR	Ana o 3	Anacardium occidentale (maranon)
58215	SGREQSCQRQFE	Ana o 3	Anacardium occidentale (maranon)
59430	SLRECCQELQEV	Ana o 3	Anacardium occidentale (maranon)
61072	SSHPSYKKLRARIRK	Ana o 1	Anacardium occidentale (maranon)
64582	TKIAIVVSGEGCVEI	Ana o 1	Anacardium occidentale (maranon)
75208	YNQRQESLRECC	Ana o 3	Anacardium occidentale (maranon)
137514	AIPAGVAHWCYNEGN	Ana o 2	Anacardium occidentale (maranon)
137516	ALVLPHWNLNSHSII	Ana o 2	Anacardium occidentale (maranon)
137519	AREERFEWISFKTND	Ana o 2	Anacardium occidentale (maranon)
137527	FHGCLASRQEWQQQD	Ana o 2	Anacardium occidentale (maranon)
137528	FQISREDARKIKFNN	Ana o 2	Anacardium occidentale (maranon)
137539	KVKDDELRVIRPSRS	Ana o 2	Anacardium occidentale (maranon)

137540	LDRTPRKFHLAGNPK	Ana o 2	Anacardium occidentale (maranon)
137542	LKWLQLSVEKGVLYK	Ana o 2	Anacardium occidentale (maranon)
137544	LNSHSIIYGCKGKGQ	Ana o 2	Anacardium occidentale (maranon)
137546	LSVCFLILFHGCLAS	Ana o 2	Anacardium occidentale (maranon)
137551	PEEVLANAFQISRED	Ana o 2	Anacardium occidentale (maranon)
137554	QDRHQKIRRFRRGDI	Ana o 2	Anacardium occidentale (maranon)
137555	QEWQQQDECQIDRLD	Ana o 2	Anacardium occidentale (maranon)
137556	QGEGMTGISYPGCPE	Ana o 2	Anacardium occidentale (maranon)
137557	QNFAVVKRAREERFE	Ana o 2	Anacardium occidentale (maranon)
137558	QQGQSGRFQDRHQKI	Ana o 2	Anacardium occidentale (maranon)
137566	RLIKQLKSEDNRGGI	Ana o 2	Anacardium occidentale (maranon)
137576	SRQEWQQQDECQIDR	Ana o 2	Anacardium occidentale (maranon)
137579	VFQQQQQHQSRGRNL	Ana o 2	Anacardium occidentale (maranon)
137580	VIRPSRSQSERGSES	Ana o 2	Anacardium occidentale (maranon)
137586	YQAPQQGRQQGQSGR	Ana o 2	Anacardium occidentale (maranon)
151824	DALEPDNRVEYEAGTVEA	Ana o 2	Anacardium occidentale (maranon)
152103	RWGQRDNGIEETICTMRLKENINDP	Ana o 2	Anacardium occidentale (maranon)
186822	ALEPDNRVEY	Ana o 2	Anacardium occidentale (maranon)
186832	DERLIKQLKSEDNRGGIVKVKD	Ana o 2	Anacardium occidentale (maranon)
186846	FKTNDRAMTSPLAGRTSVLGGM	Ana o 2	Anacardium occidentale (maranon)
186887	LIKQLKSEDNRGGIVK	Ana o 2	Anacardium occidentale (maranon)
186888	LIKQLKSEDNRGGIVKVKDD	Ana o 2	Anacardium occidentale (maranon)
186889	LIKQLKSEDNRGGIVKVKDDEL	Ana o 2	Anacardium occidentale (maranon)
186903	NDRAMTSPLAGRTSVLG	Ana o 2	Anacardium occidentale (maranon)
186919	QFRCAGVALVRHTIQ	Ana o 2	Anacardium occidentale (maranon)
186930	SEDNRGGIVKVKDD	Ana o 2	Anacardium occidentale (maranon)
186950	VEYEAGT	Ana o 2	Anacardium occidentale (maranon)
186956	VRHTIQPNGLLLPQYSNAPQLIY	Ana o 2	Anacardium occidentale (maranon)
606390	ALVLPHWNLNSHSIIYGCKG	Ana o 2	Anacardium occidentale (maranon)
606416	EKGVLYKNALVLPHWNLNSH	Ana o 2	Anacardium occidentale (maranon)
606445	GPGGENPESFYRAFSWEILE	Ana o 1	Anacardium occidentale (maranon)
606453	HPSYKKLRARIRKDTVFIVP	Ana o 1	Anacardium occidentale (maranon)
606467	KVKDDELRVIRPSRSQSERG	Ana o 2	Anacardium occidentale (maranon)
606495	MVVSYANITKGGMSVPFYNS	Ana o 1	Anacardium occidentale (maranon)
606499	PARADIYTPEVGRLTTLNSL	Ana o 2	Anacardium occidentale (maranon)
606501	PEVGRLTTLNSLNLPILKWL	Ana o 2	Anacardium occidentale (maranon)
606512	QNFAVVKRAREERFEWISFK	Ana o 2	Anacardium occidentale (maranon)

606540	VFGKQDEEFFFQGPEWRKEK	Ana o 1	Anacardium occidentale (maranon)
157186	EESQRQSQQGQR	Car i 4	Carya illinoinensis (pecan)
157218	EQHRRQQQHQQR	Car i 4	Carya illinoinensis (pecan)
157220	ERRQSRRGGRDD	Car i 4 and Jug r 4	Carya illinoinensis (pecan)
157228	FAVVKRARDEGF	Car i 4	Carya illinoinensis (pecan)
157230	FDAEFLADAFNV	Car i 4	Carya illinoinensis (pecan)
157280	FSGFDAEFLADA	Car i 4	Carya illinoinensis (pecan)
157340	GQRREFQQDRHQ	Car i 4	Carya illinoinensis (pecan)
157343	GRQQHKFGQCQL	Car i 4	Carya illinoinensis (pecan)
157381	HSVVYALRGRAE	Car i 4 and Jug r 4	Carya illinoinensis (pecan)
157603	LNAHSVVYALRG	Car i 4 and Jug r 4	Carya illinoinensis (pecan)
157725	NNVFSGFDAEFL	Car i 4	Carya illinoinensis (pecan)
157741	PHYSNAPQLVYI	Car i 4	Carya illinoinensis (pecan)
157762	PQNFAVVKRARD	Car i 4	Carya illinoinensis (pecan)
157770	QEYEQHRRQQQH	Car i 4	Carya illinoinensis (pecan)
157773	QGQQEYEQHRRQ	Car i 4	Carya illinoinensis (pecan)
157808	QQDRHQKIRHFR	Car i 4 and Jug r 4	Carya illinoinensis (pecan)
157812	QRQSQQGQRREF	Car i 4	Carya illinoinensis (pecan)
157835	REFQQDRHQKIR	Car i 4 and Jug r 4	Carya illinoinensis (pecan)
158291	SQQGQRREFQQD	Car i 4	Carya illinoinensis (pecan)
114650	VFSGFDADFLADAFN	Jug r 4	Juglans regia (English walnut)
21102	GLRGEEMEEMVQS	Jug r 1	Juglans regia (English walnut)
50901	QGLRGEEMEEMV	Jug r 1	Juglans regia (English walnut)
52113	QQQGLRGEEMEEM	Jug r 1	Juglans regia (English walnut)
55473	RQQQQQGLRGEEM	Jug r 1	Juglans regia (English walnut)
71816	VVRRQQQQQGLRG	Jug r 1	Juglans regia (English walnut)
114293	DDNGLEETICTLRLR	Jug r 4	Juglans regia (English walnut)
114339	FNRQESTLVRSRPSR	Jug r 4	Juglans regia (English walnut)
114351	GDIIAFPAGVAHWSY	Jug r 4	Juglans regia (English walnut)
114396	HRRQQQRQQRPGEHG	Jug r 4	Juglans regia (English walnut)
114404	IESWDPNNQQFQCAG	Jug r 4	Juglans regia (English walnut)
114442	LATAFQIPREDARRL	Jug r 4	Juglans regia (English walnut)
114462	LTIPQNFAVVKRARN	Jug r 4	Juglans regia (English walnut)
114468	MAKPILLSIYLFLIV	Jug r 4	Juglans regia (English walnut)
114508	PHWNLNAHSVVYALR	Jug r 4 and Car i 4	Juglans regia (English walnut)
114531	QGQQEYEQHRRQQQR	Jug r 4	Juglans regia (English walnut)
114543	QLQVIRPRWSREEQE	Jug r 4	Juglans regia (English walnut)

114555	QSENDHRRSIVRVEG	Jug r 4	Juglans regia (English walnut)
114569	REFQQDRHQKIRHFR	Jug r 4 and Car i 4	Juglans regia (English walnut)
114590	RQSQQGQSREFQQDR	Jug r 4	Juglans regia (English walnut)
114610	SIVRVEGRQLQVIRP	Jug r 4 and Car i 4	Juglans regia (English walnut)
114620	SVVYALRGRAEVQVV	Jug r 4	Juglans regia (English walnut)
114627	TGVLFPGCPETFEES	Jug r 4 and Car i 4	Juglans regia (English walnut)
114666	VRSRPSRSRSSRSER	Jug r 4	Juglans regia (English walnut)
114671	VVKRARNEGFEWVSF	Jug r 4	Juglans regia (English walnut)
114676	WSREEQEREERKERE	Jug r 4	Juglans regia (English walnut)
114695	YSNAPQLVYIARGRG	Jug r 4 and Car i 4	Juglans regia (English walnut)
157171	DQRSQEERER	Jug r 2	Juglans regia (English walnut)
157811	QRQCQQRCER	Jug r 2 and Car i 2	Juglans regia (English walnut)
157813	QRRQQEERER	Jug r 2	Juglans regia (English walnut)
158326	SYEGQGRR	Jug r 2	Juglans regia (English walnut)
158509	YEQCQQQCER	Jug r 2 and Car i 2	Juglans regia (English walnut)
174135	GYDEDNQRQHFRQCC	Jug r 1 and Car i 1	Juglans regia (English walnut)
174256	REAKELSFNMPREEI	Jug r 2 and Car i 2	Juglans regia (English walnut)
241140	AAGAKSPDQSYLRVFSNDIL	Jug r 2	Juglans regia (English walnut)
241161	ARLARGDIFVIPAGHPIAIT	Jug r 2 and Car i 2	Juglans regia (English walnut)
241183	EAKELSFNMPREEIEEIFES	Jug r 2	Juglans regia (English walnut)
241188	EQIQRQQNLNHCQYYLRQQS	Jug r 1	Juglans regia (English walnut)
241196	FTERTELLRGIENYRVVILD	Jug r 2	Juglans regia (English walnut)
241281	KATVVVYVVEGTGRYEMACP	Jug r 2	Juglans regia (English walnut)
241344	NDILVAALNTPRDRLERFFD	Jug r 2 and Car i 2	Juglans regia (English walnut)
241350	PGQFREYYAAGAKSPDQSYL	Jug r 2	Juglans regia (English walnut)
241355	QEMDVLVNYAEIKRGAMMVP	Jug r 2 and Car i 2	Juglans regia (English walnut)
241367	QSYLRVFSNDILVAALNTPR	Jug r 2	Juglans regia (English walnut)
241369	REQEEEESTGRFQKVTARLA	Jug r 2	Juglans regia (English walnut)
241371	RGIENYRVVILDANPNTSML	Jug r 2	Juglans regia (English walnut)
241538	TGRFQKVTARLARGDIFVIP	Jug r 2	Juglans regia (English walnut)
241541	TRGRATLTLVSQETRESFNL	Jug r 2	Juglans regia (English walnut)
241546	TSMLPHHKDAESVAVVTRGR	Jug r 2	Juglans regia (English walnut)
241557	YAEIKRGAMMVPHYNSKATV	Jug r 2 and Car i 2	Juglans regia (English walnut)
535661	EQQRHNPYYFHSQSIRSRH	Jug r 2	Juglans regia (English walnut)
536236	IFESQMESYFVPTERQSRRG	Jug r 2	Juglans regia (English walnut)
537779	SGGPISLKSESPSYSNQFGQ	Jug r 2	Juglans regia (English walnut)
606421	ERGVLQNNALMVPHWNFNAS	Pis v 5	Pistacia vera (pistachio)

606444	GPGGENPESFYRAFSREVLE	Pis v 3	Pistacia vera (pistachio)
606468	KVKGDLQVIRPPRRQSERG	Pis v 5	Pistacia vera (pistachio)
606500	PEVGRITSLNSLNLPILKWL	Pis v 5	Pistacia vera (pistachio)
606541	VFGKQDEEFFFQGPKWRQH	Pis v 3	Pistacia vera (pistachio)

Red font – epitopes found in proteins both from *Carya illinoinensis* and from *Juglans regia*

Blue font – epitopes not found in protein sequences

Table S3. Values of ΣA, DH_t and $ΣA_E$ parameters for entire proteins and their epitopes

Protein	Set of domains	ΣA for entire proteins	ΣA for epitopes (Mean ±SD)	Sequence coverage of epitopes [%]	DH_t for entire proteins [%]	DH_t for epitopes [%] (Mean ±SD)	$ΣA_E$ for entire proteins	$ΣA_E$ for epitopes (Mean ±SD)
Ana o 1.0101 (Q8L5L5)	3a	1.3571	1.4342 ±0.3830 (n = 8)	31.04	81.7505	81.2892 ±10.0853 (n = 8)	0.2176	0.2831 ±0.2283 (n = 8)
Ana o 1.0102 (Q8L5L6)	3a	1.3360	1.4342 ±0.3830 (n = 8)	31.16	79.4393	81.2892 ±10.0853 (n = 8)	0.2126	0.2831 ±0.2283 (n = 8)
Ana o 2.0101 (Q8GZP6)	3a	1.3657	1.3930 ±0.2400 (n = 9)	79.21	79.1667	77.6598 ±7.9794 (n = 9)	0.2276	0.2368 ±0.0795 (n = 9)
Ana o 3.0101 (Q8H2B8)	1	1.1012	0.9011 ±0.2867 (n = 3)	63.04	76.6423	68.3187 ±27.9529 (n = 3)	0.1739	0.0722 ±0.0625 (n = 3)
Car i 1.0101 (Q84XA9)	1	1.0911	0.7333	10.49	77.4648	92.8571	0.1539	0.0000
Car i 2.0101 (B3STU4)	2	1.1643	1.0004 ±0.5244 (n = 6)	13.01	82.6802	79.4904 ±4.5504 (n = 6)	0.1504	0.1710 ±0.1472 (n = 6)
Car i 4.0101 (B5KVH4)	3b	1.3604	1.3991 ±0.2643 (n = 9)	35.05	81.1508	85.4056 ±9.0801 (n = 9)	0.1880	0.2421 ±0.2241 (n = 9)
Jug r 1.0101 (P93198)	1	1.0721	1.0490 ±0.2574 (n = 3)	41.01	80.4348	90.9357 ±1.7379 (n = 3)	0.1152	0.0621 ±0.0689 (n = 3)
Jug r 2.0101 (Q9SEW4)	2	1.2277	0.9820 ±0.4892 (n = 11)	51.71	81.0811	79.7509 ±10.0932 (n = 11)	0.1923	0.1571 ±0.1423 (n = 11)
Jug r 3 (C5H617)	-	1.4369	No data	No data	66.1017	No data	0.2520	No data
Jug r 4.0101 (Q2TPW5)	3b	1.3668	1.3844 ±0.2803 (n = 14)	54.04	81.0277	79.8594 ±9.7549 (n = 14)	0.2070	0.2479 ±0.1594 (n = 14)
Pis v 1.0101 (B7P072)	1	1.1409	No data	No data	76.3514	No data	0.1744	No data
Pis v 2.0101 (B7P073)	3b	1.2942	No data	No data	75.9596	No data	0.2398	No data
Pis v 2.0201 (B7P074)	3b	1.3178	No data	No data	75.7962	No data	0.2604	No data
Pis v 3.0101 (B4X640)	3a	1.3833	1.6855 ±0.2646 (n = 2)	7.51	80.5019	81.2866 ±10.7514 (n = 2)	0.2506	0.3790 ±0.3127 (n = 2)
Pis v 4.0101 (B2BDZ8)	-	1.4345	No data	No data	80.3493	No data	0.1867	No data
Pis v 5.0101 (B7SLJ1)	3b	1.2749	1.4394 ±0.1971 (n = 2)	8.25	77.3305	71.1111 ±1.5713 (n = 2)	0.2008	0.4248 ±0.0054 (n = 2)
Mean ±SD		1.2779 ±0.1218			78.4252 ±3.8609		0.2002 ±0.0397	

Table S4. Values of ΣA, DH_t and $ΣA_E$ parameters for all epitopes

No	Set of domains	Fragment	ΣA	DH_t	$ΣA_E$
1	3a	281-300 of Ana o 1.0101; 279-298 of Ana o 1.0102	2.1000	84.2105	0.7000
2	3a	257-276 of Pis v 3.0101	1.9500	73.6842	0.6000
3	3a	335-349 of Ana o 1.0101; 333-347 of Ana o 1.0102	1.8002	78.5714	0.3334
4	3a	89-103 of Ana o 2.0101	1.7334	64.2857	0.3335
5	3b	88-104 of Car i 4.0101	1.7058	68.7500	0.5882
6	3b	378-400 of Car i 4.0101; 377-399 of Jug r 4.0101	1.6958	86.3636	0.3044
7	3b	233-247 of Jug r 4.0101	1.6666	85.7143	0.2000
8	3a	55-86 of Ana o 2.0101	1.6561	70.9677	0.3437
9	3b	417-439 of Jug r 4.0101	1.6088	76.1905	0.0910
10	3b	105-119 of Jug r 4.0101	1.6001	71.4286	0.4668
11	3a	514-533 of Ana o 1.0101; 512-531 of Ana o 1.0102	1.6000	94.7368	0.1000
12	3b	421-435 of Car i 4.0101	1.6000	85.7143	0.2666
13	3b	89-103 of Jug r 4.0101	1.6000	71.4286	0.5334
14	3a	431-452 of Ana o 1.0101; 429-450 of Ana o 1.0102	1.5911	66.6667	0.4547
15	3a	105-135 of Ana o 2.0101	1.5808	90.0000	0.2582
16	3b	251-269 of Pis v 5.0101	1.5788	72.2222	0.4210
17	3b	232-249 of Car i 4.0101	1.5558	88.2353	0.1667
18	2	684-704 of Car i 2.0101	1.5500	73.6842	0.2500
19	3b	145-159 of Jug r 4.0101	1.5334	71.4286	0.4000
20	3b	465-479 of Jug r 4.0101	1.5334	85.7143	0.0000
21	2	463-505 of Jug r 2.0101	1.4885	80.9524	0.2093
22	2	302-345 of Jug r 2.0101	1.4773	79.0698	0.3407
23	3a	137-151 of Ana o 2.0101	1.4667	71.4286	0.2000
24	3b	1-15 of Jug r 4.0101	1.4667	85.7143	0.3334
25	3a	257-351 of Ana o 2.0101	1.4632	80.8511	0.2527
26	3b	106-144 of Car i 4.0101	1.4613	81.5789	0.3074
27	2	414-457 of Jug r 2.0101	1.4545	79.0698	0.2499
28	3b	266-288 of Car i 4.0101	1.4349	76.1905	0.5457
29	3a	494-512 of Pis v 3.0101	1.4209	88.8889	0.1579
30	2	610-637 of Car i 2.0101	1.3928	77.7778	0.1428
31	2	206-265 of Jug r 2.0101	1.3667	83.0508	0.2168
32	1	96-117 of Jug r 1.0101	1.3637	90.4762	0.1364

33	3b	121-143 of Jug r 4.0101	1.3480	86.3636	0.2175
34	2	739-754 of Car i 2.0101	1.3334	85.7143	0.3334
35	3b	201-223 of Jug r 4.0101	1.3045	95.4545	0.2175
36	2	382-401 of Jug r 2.0101	1.3000	57.8947	0.1000
37	3b	313-332 of Pis v 5.0101	1.3000	70.0000	0.4285
38	3a	393-455 of Ana o 2.0101	1.2857	81.9672	0.1290
39	1	99-116 of Ana o 3.0101	1.2779	88.2353	0.1112
40	3a	407-421 of Ana o 1.0101; 405-419 of Ana o 1.0102	1.2668	71.4286	0.3333
41	3b	303-327 of Jug r 4.0101	1.2000	87.5000	0.0000
42	3a	39-77 of Ana o 1.0101; 37-75 of Ana o 1.0102	1.1538	86.8421	0.0769
43	2	541-577 of Jug r 2.0101	1.1622	75.0000	0.2432
44	2	522-541 of Car i 2.0101	1.1500	84.2105	0.3000
45	3a	215-255 of Ana o 2.0101	1.1465	75.0000	0.2928
46	3b	257-295 of Jug r 4.0101	1.1281	84.2105	0.3331
47	3a	1-48 of Ana o 2.0101	1.1249	78.7234	0.1875
48	3a	385-405 of Ana o 1.0101; 383-403 of Ana o 1.0102	1.0952	75.0000	0.0000
49	3b	28-39 of Car i 4.0101	1.0832	90.9091	0.0000
50	3b	202-219 of Car i 4.0101	1.0557	100.0000	0.0000
51	2	175-193 of Jug r 2.0101	1.0525	77.7778	0.3683
52	1	41-60 of Jug r 1.0101	1.0500	89.4737	0.0500
53	3b	304-315 of Car i 4.0101	1.0001	90.9091	0.0000
54	3a	185-199 of Ana o 2.0101	1.0000	85.7143	0.1334
55	3b	57-71 of Jug r 4.0101	1.0000	71.4286	0.2000
56	3a	145-179 of Ana o 1.0101; 143-177 of Ana o 1.0102	0.8667	92.8571	0.2667
57	1	33-89 of Ana o 3.0101	0.8420	80.3571	0.1053
58	1	68-82 of Car i 1.0101; 64-78 of Jug r 1.0101	0.7333	92.8571	0.0000
59	3b	481-503 of Jug r 4.0101	0.6957	59.0909	0.1740
60	2	101-110 of Jug r 2.0101	0.6000	100.0000	0.0000
61	1	123-134 of Ana o 3.0101	0.5833	36.3636	0.0000
62	2	272-281 of Car i 2.0101; 76-85 of Jug r 2.0101	0.5000	77.7778	0.0000
63	2	49-58 of Jug r 2.0101	0.3000	88.8889	0.0000
64	2	336-345 of Car i 2.0101; 140-149 of Jug r 2.0101	0.1000	77.7778	0.0000
		**Mean**	**1.2896**	**80.1683**	**0.2267**
		**SD**	**±0.3807**	**±10.6456**	**±0.1713**

Table S5. Values of ΣA, DH_t and ΣA_E scores of entire protein sequences and epitopes classified according to presence of sets of domains defined according to the InterPro database. Mean values with standard deviation are presented. Values denoted with the same letter as statistically significant at $p<0.05$

No	Domains	Entire proteins			Epitopes		
		ΣA	DH_t	ΣA_E	ΣA	DH_t	ΣA_E
1.	Set 1	1.1013 ±0.0290 (n = 4)[a, b, c, d]	77.7233 ±1.8681 (n = 4)[e]	0.1544 ±0.0278 (n = 4)[f, g, h, I, j]	0.9750 ±0.3092 (n = 6)[k, l, m, n]	79.6272 ±21.6151 (n = 6)	0.0672 ±0.0592 (n = 6)[j, o, p, r, s]
2	Set 2	1.1960 ±0.0448 (n = 2)	81.8807 ±1.1307 (n = 2)[e]	0.1714 ±0.0296 (n = 2)	1.0819 ±0.4726 (n = 15)[o, p, r]	79.9098 ±8.9223 (n = 15)	0.1836 ±0.1343 (n = 15)[o]
3	Set 3a	1.3605 ±0.0134 (n = 4)[a]	80.2146 ±1.1748 (n = 4)	0.2271 ±0.0169 (n = 4)[f]	1.4369 ±0.3325 (n = 19)[k, o]	79.5697 ±8.8398 (n = 19)	0.2712 ±0.1745 (n = 19)[p]
4	Set 3b	1.3228 ±0.0403 (n = 5)[b]	78.2530 ±2.6572 (n = 5)	0.2192 ±0.0299 (n = 5)[g]	1.3815 ±0.2702 (n = 24)[l, p]	80.9392 ±9.9099 (n = 24)	0.2581 ±0.1852 n = 24)[r]
5	Set 3	1.3396 ±0.0368 (n = 9)[c]	79.1248 ±2.2620 (n = 9)	0.2227 ±0.0239 (n = 9)[h]	1.4060 ±0.2969 (n = 43)[m, r]	80.3341 ±9.3671 (n = 43)	0.2639 ±0.1786 (n = 43)
6	Set 2 + 3	1.3135 ±0.0682 (n = 11)[d]	79.6259 ±2.3375 (n = 11)	0.2134 ±0.0313 (n = 11)[i]	1.3222 ±0.4049 (n = 58)[n]	80.2243 ±9.1782 (n = 58)	0.2432 ±0.1708 (n = 58)[s]
	All proteins	1.2779 ±0.1218 (n = 17)	78.4252 ±3.8609 (n = 17)	0.2002 ±0.0397 (n = 17)	1.2896 ±0.3807 (n = 64)	80.1683 ±10.6456 (n = 64)	0.2267 ±0.1713 (n = 64)

Table S6. Profile of potential biological activity of fragments of protein Ana o 1.0101.

ID	Name of peptide	Activity	Number	Sequence	Location
3458	Prolyl endopeptidase inhibitor	antiamnestic	1	GPGG	[281-284]
3460	Prolyl endopeptidase inhibitor	antiamnestic	2	PG	[273-274],[282-283]
3461	Prolyl endopeptidase inhibitor	antiamnestic	4	GP	[2-3],[281-282],[339-340],[525-526]
3257	beta-lactokinin	ACE inhibitor	1	RL	[191-192]
3258	beta-lactokinin	ACE inhibitor	3	IR	[330-331],[443-444],[480-481]
3377	ACE inhibitor (from bovine as1-CN)	ACE inhibitor	1	FGK	[514-516]
3380	ACE inhibitor	ACE inhibitor	2	RY	[122-123],[481-482]
3381	ACE inhibitor	ACE inhibitor	1	LY	[261-262]
3384	ACE inhibitor	ACE inhibitor	4	VF	[154-155],[278-279],[448-449],[513-514]
3421	ACE inhibitor	ACE inhibitor	1	LVL	[17-19]
3489	ACE inhibitor from sake lees	ACE inhibitor	1	RF	[116-117]
3543	ACE inhibitor	ACE inhibitor	1	LRP	[267-269]
3547	ACE inhibitor	ACE inhibitor	1	IRA	[330-332]
3551	ACE inhibitor (from bovine beta-Lg)	ACE inhibitor	4	LF	[11-12],[313-314],[355-356],[369-370]
3553	ACE inhibitor	ACE inhibitor	1	YG	[366-367]
3556	ACE inhibitor	ACE inhibitor	3	FY	[250-251],[290-291],[401-402]
3666	ACE inhibitor	ACE inhibitor	1	YP	[377-378]
3713	ACE inhibitor from alpha-zein	ACE inhibitor	1	LLP	[173-175]
7511	ACE inhibitor from Alaskan 14embran skin	ACE inhibitor	1	LPG	[272-274]
7512	ACE inhibitor from Alaskan 14embran skin	ACE inhibitor	4	GP	[2-3],[281-282],[339-340],[525-526]
7513	ACE inhibitor from Alaskan 14embran skin	ACE inhibitor	1	PL	[379-380]
7544	ACE inhibitor	ACE inhibitor	1	IW	[342-343]
7558	ACE inhibitor from buckwheat	ACE inhibitor	2	VK	[54-55],[164-165]
7559	ACE inhibitor from buckwheat	ACE inhibitor	1	PSY	[434-436]
7562	ACE inhibitor from soy hydrolysate	ACE inhibitor	3	IA	[252-253],[409-410],[421-422]
7582	ACE inhibitor	ACE inhibitor	1	RP	[268-269]
7583	ACE inhibitor	ACE inhibitor	3	AF	[201-202],[293-294],[502-503]
7585	ACE inhibitor	ACE inhibitor	4	LA	[25-26],[192-193],[484-485],[501-502]
7586	ACE inhibitor	ACE inhibitor	2	KR	[133-134],[230-231]
7587	ACE inhibitor	ACE inhibitor	3	VP	[204-205],[399-400],[451-452]
7588	ACE inhibitor	ACE inhibitor	5	RA	[292-293],[331-332],[405-406],[440-441],[535-536]
7589	ACE inhibitor	ACE inhibitor	1	YA	[389-390]
7590	ACE inhibitor	ACE inhibitor	1	AA	[301-302]

7591	ACE inhibitor	ACE inhibitor	1	GF	[22-23]
7592	ACE inhibitor	ACE inhibitor	1	FR	[117-118]
7593	ACE inhibitor	ACE inhibitor	1	IF	[213-214]
7597	ACE inhibitor	ACE inhibitor	1	GM	[396-397]
7600	ACE inhibitor	ACE inhibitor	2	AG	[453-454],[485-486]
7601	ACE inhibitor	ACE inhibitor	2	GH	[274-275],[454-455]
7602	ACE inhibitor	ACE inhibitor	2	HL	[95-96],[425-426]
7603	ACE inhibitor	ACE inhibitor	3	GR	[66-67],[219-220],[534-535]
7604	ACE inhibitor	ACE inhibitor	2	KG	[65-66],[394-395]
7605	ACE inhibitor	ACE inhibitor	1	FG	[514-515]
7606	ACE inhibitor	ACE inhibitor	1	DA	[209-210]
7607	ACE inhibitor	ACE inhibitor	1	GS	[351-352]
7608	ACE inhibitor	ACE inhibitor	1	GV	[82-83]
7609	ACE inhibitor	ACE inhibitor	1	MG	[1-2]
7610	ACE inhibitor	ACE inhibitor	3	GQ	[109-110],[128-129],[367-368]
7611	ACE inhibitor	ACE inhibitor	3	GK	[169-170],[486-487],[515-516]
7612	ACE inhibitor	ACE inhibitor	4	GT	[79-80],[221-222],[247-248],[320-321]
7613	ACE inhibitor	ACE inhibitor	2	WG	[78-79],[218-219]
7614	ACE inhibitor	ACE inhibitor	1	HG	[280-281]
7615	ACE inhibitor	ACE inhibitor	4	GE	[284-285],[337-338],[415-416],[507-508]
7616	ACE inhibitor	ACE inhibitor	4	GG	[81-82],[108-109],[283-284],[395-396]
7617	ACE inhibitor	ACE inhibitor	4	QG	[168-169],[238-239],[319-320],[524-525]
7618	ACE inhibitor	ACE inhibitor	3	SG	[246-247],[414-415],[462-463]
7619	ACE inhibitor	ACE inhibitor	1	LG	[21-22]
7620	ACE inhibitor	ACE inhibitor	1	GD	[239-240]
7621	ACE inhibitor	ACE inhibitor	2	TG	[80-81],[350-351]
7622	ACE inhibitor	ACE inhibitor	6	EG	[107-108],[338-339],[416-417],[477-478],[506-507],[533-534]
7623	ACE inhibitor	ACE inhibitor	4	EA	[144-145],[300-301],[371-372],[497-498]
7625	ACE inhibitor	ACE inhibitor	2	PG	[273-274],[282-283]
7628	ACE inhibitor from k-CN (fr. 67-68)	ACE inhibitor	1	VR	[236-237]
7636	ACE inhibitor from k-CN (fr. 78-80)	ACE inhibitor	1	SHP	[432-434]
7637	ACE inhibitor from bonito bowels	ACE inhibitor	1	GHF	[274-276]
7653	ACE inhibitor from wakame	ACE inhibitor	1	KYY	[59-61]
7680	ACE inhibitor from pea vicilin	ACE inhibitor	3	QK	[49-50],[111-112],[179-180]
7682	ACE inhibitor from 15embra	ACE inhibitor	1	NY	[131-132]
7684	ACE inhibitor from 15embra	ACE inhibitor	2	SY	[388-389],[435-436]

7685	ACE inhibitor	ACE inhibitor	3	SF	[8-9],[289-290],[352-353]
7691	ACE inhibitor from wakame	ACE inhibitor	3	KY	[59-60],[189-190],[365-366]
7692	ACE inhibitor	ACE inhibitor	2	KF	[6-7],[176-177]
7693	ACE inhibitor from wakame	ACE inhibitor	6	KL	[182-183],[260-261],[312-313],[354-355],[382-383],[438-439]
7697	ACE inhibitor from wakame	ACE inhibitor	4	YK	[61-62],[123-124],[132-133],[436-437]
7698	ACE inhibitor from wakame	ACE inhibitor	2	NK	[229-230],[364-365]
7741	ACE inhibitor	ACE inhibitor	1	RR	[335-336]
7742	ACE inhibitor	ACE inhibitor	1	AR	[441-442]
7743	ACE inhibitor	ACE inhibitor	1	KA	[324-325]
7751	ACE inhibitor from shark meat hydrolysate	ACE inhibitor	1	CF	[471-472]
7820	ACE inhibitor from wheat gliadin	ACE inhibitor	1	GPP	[2-4]
7823	ACE inhibitor from micro algae	ACE inhibitor	1	FAL	[23-25]
7826	ACE inhibitor	ACE inhibitor	3	EI	[297-298],[420-421],[468-469]
7828	ACE inhibitor	ACE inhibitor	3	EV	[277-278],[473-474],[509-510]
7829	ACE inhibitor	ACE inhibitor	1	VE	[419-420]
7830	ACE inhibitor	ACE inhibitor	2	TE	[166-167],[346-347]
7833	ACE inhibitor	ACE inhibitor	1	PT	[4-5]
7834	ACE inhibitor	ACE inhibitor	1	TQ	[178-179]
7836	ACE inhibitor	ACE inhibitor	2	PP	[3-4],[378-379]
7837	ACE inhibitor	ACE inhibitor	1	PQ	[199-200]
7838	ACE inhibitor	ACE inhibitor	2	EW	[77-78],[527-528]
7839	ACE inhibitor	ACE inhibitor	2	ME	[494-495],[505-506]
7840	ACE inhibitor	ACE inhibitor	10	EK	[58-59],[63-64],[93-94],[188-189],[259-260],[311-312],[315-316],[381-382],[495-496],[531-532]
7841	ACE inhibitor	ACE inhibitor	9	KE	[50-51],[55-56],[62-63],[125-126],[327-328],[496-497],[499-500],[530-531],[532-533]
7842	ACE inhibitor	ACE inhibitor	2	HP	[433-434],[455-456]
7843	ACE inhibitor	ACE inhibitor	1	PH	[424-425]
8193	ACE inhibitor	ACE inhibitor	1	AI	[410-411]
8951	ACE inhibitor	ACE inhibitor	1	AV	[193-194]
9037	ACE inhibitor	ACE inhibitor	1	GKV	[169-171]
9048	ACE inhibitor	ACE inhibitor	1	LVQ	[263-265]
9053	ACE inhibitor	ACE inhibitor	1	FYN	[401-403]
9060	ACE inhibitor	ACE inhibitor	1	AVL	[193-195]
9064	ACE inhibitor	ACE inhibitor	3	LEK	[187-189],[310-312],[380-382]
9072	ACE inhibitor	ACE inhibitor	1	DY	[376-377]

9073	ACE inhibitor	ACE inhibitor	1	TP	[248-249]
9074	ACE inhibitor	ACE inhibitor	1	DF	[159-160]
9075	ACE inhibitor	ACE inhibitor	1	DM	[384-385]
9076	ACE inhibitor	ACE inhibitor	1	FQ	[523-524]
9077	ACE inhibitor	ACE inhibitor	1	YV	[153-154]
9079	ACE inhibitor	ACE inhibitor	2	IL	[226-227],[298-299]
9142	ACE inhibitor	ACE inhibitor	1	MGP	[1-3]
9146	ACE inhibitor	ACE inhibitor	1	QGP	[524-526]
9173	ACE inhibitor	ACE inhibitor	3	RG	[127-128],[220-221],[336-337]
9183	ACE inhibitor	ACE inhibitor	1	GTG	[79-81]
9184	ACE inhibitor	ACE inhibitor	2	ST	[87-88],[349-350]
9185	ACE inhibitor	ACE inhibitor	1	YN	[402-403]
9213	ACE inhibitor	ACE inhibitor	2	LR	[267-268],[439-440]
9731	ACE inhibitor	ACE inhibitor	1	VVL	[171-173]
3283	Antithrombotic peptide	antithrombotic	4	GP	[2-3],[281-282],[339-340],[525-526]
3285	Antithrombotic peptide	antithrombotic	2	PG	[273-274],[282-283]
3354	Antithrombotic peptide	antithrombotic	1	DEE	[518-520]
3462		antithrombotic	1	GPGG	[281-284]
2882	Immunostimulating peptide	immunomodulating	1	YG	[366-367]
3351	Stimulating vasoactive substance release	stimulating	5	EEE	[72-74],[73-75],[74-76],[75-77],[146-148]
3355	Stimulating vasoactive substance release	stimulating	1	SSS	[427-429]
8320	Glucose uptake stimulating peptide	stimulating	4	VL	[16-17],[18-19],[172-173],[194-195]
8321	Glucose uptake stimulating peptide	stimulating	4	LV	[13-14],[17-18],[195-196],[263-264]
8322	Glucose uptake stimulating peptide	stimulating	4	IV	[241-242],[411-412],[450-451],[469-470]
8323	Glucose uptake stimulating peptide	stimulating	2	IL	[226-227],[298-299]
8325	Glucose uptake stimulating peptide	stimulating	1	II	[490-491]
8326	Glucose uptake stimulating peptide	stimulating	2	LL	[173-174],[183-184]
8329	Stimulating vasoactive substance release	stimulating	10	EE	[72-73],[73-74],[74-75],[75-76],[76-77],[146-147],[147-148],[347-348],[508-509],[519-520]
3066	Immunostimulating peptide	immunostimulating	2	EAE	[144-146],[371-373]
2890	neuropeptide	neuropeptide	3	GQ	[109-110],[128-129],[367-368]
8310	Anxiolytic peptide	neuropeptide	1	YL	[262-263]
9534	Kyotorphin	neuropeptide	2	YR	[190-191],[291-292]
2749	peptide regulating ion flow	regulating	1	DY	[376-377]
2753	peptide regulating the stomach mucosal 17embranę activity	regulating	4	GP	[2-3],[281-282],[339-340],[525-526]
2754	peptide regulating the stomach mucosal 17embranę activity	regulating	2	PG	[273-274],[282-283]

2755	peptide regulating the stomach mucosal 18embranę activity	regulating	1	GPGG	[281-284]
3305		antioxidative	1	LH	[184-185]
3317		antioxidative	2	HL	[95-96],[425-426]
7863	peptide from prawn muscle (Penaeus japonicus)	antioxidative	1	FKK	[356-358]
7872	peptide from soybean protein isolates: beta-conglycinin and glycinin	antioxidative	1	LY	[261-262]
7888	antioxidative peptide	antioxidative	2	EL	[31-32],[500-501]
7931	synthetic peptide	antioxidative	1	KYY	[59-61]
7937	synthetic peptide	antioxidative	1	YYK	[60-62]
7988	synthetic peptide	antioxidative	1	LHA	[184-186]
8029	synthetic peptide	antioxidative	1	PHL	[424-426]
8114	peptide derived from sardinelle by-products proteins (Sardinella aurita)	antioxidative	1	GGE	[283-285]
8130	peptide derived from dried bonito	antioxidative	1	EAK	[497-499]
8134	peptide derived from dried bonito	antioxidative	3	KD	[307-308],[358-359],[445-446]
8139	synthetic peptide	antioxidative	1	PEL	[30-32]
8215	Antioxidative peptide	antioxidative	3	IR	[330-331],[443-444],[480-481]
8217	Antioxidative peptide	antioxidative	2	LK	[32-33],[303-304]
8987	Antioxidative peptide	antioxidative	1	GPP	[2-4]
9082	Antioxidative peptide	antioxidative	2	WG	[78-79],[218-219]
9363	Antioxidative peptide	antioxidative	1	NEN	[464-466]
9368	Antioxidative peptide	antioxidative	1	EQC	[51-53]
3751		bacterial permease ligand	5	KK	[64-65],[124-125],[357-358],[437-438],[487-488]
4005		activating ubiquitin-mediated proteolysis	5	RA	[292-293],[331-332],[405-406],[440-441],[535-536]
4006	Ubiqitin-mediated proteolysis activating peptide	activating ubiquitin-mediated proteolysis	4	LA	[25-26],[192-193],[484-485],[501-502]
9580	Hypolipidemic peptide	hypolipidemic	1	EF	[520-521]
9548	Alpha-glucosidase inhibitor	alpha-glucosidase inhibitor	1	YP	[377-378]
9650	Alpha-glucosidase inhibitor	alpha-glucosidase inhibitor	4	EA	[144-145],[300-301],[371-372],[497-498]
9651	Alpha-glucosidase inhibitor	alpha-glucosidase inhibitor	2	PP	[3-4],[378-379]
9693	Alpha-glucosidase inhibitor	alpha-glucosidase inhibitor	1	VE	[419-420]
9694	Alpha-glucosidase inhibitor	alpha-glucosidase inhibitor	3	PE	[30-31],[287-288],[526-527]
9695	Alpha-glucosidase inhibitor	alpha-glucosidase inhibitor	2	AD	[210-211],[536-537]
9383	HMG-CoA reductase inhibitor	HMG-CoA reductase inhibitor	1	GGV	[81-83]
3169	dipeptidyl peptidase IV inhibitor (DPP IV inhibitor)	dipeptidyl peptidase IV inhibitor	4	GP	[2-3],[281-282],[339-340],[525-526]
3170	dipeptidyl peptidase IV inhibitor (DPP IV inhibitor)	dipeptidyl peptidase IV inhibitor	2	PP	[3-4],[378-379]

3172	dipeptidyl peptidase IV inhibitor (DPP IV inhibitor)	dipeptidyl peptidase IV inhibitor	2	VA	[196-197],[460-461]
3174	dipeptidyl peptidase IV inhibitor (DPP IV inhibitor)	dipeptidyl peptidase IV inhibitor	1	KA	[324-325]
3175	dipeptidyl peptidase IV inhibitor (DPP IV inhibitor)	dipeptidyl peptidase IV inhibitor	4	LA	[25-26],[192-193],[484-485],[501-502]
3176	dipeptidyl peptidase IV inhibitor (DPP IV inhibitor)	dipeptidyl peptidase IV inhibitor	2	FA	[23-24],[457-458]
3179	dipeptidyl peptidase IV inhibitor (DPP IV inhibitor)	dipeptidyl peptidase IV inhibitor	2	PA	[91-92],[452-453]
3180	dipeptidyl peptidase IV inhibitor (DPP IV inhibitor)	dipeptidyl peptidase IV inhibitor	2	LP	[174-175],[272-273]
3181	dipeptidyl peptidase IV inhibitor (DPP IV inhibitor)	dipeptidyl peptidase IV inhibitor	3	VP	[204-205],[399-400],[451-452]
3182	dipeptidyl peptidase IV inhibitor (DPP IV inhibitor)	dipeptidyl peptidase IV inhibitor	2	LL	[173-174],[183-184]
3183	dipeptidyl peptidase IV inhibitor (DPP IV inhibitor)	dipeptidyl peptidase IV inhibitor	4	VV	[171-172],[203-204],[386-387],[412-413]
3184	dipeptidyl peptidase IV inhibitor (DPP IV inhibitor)	dipeptidyl peptidase IV inhibitor	1	HA	[185-186]
8503	Dipeptidyl peptidase IV inhibitor (DPP IV inhibitor)	dipeptidyl peptidase IV inhibitor	1	TP	[248-249]
8504	Dipeptidyl peptidase IV inhibitor (DPP IV inhibitor)	dipeptidyl peptidase IV inhibitor	1	WP	[343-344]
8518	dipeptidyl peptidase IV inhibitor (DPP IV inhibitor)	dipeptidyl peptidase IV inhibitor	1	RP	[268-269]
8520	dipeptidyl peptidase IV inhibitor (DPP IV inhibitor)	dipeptidyl peptidase IV inhibitor	2	HP	[433-434],[455-456]
8521	dipeptidyl peptidase IV inhibitor (DPP IV inhibitor)	dipeptidyl peptidase IV inhibitor	1	YP	[377-378]
8525	dipeptidyl peptidase IV inhibitor (DPP IV inhibitor)	dipeptidyl peptidase IV inhibitor	3	IA	[252-253],[409-410],[421-422]
8526	dipeptidyl peptidase IV inhibitor (DPP IV inhibitor)	dipeptidyl peptidase IV inhibitor	5	RA	[292-293],[331-332],[405-406],[440-441],[535-536]
8529	dipeptidyl peptidase IV inhibitor (DPP IV inhibitor)	dipeptidyl peptidase IV inhibitor	2	EP	[85-86],[90-91]
8530	dipeptidyl peptidase IV inhibitor (DPP IV inhibitor)	dipeptidyl peptidase IV inhibitor	3	AL.	[151-152],[198-199],[286-287]
8555	dipeptidyl peptidase IV inhibitor (DPP IV inhibitor)	dipeptidyl peptidase IV inhibitor	2	FL	[12-13],[266-267]
8557	dipeptidyl peptidase IV inhibitor (DPP IV inhibitor)	dipeptidyl peptidase IV inhibitor	2	HL	[95-96],[425-426]
8558	dipeptidyl peptidase IV inhibitor (DPP IV inhibitor)	dipeptidyl peptidase IV inhibitor	10	EK	[58-59],[63-64],[93-94],[188-189],[259-260],[311-312],[315-316],[381-382],[495-496],[531-532]
8559	dipeptidyl peptidase IV inhibitor (DPP IV inhibitor)	dipeptidyl peptidase IV inhibitor	3	AL.	[24-25],[186-187],[302-303]
8560	dipeptidyl peptidase IV inhibitor (DPP IV inhibitor)	dipeptidyl peptidase IV inhibitor	1	SL	[10-11]
8594	dipeptidyl peptidase IV inhibitor (DPP IV inhibitor)	dipeptidyl peptidase IV inhibitor	1	VR	[236-237]
8598	dipeptidyl peptidase IV inhibitor (DPP IV inhibitor)	dipeptidyl peptidase IV inhibitor	1	WRK	[528-530]
8637	dipeptidyl peptidase IV inhibitor (DPP IV inhibitor)	dipeptidyl peptidase IV inhibitor	1	AA	[301-302]
8638	dipeptidyl peptidase IV inhibitor (DPP IV inhibitor)	dipeptidyl peptidase IV inhibitor	1	PL	[379-380]
8652	dipeptidyl peptidase IV inhibitor (DPP IV inhibitor)	dipeptidyl peptidase IV inhibitor	1	PPL	[378-380]
8675	dipeptidyl peptidase IV inhibitor (DPP IV inhibitor)	dipeptidyl peptidase IV inhibitor	1	WR	[528-529]
8691	dipeptidyl peptidase IV inhibitor (DPP IV inhibitor)	dipeptidyl peptidase IV inhibitor	1	WE	[296-297]
8696	dipeptidyl peptidase IV inhibitor (DPP IV inhibitor)	dipeptidyl peptidase IV inhibitor	1	YT	[482-483]
8697	dipeptidyl peptidase IV inhibitor (DPP IV inhibitor)	dipeptidyl peptidase IV inhibitor	2	WG	[78-79],[218-219]
8757	dipeptidyl peptidase IV inhibitor (DPP IV inhibitor)	dipeptidyl peptidase IV inhibitor	2	AD	[210-211],[536-537]
8758	dipeptidyl peptidase IV inhibitor (DPP IV inhibitor)	dipeptidyl peptidase IV inhibitor	4	AE	[92-93],[145-146],[372-373],[476-477]

8759	dipeptidyl peptidase IV inhibitor (DPP IV inhibitor)	dipeptidyl peptidase IV inhibitor	3	AF	[201-202],[293-294],[502-503]
8760	dipeptidyl peptidase IV inhibitor (DPP IV inhibitor)	dipeptidyl peptidase IV inhibitor	2	AG	[453-454],[485-486]
8762	dipeptidyl peptidase IV inhibitor (DPP IV inhibitor)	dipeptidyl peptidase IV inhibitor	2	AS	[325-326],[461-462]
8763	dipeptidyl peptidase IV inhibitor (DPP IV inhibitor)	dipeptidyl peptidase IV inhibitor	2	AT	[406-407],[458-459]
8764	dipeptidyl peptidase IV inhibitor (DPP IV inhibitor)	dipeptidyl peptidase IV inhibitor	1	AV	[193-194]
8767	dipeptidyl peptidase IV inhibitor (DPP IV inhibitor)	dipeptidyl peptidase IV inhibitor	2	DP	[29-30],[359-360]
8768	dipeptidyl peptidase IV inhibitor (DPP IV inhibitor)	dipeptidyl peptidase IV inhibitor	1	DQ	[318-319]
8770	dipeptidyl peptidase IV inhibitor (DPP IV inhibitor)	dipeptidyl peptidase IV inhibitor	6	EG	[107-108],[338-339],[416-417],[477-478],[506-507],[533-534]
8771	dipeptidyl peptidase IV inhibitor (DPP IV inhibitor)	dipeptidyl peptidase IV inhibitor	1	EH	[70-71]
8772	dipeptidyl peptidase IV inhibitor (DPP IV inhibitor)	dipeptidyl peptidase IV inhibitor	3	EI	[297-298],[420-421],[468-469]
8773	dipeptidyl peptidase IV inhibitor (DPP IV inhibitor)	dipeptidyl peptidase IV inhibitor	3	ES	[232-233],[288-289],[348-349]
8775	dipeptidyl peptidase IV inhibitor (DPP IV inhibitor)	dipeptidyl peptidase IV inhibitor	3	EV	[277-278],[473-474],[509-510]
8776	dipeptidyl peptidase IV inhibitor (DPP IV inhibitor)	dipeptidyl peptidase IV inhibitor	2	EW	[77-78],[527-528]
8779	dipeptidyl peptidase IV inhibitor (DPP IV inhibitor)	dipeptidyl peptidase IV inhibitor	1	FQ	[523-524]
8780	dipeptidyl peptidase IV inhibitor (DPP IV inhibitor)	dipeptidyl peptidase IV inhibitor	1	FR	[117-118]
8781	dipeptidyl peptidase IV inhibitor (DPP IV inhibitor)	dipeptidyl peptidase IV inhibitor	4	GE	[284-285],[337-338],[415-416],[507-508]
8782	dipeptidyl peptidase IV inhibitor (DPP IV inhibitor)	dipeptidyl peptidase IV inhibitor	1	GF	[22-23]
8783	dipeptidyl peptidase IV inhibitor (DPP IV inhibitor)	dipeptidyl peptidase IV inhibitor	4	GG	[81-82],[108-109],[283-284],[395-396]
8784	dipeptidyl peptidase IV inhibitor (DPP IV inhibitor)	dipeptidyl peptidase IV inhibitor	2	GH	[274-275],[454-455]
8786	dipeptidyl peptidase IV inhibitor (DPP IV inhibitor)	dipeptidyl peptidase IV inhibitor	1	GV	[82-83]
8790	dipeptidyl peptidase IV inhibitor (DPP IV inhibitor)	dipeptidyl peptidase IV inhibitor	2	HE	[71-72],[89-90]
8791	dipeptidyl peptidase IV inhibitor (DPP IV inhibitor)	dipeptidyl peptidase IV inhibitor	1	HF	[275-276]
8801	dipeptidyl peptidase IV inhibitor (DPP IV inhibitor)	dipeptidyl peptidase IV inhibitor	1	II	[490-491]
8802	dipeptidyl peptidase IV inhibitor (DPP IV inhibitor)	dipeptidyl peptidase IV inhibitor	2	IL	[226-227],[298-299]
8803	dipeptidyl peptidase IV inhibitor (DPP IV inhibitor)	dipeptidyl peptidase IV inhibitor	1	IM	[322-323]
8804	dipeptidyl peptidase IV inhibitor (DPP IV inhibitor)	dipeptidyl peptidase IV inhibitor	1	IN	[234-235]
8806	dipeptidyl peptidase IV inhibitor (DPP IV inhibitor)	dipeptidyl peptidase IV inhibitor	3	IR	[330-331],[443-444],[480-481]
8807	dipeptidyl peptidase IV inhibitor (DPP IV inhibitor)	dipeptidyl peptidase IV inhibitor	1	IW	[342-343]
8808	dipeptidyl peptidase IV inhibitor (DPP IV inhibitor)	dipeptidyl peptidase IV inhibitor	9	KE	[50-51],[55-56],[62-63],[125-126],[327-328],[496-497],[499-500],[530-531],[532-533]
8809	dipeptidyl peptidase IV inhibitor (DPP IV inhibitor)	dipeptidyl peptidase IV inhibitor	2	KF	[6-7],[176-177]
8810	dipeptidyl peptidase IV inhibitor (DPP IV inhibitor)	dipeptidyl peptidase IV inhibitor	2	KG	[65-66],[394-395]
8811	dipeptidyl peptidase IV inhibitor (DPP IV inhibitor)	dipeptidyl peptidase IV inhibitor	2	KH	[36-37],[94-95]
8812	dipeptidyl peptidase IV inhibitor (DPP IV inhibitor)	dipeptidyl peptidase IV inhibitor	4	KI	[27-28],[225-226],[341-342],[408-409]
8813	dipeptidyl peptidase IV inhibitor (DPP IV inhibitor)	dipeptidyl peptidase IV inhibitor	5	KK	[64-65],[124-125],[357-358],[437-438],[487-488]
8814	dipeptidyl peptidase IV inhibitor (DPP IV inhibitor)	dipeptidyl peptidase IV inhibitor	2	KR	[133-134],[230-231]

8815	dipeptidyl peptidase IV inhibitor (DPP IV inhibitor)	dipeptidyl peptidase IV inhibitor	2	KS	[180-181],[430-431]
8816	dipeptidyl peptidase IV inhibitor (DPP IV inhibitor)	dipeptidyl peptidase IV inhibitor	2	KT	[165-166],[304-305]
8817	dipeptidyl peptidase IV inhibitor (DPP IV inhibitor)	dipeptidyl peptidase IV inhibitor	5	KV	[40-41],[163-164],[170-171],[492-493],[512-513]
8819	dipeptidyl peptidase IV inhibitor (DPP IV inhibitor)	dipeptidyl peptidase IV inhibitor	3	KY	[59-60],[189-190],[365-366]
8820	dipeptidyl peptidase IV inhibitor (DPP IV inhibitor)	dipeptidyl peptidase IV inhibitor	1	LH	[184-185]
8825	dipeptidyl peptidase IV inhibitor (DPP IV inhibitor)	dipeptidyl peptidase IV inhibitor	4	LV	[13-14],[17-18],[195-196],[263-264]
8826	dipeptidyl peptidase IV inhibitor (DPP IV inhibitor)	dipeptidyl peptidase IV inhibitor	2	ME	[494-495],[505-506]
8828	dipeptidyl peptidase IV inhibitor (DPP IV inhibitor)	dipeptidyl peptidase IV inhibitor	1	MG	[1-2]
8831	dipeptidyl peptidase IV inhibitor (DPP IV inhibitor)	dipeptidyl peptidase IV inhibitor	1	MK	[323-324]
8836	dipeptidyl peptidase IV inhibitor (DPP IV inhibitor)	dipeptidyl peptidase IV inhibitor	1	MR	[100-101]
8837	dipeptidyl peptidase IV inhibitor (DPP IV inhibitor)	dipeptidyl peptidase IV inhibitor	1	MV	[385-386]
8839	dipeptidyl peptidase IV inhibitor (DPP IV inhibitor)	dipeptidyl peptidase IV inhibitor	1	NA	[475-476]
8840	dipeptidyl peptidase IV inhibitor (DPP IV inhibitor)	dipeptidyl peptidase IV inhibitor	1	ND	[255-256]
8841	dipeptidyl peptidase IV inhibitor (DPP IV inhibitor)	dipeptidyl peptidase IV inhibitor	2	NE	[258-259],[464-465]
8845	dipeptidyl peptidase IV inhibitor (DPP IV inhibitor)	dipeptidyl peptidase IV inhibitor	2	NL	[271-272],[466-467]
8847	dipeptidyl peptidase IV inhibitor (DPP IV inhibitor)	dipeptidyl peptidase IV inhibitor	1	NN	[254-255]
8851	dipeptidyl peptidase IV inhibitor (DPP IV inhibitor)	dipeptidyl peptidase IV inhibitor	1	NV	[235-236]
8853	dipeptidyl peptidase IV inhibitor (DPP IV inhibitor)	dipeptidyl peptidase IV inhibitor	1	NY	[131-132]
8854	dipeptidyl peptidase IV inhibitor (DPP IV inhibitor)	dipeptidyl peptidase IV inhibitor	4	PF	[249-250],[344-345],[400-401],[456-457]
8855	dipeptidyl peptidase IV inhibitor (DPP IV inhibitor)	dipeptidyl peptidase IV inhibitor	2	PG	[273-274],[282-283]
8856	dipeptidyl peptidase IV inhibitor (DPP IV inhibitor)	dipeptidyl peptidase IV inhibitor	1	PH	[424-425]
8858	dipeptidyl peptidase IV inhibitor (DPP IV inhibitor)	dipeptidyl peptidase IV inhibitor	2	PK	[175-176],[340-341]
8861	dipeptidyl peptidase IV inhibitor (DPP IV inhibitor)	dipeptidyl peptidase IV inhibitor	1	PQ	[199-200]
8862	dipeptidyl peptidase IV inhibitor (DPP IV inhibitor)	dipeptidyl peptidase IV inhibitor	4	PS	[86-87],[205-206],[360-361],[434-435]
8863	dipeptidyl peptidase IV inhibitor (DPP IV inhibitor)	dipeptidyl peptidase IV inhibitor	1	PT	[4-5]
8864	dipeptidyl peptidase IV inhibitor (DPP IV inhibitor)	dipeptidyl peptidase IV inhibitor	1	PV	[269-270]
8866	dipeptidyl peptidase IV inhibitor (DPP IV inhibitor)	dipeptidyl peptidase IV inhibitor	1	PY	[152-153]
8867	dipeptidyl peptidase IV inhibitor (DPP IV inhibitor)	dipeptidyl peptidase IV inhibitor	1	QA	[200-201]
8868	dipeptidyl peptidase IV inhibitor (DPP IV inhibitor)	dipeptidyl peptidase IV inhibitor	2	QD	[317-318],[517-518]
8869	dipeptidyl peptidase IV inhibitor (DPP IV inhibitor)	dipeptidyl peptidase IV inhibitor	2	QE	[106-107],[120-121]
8870	dipeptidyl peptidase IV inhibitor (DPP IV inhibitor)	dipeptidyl peptidase IV inhibitor	1	QF	[265-266]
8871	dipeptidyl peptidase IV inhibitor (DPP IV inhibitor)	dipeptidyl peptidase IV inhibitor	4	QG	[168-169],[238-239],[319-320],[524-525]
8872	dipeptidyl peptidase IV inhibitor (DPP IV inhibitor)	dipeptidyl peptidase IV inhibitor	1	QH	[129-130]
8873	dipeptidyl peptidase IV inhibitor (DPP IV inhibitor)	dipeptidyl peptidase IV inhibitor	1	QI	[329-330]
8874	dipeptidyl peptidase IV inhibitor (DPP IV inhibitor)	dipeptidyl peptidase IV inhibitor	2	QL	[113-114],[368-369]
8876	dipeptidyl peptidase IV inhibitor (DPP IV inhibitor)	dipeptidyl peptidase IV inhibitor	2	QQ	[48-49],[110-111]

8877	dipeptidyl peptidase IV inhibitor (DPP IV inhibitor)	dipeptidyl peptidase IV inhibitor	1	QS	[362-363]
8881	dipeptidyl peptidase IV inhibitor (DPP IV inhibitor)	dipeptidyl peptidase IV inhibitor	1	QY	[44-45]
8882	dipeptidyl peptidase IV inhibitor (DPP IV inhibitor)	dipeptidyl peptidase IV inhibitor	3	RG	[127-128],[220-221],[336-337]
8884	dipeptidyl peptidase IV inhibitor (DPP IV inhibitor)	dipeptidyl peptidase IV inhibitor	2	RI	[374-375],[442-443]
8885	dipeptidyl peptidase IV inhibitor (DPP IV inhibitor)	dipeptidyl peptidase IV inhibitor	2	RK	[444-445],[529-530]
8886	dipeptidyl peptidase IV inhibitor (DPP IV inhibitor)	dipeptidyl peptidase IV inhibitor	1	RL	[191-192]
8889	dipeptidyl peptidase IV inhibitor (DPP IV inhibitor)	dipeptidyl peptidase IV inhibitor	1	RR	[335-336]
8891	dipeptidyl peptidase IV inhibitor (DPP IV inhibitor)	dipeptidyl peptidase IV inhibitor	3	SF	[8-9],[289-290],[352-353]
8892	dipeptidyl peptidase IV inhibitor (DPP IV inhibitor)	dipeptidyl peptidase IV inhibitor	2	SH	[206-207],[432-433]
8893	dipeptidyl peptidase IV inhibitor (DPP IV inhibitor)	dipeptidyl peptidase IV inhibitor	3	SI	[212-213],[233-234],[243-244]
8894	dipeptidyl peptidase IV inhibitor (DPP IV inhibitor)	dipeptidyl peptidase IV inhibitor	4	SK	[181-182],[306-307],[326-327],[429-430]
8895	dipeptidyl peptidase IV inhibitor (DPP IV inhibitor)	dipeptidyl peptidase IV inhibitor	2	SV	[15-16],[398-399]
8896	dipeptidyl peptidase IV inhibitor (DPP IV inhibitor)	dipeptidyl peptidase IV inhibitor	2	SW	[217-218],[295-296]
8897	dipeptidyl peptidase IV inhibitor (DPP IV inhibitor)	dipeptidyl peptidase IV inhibitor	2	SY	[388-389],[435-436]
8899	dipeptidyl peptidase IV inhibitor (DPP IV inhibitor)	dipeptidyl peptidase IV inhibitor	2	TE	[166-167],[346-347]
8901	dipeptidyl peptidase IV inhibitor (DPP IV inhibitor)	dipeptidyl peptidase IV inhibitor	2	TG	[80-81],[350-351]
8902	dipeptidyl peptidase IV inhibitor (DPP IV inhibitor)	dipeptidyl peptidase IV inhibitor	1	TH	[88-89]
8903	dipeptidyl peptidase IV inhibitor (DPP IV inhibitor)	dipeptidyl peptidase IV inhibitor	2	TI	[222-223],[321-322]
8904	dipeptidyl peptidase IV inhibitor (DPP IV inhibitor)	dipeptidyl peptidase IV inhibitor	5	TK	[5-6],[162-163],[224-225],[393-394],[407-408]
8905	dipeptidyl peptidase IV inhibitor (DPP IV inhibitor)	dipeptidyl peptidase IV inhibitor	2	TL	[309-310],[483-484]
8908	dipeptidyl peptidase IV inhibitor (DPP IV inhibitor)	dipeptidyl peptidase IV inhibitor	1	TQ	[178-179]
8910	dipeptidyl peptidase IV inhibitor (DPP IV inhibitor)	dipeptidyl peptidase IV inhibitor	1	TS	[305-306]
8911	dipeptidyl peptidase IV inhibitor (DPP IV inhibitor)	dipeptidyl peptidase IV inhibitor	1	TT	[161-162]
8912	dipeptidyl peptidase IV inhibitor (DPP IV inhibitor)	dipeptidyl peptidase IV inhibitor	2	TV	[447-448],[459-460]
8915	dipeptidyl peptidase IV inhibitor (DPP IV inhibitor)	dipeptidyl peptidase IV inhibitor	2	VD	[83-84],[510-511]
8916	dipeptidyl peptidase IV inhibitor (DPP IV inhibitor)	dipeptidyl peptidase IV inhibitor	1	VE	[419-420]
8917	dipeptidyl peptidase IV inhibitor (DPP IV inhibitor)	dipeptidyl peptidase IV inhibitor	4	VF	[154-155],[278-279],[448-449],[513-514]
8921	dipeptidyl peptidase IV inhibitor (DPP IV inhibitor)	dipeptidyl peptidase IV inhibitor	2	VK	[54-55],[164-165]
8922	dipeptidyl peptidase IV inhibitor (DPP IV inhibitor)	dipeptidyl peptidase IV inhibitor	4	VL	[16-17],[18-19],[172-173],[194-195]
8923	dipeptidyl peptidase IV inhibitor (DPP IV inhibitor)	dipeptidyl peptidase IV inhibitor	1	VM	[493-494]
8924	dipeptidyl peptidase IV inhibitor (DPP IV inhibitor)	dipeptidyl peptidase IV inhibitor	2	VN	[270-271],[474-475]
8925	dipeptidyl peptidase IV inhibitor (DPP IV inhibitor)	dipeptidyl peptidase IV inhibitor	2	VQ	[41-42],[264-265]
8926	dipeptidyl peptidase IV inhibitor (DPP IV inhibitor)	dipeptidyl peptidase IV inhibitor	5	VS	[14-15],[216-217],[242-243],[387-388],[413-414]
8932	dipeptidyl peptidase IV inhibitor (DPP IV inhibitor)	dipeptidyl peptidase IV inhibitor	1	YA	[389-390]
8933	dipeptidyl peptidase IV inhibitor (DPP IV inhibitor)	dipeptidyl peptidase IV inhibitor	1	YD	[45-46]
8936	dipeptidyl peptidase IV inhibitor (DPP IV inhibitor)	dipeptidyl peptidase IV inhibitor	1	YG	[366-367]

8938	dipeptidyl peptidase IV inhibitor (DPP IV inhibitor)	dipeptidyl peptidase IV inhibitor	1	YI	[251-252]
8939	dipeptidyl peptidase IV inhibitor (DPP IV inhibitor)	dipeptidyl peptidase IV inhibitor	4	YK	[61-62],[123-124],[132-133],[436-437]
8940	dipeptidyl peptidase IV inhibitor (DPP IV inhibitor)	dipeptidyl peptidase IV inhibitor	1	YL	[262-263]
8942	dipeptidyl peptidase IV inhibitor (DPP IV inhibitor)	dipeptidyl peptidase IV inhibitor	1	YN	[402-403]
8944	dipeptidyl peptidase IV inhibitor (DPP IV inhibitor)	dipeptidyl peptidase IV inhibitor	2	YR	[190-191],[291-292]
8946	dipeptidyl peptidase IV inhibitor (DPP IV inhibitor)	dipeptidyl peptidase IV inhibitor	1	YV	[153-154]
8948	dipeptidyl peptidase IV inhibitor (DPP IV inhibitor)	dipeptidyl peptidase IV inhibitor	1	YY	[60-61]
9476	DPP-III inhibitor	dipeptidyl peptidase III inhibitor	1	YY	[60-61]
9478	DPP-III inhibitor	dipeptidyl peptidase III inhibitor	2	LR	[267-268],[439-440]
9479	DPP-III inhibitor	dipeptidyl peptidase III inhibitor	1	MR	[100-101]
9482	DPP-III inhibitor	dipeptidyl peptidase III inhibitor	1	YL	[262-263]
9483	DPP-III inhibitor	dipeptidyl peptidase III inhibitor	4	YK	[61-62],[123-124],[132-133],[436-437]
9484	DPP-III inhibitor	dipeptidyl peptidase III inhibitor	2	YR	[190-191],[291-292]
9485	DPP-III inhibitor	dipeptidyl peptidase III inhibitor	1	RR	[335-336]
9487	DPP-III inhibitor	dipeptidyl peptidase III inhibitor	4	GE	[284-285],[337-338],[415-416],[507-508]
9488	DPP-III inhibitor	dipeptidyl peptidase III inhibitor	1	GF	[22-23]
9490	DPP-III inhibitor	dipeptidyl peptidase III inhibitor	1	RF	[116-117]
9492	DPP-III inhibitor	dipeptidyl peptidase III inhibitor	1	DA	[209-210]
9493	DPP-III inhibitor	dipeptidyl peptidase III inhibitor	2	HL	[95-96],[425-426]
9495	DPP-III inhibitor	dipeptidyl peptidase III inhibitor	1	HF	[275-276]
9496	DPP-III inhibitor	dipeptidyl peptidase III inhibitor	2	HP	[433-434],[455-456]
9499	DPP-III inhibitor	dipeptidyl peptidase III inhibitor	4	LA	[25-26],[192-193],[484-485],[501-502]
9500	DPP-III inhibitor	dipeptidyl peptidase III inhibitor	2	FA	[23-24],[457-458]
9501	DPP-III inhibitor	dipeptidyl peptidase III inhibitor	1	FR	[117-118]
9502	DPP-III inhibitor	dipeptidyl peptidase III inhibitor	2	FL	[12-13],[266-267]
9504	DPP-III inhibitor	dipeptidyl peptidase III inhibitor	3	PE	[30-31],[287-288],[526-527]
9505	DPP-III inhibitor	dipeptidyl peptidase III inhibitor	4	PF	[249-250],[344-345],[400-401],[456-457]
9508	DPP-III inhibitor	dipeptidyl peptidase III inhibitor	1	YG	[366-367]
9510	DPP-III inhibitor	dipeptidyl peptidase III inhibitor	1	YI	[251-252]
9511	DPP-III inhibitor	dipeptidyl peptidase III inhibitor	1	KA	[324-325]
8247	CaMPDE inhibitor	CaMPDE inhibitor	3	IR	[330-331],[443-444],[480-481]
8249	CaMPDE inhibitor	CaMPDE inhibitor	2	KF	[6-7],[176-177]
8250	CaMPDE inhibitor	CaMPDE inhibitor	1	EF	[520-521]
2835	Renin inhibitor	renin inhibitor	3	FT	[160-161],[177-178],[345-346]
2842	Renin inhibitor	renin inhibitor	2	LR	[267-268],[439-440]
8246	renin inhibitor	renin inhibitor	3	IR	[330-331],[443-444],[480-481]

8248	Renin inhibitor	renin inhibitor	2	KF	[6-7],[176-177]
8251	Renin inhibitor	renin inhibitor	1	EF	[520-521]
9431	Renin inhibitor	renin inhibitor	1	QF	[265-266]
9432	Renin inhibitor	renin inhibitor	3	SF	[8-9],[289-290],[352-353]
9433	Renin inhibitor	renin inhibitor	1	YA	[389-390]
9470	Renin inhibitor	renin inhibitor	1	LY	[261-262]

Table S7. Profile of potential biological activity of fragments of protein Ana o 1.0102.

ID	Name of peptide	Activity	Number	Sequence	Location
3458	Prolyl endopeptidase inhibitor	antiamnestic	1	GPGG	[279-282]
3460	Prolyl endopeptidase inhibitor	antiamnestic	2	PG	[271-272],[280-281]
3461	Prolyl endopeptidase inhibitor	antiamnestic	3	GP	[279-280],[337-338],[523-524]
3257	beta-lactokinin	ACE inhibitor	1	RL	[189-190]
3258	beta-lactokinin	ACE inhibitor	2	IR	[441-442],[478-479]
3377	ACE inhibitor (from bovine as1-CN)	ACE inhibitor	1	FGK	[512-514]
3380	ACE inhibitor	ACE inhibitor	2	RY	[120-121],[479-480]
3381	ACE inhibitor	ACE inhibitor	1	LY	[259-260]
3384	ACE inhibitor	ACE inhibitor	4	VF	[152-153],[276-277],[446-447],[511-512]
3421	ACE inhibitor	ACE inhibitor	1	LVL	[15-17]
3489	ACE inhibitor from sake lees	ACE inhibitor	1	RF	[114-115]
3543	ACE inhibitor	ACE inhibitor	1	LRP	[265-267]
3551	ACE inhibitor (from bovine beta-Lg)	ACE inhibitor	4	LF	[9-10],[311-312],[353-354],[367-368]
3553	ACE inhibitor	ACE inhibitor	1	YG	[364-365]
3556	ACE inhibitor	ACE inhibitor	3	FY	[248-249],[288-289],[399-400]
3666	ACE inhibitor	ACE inhibitor	1	YP	[375-376]
3713	ACE inhibitor from alpha-zein	ACE inhibitor	1	LLP	[171-173]
7511	ACE inhibitor from Alaskan pollack skin	ACE inhibitor	1	LPG	[270-272]
7512	ACE inhibitor from Alaskan pollack skin	ACE inhibitor	3	GP	[279-280],[337-338],[523-524]
7513	ACE inhibitor from Alaskan pollack skin	ACE inhibitor	1	PL	[377-378]
7544	ACE inhibitor	ACE inhibitor	1	IW	[340-341]
7558	ACE inhibitor from buckwheat	ACE inhibitor	2	VK	[52-53],[162-163]
7559	ACE inhibitor from buckwheat	ACE inhibitor	1	PSY	[432-434]
7562	ACE inhibitor from soy hydrolysate	ACE inhibitor	3	IA	[250-251],[407-408],[419-420]
7582	ACE inhibitor	ACE inhibitor	1	RP	[266-267]
7583	ACE inhibitor	ACE inhibitor	3	AF	[199-200],[291-292],[500-501]
7585	ACE inhibitor	ACE inhibitor	4	LA	[23-24],[190-191],[482-483],[499-500]
7586	ACE inhibitor	ACE inhibitor	2	KR	[131-132],[228-229]
7587	ACE inhibitor	ACE inhibitor	3	VP	[202-203],[397-398],[449-450]

7588	ACE inhibitor	ACE inhibitor	5	RA	[290-291],[329-330],[403-404],[438-439],[533-534]
7589	ACE inhibitor	ACE inhibitor	1	YA	[387-388]
7590	ACE inhibitor	ACE inhibitor	1	AA	[299-300]
7591	ACE inhibitor	ACE inhibitor	1	GF	[20-21]
7592	ACE inhibitor	ACE inhibitor	1	FR	[115-116]
7593	ACE inhibitor	ACE inhibitor	1	IF	[211-212]
7597	ACE inhibitor	ACE inhibitor	1	GM	[394-395]
7600	ACE inhibitor	ACE inhibitor	2	AG	[451-452],[483-484]
7601	ACE inhibitor	ACE inhibitor	2	GH	[272-273],[452-453]
7602	ACE inhibitor	ACE inhibitor	2	HL	[93-94],[423-424]
7603	ACE inhibitor	ACE inhibitor	3	GR	[64-65],[217-218],[532-533]
7604	ACE inhibitor	ACE inhibitor	2	KG	[63-64],[392-393]
7605	ACE inhibitor	ACE inhibitor	1	FG	[512-513]
7606	ACE inhibitor	ACE inhibitor	1	DA	[207-208]
7607	ACE inhibitor	ACE inhibitor	1	GS	[349-350]
7608	ACE inhibitor	ACE inhibitor	1	GV	[80-81]
7610	ACE inhibitor	ACE inhibitor	3	GQ	[107-108],[126-127],[365-366]
7611	ACE inhibitor	ACE inhibitor	3	GK	[167-168],[484-485],[513-514]
7612	ACE inhibitor	ACE inhibitor	4	GT	[77-78],[219-220],[245-246],[318-319]
7613	ACE inhibitor	ACE inhibitor	2	WG	[76-77],[216-217]
7614	ACE inhibitor	ACE inhibitor	1	HG	[278-279]
7615	ACE inhibitor	ACE inhibitor	4	GE	[282-283],[335-336],[413-414],[505-506]
7616	ACE inhibitor	ACE inhibitor	4	GG	[79-80],[106-107],[281-282],[393-394]
7617	ACE inhibitor	ACE inhibitor	4	QG	[166-167],[236-237],[317-318],[522-523]
7618	ACE inhibitor	ACE inhibitor	3	SG	[244-245],[412-413],[460-461]
7619	ACE inhibitor	ACE inhibitor	1	LG	[19-20]
7620	ACE inhibitor	ACE inhibitor	1	GD	[237-238]
7621	ACE inhibitor	ACE inhibitor	2	TG	[78-79],[348-349]
7622	ACE inhibitor	ACE inhibitor	6	EG	[105-106],[336-337],[414-415],[475-476],[504-505],[531-532]
7623	ACE inhibitor	ACE inhibitor	4	EA	[142-143],[298-299],[369-370],[495-496]
7625	ACE inhibitor	ACE inhibitor	2	PG	[271-272],[280-281]
7628	ACE inhibitor from k-CN (fr. 67-68)	ACE inhibitor	2	VR	[234-235],[328-329]

7636	ACE inhibitor from k-CN (fr. 78-80)	ACE inhibitor	1	SHP	[430-432]
7637	ACE inhibitor from bonito bowels	ACE inhibitor	1	GHF	[272-274]
7653	ACE inhibitor from wakame	ACE inhibitor	1	KYY	[57-59]
7680	ACE inhibitor from pea vicilin	ACE inhibitor	3	QK	[47-48],[109-110],[177-178]
7682	ACE inhibitor from garlic	ACE inhibitor	1	NY	[129-130]
7684	ACE inhibitor from garlic	ACE inhibitor	2	SY	[386-387],[433-434]
7685	ACE inhibitor from garlic	ACE inhibitor	3	SF	[6-7],[287-288],[350-351]
7691	ACE inhibitor from wakame	ACE inhibitor	3	KY	[57-58],[187-188],[363-364]
7692	ACE inhibitor	ACE inhibitor	2	KF	[4-5],[174-175]
7693	ACE inhibitor from wakame	ACE inhibitor	6	KL	[180-181],[258-259],[310-311],[352-353],[380-381],[436-437]
7697	ACE inhibitor from wakame	ACE inhibitor	4	YK	[59-60],[121-122],[130-131],[434-435]
7698	ACE inhibitor from wakame	ACE inhibitor	2	NK	[227-228],[362-363]
7741	ACE inhibitor	ACE inhibitor	1	RR	[333-334]
7742	ACE inhibitor	ACE inhibitor	1	AR	[439-440]
7743	ACE inhibitor	ACE inhibitor	1	KA	[322-323]
7751	ACE inhibitor from shark meat hydrolysate	ACE inhibitor	1	CF	[469-470]
7823	ACE inhibitor from micro algae	ACE inhibitor	1	FAL	[21-23]
7826	ACE inhibitor	ACE inhibitor	3	EI	[295-296],[418-419],[466-467]
7828	ACE inhibitor	ACE inhibitor	3	EV	[275-276],[471-472],[507-508]
7829	ACE inhibitor	ACE inhibitor	1	VE	[417-418]
7830	ACE inhibitor	ACE inhibitor	2	TE	[164-165],[344-345]
7833	ACE inhibitor	ACE inhibitor	1	PT	[2-3]
7834	ACE inhibitor	ACE inhibitor	1	TQ	[176-177]
7836	ACE inhibitor	ACE inhibitor	2	PP	[1-2],[376-377]
7837	ACE inhibitor	ACE inhibitor	1	PQ	[197-198]
7838	ACE inhibitor	ACE inhibitor	2	EW	[75-76],[525-526]
7839	ACE inhibitor	ACE inhibitor	2	ME	[492-493],[503-504]
7840	ACE inhibitor	ACE inhibitor	10	EK	[56-57],[61-62],[91-92],[186-187],[257-258],[309-310],[313-314],[379-380],[493-494],[529-530]
7841	ACE inhibitor	ACE inhibitor	9	KE	[48-49],[53-54],[60-61],[123-124],[325-326],[494-495],[497-498],[528-529],[530-531]
7842	ACE inhibitor	ACE inhibitor	2	HP	[431-432],[453-454]
7843	ACE inhibitor	ACE inhibitor	1	PH	[422-423]

8193	ACE inhibitor	ACE inhibitor	1	AI	[408-409]
8951	ACE inhibitor	ACE inhibitor	1	AV	[191-192]
9037	ACE inhibitor	ACE inhibitor	1	GKV	[167-169]
9048	ACE inhibitor	ACE inhibitor	1	LVQ	[261-263]
9053	ACE inhibitor	ACE inhibitor	1	FYN	[399-401]
9060	ACE inhibitor	ACE inhibitor	1	AVL	[191-193]
9064	ACE inhibitor	ACE inhibitor	3	LEK	[185-187],[308-310],[378-380]
9072	ACE inhibitor	ACE inhibitor	1	DY	[374-375]
9073	ACE inhibitor	ACE inhibitor	1	TP	[246-247]
9074	ACE inhibitor	ACE inhibitor	1	DF	[157-158]
9075	ACE inhibitor	ACE inhibitor	1	DM	[382-383]
9076	ACE inhibitor	ACE inhibitor	1	FQ	[521-522]
9077	ACE inhibitor	ACE inhibitor	1	YV	[151-152]
9079	ACE inhibitor	ACE inhibitor	2	IL	[224-225],[296-297]
9146	ACE inhibitor	ACE inhibitor	1	QGP	[522-524]
9173	ACE inhibitor	ACE inhibitor	3	RG	[125-126],[218-219],[334-335]
9183	ACE inhibitor	ACE inhibitor	1	GTG	[77-79]
9184	ACE inhibitor	ACE inhibitor	2	ST	[85-86],[347-348]
9185	ACE inhibitor	ACE inhibitor	1	YN	[400-401]
9213	ACE inhibitor	ACE inhibitor	2	LR	[265-266],[437-438]
9731	ACE inhibitor	ACE inhibitor	1	VVL	[169-171]
3283	Antithrombotic peptide	antithrombotic	3	GP	[279-280],[337-338],[523-524]
3285	Antithrombotic peptide	antithrombotic	2	PG	[271-272],[280-281]
3354	Antithrombotic peptide	antithrombotic	1	DEE	[516-518]
3462		antithrombotic	1	GPGG	[279-282]
2882	Immunostimulating peptide	immunomodulating	1	YG	[364-365]
3351	Stimulating vasoactive substance release	stimulating	5	EEE	[70-72],[71-73],[72-74],[73-75],[144-146]
3355	Stimulating vasoactive substance release	stimulating	1	SSS	[425-427]
8320	Glucose uptake stimulating peptide	stimulating	4	VL	[14-15],[16-17],[170-171],[192-193]
8321	Glucose uptake stimulating peptide	stimulating	4	LV	[11-12],[15-16],[193-194],[261-262]
8322	Glucose uptake stimulating peptide	stimulating	4	IV	[239-240],[409-410],[448-449],[467-468]
8323	Glucose uptake stimulating peptide	stimulating	2	IL	[224-225],[296-297]

8325	Glucose uptake stimulating peptide	stimulating	1	II	[488-489]
8326	Glucose uptake stimulating peptide	stimulating	2	LL	[171-172],[181-182]
8329	Stimulating vasoactive substance release	stimulating	10	EE	[70-71],[71-72],[72-73],[73-74],[74-75],[144-145],[145-146],[345-346],[506-507],[517-518]
3066	Immunostimulating peptide	immunostimulating	2	EAE	[142-144],[369-371]
2890	neuropeptide	neuropeptide	3	GQ	[107-108],[126-127],[365-366]
8310	Anxiolytic peptide	neuropeptide	1	YL	[260-261]
9534	Kyotorphin	neuropeptide	2	YR	[188-189],[289-290]
2749	peptide regulating ion flow	regulating	1	DY	[374-375]
2753	peptide regulating the stomach mucosal membrane activity	regulating	3	GP	[279-280],[337-338],[523-524]
2754	peptide regulating the stomach mucosal membrane activity	regulating	2	PG	[271-272],[280-281]
2755	peptide regulating the stomach mucosal membrane activity	regulating	1	GPGG	[279-282]
3305		antioxidative	1	LH	[182-183]
3317		antioxidative	2	HL	[93-94],[423-424]
7863	peptide from prawn muscle (Penaeus japonicus)	antioxidative	1	FKK	[354-356]
7872	peptide from soybean protein isolates: beta-conglycinin and glycinin	antioxidative	1	LY	[259-260]
7888	antioxidative peptide	antioxidative	2	EL	[29-30],[498-499]
7931	synthetic peptide	antioxidative	1	KYY	[57-59]
7937	synthetic peptide	antioxidative	1	YYK	[58-60]
7988	synthetic peptide	antioxidative	1	LHA	[182-184]
8029	synthetic peptide	antioxidative	1	PHL	[422-424]
8114	peptide derived from sardinelle by-products proteins (Sardinella aurita)	antioxidative	1	GGE	[281-283]
8130	peptide derived from dried bonito	antioxidative	1	EAK	[495-497]
8134	peptide derived from dried bonito	antioxidative	3	KD	[305-306],[356-357],[443-444]
8139	synthetic peptide	antioxidative	1	PEL	[28-30]
8215	Antioxidative peptide	antioxidative	2	IR	[441-442],[478-479]
8217	Antioxidative peptide	antioxidative	2	LK	[30-31],[301-302]
9082	Antioxidative peptide	antioxidative	2	WG	[76-77],[216-217]
9363	Antioxidative peptide	antioxidative	1	NEN	[462-464]
9368	Antioxidative peptide	antioxidative	1	EQC	[49-51]
3751		bacterial permease ligand	5	KK	[62-63],[122-123],[355-356],[435-436],[485-486]

4005		activating ubiquitin-mediated proteolysis	5	RA	[290-291],[329-330],[403-404],[438-439],[533-534]
4006	Ubiqitin-mediated proteolysis activating peptide	activating ubiquitin-mediated proteolysis	4	LA	[23-24],[190-191],[482-483],[499-500]
9580	Hypolipidemic peptide	hypolipidemic	1	EF	[518-519]
9548	Alpha-glucosidase inhibitor	alpha-glucosidase inhibitor	1	YP	[375-376]
9650	Alpha-glucosidase inhibitor	alpha-glucosidase inhibitor	4	EA	[142-143],[298-299],[369-370],[495-496]
9651	Alpha-glucosidase inhibitor	alpha-glucosidase inhibitor	2	PP	[1-2],[376-377]
9693	Alpha-glucosidase inhibitor	alpha-glucosidase inhibitor	1	VE	[417-418]
9694	Alpha-glucosidase inhibitor	alpha-glucosidase inhibitor	3	PE	[28-29],[285-286],[524-525]
9695	Alpha-glucosidase inhibitor	alpha-glucosidase inhibitor	2	AD	[208-209],[534-535]
9383	HMG-CoA reductase inhibitor	HMG-CoA reductase inhibitor	1	GGV	[79-81]
3169	dipeptidyl peptidase IV inhibitor (DPP IV inhibitor)	dipeptidyl peptidase IV inhibitor	3	GP	[279-280],[337-338],[523-524]
3170	dipeptidyl peptidase IV inhibitor (DPP IV inhibitor)	dipeptidyl peptidase IV inhibitor	2	PP	[1-2],[376-377]
3172	dipeptidyl peptidase IV inhibitor (DPP IV inhibitor)	dipeptidyl peptidase IV inhibitor	2	VA	[194-195],[458-459]
3174	dipeptidyl peptidase IV inhibitor (DPP IV inhibitor)	dipeptidyl peptidase IV inhibitor	1	KA	[322-323]
3175	dipeptidyl peptidase IV inhibitor (DPP IV inhibitor)	dipeptidyl peptidase IV inhibitor	4	LA	[23-24],[190-191],[482-483],[499-500]
3176	dipeptidyl peptidase IV inhibitor (DPP IV inhibitor)	dipeptidyl peptidase IV inhibitor	2	FA	[21-22],[455-456]
3179	dipeptidyl peptidase IV inhibitor (DPP IV inhibitor)	dipeptidyl peptidase IV inhibitor	2	PA	[89-90],[450-451]
3180	dipeptidyl peptidase IV inhibitor (DPP IV inhibitor)	dipeptidyl peptidase IV inhibitor	2	LP	[172-173],[270-271]
3181	dipeptidyl peptidase IV inhibitor (DPP IV inhibitor)	dipeptidyl peptidase IV inhibitor	3	VP	[202-203],[397-398],[449-450]
3182	dipeptidyl peptidase IV inhibitor (DPP IV inhibitor)	dipeptidyl peptidase IV inhibitor	2	LL	[171-172],[181-182]
3183	dipeptidyl peptidase IV inhibitor (DPP IV inhibitor)	dipeptidyl peptidase IV inhibitor	4	VV	[169-170],[201-202],[384-385],[410-411]
3184	dipeptidyl peptidase IV inhibitor (DPP IV inhibitor)	dipeptidyl peptidase IV inhibitor	1	HA	[183-184]
8503	Dipeptidyl peptidase IV inhibitor (DPP IV inhibitor)	dipeptidyl peptidase IV inhibitor	1	TP	[246-247]
8504	Dipeptidyl peptidase IV inhibitor (DPP IV inhibitor)	dipeptidyl peptidase IV inhibitor	1	WP	[341-342]
8518	dipeptidyl peptidase IV inhibitor (DPP IV inhibitor)	dipeptidyl peptidase IV inhibitor	1	RP	[266-267]
8520	dipeptidyl peptidase IV inhibitor (DPP IV inhibitor)	dipeptidyl peptidase IV inhibitor	2	HP	[431-432],[453-454]
8521	dipeptidyl peptidase IV inhibitor (DPP IV inhibitor)	dipeptidyl peptidase IV inhibitor	1	YP	[375-376]
8525	dipeptidyl peptidase IV inhibitor (DPP IV inhibitor)	dipeptidyl peptidase IV inhibitor	3	IA	[250-251],[407-408],[419-420]
8526	dipeptidyl peptidase IV inhibitor (DPP IV inhibitor)	dipeptidyl peptidase IV inhibitor	5	RA	[290-291],[329-330],[403-404],[438-439],[533-534]
8529	dipeptidyl peptidase IV inhibitor (DPP IV inhibitor)	dipeptidyl peptidase IV inhibitor	2	EP	[83-84],[88-89]
8530	dipeptidyl peptidase IV inhibitor (DPP IV inhibitor)	dipeptidyl peptidase IV inhibitor	3	NP	[149-150],[196-197],[284-285]

8555	dipeptidyl peptidase IV inhibitor (DPP IV inhibitor)	dipeptidyl peptidase IV inhibitor	2	FL	[10-11],[264-265]
8557	dipeptidyl peptidase IV inhibitor (DPP IV inhibitor)	dipeptidyl peptidase IV inhibitor	2	HL	[93-94],[423-424]
8558	dipeptidyl peptidase IV inhibitor (DPP IV inhibitor)	dipeptidyl peptidase IV inhibitor	10	EK	[56-57],[61-62],[91-92],[186-187],[257-258],[309-310],[313-314],[379-380],[493-494],[529-530]
8559	dipeptidyl peptidase IV inhibitor (DPP IV inhibitor)	dipeptidyl peptidase IV inhibitor	3	AL	[22-23],[184-185],[300-301]
8560	dipeptidyl peptidase IV inhibitor (DPP IV inhibitor)	dipeptidyl peptidase IV inhibitor	1	SL	[8-9]
8594	dipeptidyl peptidase IV inhibitor (DPP IV inhibitor)	dipeptidyl peptidase IV inhibitor	2	VR	[234-235],[328-329]
8598	dipeptidyl peptidase IV inhibitor (DPP IV inhibitor)	dipeptidyl peptidase IV inhibitor	1	WRK	[526-528]
8637	dipeptidyl peptidase IV inhibitor (DPP IV inhibitor)	dipeptidyl peptidase IV inhibitor	1	AA	[299-300]
8638	dipeptidyl peptidase IV inhibitor (DPP IV inhibitor)	dipeptidyl peptidase IV inhibitor	1	PL	[377-378]
8652	dipeptidyl peptidase IV inhibitor (DPP IV inhibitor)	dipeptidyl peptidase IV inhibitor	1	PPL	[376-378]
8675	dipeptidyl peptidase IV inhibitor (DPP IV inhibitor)	dipeptidyl peptidase IV inhibitor	1	WR	[526-527]
8691	dipeptidyl peptidase IV inhibitor (DPP IV inhibitor)	dipeptidyl peptidase IV inhibitor	1	WE	[294-295]
8696	dipeptidyl peptidase IV inhibitor (DPP IV inhibitor)	dipeptidyl peptidase IV inhibitor	1	YT	[480-481]
8697	dipeptidyl peptidase IV inhibitor (DPP IV inhibitor)	dipeptidyl peptidase IV inhibitor	2	WG	[76-77],[216-217]
8757	dipeptidyl peptidase IV inhibitor (DPP IV inhibitor)	dipeptidyl peptidase IV inhibitor	2	AD	[208-209],[534-535]
8758	dipeptidyl peptidase IV inhibitor (DPP IV inhibitor)	dipeptidyl peptidase IV inhibitor	4	AE	[90-91],[143-144],[370-371],[474-475]
8759	dipeptidyl peptidase IV inhibitor (DPP IV inhibitor)	dipeptidyl peptidase IV inhibitor	3	AF	[199-200],[291-292],[500-501]
8760	dipeptidyl peptidase IV inhibitor (DPP IV inhibitor)	dipeptidyl peptidase IV inhibitor	2	AG	[451-452],[483-484]
8762	dipeptidyl peptidase IV inhibitor (DPP IV inhibitor)	dipeptidyl peptidase IV inhibitor	2	AS	[323-324],[459-460]
8763	dipeptidyl peptidase IV inhibitor (DPP IV inhibitor)	dipeptidyl peptidase IV inhibitor	2	AT	[404-405],[456-457]
8764	dipeptidyl peptidase IV inhibitor (DPP IV inhibitor)	dipeptidyl peptidase IV inhibitor	1	AV	[191-192]
8767	dipeptidyl peptidase IV inhibitor (DPP IV inhibitor)	dipeptidyl peptidase IV inhibitor	2	DP	[27-28],[357-358]
8768	dipeptidyl peptidase IV inhibitor (DPP IV inhibitor)	dipeptidyl peptidase IV inhibitor	1	DQ	[316-317]
8770	dipeptidyl peptidase IV inhibitor (DPP IV inhibitor)	dipeptidyl peptidase IV inhibitor	6	EG	[105-106],[336-337],[414-415],[475-476],[504-505],[531-532]
8771	dipeptidyl peptidase IV inhibitor (DPP IV inhibitor)	dipeptidyl peptidase IV inhibitor	1	EH	[68-69]
8772	dipeptidyl peptidase IV inhibitor (DPP IV inhibitor)	dipeptidyl peptidase IV inhibitor	3	EI	[295-296],[418-419],[466-467]
8773	dipeptidyl peptidase IV inhibitor (DPP IV inhibitor)	dipeptidyl peptidase IV inhibitor	3	ES	[230-231],[286-287],[346-347]
8775	dipeptidyl peptidase IV inhibitor (DPP IV inhibitor)	dipeptidyl peptidase IV inhibitor	3	EV	[275-276],[471-472],[507-508]
8776	dipeptidyl peptidase IV inhibitor (DPP IV inhibitor)	dipeptidyl peptidase IV inhibitor	2	EW	[75-76],[525-526]
8779	dipeptidyl peptidase IV inhibitor (DPP IV inhibitor)	dipeptidyl peptidase IV inhibitor	1	FQ	[521-522]
8780	dipeptidyl peptidase IV inhibitor (DPP IV inhibitor)	dipeptidyl peptidase IV inhibitor	1	FR	[115-116]

8781	dipeptidyl peptidase IV inhibitor (DPP IV inhibitor)	dipeptidyl peptidase IV inhibitor	4	GE	[282-283],[335-336],[413-414],[505-506]
8782	dipeptidyl peptidase IV inhibitor (DPP IV inhibitor)	dipeptidyl peptidase IV inhibitor	1	GF	[20-21]
8783	dipeptidyl peptidase IV inhibitor (DPP IV inhibitor)	dipeptidyl peptidase IV inhibitor	4	GG	[79-80],[106-107],[281-282],[393-394]
8784	dipeptidyl peptidase IV inhibitor (DPP IV inhibitor)	dipeptidyl peptidase IV inhibitor	2	GH	[272-273],[452-453]
8786	dipeptidyl peptidase IV inhibitor (DPP IV inhibitor)	dipeptidyl peptidase IV inhibitor	1	GV	[80-81]
8790	dipeptidyl peptidase IV inhibitor (DPP IV inhibitor)	dipeptidyl peptidase IV inhibitor	2	HE	[69-70],[87-88]
8791	dipeptidyl peptidase IV inhibitor (DPP IV inhibitor)	dipeptidyl peptidase IV inhibitor	1	HF	[273-274]
8801	dipeptidyl peptidase IV inhibitor (DPP IV inhibitor)	dipeptidyl peptidase IV inhibitor	1	II	[488-489]
8802	dipeptidyl peptidase IV inhibitor (DPP IV inhibitor)	dipeptidyl peptidase IV inhibitor	2	IL	[224-225],[296-297]
8803	dipeptidyl peptidase IV inhibitor (DPP IV inhibitor)	dipeptidyl peptidase IV inhibitor	1	IM	[320-321]
8804	dipeptidyl peptidase IV inhibitor (DPP IV inhibitor)	dipeptidyl peptidase IV inhibitor	1	IN	[232-233]
8806	dipeptidyl peptidase IV inhibitor (DPP IV inhibitor)	dipeptidyl peptidase IV inhibitor	2	IR	[441-442],[478-479]
8807	dipeptidyl peptidase IV inhibitor (DPP IV inhibitor)	dipeptidyl peptidase IV inhibitor	1	IW	[340-341]
8808	dipeptidyl peptidase IV inhibitor (DPP IV inhibitor)	dipeptidyl peptidase IV inhibitor	9	KE	[48-49],[53-54],[60-61],[123-124],[325-326],[494-495],[497-498],[528-529],[530-531]
8809	dipeptidyl peptidase IV inhibitor (DPP IV inhibitor)	dipeptidyl peptidase IV inhibitor	2	KF	[4-5],[174-175]
8810	dipeptidyl peptidase IV inhibitor (DPP IV inhibitor)	dipeptidyl peptidase IV inhibitor	2	KG	[63-64],[392-393]
8811	dipeptidyl peptidase IV inhibitor (DPP IV inhibitor)	dipeptidyl peptidase IV inhibitor	2	KH	[34-35],[92-93]
8812	dipeptidyl peptidase IV inhibitor (DPP IV inhibitor)	dipeptidyl peptidase IV inhibitor	4	KI	[25-26],[223-224],[339-340],[406-407]
8813	dipeptidyl peptidase IV inhibitor (DPP IV inhibitor)	dipeptidyl peptidase IV inhibitor	5	KK	[62-63],[122-123],[355-356],[435-436],[485-486]
8814	dipeptidyl peptidase IV inhibitor (DPP IV inhibitor)	dipeptidyl peptidase IV inhibitor	2	KR	[131-132],[228-229]
8815	dipeptidyl peptidase IV inhibitor (DPP IV inhibitor)	dipeptidyl peptidase IV inhibitor	2	KS	[178-179],[428-429]
8816	dipeptidyl peptidase IV inhibitor (DPP IV inhibitor)	dipeptidyl peptidase IV inhibitor	2	KT	[163-164],[302-303]
8817	dipeptidyl peptidase IV inhibitor (DPP IV inhibitor)	dipeptidyl peptidase IV inhibitor	5	KV	[38-39],[161-162],[168-169],[490-491],[510-511]
8819	dipeptidyl peptidase IV inhibitor (DPP IV inhibitor)	dipeptidyl peptidase IV inhibitor	3	KY	[57-58],[187-188],[363-364]
8820	dipeptidyl peptidase IV inhibitor (DPP IV inhibitor)	dipeptidyl peptidase IV inhibitor	1	LH	[182-183]
8825	dipeptidyl peptidase IV inhibitor (DPP IV inhibitor)	dipeptidyl peptidase IV inhibitor	4	LV	[11-12],[15-16],[193-194],[261-262]
8826	dipeptidyl peptidase IV inhibitor (DPP IV inhibitor)	dipeptidyl peptidase IV inhibitor	2	ME	[492-493],[503-504]
8831	dipeptidyl peptidase IV inhibitor (DPP IV inhibitor)	dipeptidyl peptidase IV inhibitor	1	MK	[321-322]
8836	dipeptidyl peptidase IV inhibitor (DPP IV inhibitor)	dipeptidyl peptidase IV inhibitor	1	MR	[98-99]
8837	dipeptidyl peptidase IV inhibitor (DPP IV inhibitor)	dipeptidyl peptidase IV inhibitor	1	MV	[383-384]
8839	dipeptidyl peptidase IV inhibitor (DPP IV inhibitor)	dipeptidyl peptidase IV inhibitor	1	NA	[473-474]

8840	dipeptidyl peptidase IV inhibitor (DPP IV inhibitor)	dipeptidyl peptidase IV inhibitor	1	ND	[253-254]
8841	dipeptidyl peptidase IV inhibitor (DPP IV inhibitor)	dipeptidyl peptidase IV inhibitor	2	NE	[256-257],[462-463]
8845	dipeptidyl peptidase IV inhibitor (DPP IV inhibitor)	dipeptidyl peptidase IV inhibitor	2	NL	[269-270],[464-465]
8847	dipeptidyl peptidase IV inhibitor (DPP IV inhibitor)	dipeptidyl peptidase IV inhibitor	1	NN	[252-253]
8851	dipeptidyl peptidase IV inhibitor (DPP IV inhibitor)	dipeptidyl peptidase IV inhibitor	1	NV	[233-234]
8853	dipeptidyl peptidase IV inhibitor (DPP IV inhibitor)	dipeptidyl peptidase IV inhibitor	1	NY	[129-130]
8854	dipeptidyl peptidase IV inhibitor (DPP IV inhibitor)	dipeptidyl peptidase IV inhibitor	4	PF	[247-248],[342-343],[398-399],[454-455]
8855	dipeptidyl peptidase IV inhibitor (DPP IV inhibitor)	dipeptidyl peptidase IV inhibitor	2	PG	[271-272],[280-281]
8856	dipeptidyl peptidase IV inhibitor (DPP IV inhibitor)	dipeptidyl peptidase IV inhibitor	1	PH	[422-423]
8858	dipeptidyl peptidase IV inhibitor (DPP IV inhibitor)	dipeptidyl peptidase IV inhibitor	2	PK	[173-174],[338-339]
8861	dipeptidyl peptidase IV inhibitor (DPP IV inhibitor)	dipeptidyl peptidase IV inhibitor	1	PQ	[197-198]
8862	dipeptidyl peptidase IV inhibitor (DPP IV inhibitor)	dipeptidyl peptidase IV inhibitor	4	PS	[84-85],[203-204],[358-359],[432-433]
8863	dipeptidyl peptidase IV inhibitor (DPP IV inhibitor)	dipeptidyl peptidase IV inhibitor	1	PT	[2-3]
8864	dipeptidyl peptidase IV inhibitor (DPP IV inhibitor)	dipeptidyl peptidase IV inhibitor	1	PV	[267-268]
8866	dipeptidyl peptidase IV inhibitor (DPP IV inhibitor)	dipeptidyl peptidase IV inhibitor	1	PY	[150-151]
8867	dipeptidyl peptidase IV inhibitor (DPP IV inhibitor)	dipeptidyl peptidase IV inhibitor	1	QA	[198-199]
8868	dipeptidyl peptidase IV inhibitor (DPP IV inhibitor)	dipeptidyl peptidase IV inhibitor	2	QD	[315-316],[515-516]
8869	dipeptidyl peptidase IV inhibitor (DPP IV inhibitor)	dipeptidyl peptidase IV inhibitor	2	QE	[104-105],[118-119]
8870	dipeptidyl peptidase IV inhibitor (DPP IV inhibitor)	dipeptidyl peptidase IV inhibitor	1	QF	[263-264]
8871	dipeptidyl peptidase IV inhibitor (DPP IV inhibitor)	dipeptidyl peptidase IV inhibitor	4	QG	[166-167],[236-237],[317-318],[522-523]
8872	dipeptidyl peptidase IV inhibitor (DPP IV inhibitor)	dipeptidyl peptidase IV inhibitor	1	QH	[127-128]
8874	dipeptidyl peptidase IV inhibitor (DPP IV inhibitor)	dipeptidyl peptidase IV inhibitor	2	QL	[111-112],[366-367]
8876	dipeptidyl peptidase IV inhibitor (DPP IV inhibitor)	dipeptidyl peptidase IV inhibitor	2	QQ	[46-47],[108-109]
8877	dipeptidyl peptidase IV inhibitor (DPP IV inhibitor)	dipeptidyl peptidase IV inhibitor	1	QS	[360-361]
8879	dipeptidyl peptidase IV inhibitor (DPP IV inhibitor)	dipeptidyl peptidase IV inhibitor	1	QV	[327-328]
8881	dipeptidyl peptidase IV inhibitor (DPP IV inhibitor)	dipeptidyl peptidase IV inhibitor	1	QY	[42-43]
8882	dipeptidyl peptidase IV inhibitor (DPP IV inhibitor)	dipeptidyl peptidase IV inhibitor	3	RG	[125-126],[218-219],[334-335]
8884	dipeptidyl peptidase IV inhibitor (DPP IV inhibitor)	dipeptidyl peptidase IV inhibitor	2	RI	[372-373],[440-441]
8885	dipeptidyl peptidase IV inhibitor (DPP IV inhibitor)	dipeptidyl peptidase IV inhibitor	2	RK	[442-443],[527-528]
8886	dipeptidyl peptidase IV inhibitor (DPP IV inhibitor)	dipeptidyl peptidase IV inhibitor	1	RL	[189-190]
8889	dipeptidyl peptidase IV inhibitor (DPP IV inhibitor)	dipeptidyl peptidase IV inhibitor	1	RR	[333-334]
8891	dipeptidyl peptidase IV inhibitor (DPP IV inhibitor)	dipeptidyl peptidase IV inhibitor	3	SF	[6-7],[287-288],[350-351]

8892	dipeptidyl peptidase IV inhibitor (DPP IV inhibitor)	dipeptidyl peptidase IV inhibitor	2	SH	[204-205],[430-431]
8893	dipeptidyl peptidase IV inhibitor (DPP IV inhibitor)	dipeptidyl peptidase IV inhibitor	3	SI	[210-211],[231-232],[241-242]
8894	dipeptidyl peptidase IV inhibitor (DPP IV inhibitor)	dipeptidyl peptidase IV inhibitor	4	SK	[179-180],[304-305],[324-325],[427-428]
8895	dipeptidyl peptidase IV inhibitor (DPP IV inhibitor)	dipeptidyl peptidase IV inhibitor	2	SV	[13-14],[396-397]
8896	dipeptidyl peptidase IV inhibitor (DPP IV inhibitor)	dipeptidyl peptidase IV inhibitor	2	SW	[215-216],[293-294]
8897	dipeptidyl peptidase IV inhibitor (DPP IV inhibitor)	dipeptidyl peptidase IV inhibitor	2	SY	[386-387],[433-434]
8899	dipeptidyl peptidase IV inhibitor (DPP IV inhibitor)	dipeptidyl peptidase IV inhibitor	2	TE	[164-165],[344-345]
8901	dipeptidyl peptidase IV inhibitor (DPP IV inhibitor)	dipeptidyl peptidase IV inhibitor	2	TG	[78-79],[348-349]
8902	dipeptidyl peptidase IV inhibitor (DPP IV inhibitor)	dipeptidyl peptidase IV inhibitor	1	TH	[86-87]
8903	dipeptidyl peptidase IV inhibitor (DPP IV inhibitor)	dipeptidyl peptidase IV inhibitor	2	TI	[220-221],[319-320]
8904	dipeptidyl peptidase IV inhibitor (DPP IV inhibitor)	dipeptidyl peptidase IV inhibitor	5	TK	[3-4],[160-161],[222-223],[391-392],[405-406]
8905	dipeptidyl peptidase IV inhibitor (DPP IV inhibitor)	dipeptidyl peptidase IV inhibitor	2	TL	[307-308],[481-482]
8908	dipeptidyl peptidase IV inhibitor (DPP IV inhibitor)	dipeptidyl peptidase IV inhibitor	1	TQ	[176-177]
8910	dipeptidyl peptidase IV inhibitor (DPP IV inhibitor)	dipeptidyl peptidase IV inhibitor	1	TS	[303-304]
8911	dipeptidyl peptidase IV inhibitor (DPP IV inhibitor)	dipeptidyl peptidase IV inhibitor	1	TT	[159-160]
8912	dipeptidyl peptidase IV inhibitor (DPP IV inhibitor)	dipeptidyl peptidase IV inhibitor	2	TV	[445-446],[457-458]
8915	dipeptidyl peptidase IV inhibitor (DPP IV inhibitor)	dipeptidyl peptidase IV inhibitor	2	VD	[81-82],[508-509]
8916	dipeptidyl peptidase IV inhibitor (DPP IV inhibitor)	dipeptidyl peptidase IV inhibitor	1	VE	[417-418]
8917	dipeptidyl peptidase IV inhibitor (DPP IV inhibitor)	dipeptidyl peptidase IV inhibitor	4	VF	[152-153],[276-277],[446-447],[511-512]
8921	dipeptidyl peptidase IV inhibitor (DPP IV inhibitor)	dipeptidyl peptidase IV inhibitor	2	VK	[52-53],[162-163]
8922	dipeptidyl peptidase IV inhibitor (DPP IV inhibitor)	dipeptidyl peptidase IV inhibitor	4	VL	[14-15],[16-17],[170-171],[192-193]
8923	dipeptidyl peptidase IV inhibitor (DPP IV inhibitor)	dipeptidyl peptidase IV inhibitor	1	VM	[491-492]
8924	dipeptidyl peptidase IV inhibitor (DPP IV inhibitor)	dipeptidyl peptidase IV inhibitor	2	VN	[268-269],[472-473]
8925	dipeptidyl peptidase IV inhibitor (DPP IV inhibitor)	dipeptidyl peptidase IV inhibitor	2	VQ	[39-40],[262-263]
8926	dipeptidyl peptidase IV inhibitor (DPP IV inhibitor)	dipeptidyl peptidase IV inhibitor	5	VS	[12-13],[214-215],[240-241],[385-386],[411-412]
8932	dipeptidyl peptidase IV inhibitor (DPP IV inhibitor)	dipeptidyl peptidase IV inhibitor	1	YA	[387-388]
8933	dipeptidyl peptidase IV inhibitor (DPP IV inhibitor)	dipeptidyl peptidase IV inhibitor	1	YD	[43-44]
8936	dipeptidyl peptidase IV inhibitor (DPP IV inhibitor)	dipeptidyl peptidase IV inhibitor	1	YG	[364-365]
8938	dipeptidyl peptidase IV inhibitor (DPP IV inhibitor)	dipeptidyl peptidase IV inhibitor	1	YI	[249-250]
8939	dipeptidyl peptidase IV inhibitor (DPP IV inhibitor)	dipeptidyl peptidase IV inhibitor	4	YK	[59-60],[121-122],[130-131],[434-435]
8940	dipeptidyl peptidase IV inhibitor (DPP IV inhibitor)	dipeptidyl peptidase IV inhibitor	1	YL	[260-261]
8942	dipeptidyl peptidase IV inhibitor (DPP IV inhibitor)	dipeptidyl peptidase IV inhibitor	1	YN	[400-401]

8944	dipeptidyl peptidase IV inhibitor (DPP IV inhibitor)	dipeptidyl peptidase IV inhibitor	2	YR	[188-189],[289-290]
8946	dipeptidyl peptidase IV inhibitor (DPP IV inhibitor)	dipeptidyl peptidase IV inhibitor	1	YV	[151-152]
8948	dipeptidyl peptidase IV inhibitor (DPP IV inhibitor)	dipeptidyl peptidase IV inhibitor	1	YY	[58-59]
9476	DPP-III inhibitor	dipeptidyl peptidase III inhibitor	1	YY	[58-59]
9478	DPP-III inhibitor	dipeptidyl peptidase III inhibitor	2	LR	[265-266],[437-438]
9479	DPP-III inhibitor	dipeptidyl peptidase III inhibitor	1	MR	[98-99]
9482	DPP-III inhibitor	dipeptidyl peptidase III inhibitor	1	YL	[260-261]
9483	DPP-III inhibitor	dipeptidyl peptidase III inhibitor	4	YK	[59-60],[121-122],[130-131],[434-435]
9484	DPP-III inhibitor	dipeptidyl peptidase III inhibitor	2	YR	[188-189],[289-290]
9485	DPP-III inhibitor	dipeptidyl peptidase III inhibitor	1	RR	[333-334]
9487	DPP-III inhibitor	dipeptidyl peptidase III inhibitor	4	GE	[282-283],[335-336],[413-414],[505-506]
9488	DPP-III inhibitor	dipeptidyl peptidase III inhibitor	1	GF	[20-21]
9490	DPP-III inhibitor	dipeptidyl peptidase III inhibitor	1	RF	[114-115]
9492	DPP-III inhibitor	dipeptidyl peptidase III inhibitor	1	DA	[207-208]
9493	DPP-III inhibitor	dipeptidyl peptidase III inhibitor	2	HL	[93-94],[423-424]
9495	DPP-III inhibitor	dipeptidyl peptidase III inhibitor	1	HF	[273-274]
9496	DPP-III inhibitor	dipeptidyl peptidase III inhibitor	2	HP	[431-432],[453-454]
9499	DPP-III inhibitor	dipeptidyl peptidase III inhibitor	4	LA	[23-24],[190-191],[482-483],[499-500]
9500	DPP-III inhibitor	dipeptidyl peptidase III inhibitor	2	FA	[21-22],[455-456]
9501	DPP-III inhibitor	dipeptidyl peptidase III inhibitor	1	FR	[115-116]
9502	DPP-III inhibitor	dipeptidyl peptidase III inhibitor	2	FL	[10-11],[264-265]
9504	DPP-III inhibitor	dipeptidyl peptidase III inhibitor	3	PE	[28-29],[285-286],[524-525]
9505	DPP-III inhibitor	dipeptidyl peptidase III inhibitor	4	PF	[247-248],[342-343],[398-399],[454-455]
9508	DPP-III inhibitor	dipeptidyl peptidase III inhibitor	1	YG	[364-365]
9510	DPP-III inhibitor	dipeptidyl peptidase III inhibitor	1	YI	[249-250]
9511	DPP-III inhibitor	dipeptidyl peptidase III inhibitor	1	KA	[322-323]
8247	CaMPDE inhibitor	CaMPDE inhibitor	2	IR	[441-442],[478-479]
8249	CaMPDE inhibitor	CaMPDE inhibitor	2	KF	[4-5],[174-175]
8250	CaMPDE inhibitor	CaMPDE inhibitor	1	EF	[518-519]
2835	Renin inhibitor	renin inhibitor	3	FT	[158-159],[175-176],[343-344]
2842	Renin inhibitor	renin inhibitor	2	LR	[265-266],[437-438]
8246	renin inhibitor	renin inhibitor	2	IR	[441-442],[478-479]

8248	Renin inhibitor	renin inhibitor	2	KF	[4-5],[174-175]
8251	Renin inhibitor	renin inhibitor	1	EF	[518-519]
9431	Renin inhibitor	renin inhibitor	1	QF	[263-264]
9432	Renin inhibitor	renin inhibitor	3	SF	[6-7],[287-288],[350-351]
9433	Renin inhibitor	renin inhibitor	1	YA	[387-388]
9470	Renin inhibitor	renin inhibitor	1	LY	[259-260]

Table S8. Profile of potential biological activity of fragments of protein Ana o 2.0101.

ID	Name of peptide	Activity	Number	Sequence	Location
3460	Prolyl endopeptidase inhibitor	antiamnestic	1	PG	[99-100]
2653	ACE inhibitor	ACE inhibitor	1	VLP	[331-333]
3257	beta-lactokinin	ACE inhibitor	4	RL	[29-30],[217-218],[281-282],[301-302]
3258	beta-lactokinin	ACE inhibitor	2	IR	[127-128],[242-243]
3381	ACE inhibitor	ACE inhibitor	1	LY	[325-326]
3383	ACE inhibitor	ACE inhibitor	3	IY	[85-86],[294-295],[343-344]
3384	ACE inhibitor	ACE inhibitor	2	VF	[185-186],[362-363]
3386	ACE inhibitor	ACE inhibitor	1	KW	[314-315]
3421	ACE inhibitor	ACE inhibitor	1	LVL	[330-332]
3489	ACE inhibitor from sake lees	ACE inhibitor	3	RF	[119-120],[129-130],[389-390]
3507	ACE inhibitor (beta-LG fr. 78-80)	ACE inhibitor	1	IPA	[138-140]
3528	ACE inhibitor	ACE inhibitor	1	LVR	[63-65]
3537	ACE inhibitor	ACE inhibitor	1	PR	[173-174]
3551	ACE inhibitor (from bovine beta-Lg)	ACE inhibitor	2	LF	[8-9],[199-200]
3553	ACE inhibitor	ACE inhibitor	1	YG	[344-345]
3597	ACE inhibitor	ACE inhibitor	1	AIP	[137-139]
3666	ACE inhibitor	ACE inhibitor	1	YP	[98-99]
3713	ACE inhibitor from alpha-zein	ACE inhibitor	1	LLP	[74-76]
7513	ACE inhibitor from Alaskan pollack skin	ACE inhibitor	1	PL	[405-406]
7543	ACE inhibitor	ACE inhibitor	1	AW	[48-49]
7547	ACE inhibitor	ACE inhibitor	1	IRP	[242-244]
7558	ACE inhibitor from buckwheat	ACE inhibitor	3	VK	[232-233],[234-235],[382-383]
7562	ACE inhibitor from soy hydrolysate	ACE inhibitor	1	IA	[136-137]
7580	ACE inhibitor	ACE inhibitor	1	RW	[265-266]
7581	ACE inhibitor	ACE inhibitor	1	IP	[138-139]
7582	ACE inhibitor	ACE inhibitor	1	RP	[243-244]
7583	ACE inhibitor	ACE inhibitor	2	AF	[211-212],[424-425]
7584	ACE inhibitor	ACE inhibitor	2	AP	[81-82],[107-108]
7585	ACE inhibitor	ACE inhibitor	5	LA	[13-14],[178-179],[208-209],[406-407],[421-422]

7586	ACE inhibitor	ACE inhibitor	2	KR	[263-264],[383-384]
7587	ACE inhibitor	ACE inhibitor	1	VP	[375-376]
7588	ACE inhibitor	ACE inhibitor	3	RA	[291-292],[384-385],[400-401]
7591	ACE inhibitor	ACE inhibitor	1	GF	[202-203]
7592	ACE inhibitor	ACE inhibitor	2	FR	[56-57],[130-131]
7594	ACE inhibitor	ACE inhibitor	1	VG	[299-300]
7596	ACE inhibitor	ACE inhibitor	3	GI	[95-96],[230-231],[272-273]
7597	ACE inhibitor	ACE inhibitor	2	GM	[92-93],[415-416]
7599	ACE inhibitor	ACE inhibitor	1	GL	[72-73]
7600	ACE inhibitor	ACE inhibitor	5	AG	[43-44],[59-60],[140-141],[179-180],[407-408]
7602	ACE inhibitor	ACE inhibitor	1	HL	[177-178]
7603	ACE inhibitor	ACE inhibitor	5	GR	[111-112],[118-119],[196-197],[300-301],[408-409]
7604	ACE inhibitor	ACE inhibitor	3	KG	[322-323],[347-348],[349-350]
7605	ACE inhibitor	ACE inhibitor	1	FG	[358-359]
7606	ACE inhibitor	ACE inhibitor	3	DA	[31-32],[431-432],[456-457]
7607	ACE inhibitor	ACE inhibitor	1	GS	[252-253]
7608	ACE inhibitor	ACE inhibitor	3	GV	[60-61],[141-142],[323-324]
7610	ACE inhibitor	ACE inhibitor	4	GQ	[115-116],[267-268],[350-351],[370-371]
7611	ACE inhibitor	ACE inhibitor	1	GK	[348-349]
7612	ACE inhibitor	ACE inhibitor	1	GT	[44-45]
7613	ACE inhibitor	ACE inhibitor	1	WG	[266-267]
7614	ACE inhibitor	ACE inhibitor	1	HG	[10-11]
7615	ACE inhibitor	ACE inhibitor	3	GE	[90-91],[365-366],[447-448]
7616	ACE inhibitor	ACE inhibitor	2	GG	[229-230],[414-415]
7617	ACE inhibitor	ACE inhibitor	3	QG	[89-90],[110-111],[114-115]
7618	ACE inhibitor	ACE inhibitor	3	SG	[117-118],[201-202],[446-447]
7619	ACE inhibitor	ACE inhibitor	1	LG	[413-414]
7620	ACE inhibitor	ACE inhibitor	1	GD	[133-134]
7621	ACE inhibitor	ACE inhibitor	1	TG	[94-95]
7622	ACE inhibitor	ACE inhibitor	3	EG	[91-92],[149-150],[369-370]
7623	ACE inhibitor	ACE inhibitor	3	EA	[42-43],[47-48],[210-211]
7624	ACE inhibitor	ACE inhibitor	2	NG	[71-72],[271-272]

7625	ACE inhibitor	ACE inhibitor	1	PG	[99-100]
7628	ACE inhibitor from k-CN (fr. 67-68)	ACE inhibitor	2	VR	[64-65],[367-368]
7657	ACE inhibitor from human plasma	ACE inhibitor	1	LIY	[84-86]
7667	ACE inhibitor from k-CN (94-97)	ACE inhibitor	1	IAIP	[136-139]
7680	ACE inhibitor from pea vicilin	ACE inhibitor	1	QK	[125-126]
7681	ACE inhibitor from soy	ACE inhibitor	1	DG	[364-365]
7683	ACE inhibitor from garlic	ACE inhibitor	2	NF	[357-358],[378-379]
7684	ACE inhibitor from garlic	ACE inhibitor	1	SY	[97-98]
7685	ACE inhibitor from garlic	ACE inhibitor	1	SF	[394-395]
7692	ACE inhibitor	ACE inhibitor	2	KF	[175-176],[436-437]
7697	ACE inhibitor from wakame	ACE inhibitor	1	YK	[326-327]
7741	ACE inhibitor	ACE inhibitor	3	RR	[128-129],[131-132],[264-265]
7742	ACE inhibitor	ACE inhibitor	3	AR	[290-291],[385-386],[432-433]
7751	ACE inhibitor from shark meat hydrolysate	ACE inhibitor	1	CF	[4-5]
7752	ACE inhibitor from shark meat hydrolysate	ACE inhibitor	1	EY	[40-41]
7827	ACE inhibitor	ACE inhibitor	1	IE	[273-274]
7828	ACE inhibitor	ACE inhibitor	3	EV	[298-299],[366-367],[419-420]
7829	ACE inhibitor	ACE inhibitor	3	VE	[39-40],[46-47],[320-321]
7830	ACE inhibitor	ACE inhibitor	1	TE	[205-206]
7831	ACE inhibitor	ACE inhibitor	1	LQ	[316-317]
7832	ACE inhibitor	ACE inhibitor	3	LN	[305-306],[308-309],[337-338]
7835	ACE inhibitor	ACE inhibitor	1	AH	[143-144]
7837	ACE inhibitor	ACE inhibitor	4	PQ	[76-77],[82-83],[108-109],[376-377]
7838	ACE inhibitor	ACE inhibitor	2	EW	[18-19],[391-392]
7840	ACE inhibitor	ACE inhibitor	2	EK	[262-263],[321-322]
7841	ACE inhibitor	ACE inhibitor	1	KE	[283-284]
7843	ACE inhibitor	ACE inhibitor	1	PH	[333-334]
8182	ACE Inhibitor	ACE inhibitor	1	ALEP	[32-35]
8184	ACE Inhibitor	ACE inhibitor	1	IQP	[68-70]
8193	ACE inhibitor	ACE inhibitor	1	AI	[137-138]
8951	ACE inhibitor	ACE inhibitor	1	AV	[380-381]
9046	ACE inhibitor	ACE inhibitor	1	VQV	[352-354]

9050	ACE inhibitor	ACE inhibitor	1	VLY	[324-326]
9066	ACE inhibitor	ACE inhibitor	1	YPG	[98-100]
9073	ACE inhibitor	ACE inhibitor	2	TP	[172-173],[296-297]
9076	ACE inhibitor	ACE inhibitor	4	FQ	[120-121],[186-187],[212-213],[425-426]
9077	ACE inhibitor	ACE inhibitor	1	YV	[86-87]
9078	ACE inhibitor	ACE inhibitor	1	YE	[41-42]
9079	ACE inhibitor	ACE inhibitor	2	IL	[7-8],[312-313]
9107	ACE inhibitor	ACE inhibitor	1	WL	[315-316]
9173	ACE inhibitor	ACE inhibitor	4	RG	[132-133],[195-196],[228-229],[251-252]
9185	ACE inhibitor	ACE inhibitor	1	YN	[147-148]
9196	ACE inhibitor	ACE inhibitor	1	AVV	[380-382]
9213	ACE inhibitor	ACE inhibitor	1	LR	[239-240]
9325	ACE inhibitor	ACE inhibitor	1	GVLY	[323-326]
9566	ACE inhibitor	ACE inhibitor	1	QP	[69-70]
9704	ACE inhibitor	ACE inhibitor	1	IIY	[342-344]
9742	ACE inhibitor	ACE inhibitor	1	EKR	[262-264]
3285	Antithrombotic peptide	antithrombotic	1	PG	[99-100]
9660	Antithrombotic peptide	antithrombotic	1	RGD	[132-134]
2882	Immunostimulating peptide	immunomodulating	1	YG	[344-345]
3027	Kentsin	immunomodulating	1	TPRK	[172-175]
3351	Stimulating vasoactive substance release	stimulating	1	EEE	[256-258]
3356	Stimulating vasoactive substance release	stimulating	1	LLL	[73-75]
8320	Glucose uptake stimulating peptide	stimulating	4	VL	[324-325],[331-332],[412-413],[420-421]
8321	Glucose uptake stimulating peptide	stimulating	3	LV	[63-64],[330-331],[373-374]
8322	Glucose uptake stimulating peptide	stimulating	1	IV	[231-232]
8323	Glucose uptake stimulating peptide	stimulating	2	IL	[7-8],[312-313]
8324	Glucose uptake stimulating peptide	stimulating	3	LI	[6-7],[84-85],[218-219]
8325	Glucose uptake stimulating peptide	stimulating	2	II	[135-136],[342-343]
8326	Glucose uptake stimulating peptide	stimulating	4	LL	[73-74],[74-75],[159-160],[207-208]
8329	Stimulating vasoactive substance release	stimulating	5	EE	[256-257],[257-258],[274-275],[387-388],[418-419]
8330	Stimulating vasoactive substance release	stimulating	5	SE	[224-225],[249-250],[253-254],[255-256],[259-260]
2890	neuropeptide	neuropeptide	4	GQ	[115-116],[267-268],[350-351],[370-371]

2754	peptide regulating the stomach mucosal membrane activity	regulating	1	PG	[99-100]
3317		antioxidative	1	HL	[177-178]
3319		antioxidative	1	HH	[451-452]
7872	peptide from soybean protein isolates: beta-conglycinin and glycinin	antioxidative	1	LY	[325-326]
7873	peptide from soybean protein isolates: beta-conglycinin and glycinin	antioxidative	3	IY	[85-86],[294-295],[343-344]
7886	peptide derived from egg white albumin	antioxidative	1	AH	[143-144]
7888	antioxidative peptide	antioxidative	2	EL	[206-207],[238-239]
7922	synthetic peptide	antioxidative	1	SHH	[450-452]
8037	synthetic peptide	antioxidative	1	PHW	[333-335]
8064	synthetic peptide	antioxidative	1	RHQ	[123-125]
8067	synthetic peptide	antioxidative	1	RHT	[65-67]
8073	synthetic peptide	antioxidative	1	RWG	[265-267]
8105	peptide derived from dried bonito	antioxidative	1	VKV	[232-234]
8134	peptide derived from dried bonito	antioxidative	2	KD	[183-184],[235-236]
8214	Antioxidative peptide	antioxidative	1	RW	[265-266]
8215	Antioxidative peptide	antioxidative	2	IR	[127-128],[242-243]
8217	Antioxidative peptide	antioxidative	3	LK	[222-223],[282-283],[313-314]
8219	antioxidative peptide	antioxidative	1	TY	[104-105]
8460	Antioxidant peptide from marine bivalve (Mactra veneriformis)	antioxidative	1	AW	[48-49]
9082	Antioxidative peptide	antioxidative	1	WG	[266-267]
3164	laminin-like peptide	embryotoxic	1	RGD	[132-134]
4005		activating ubiquitin-mediated proteolysis	3	RA	[291-292],[384-385],[400-401]
4006	Ubiqitin-mediated proteolysis activating peptide	activating ubiquitin-mediated proteolysis	5	LA	[13-14],[178-179],[208-209],[406-407],[421-422]
9548	Alpha-glucosidase inhibitor	alpha-glucosidase inhibitor	1	YP	[98-99]
9549	Alpha-glucosidase inhibitor	alpha-glucosidase inhibitor	1	YPG	[98-100]
9650	Alpha-glucosidase inhibitor	alpha-glucosidase inhibitor	3	EA	[42-43],[47-48],[210-211]
9693	Alpha-glucosidase inhibitor	alpha-glucosidase inhibitor	3	VE	[39-40],[46-47],[320-321]
9694	Alpha-glucosidase inhibitor	alpha-glucosidase inhibitor	3	PE	[102-103],[297-298],[417-418]
9695	Alpha-glucosidase inhibitor	alpha-glucosidase inhibitor	1	AD	[292-293]

3171	dipeptidyl peptidase IV inhibitor (DPP IV inhibitor)	dipeptidyl peptidase IV inhibitor	1	MP	[416-417]
3172	dipeptidyl peptidase IV inhibitor (DPP IV inhibitor)	dipeptidyl peptidase IV inhibitor	2	VA	[61-62],[142-143]
3175	dipeptidyl peptidase IV inhibitor (DPP IV inhibitor)	dipeptidyl peptidase IV inhibitor	5	LA	[13-14],[178-179],[208-209],[406-407],[421-422]
3176	dipeptidyl peptidase IV inhibitor (DPP IV inhibitor)	dipeptidyl peptidase IV inhibitor	1	FA	[379-380]
3177	dipeptidyl peptidase IV inhibitor (DPP IV inhibitor)	dipeptidyl peptidase IV inhibitor	2	AP	[81-82],[107-108]
3179	dipeptidyl peptidase IV inhibitor (DPP IV inhibitor)	dipeptidyl peptidase IV inhibitor	2	PA	[139-140],[289-290]
3180	dipeptidyl peptidase IV inhibitor (DPP IV inhibitor)	dipeptidyl peptidase IV inhibitor	3	LP	[75-76],[310-311],[332-333]
3181	dipeptidyl peptidase IV inhibitor (DPP IV inhibitor)	dipeptidyl peptidase IV inhibitor	1	VP	[375-376]
3182	dipeptidyl peptidase IV inhibitor (DPP IV inhibitor)	dipeptidyl peptidase IV inhibitor	4	LL	[73-74],[74-75],[159-160],[207-208]
3183	dipeptidyl peptidase IV inhibitor (DPP IV inhibitor)	dipeptidyl peptidase IV inhibitor	5	VV	[87-88],[154-155],[354-355],[374-375],[381-382]
8304	Dipeptidyl peptidase IV inhibitor (DPP IV inhibitor)	dipeptidyl peptidase IV inhibitor	1	IPA	[138-140]
8501	Dipeptidyl peptidase IV inhibitor (DPP IV inhibitor)	dipeptidyl peptidase IV inhibitor	1	IP	[138-139]
8503	Dipeptidyl peptidase IV inhibitor (DPP IV inhibitor)	dipeptidyl peptidase IV inhibitor	2	TP	[172-173],[296-297]
8505	Dipeptidyl peptidase IV inhibitor (DPP IV inhibitor)	dipeptidyl peptidase IV inhibitor	2	SP	[152-153],[404-405]
8518	dipeptidyl peptidase IV inhibitor (DPP IV inhibitor)	dipeptidyl peptidase IV inhibitor	1	RP	[243-244]
8521	dipeptidyl peptidase IV inhibitor (DPP IV inhibitor)	dipeptidyl peptidase IV inhibitor	1	YP	[98-99]
8525	dipeptidyl peptidase IV inhibitor (DPP IV inhibitor)	dipeptidyl peptidase IV inhibitor	1	IA	[136-137]
8526	dipeptidyl peptidase IV inhibitor (DPP IV inhibitor)	dipeptidyl peptidase IV inhibitor	3	RA	[291-292],[384-385],[400-401]
8529	dipeptidyl peptidase IV inhibitor (DPP IV inhibitor)	dipeptidyl peptidase IV inhibitor	1	EP	[34-35]
8530	dipeptidyl peptidase IV inhibitor (DPP IV inhibitor)	dipeptidyl peptidase IV inhibitor	1	NP	[181-182]
8532	dipeptidyl peptidase IV inhibitor (DPP IV inhibitor)	dipeptidyl peptidase IV inhibitor	1	QP	[69-70]
8555	dipeptidyl peptidase IV inhibitor (DPP IV inhibitor)	dipeptidyl peptidase IV inhibitor	1	FL	[5-6]
8557	dipeptidyl peptidase IV inhibitor (DPP IV inhibitor)	dipeptidyl peptidase IV inhibitor	1	HL	[177-178]
8558	dipeptidyl peptidase IV inhibitor (DPP IV inhibitor)	dipeptidyl peptidase IV inhibitor	2	EK	[262-263],[321-322]
8559	dipeptidyl peptidase IV inhibitor (DPP IV inhibitor)	dipeptidyl peptidase IV inhibitor	3	AL	[32-33],[62-63],[329-330]
8560	dipeptidyl peptidase IV inhibitor (DPP IV inhibitor)	dipeptidyl peptidase IV inhibitor	1	SL	[307-308]
8561	dipeptidyl peptidase IV inhibitor (DPP IV inhibitor)	dipeptidyl peptidase IV inhibitor	1	GL	[72-73]
8594	dipeptidyl peptidase IV inhibitor (DPP IV inhibitor)	dipeptidyl peptidase IV inhibitor	2	VR	[64-65],[367-368]
8638	dipeptidyl peptidase IV inhibitor (DPP IV inhibitor)	dipeptidyl peptidase IV inhibitor	1	PL	[405-406]
8677	dipeptidyl peptidase IV inhibitor (DPP IV inhibitor)	dipeptidyl peptidase IV inhibitor	1	WL	[315-316]
8678	dipeptidyl peptidase IV inhibitor (DPP IV inhibitor)	dipeptidyl peptidase IV inhibitor	1	WQ	[19-20]
8679	dipeptidyl peptidase IV inhibitor (DPP IV inhibitor)	dipeptidyl peptidase IV inhibitor	1	WI	[392-393]

8680	dipeptidyl peptidase IV inhibitor (DPP IV inhibitor)	dipeptidyl peptidase IV inhibitor	1	WN	[335-336]
8684	dipeptidyl peptidase IV inhibitor (DPP IV inhibitor)	dipeptidyl peptidase IV inhibitor	1	WC	[145-146]
8693	dipeptidyl peptidase IV inhibitor (DPP IV inhibitor)	dipeptidyl peptidase IV inhibitor	1	IQP	[68-70]
8695	dipeptidyl peptidase IV inhibitor (DPP IV inhibitor)	dipeptidyl peptidase IV inhibitor	1	AW	[48-49]
8696	dipeptidyl peptidase IV inhibitor (DPP IV inhibitor)	dipeptidyl peptidase IV inhibitor	1	YT	[295-296]
8697	dipeptidyl peptidase IV inhibitor (DPP IV inhibitor)	dipeptidyl peptidase IV inhibitor	1	WG	[266-267]
8757	dipeptidyl peptidase IV inhibitor (DPP IV inhibitor)	dipeptidyl peptidase IV inhibitor	1	AD	[292-293]
8758	dipeptidyl peptidase IV inhibitor (DPP IV inhibitor)	dipeptidyl peptidase IV inhibitor	1	AE	[209-210]
8759	dipeptidyl peptidase IV inhibitor (DPP IV inhibitor)	dipeptidyl peptidase IV inhibitor	2	AF	[211-212],[424-425]
8760	dipeptidyl peptidase IV inhibitor (DPP IV inhibitor)	dipeptidyl peptidase IV inhibitor	5	AG	[43-44],[59-60],[140-141],[179-180],[407-408]
8761	dipeptidyl peptidase IV inhibitor (DPP IV inhibitor)	dipeptidyl peptidase IV inhibitor	1	AH	[143-144]
8762	dipeptidyl peptidase IV inhibitor (DPP IV inhibitor)	dipeptidyl peptidase IV inhibitor	1	AS	[14-15]
8764	dipeptidyl peptidase IV inhibitor (DPP IV inhibitor)	dipeptidyl peptidase IV inhibitor	1	AV	[380-381]
8766	dipeptidyl peptidase IV inhibitor (DPP IV inhibitor)	dipeptidyl peptidase IV inhibitor	4	DN	[36-37],[226-227],[270-271],[356-357]
8767	dipeptidyl peptidase IV inhibitor (DPP IV inhibitor)	dipeptidyl peptidase IV inhibitor	2	DP	[50-51],[288-289]
8769	dipeptidyl peptidase IV inhibitor (DPP IV inhibitor)	dipeptidyl peptidase IV inhibitor	4	DR	[28-29],[122-123],[170-171],[399-400]
8770	dipeptidyl peptidase IV inhibitor (DPP IV inhibitor)	dipeptidyl peptidase IV inhibitor	3	EG	[91-92],[149-150],[369-370]
8773	dipeptidyl peptidase IV inhibitor (DPP IV inhibitor)	dipeptidyl peptidase IV inhibitor	3	ES	[254-255],[258-259],[448-449]
8774	dipeptidyl peptidase IV inhibitor (DPP IV inhibitor)	dipeptidyl peptidase IV inhibitor	2	ET	[103-104],[275-276]
8775	dipeptidyl peptidase IV inhibitor (DPP IV inhibitor)	dipeptidyl peptidase IV inhibitor	3	EV	[298-299],[366-367],[419-420]
8776	dipeptidyl peptidase IV inhibitor (DPP IV inhibitor)	dipeptidyl peptidase IV inhibitor	2	EW	[18-19],[391-392]
8777	dipeptidyl peptidase IV inhibitor (DPP IV inhibitor)	dipeptidyl peptidase IV inhibitor	1	EY	[40-41]
8778	dipeptidyl peptidase IV inhibitor (DPP IV inhibitor)	dipeptidyl peptidase IV inhibitor	1	FN	[437-438]
8779	dipeptidyl peptidase IV inhibitor (DPP IV inhibitor)	dipeptidyl peptidase IV inhibitor	4	FQ	[120-121],[186-187],[212-213],[425-426]
8780	dipeptidyl peptidase IV inhibitor (DPP IV inhibitor)	dipeptidyl peptidase IV inhibitor	2	FR	[56-57],[130-131]
8781	dipeptidyl peptidase IV inhibitor (DPP IV inhibitor)	dipeptidyl peptidase IV inhibitor	3	GE	[90-91],[365-366],[447-448]
8782	dipeptidyl peptidase IV inhibitor (DPP IV inhibitor)	dipeptidyl peptidase IV inhibitor	1	GF	[202-203]
8783	dipeptidyl peptidase IV inhibitor (DPP IV inhibitor)	dipeptidyl peptidase IV inhibitor	2	GG	[229-230],[414-415]
8785	dipeptidyl peptidase IV inhibitor (DPP IV inhibitor)	dipeptidyl peptidase IV inhibitor	3	GI	[95-96],[230-231],[272-273]
8786	dipeptidyl peptidase IV inhibitor (DPP IV inhibitor)	dipeptidyl peptidase IV inhibitor	3	GV	[60-61],[141-142],[323-324]
8790	dipeptidyl peptidase IV inhibitor (DPP IV inhibitor)	dipeptidyl peptidase IV inhibitor	1	HE	[53-54]
8792	dipeptidyl peptidase IV inhibitor (DPP IV inhibitor)	dipeptidyl peptidase IV inhibitor	1	HH	[451-452]

8795	dipeptidyl peptidase IV inhibitor (DPP IV inhibitor)	dipeptidyl peptidase IV inhibitor	1	HS	[340-341]
8796	dipeptidyl peptidase IV inhibitor (DPP IV inhibitor)	dipeptidyl peptidase IV inhibitor	1	HT	[66-67]
8798	dipeptidyl peptidase IV inhibitor (DPP IV inhibitor)	dipeptidyl peptidase IV inhibitor	2	HW	[144-145],[334-335]
8801	dipeptidyl peptidase IV inhibitor (DPP IV inhibitor)	dipeptidyl peptidase IV inhibitor	2	II	[135-136],[342-343]
8802	dipeptidyl peptidase IV inhibitor (DPP IV inhibitor)	dipeptidyl peptidase IV inhibitor	2	IL	[7-8],[312-313]
8804	dipeptidyl peptidase IV inhibitor (DPP IV inhibitor)	dipeptidyl peptidase IV inhibitor	1	IN	[286-287]
8805	dipeptidyl peptidase IV inhibitor (DPP IV inhibitor)	dipeptidyl peptidase IV inhibitor	1	IQ	[68-69]
8806	dipeptidyl peptidase IV inhibitor (DPP IV inhibitor)	dipeptidyl peptidase IV inhibitor	2	IR	[127-128],[242-243]
8808	dipeptidyl peptidase IV inhibitor (DPP IV inhibitor)	dipeptidyl peptidase IV inhibitor	1	KE	[283-284]
8809	dipeptidyl peptidase IV inhibitor (DPP IV inhibitor)	dipeptidyl peptidase IV inhibitor	2	KF	[175-176],[436-437]
8810	dipeptidyl peptidase IV inhibitor (DPP IV inhibitor)	dipeptidyl peptidase IV inhibitor	3	KG	[322-323],[347-348],[349-350]
8812	dipeptidyl peptidase IV inhibitor (DPP IV inhibitor)	dipeptidyl peptidase IV inhibitor	2	KI	[126-127],[434-435]
8814	dipeptidyl peptidase IV inhibitor (DPP IV inhibitor)	dipeptidyl peptidase IV inhibitor	2	KR	[263-264],[383-384]
8815	dipeptidyl peptidase IV inhibitor (DPP IV inhibitor)	dipeptidyl peptidase IV inhibitor	1	KS	[223-224]
8816	dipeptidyl peptidase IV inhibitor (DPP IV inhibitor)	dipeptidyl peptidase IV inhibitor	1	KT	[396-397]
8817	dipeptidyl peptidase IV inhibitor (DPP IV inhibitor)	dipeptidyl peptidase IV inhibitor	1	KV	[233-234]
8818	dipeptidyl peptidase IV inhibitor (DPP IV inhibitor)	dipeptidyl peptidase IV inhibitor	1	KW	[314-315]
8821	dipeptidyl peptidase IV inhibitor (DPP IV inhibitor)	dipeptidyl peptidase IV inhibitor	3	LI	[6-7],[84-85],[218-219]
8823	dipeptidyl peptidase IV inhibitor (DPP IV inhibitor)	dipeptidyl peptidase IV inhibitor	3	LN	[305-306],[308-309],[337-338]
8824	dipeptidyl peptidase IV inhibitor (DPP IV inhibitor)	dipeptidyl peptidase IV inhibitor	2	LT	[302-303],[444-445]
8825	dipeptidyl peptidase IV inhibitor (DPP IV inhibitor)	dipeptidyl peptidase IV inhibitor	3	LV	[63-64],[330-331],[373-374]
8832	dipeptidyl peptidase IV inhibitor (DPP IV inhibitor)	dipeptidyl peptidase IV inhibitor	1	ML	[372-373]
8836	dipeptidyl peptidase IV inhibitor (DPP IV inhibitor)	dipeptidyl peptidase IV inhibitor	2	MR	[280-281],[453-454]
8839	dipeptidyl peptidase IV inhibitor (DPP IV inhibitor)	dipeptidyl peptidase IV inhibitor	3	NA	[80-81],[328-329],[423-424]
8840	dipeptidyl peptidase IV inhibitor (DPP IV inhibitor)	dipeptidyl peptidase IV inhibitor	2	ND	[287-288],[398-399]
8841	dipeptidyl peptidase IV inhibitor (DPP IV inhibitor)	dipeptidyl peptidase IV inhibitor	1	NE	[148-149]
8842	dipeptidyl peptidase IV inhibitor (DPP IV inhibitor)	dipeptidyl peptidase IV inhibitor	2	NF	[357-358],[378-379]
8843	dipeptidyl peptidase IV inhibitor (DPP IV inhibitor)	dipeptidyl peptidase IV inhibitor	2	NG	[71-72],[271-272]
8844	dipeptidyl peptidase IV inhibitor (DPP IV inhibitor)	dipeptidyl peptidase IV inhibitor	1	NH	[52-53]
8845	dipeptidyl peptidase IV inhibitor (DPP IV inhibitor)	dipeptidyl peptidase IV inhibitor	3	NL	[198-199],[309-310],[336-337]
8847	dipeptidyl peptidase IV inhibitor (DPP IV inhibitor)	dipeptidyl peptidase IV inhibitor	1	NN	[438-439]
8848	dipeptidyl peptidase IV inhibitor (DPP IV inhibitor)	dipeptidyl peptidase IV inhibitor	2	NQ	[167-168],[439-440]

8849	dipeptidyl peptidase IV inhibitor (DPP IV inhibitor)	dipeptidyl peptidase IV inhibitor	3	NR	[37-38],[227-228],[360-361]
8855	dipeptidyl peptidase IV inhibitor (DPP IV inhibitor)	dipeptidyl peptidase IV inhibitor	1	PG	[99-100]
8856	dipeptidyl peptidase IV inhibitor (DPP IV inhibitor)	dipeptidyl peptidase IV inhibitor	1	PH	[333-334]
8857	dipeptidyl peptidase IV inhibitor (DPP IV inhibitor)	dipeptidyl peptidase IV inhibitor	1	PI	[311-312]
8858	dipeptidyl peptidase IV inhibitor (DPP IV inhibitor)	dipeptidyl peptidase IV inhibitor	1	PK	[182-183]
8860	dipeptidyl peptidase IV inhibitor (DPP IV inhibitor)	dipeptidyl peptidase IV inhibitor	2	PN	[51-52],[70-71]
8861	dipeptidyl peptidase IV inhibitor (DPP IV inhibitor)	dipeptidyl peptidase IV inhibitor	4	PQ	[76-77],[82-83],[108-109],[376-377]
8862	dipeptidyl peptidase IV inhibitor (DPP IV inhibitor)	dipeptidyl peptidase IV inhibitor	1	PS	[244-245]
8864	dipeptidyl peptidase IV inhibitor (DPP IV inhibitor)	dipeptidyl peptidase IV inhibitor	1	PV	[153-154]
8867	dipeptidyl peptidase IV inhibitor (DPP IV inhibitor)	dipeptidyl peptidase IV inhibitor	1	QA	[106-107]
8868	dipeptidyl peptidase IV inhibitor (DPP IV inhibitor)	dipeptidyl peptidase IV inhibitor	2	QD	[22-23],[121-122]
8869	dipeptidyl peptidase IV inhibitor (DPP IV inhibitor)	dipeptidyl peptidase IV inhibitor	1	QE	[17-18]
8870	dipeptidyl peptidase IV inhibitor (DPP IV inhibitor)	dipeptidyl peptidase IV inhibitor	1	QF	[55-56]
8871	dipeptidyl peptidase IV inhibitor (DPP IV inhibitor)	dipeptidyl peptidase IV inhibitor	3	QG	[89-90],[110-111],[114-115]
8872	dipeptidyl peptidase IV inhibitor (DPP IV inhibitor)	dipeptidyl peptidase IV inhibitor	1	QH	[191-192]
8873	dipeptidyl peptidase IV inhibitor (DPP IV inhibitor)	dipeptidyl peptidase IV inhibitor	2	QI	[26-27],[426-427]
8874	dipeptidyl peptidase IV inhibitor (DPP IV inhibitor)	dipeptidyl peptidase IV inhibitor	4	QL	[83-84],[168-169],[221-222],[317-318]
8875	dipeptidyl peptidase IV inhibitor (DPP IV inhibitor)	dipeptidyl peptidase IV inhibitor	2	QN	[166-167],[377-378]
8876	dipeptidyl peptidase IV inhibitor (DPP IV inhibitor)	dipeptidyl peptidase IV inhibitor	9	QQ	[20-21],[21-22],[109-110],[113-114],[187-188],[188-189],[189-190],[190-191],[440-441]
8877	dipeptidyl peptidase IV inhibitor (DPP IV inhibitor)	dipeptidyl peptidase IV inhibitor	3	QS	[116-117],[193-194],[248-249]
8878	dipeptidyl peptidase IV inhibitor (DPP IV inhibitor)	dipeptidyl peptidase IV inhibitor	1	QT	[441-442]
8879	dipeptidyl peptidase IV inhibitor (DPP IV inhibitor)	dipeptidyl peptidase IV inhibitor	3	QV	[213-214],[351-352],[353-354]
8881	dipeptidyl peptidase IV inhibitor (DPP IV inhibitor)	dipeptidyl peptidase IV inhibitor	1	QY	[77-78]
8882	dipeptidyl peptidase IV inhibitor (DPP IV inhibitor)	dipeptidyl peptidase IV inhibitor	4	RG	[132-133],[195-196],[228-229],[251-252]
8883	dipeptidyl peptidase IV inhibitor (DPP IV inhibitor)	dipeptidyl peptidase IV inhibitor	2	RH	[65-66],[123-124]
8885	dipeptidyl peptidase IV inhibitor (DPP IV inhibitor)	dipeptidyl peptidase IV inhibitor	2	RK	[174-175],[433-434]
8886	dipeptidyl peptidase IV inhibitor (DPP IV inhibitor)	dipeptidyl peptidase IV inhibitor	4	RL	[29-30],[217-218],[281-282],[301-302]
8888	dipeptidyl peptidase IV inhibitor (DPP IV inhibitor)	dipeptidyl peptidase IV inhibitor	1	RN	[197-198]
8889	dipeptidyl peptidase IV inhibitor (DPP IV inhibitor)	dipeptidyl peptidase IV inhibitor	3	RR	[128-129],[131-132],[264-265]
8890	dipeptidyl peptidase IV inhibitor (DPP IV inhibitor)	dipeptidyl peptidase IV inhibitor	1	RW	[265-266]
8891	dipeptidyl peptidase IV inhibitor (DPP IV inhibitor)	dipeptidyl peptidase IV inhibitor	1	SF	[394-395]

8892	dipeptidyl peptidase IV inhibitor (DPP IV inhibitor)	dipeptidyl peptidase IV inhibitor	2	SH	[339-340],[450-451]
8893	dipeptidyl peptidase IV inhibitor (DPP IV inhibitor)	dipeptidyl peptidase IV inhibitor	1	SI	[341-342]
8895	dipeptidyl peptidase IV inhibitor (DPP IV inhibitor)	dipeptidyl peptidase IV inhibitor	3	SV	[2-3],[319-320],[411-412]
8897	dipeptidyl peptidase IV inhibitor (DPP IV inhibitor)	dipeptidyl peptidase IV inhibitor	1	SY	[97-98]
8899	dipeptidyl peptidase IV inhibitor (DPP IV inhibitor)	dipeptidyl peptidase IV inhibitor	1	TE	[205-206]
8901	dipeptidyl peptidase IV inhibitor (DPP IV inhibitor)	dipeptidyl peptidase IV inhibitor	1	TG	[94-95]
8903	dipeptidyl peptidase IV inhibitor (DPP IV inhibitor)	dipeptidyl peptidase IV inhibitor	2	TI	[67-68],[276-277]
8905	dipeptidyl peptidase IV inhibitor (DPP IV inhibitor)	dipeptidyl peptidase IV inhibitor	3	TL	[158-159],[304-305],[443-444]
8906	dipeptidyl peptidase IV inhibitor (DPP IV inhibitor)	dipeptidyl peptidase IV inhibitor	1	TM	[279-280]
8907	dipeptidyl peptidase IV inhibitor (DPP IV inhibitor)	dipeptidyl peptidase IV inhibitor	1	TN	[397-398]
8910	dipeptidyl peptidase IV inhibitor (DPP IV inhibitor)	dipeptidyl peptidase IV inhibitor	3	TS	[403-404],[410-411],[445-446]
8911	dipeptidyl peptidase IV inhibitor (DPP IV inhibitor)	dipeptidyl peptidase IV inhibitor	2	TT	[303-304],[442-443]
8912	dipeptidyl peptidase IV inhibitor (DPP IV inhibitor)	dipeptidyl peptidase IV inhibitor	2	TV	[45-46],[156-157]
8914	dipeptidyl peptidase IV inhibitor (DPP IV inhibitor)	dipeptidyl peptidase IV inhibitor	1	TY	[104-105]
8915	dipeptidyl peptidase IV inhibitor (DPP IV inhibitor)	dipeptidyl peptidase IV inhibitor	2	VD	[214-215],[355-356]
8916	dipeptidyl peptidase IV inhibitor (DPP IV inhibitor)	dipeptidyl peptidase IV inhibitor	3	VE	[39-40],[46-47],[320-321]
8917	dipeptidyl peptidase IV inhibitor (DPP IV inhibitor)	dipeptidyl peptidase IV inhibitor	2	VF	[185-186],[362-363]
8918	dipeptidyl peptidase IV inhibitor (DPP IV inhibitor)	dipeptidyl peptidase IV inhibitor	1	VG	[299-300]
8920	dipeptidyl peptidase IV inhibitor (DPP IV inhibitor)	dipeptidyl peptidase IV inhibitor	1	VI	[241-242]
8921	dipeptidyl peptidase IV inhibitor (DPP IV inhibitor)	dipeptidyl peptidase IV inhibitor	3	VK	[232-233],[234-235],[382-383]
8922	dipeptidyl peptidase IV inhibitor (DPP IV inhibitor)	dipeptidyl peptidase IV inhibitor	4	VL	[324-325],[331-332],[412-413],[420-421]
8925	dipeptidyl peptidase IV inhibitor (DPP IV inhibitor)	dipeptidyl peptidase IV inhibitor	2	VQ	[88-89],[352-353]
8926	dipeptidyl peptidase IV inhibitor (DPP IV inhibitor)	dipeptidyl peptidase IV inhibitor	1	VS	[162-163]
8927	dipeptidyl peptidase IV inhibitor (DPP IV inhibitor)	dipeptidyl peptidase IV inhibitor	2	VT	[155-156],[157-158]
8930	dipeptidyl peptidase IV inhibitor (DPP IV inhibitor)	dipeptidyl peptidase IV inhibitor	1	WD	[49-50]
8934	dipeptidyl peptidase IV inhibitor (DPP IV inhibitor)	dipeptidyl peptidase IV inhibitor	1	YE	[41-42]
8936	dipeptidyl peptidase IV inhibitor (DPP IV inhibitor)	dipeptidyl peptidase IV inhibitor	1	YG	[344-345]
8939	dipeptidyl peptidase IV inhibitor (DPP IV inhibitor)	dipeptidyl peptidase IV inhibitor	1	YK	[326-327]
8942	dipeptidyl peptidase IV inhibitor (DPP IV inhibitor)	dipeptidyl peptidase IV inhibitor	1	YN	[147-148]
8943	dipeptidyl peptidase IV inhibitor (DPP IV inhibitor)	dipeptidyl peptidase IV inhibitor	1	YQ	[105-106]
8945	dipeptidyl peptidase IV inhibitor (DPP IV inhibitor)	dipeptidyl peptidase IV inhibitor	1	YS	[78-79]
8946	dipeptidyl peptidase IV inhibitor (DPP IV inhibitor)	dipeptidyl peptidase IV inhibitor	1	YV	[86-87]

9339	Dipeptidyl peptidase IV inhibitor	dipeptidyl peptidase IV inhibitor	1	LPQ	[75-77]
9477	DPP-III inhibitor	dipeptidyl peptidase III inhibitor	1	RW	[265-266]
9478	DPP-III inhibitor	dipeptidyl peptidase III inhibitor	1	LR	[239-240]
9479	DPP-III inhibitor	dipeptidyl peptidase III inhibitor	2	MR	[280-281],[453-454]
9483	DPP-III inhibitor	dipeptidyl peptidase III inhibitor	1	YK	[326-327]
9485	DPP-III inhibitor	dipeptidyl peptidase III inhibitor	3	RR	[128-129],[131-132],[264-265]
9487	DPP-III inhibitor	dipeptidyl peptidase III inhibitor	3	GE	[90-91],[365-366],[447-448]
9488	DPP-III inhibitor	dipeptidyl peptidase III inhibitor	1	GF	[202-203]
9489	DPP-III inhibitor	dipeptidyl peptidase III inhibitor	1	PR	[173-174]
9490	DPP-III inhibitor	dipeptidyl peptidase III inhibitor	3	RF	[119-120],[129-130],[389-390]
9491	DPP-III inhibitor	dipeptidyl peptidase III inhibitor	3	RV	[38-39],[240-241],[361-362]
9492	DPP-III inhibitor	dipeptidyl peptidase III inhibitor	3	DA	[31-32],[431-432],[456-457]
9493	DPP-III inhibitor	dipeptidyl peptidase III inhibitor	1	HL	[177-178]
9499	DPP-III inhibitor	dipeptidyl peptidase III inhibitor	5	LA	[13-14],[178-179],[208-209],[406-407],[421-422]
9500	DPP-III inhibitor	dipeptidyl peptidase III inhibitor	1	FA	[379-380]
9501	DPP-III inhibitor	dipeptidyl peptidase III inhibitor	2	FR	[56-57],[130-131]
9502	DPP-III inhibitor	dipeptidyl peptidase III inhibitor	1	FL	[5-6]
9504	DPP-III inhibitor	dipeptidyl peptidase III inhibitor	3	PE	[102-103],[297-298],[417-418]
9508	DPP-III inhibitor	dipeptidyl peptidase III inhibitor	1	YG	[344-345]
8247	CaMPDE inhibitor	CaMPDE inhibitor	2	IR	[127-128],[242-243]
8249	CaMPDE inhibitor	CaMPDE inhibitor	2	KF	[175-176],[436-437]
2842	Renin inhibitor	renin inhibitor	1	LR	[239-240]
8246	renin inhibitor	renin inhibitor	2	IR	[127-128],[242-243]
8248	Renin inhibitor	renin inhibitor	2	KF	[175-176],[436-437]
9430	Renin inhibitor	renin inhibitor	3	NR	[37-38],[227-228],[360-361]
9431	Renin inhibitor	renin inhibitor	1	QF	[55-56]
9432	Renin inhibitor	renin inhibitor	1	SF	[394-395]
9470	Renin inhibitor	renin inhibitor	1	LY	[325-326]

Table S9. Profile of potential biological activity of fragments of protein Ana o 3.0101.

ID	Name of peptide	Activity	Number	Sequence	Location
3380	ACE inhibitor	ACE inhibitor	2	RY	[54-55],[65-66]
3381	ACE inhibitor	ACE inhibitor	1	LY	[113-114]
3383	ACE inhibitor	ACE inhibitor	1	IY	[22-23]
3489	ACE inhibitor from sake lees	ACE inhibitor	1	RF	[48-49]
3537	ACE inhibitor	ACE inhibitor	1	PR	[121-122]
3714	ACE inhibitor	ACE inhibitor	1	LQQ	[99-101]
7558	ACE inhibitor from buckwheat	ACE inhibitor	1	VK	[56-57]
7583	ACE inhibitor	ACE inhibitor	1	AF	[10-11]
7588	ACE inhibitor	ACE inhibitor	1	RA	[24-25]
7592	ACE inhibitor	ACE inhibitor	1	FR	[49-50]
7603	ACE inhibitor	ACE inhibitor	2	GR	[34-35],[64-65]
7604	ACE inhibitor	ACE inhibitor	1	KG	[106-107]
7615	ACE inhibitor	ACE inhibitor	1	GE	[107-108]
7616	ACE inhibitor	ACE inhibitor	1	GG	[63-64]
7617	ACE inhibitor	ACE inhibitor	1	QG	[130-131]
7618	ACE inhibitor	ACE inhibitor	1	SG	[33-34]
7628	ACE inhibitor from k-CN (fr. 67-68)	ACE inhibitor	2	VR	[96-97],[110-111]
7684	ACE inhibitor from garlic	ACE inhibitor	1	SY	[137-138]
7692	ACE inhibitor	ACE inhibitor	1	KF	[3-4]
7741	ACE inhibitor	ACE inhibitor	1	RR	[85-86]
7828	ACE inhibitor	ACE inhibitor	4	EV	[28-29],[59-60],[82-83],[109-110]
7829	ACE inhibitor	ACE inhibitor	2	VE	[27-28],[29-30]
7831	ACE inhibitor	ACE inhibitor	2	LQ	[80-81],[99-100]
8193	ACE inhibitor	ACE inhibitor	1	AI	[25-26]
8951	ACE inhibitor	ACE inhibitor	1	AV	[12-13]
9060	ACE inhibitor	ACE inhibitor	1	AVL	[12-14]
9076	ACE inhibitor	ACE inhibitor	1	FQ	[134-135]
9077	ACE inhibitor	ACE inhibitor	1	YV	[55-56]
9078	ACE inhibitor	ACE inhibitor	1	YE	[114-115]

9173	ACE inhibitor	ACE inhibitor	1	RG	[62-63]
9185	ACE inhibitor	ACE inhibitor	1	YN	[66-67]
9213	ACE inhibitor	ACE inhibitor	1	LR	[73-74]
9755	ACE inhibitor	ACE inhibitor	1	PRI	[121-123]
3356	Stimulating vasoactive substance release	stimulating	3	LLL	[5-7],[6-8],[14-16]
8320	Glucose uptake stimulating peptide	stimulating	1	VL	[13-14]
8321	Glucose uptake stimulating peptide	stimulating	1	LV	[16-17]
8322	Glucose uptake stimulating peptide	stimulating	1	IV	[26-27]
8326	Glucose uptake stimulating peptide	stimulating	5	LL	[5-6],[6-7],[7-8],[14-15],[15-16]
8329	Stimulating vasoactive substance release	stimulating	3	EE	[30-31],[44-45],[108-109]
8330	Stimulating vasoactive substance release	stimulating	1	SE	[118-119]
9534	Kyotorphin	neuropeptide	1	YR	[23-24]
7872	peptide from soybean protein isolates: beta-conglycinin and glycinin	antioxidative	1	LY	[113-114]
7873	peptide from soybean protein isolates: beta-conglycinin and glycinin	antioxidative	1	IY	[22-23]
7888	antioxidative peptide	antioxidative	3	EL	[79-80],[112-113],[119-120]
4005		activating ubiquitin-mediated proteolysis	1	RA	[24-25]
9693	Alpha-glucosidase inhibitor	alpha-glucosidase inhibitor	2	VE	[27-28],[29-30]
3172	dipeptidyl peptidase IV inhibitor (DPP IV inhibitor)	dipeptidyl peptidase IV inhibitor	1	VA	[17-18]
3173	dipeptidyl peptidase IV inhibitor (DPP IV inhibitor)	dipeptidyl peptidase IV inhibitor	1	MA	[1-2]
3176	dipeptidyl peptidase IV inhibitor (DPP IV inhibitor)	dipeptidyl peptidase IV inhibitor	1	FA	[11-12]
3180	dipeptidyl peptidase IV inhibitor (DPP IV inhibitor)	dipeptidyl peptidase IV inhibitor	1	LP	[120-121]
3182	dipeptidyl peptidase IV inhibitor (DPP IV inhibitor)	dipeptidyl peptidase IV inhibitor	5	LL	[5-6],[6-7],[7-8],[14-15],[15-16]
8505	Dipeptidyl peptidase IV inhibitor (DPP IV inhibitor)	dipeptidyl peptidase IV inhibitor	1	SP	[127-128]
8526	dipeptidyl peptidase IV inhibitor (DPP IV inhibitor)	dipeptidyl peptidase IV inhibitor	1	RA	[24-25]
8531	dipeptidyl peptidase IV inhibitor (DPP IV inhibitor)	dipeptidyl peptidase IV inhibitor	1	TA	[116-117]
8555	dipeptidyl peptidase IV inhibitor (DPP IV inhibitor)	dipeptidyl peptidase IV inhibitor	1	FL	[4-5]
8560	dipeptidyl peptidase IV inhibitor (DPP IV inhibitor)	dipeptidyl peptidase IV inhibitor	1	SL	[72-73]
8594	dipeptidyl peptidase IV inhibitor (DPP IV inhibitor)	dipeptidyl peptidase IV inhibitor	2	VR	[96-97],[110-111]
8759	dipeptidyl peptidase IV inhibitor (DPP IV inhibitor)	dipeptidyl peptidase IV inhibitor	1	AF	[10-11]
8762	dipeptidyl peptidase IV inhibitor (DPP IV inhibitor)	dipeptidyl peptidase IV inhibitor	2	AS	[20-21],[117-118]
8764	dipeptidyl peptidase IV inhibitor (DPP IV inhibitor)	dipeptidyl peptidase IV inhibitor	1	AV	[12-13]
8769	dipeptidyl peptidase IV inhibitor (DPP IV inhibitor)	dipeptidyl peptidase IV inhibitor	1	DR	[84-85]

8773	dipeptidyl peptidase IV inhibitor (DPP IV inhibitor)	dipeptidyl peptidase IV inhibitor	1	ES	[71-72]
8774	dipeptidyl peptidase IV inhibitor (DPP IV inhibitor)	dipeptidyl peptidase IV inhibitor	1	ET	[115-116]
8775	dipeptidyl peptidase IV inhibitor (DPP IV inhibitor)	dipeptidyl peptidase IV inhibitor	4	EV	[28-29],[59-60],[82-83],[109-110]
8779	dipeptidyl peptidase IV inhibitor (DPP IV inhibitor)	dipeptidyl peptidase IV inhibitor	1	FQ	[134-135]
8780	dipeptidyl peptidase IV inhibitor (DPP IV inhibitor)	dipeptidyl peptidase IV inhibitor	1	FR	[49-50]
8781	dipeptidyl peptidase IV inhibitor (DPP IV inhibitor)	dipeptidyl peptidase IV inhibitor	1	GE	[107-108]
8783	dipeptidyl peptidase IV inhibitor (DPP IV inhibitor)	dipeptidyl peptidase IV inhibitor	1	GG	[63-64]
8809	dipeptidyl peptidase IV inhibitor (DPP IV inhibitor)	dipeptidyl peptidase IV inhibitor	1	KF	[3-4]
8810	dipeptidyl peptidase IV inhibitor (DPP IV inhibitor)	dipeptidyl peptidase IV inhibitor	1	KG	[106-107]
8825	dipeptidyl peptidase IV inhibitor (DPP IV inhibitor)	dipeptidyl peptidase IV inhibitor	1	LV	[16-17]
8837	dipeptidyl peptidase IV inhibitor (DPP IV inhibitor)	dipeptidyl peptidase IV inhibitor	1	MV	[95-96]
8839	dipeptidyl peptidase IV inhibitor (DPP IV inhibitor)	dipeptidyl peptidase IV inhibitor	1	NA	[19-20]
8845	dipeptidyl peptidase IV inhibitor (DPP IV inhibitor)	dipeptidyl peptidase IV inhibitor	1	NL	[91-92]
8848	dipeptidyl peptidase IV inhibitor (DPP IV inhibitor)	dipeptidyl peptidase IV inhibitor	1	NQ	[67-68]
8862	dipeptidyl peptidase IV inhibitor (DPP IV inhibitor)	dipeptidyl peptidase IV inhibitor	1	PS	[128-129]
8869	dipeptidyl peptidase IV inhibitor (DPP IV inhibitor)	dipeptidyl peptidase IV inhibitor	5	QE	[58-59],[70-71],[78-79],[81-82],[102-103]
8870	dipeptidyl peptidase IV inhibitor (DPP IV inhibitor)	dipeptidyl peptidase IV inhibitor	2	QF	[42-43],[133-134]
8871	dipeptidyl peptidase IV inhibitor (DPP IV inhibitor)	dipeptidyl peptidase IV inhibitor	1	QG	[130-131]
8873	dipeptidyl peptidase IV inhibitor (DPP IV inhibitor)	dipeptidyl peptidase IV inhibitor	1	QI	[104-105]
8874	dipeptidyl peptidase IV inhibitor (DPP IV inhibitor)	dipeptidyl peptidase IV inhibitor	1	QL	[98-99]
8875	dipeptidyl peptidase IV inhibitor (DPP IV inhibitor)	dipeptidyl peptidase IV inhibitor	1	QN	[90-91]
8876	dipeptidyl peptidase IV inhibitor (DPP IV inhibitor)	dipeptidyl peptidase IV inhibitor	3	QQ	[46-47],[100-101],[101-102]
8877	dipeptidyl peptidase IV inhibitor (DPP IV inhibitor)	dipeptidyl peptidase IV inhibitor	2	QS	[37-38],[135-136]
8882	dipeptidyl peptidase IV inhibitor (DPP IV inhibitor)	dipeptidyl peptidase IV inhibitor	1	RG	[62-63]
8884	dipeptidyl peptidase IV inhibitor (DPP IV inhibitor)	dipeptidyl peptidase IV inhibitor	1	RI	[122-123]
8888	dipeptidyl peptidase IV inhibitor (DPP IV inhibitor)	dipeptidyl peptidase IV inhibitor	1	RN	[50-51]
8889	dipeptidyl peptidase IV inhibitor (DPP IV inhibitor)	dipeptidyl peptidase IV inhibitor	1	RR	[85-86]
8893	dipeptidyl peptidase IV inhibitor (DPP IV inhibitor)	dipeptidyl peptidase IV inhibitor	2	SI	[21-22],[125-126]
8897	dipeptidyl peptidase IV inhibitor (DPP IV inhibitor)	dipeptidyl peptidase IV inhibitor	1	SY	[137-138]
8915	dipeptidyl peptidase IV inhibitor (DPP IV inhibitor)	dipeptidyl peptidase IV inhibitor	1	VD	[83-84]
8916	dipeptidyl peptidase IV inhibitor (DPP IV inhibitor)	dipeptidyl peptidase IV inhibitor	2	VE	[27-28],[29-30]
8921	dipeptidyl peptidase IV inhibitor (DPP IV inhibitor)	dipeptidyl peptidase IV inhibitor	1	VK	[56-57]

8922	dipeptidyl peptidase IV inhibitor (DPP IV inhibitor)	dipeptidyl peptidase IV inhibitor	1	VL	[13-14]
8925	dipeptidyl peptidase IV inhibitor (DPP IV inhibitor)	dipeptidyl peptidase IV inhibitor	1	VQ	[60-61]
8934	dipeptidyl peptidase IV inhibitor (DPP IV inhibitor)	dipeptidyl peptidase IV inhibitor	1	YE	[114-115]
8942	dipeptidyl peptidase IV inhibitor (DPP IV inhibitor)	dipeptidyl peptidase IV inhibitor	1	YN	[66-67]
8944	dipeptidyl peptidase IV inhibitor (DPP IV inhibitor)	dipeptidyl peptidase IV inhibitor	1	YR	[23-24]
8946	dipeptidyl peptidase IV inhibitor (DPP IV inhibitor)	dipeptidyl peptidase IV inhibitor	1	YV	[55-56]
9478	DPP-III inhibitor	dipeptidyl peptidase III inhibitor	1	LR	[73-74]
9484	DPP-III inhibitor	dipeptidyl peptidase III inhibitor	1	YR	[23-24]
9485	DPP-III inhibitor	dipeptidyl peptidase III inhibitor	1	RR	[85-86]
9487	DPP-III inhibitor	dipeptidyl peptidase III inhibitor	1	GE	[107-108]
9489	DPP-III inhibitor	dipeptidyl peptidase III inhibitor	1	PR	[121-122]
9490	DPP-III inhibitor	dipeptidyl peptidase III inhibitor	1	RF	[48-49]
9500	DPP-III inhibitor	dipeptidyl peptidase III inhibitor	1	FA	[11-12]
9501	DPP-III inhibitor	dipeptidyl peptidase III inhibitor	1	FR	[49-50]
9502	DPP-III inhibitor	dipeptidyl peptidase III inhibitor	1	FL	[4-5]
8249	CaMPDE inhibitor	CaMPDE inhibitor	1	KF	[3-4]
2842	Renin inhibitor	renin inhibitor	1	LR	[73-74]
8248	Renin inhibitor	renin inhibitor	1	KF	[3-4]
9431	Renin inhibitor	renin inhibitor	2	QF	[42-43],[133-134]
9470	Renin inhibitor	renin inhibitor	1	LY	[113-114]

Table S10. Profile of potential biological activity of fragments of protein Car i 1.0101.

ID	Name of peptide	Activity	Number	Sequence	Location
3258	beta-lactokinin	ACE inhibitor	2	IR	[110-111],[138-139]
3515	ACE inhibitor	ACE inhibitor	1	GGY	[67-69]
3518	ACE inhibitor	ACE inhibitor	1	VAA	[4-6]
3532	ACE inhibitor	ACE inhibitor	1	GY	[68-69]
3537	ACE inhibitor	ACE inhibitor	1	PR	[36-37]
3551	ACE inhibitor (from bovine beta-Lg)	ACE inhibitor	1	LF	[12-13]
7542	ACE inhibitor	ACE inhibitor	1	DLP	[124-126]
7583	ACE inhibitor	ACE inhibitor	1	AF	[19-20]
7590	ACE inhibitor	ACE inhibitor	3	AA	[5-6],[17-18],[18-19]
7592	ACE inhibitor	ACE inhibitor	2	FR	[20-21],[78-79]
7596	ACE inhibitor	ACE inhibitor	2	GI	[109-110],[130-131]
7599	ACE inhibitor	ACE inhibitor	1	GL	[96-97]
7615	ACE inhibitor	ACE inhibitor	2	GE	[40-41],[112-113]
7616	ACE inhibitor	ACE inhibitor	1	GG	[67-68]
7618	ACE inhibitor	ACE inhibitor	1	SG	[66-67]
7622	ACE inhibitor	ACE inhibitor	2	EG	[95-96],[108-109]
7628	ACE inhibitor from k-CN (fr. 67-68)	ACE inhibitor	1	VR	[101-102]
7644	ACE inhibitor from porcine myosin (306-308)	ACE inhibitor	1	ITT	[24-26]
7741	ACE inhibitor	ACE inhibitor	3	RR	[37-38],[38-39],[139-140]
7742	ACE inhibitor	ACE inhibitor	1	AR	[2-3]
7807	ACE inhibitor from caprine b-Lg	ACE inhibitor	1	LLF	[11-13]
7826	ACE inhibitor	ACE inhibitor	2	EI	[28-29],[137-138]
7832	ACE inhibitor	ACE inhibitor	1	LN	[53-54]
7839	ACE inhibitor	ACE inhibitor	3	ME	[27-28],[88-89],[115-116]
7841	ACE inhibitor	ACE inhibitor	1	KE	[127-128]
8951	ACE inhibitor	ACE inhibitor	1	AV	[100-101]
9072	ACE inhibitor	ACE inhibitor	1	DY	[58-59]
9173	ACE inhibitor	ACE inhibitor	2	RG	[39-40],[111-112]
9213	ACE inhibitor	ACE inhibitor	2	LR	[60-61],[97-98]

9309	ACE inhibitor	ACE inhibitor	1	RRR	[37-39]
9730	ACE inhibitor	ACE inhibitor	1	NPR	[35-37]
8321	Glucose uptake stimulating peptide	stimulating	1	LV	[8-9]
8326	Glucose uptake stimulating peptide	stimulating	2	LL	[7-8],[11-12]
8329	Stimulating vasoactive substance release	stimulating	4	EE	[89-90],[107-108],[113-114],[116-117]
8310	Anxiolytic peptide	neuropeptide	2	YL	[52-53],[59-60]
2749	peptide regulating ion flow	regulating	1	DY	[58-59]
8215	Antioxidative peptide	antioxidative	2	IR	[110-111],[138-139]
9357	Antioxidative peptide	antioxidative	1	CQC	[92-94]
9368	Antioxidative peptide	antioxidative	1	EQC	[90-92]
3172	dipeptidyl peptidase IV inhibitor (DPP IV inhibitor)	dipeptidyl peptidase IV inhibitor	3	VA	[4-5],[9-10],[14-15]
3173	dipeptidyl peptidase IV inhibitor (DPP IV inhibitor)	dipeptidyl peptidase IV inhibitor	1	MA	[1-2]
3180	dipeptidyl peptidase IV inhibitor (DPP IV inhibitor)	dipeptidyl peptidase IV inhibitor	1	LP	[125-126]
3182	dipeptidyl peptidase IV inhibitor (DPP IV inhibitor)	dipeptidyl peptidase IV inhibitor	2	LL	[7-8],[11-12]
8530	dipeptidyl peptidase IV inhibitor (DPP IV inhibitor)	dipeptidyl peptidase IV inhibitor	1	NP	[35-36]
8559	dipeptidyl peptidase IV inhibitor (DPP IV inhibitor)	dipeptidyl peptidase IV inhibitor	2	AL	[6-7],[10-11]
8561	dipeptidyl peptidase IV inhibitor (DPP IV inhibitor)	dipeptidyl peptidase IV inhibitor	1	GL	[96-97]
8594	dipeptidyl peptidase IV inhibitor (DPP IV inhibitor)	dipeptidyl peptidase IV inhibitor	1	VR	[101-102]
8637	dipeptidyl peptidase IV inhibitor (DPP IV inhibitor)	dipeptidyl peptidase IV inhibitor	3	AA	[5-6],[17-18],[18-19]
8692	dipeptidyl peptidase IV inhibitor (DPP IV inhibitor)	dipeptidyl peptidase IV inhibitor	1	WF	[142-143]
8759	dipeptidyl peptidase IV inhibitor (DPP IV inhibitor)	dipeptidyl peptidase IV inhibitor	1	AF	[19-20]
8762	dipeptidyl peptidase IV inhibitor (DPP IV inhibitor)	dipeptidyl peptidase IV inhibitor	1	AS	[122-123]
8764	dipeptidyl peptidase IV inhibitor (DPP IV inhibitor)	dipeptidyl peptidase IV inhibitor	1	AV	[100-101]
8766	dipeptidyl peptidase IV inhibitor (DPP IV inhibitor)	dipeptidyl peptidase IV inhibitor	2	DN	[34-35],[72-73]
8770	dipeptidyl peptidase IV inhibitor (DPP IV inhibitor)	dipeptidyl peptidase IV inhibitor	2	EG	[95-96],[108-109]
8772	dipeptidyl peptidase IV inhibitor (DPP IV inhibitor)	dipeptidyl peptidase IV inhibitor	2	EI	[28-29],[137-138]
8773	dipeptidyl peptidase IV inhibitor (DPP IV inhibitor)	dipeptidyl peptidase IV inhibitor	1	ES	[41-42]
8780	dipeptidyl peptidase IV inhibitor (DPP IV inhibitor)	dipeptidyl peptidase IV inhibitor	2	FR	[20-21],[78-79]
8781	dipeptidyl peptidase IV inhibitor (DPP IV inhibitor)	dipeptidyl peptidase IV inhibitor	2	GE	[40-41],[112-113]
8783	dipeptidyl peptidase IV inhibitor (DPP IV inhibitor)	dipeptidyl peptidase IV inhibitor	1	GG	[67-68]
8785	dipeptidyl peptidase IV inhibitor (DPP IV inhibitor)	dipeptidyl peptidase IV inhibitor	2	GI	[109-110],[130-131]
8788	dipeptidyl peptidase IV inhibitor (DPP IV inhibitor)	dipeptidyl peptidase IV inhibitor	1	GY	[68-69]

8791	dipeptidyl peptidase IV inhibitor (DPP IV inhibitor)	dipeptidyl peptidase IV inhibitor	1	HF	[77-78]
8805	dipeptidyl peptidase IV inhibitor (DPP IV inhibitor)	dipeptidyl peptidase IV inhibitor	1	IQ	[47-48]
8806	dipeptidyl peptidase IV inhibitor (DPP IV inhibitor)	dipeptidyl peptidase IV inhibitor	2	IR	[110-111],[138-139]
8808	dipeptidyl peptidase IV inhibitor (DPP IV inhibitor)	dipeptidyl peptidase IV inhibitor	1	KE	[127-128]
8823	dipeptidyl peptidase IV inhibitor (DPP IV inhibitor)	dipeptidyl peptidase IV inhibitor	1	LN	[53-54]
8825	dipeptidyl peptidase IV inhibitor (DPP IV inhibitor)	dipeptidyl peptidase IV inhibitor	1	LV	[8-9]
8826	dipeptidyl peptidase IV inhibitor (DPP IV inhibitor)	dipeptidyl peptidase IV inhibitor	3	ME	[27-28],[88-89],[115-116]
8837	dipeptidyl peptidase IV inhibitor (DPP IV inhibitor)	dipeptidyl peptidase IV inhibitor	1	MV	[118-119]
8839	dipeptidyl peptidase IV inhibitor (DPP IV inhibitor)	dipeptidyl peptidase IV inhibitor	1	NA	[16-17]
8848	dipeptidyl peptidase IV inhibitor (DPP IV inhibitor)	dipeptidyl peptidase IV inhibitor	1	NQ	[73-74]
8849	dipeptidyl peptidase IV inhibitor (DPP IV inhibitor)	dipeptidyl peptidase IV inhibitor	1	NR	[54-55]
8858	dipeptidyl peptidase IV inhibitor (DPP IV inhibitor)	dipeptidyl peptidase IV inhibitor	1	PK	[126-127]
8867	dipeptidyl peptidase IV inhibitor (DPP IV inhibitor)	dipeptidyl peptidase IV inhibitor	1	QA	[99-100]
8868	dipeptidyl peptidase IV inhibitor (DPP IV inhibitor)	dipeptidyl peptidase IV inhibitor	1	QD	[57-58]
8869	dipeptidyl peptidase IV inhibitor (DPP IV inhibitor)	dipeptidyl peptidase IV inhibitor	1	QE	[106-107]
8872	dipeptidyl peptidase IV inhibitor (DPP IV inhibitor)	dipeptidyl peptidase IV inhibitor	1	QH	[76-77]
8873	dipeptidyl peptidase IV inhibitor (DPP IV inhibitor)	dipeptidyl peptidase IV inhibitor	1	QI	[46-47]
8874	dipeptidyl peptidase IV inhibitor (DPP IV inhibitor)	dipeptidyl peptidase IV inhibitor	1	QL	[84-85]
8876	dipeptidyl peptidase IV inhibitor (DPP IV inhibitor)	dipeptidyl peptidase IV inhibitor	6	QQ	[50-51],[62-63],[83-84],[103-104],[104-105],[105-106]
8881	dipeptidyl peptidase IV inhibitor (DPP IV inhibitor)	dipeptidyl peptidase IV inhibitor	1	QY	[51-52]
8882	dipeptidyl peptidase IV inhibitor (DPP IV inhibitor)	dipeptidyl peptidase IV inhibitor	2	RG	[39-40],[111-112]
8889	dipeptidyl peptidase IV inhibitor (DPP IV inhibitor)	dipeptidyl peptidase IV inhibitor	3	RR	[37-38],[38-39],[139-140]
8896	dipeptidyl peptidase IV inhibitor (DPP IV inhibitor)	dipeptidyl peptidase IV inhibitor	1	SW	[141-142]
8903	dipeptidyl peptidase IV inhibitor (DPP IV inhibitor)	dipeptidyl peptidase IV inhibitor	1	TI	[23-24]
8906	dipeptidyl peptidase IV inhibitor (DPP IV inhibitor)	dipeptidyl peptidase IV inhibitor	1	TM	[26-27]
8911	dipeptidyl peptidase IV inhibitor (DPP IV inhibitor)	dipeptidyl peptidase IV inhibitor	2	TT	[22-23],[25-26]
8925	dipeptidyl peptidase IV inhibitor (DPP IV inhibitor)	dipeptidyl peptidase IV inhibitor	1	VQ	[119-120]
8933	dipeptidyl peptidase IV inhibitor (DPP IV inhibitor)	dipeptidyl peptidase IV inhibitor	1	YD	[69-70]
8940	dipeptidyl peptidase IV inhibitor (DPP IV inhibitor)	dipeptidyl peptidase IV inhibitor	2	YL	[52-53],[59-60]
9478	DPP-III inhibitor	dipeptidyl peptidase III inhibitor	2	LR	[60-61],[97-98]
9482	DPP-III inhibitor	dipeptidyl peptidase III inhibitor	2	YL	[52-53],[59-60]
9485	DPP-III inhibitor	dipeptidyl peptidase III inhibitor	3	RR	[37-38],[38-39],[139-140]

9487	DPP-III inhibitor	dipeptidyl peptidase III inhibitor	2	GE	[40-41],[112-113]
9489	DPP-III inhibitor	dipeptidyl peptidase III inhibitor	1	PR	[36-37]
9491	DPP-III inhibitor	dipeptidyl peptidase III inhibitor	1	RV	[3-4]
9495	DPP-III inhibitor	dipeptidyl peptidase III inhibitor	1	HF	[77-78]
9501	DPP-III inhibitor	dipeptidyl peptidase III inhibitor	2	FR	[20-21],[78-79]
8247	CaMPDE inhibitor	CaMPDE inhibitor	2	IR	[110-111],[138-139]
2842	Renin inhibitor	renin inhibitor	2	LR	[60-61],[97-98]
8246	renin inhibitor	renin inhibitor	2	IR	[110-111],[138-139]
9430	Renin inhibitor	renin inhibitor	1	NR	[54-55]

Table S11. Profile of potential biological activity of fragments of protein Car i 2.0101.

ID	Name of peptide	Activity	Number	Sequence	Location
3460	Prolyl endopeptidase inhibitor	antiamnestic	1	PG	[499-500]
3461	Prolyl endopeptidase inhibitor	antiamnestic	2	GP	[356-357],[580-581]
2653	ACE inhibitor	ACE inhibitor	1	VLP	[429-431]
3257	beta-lactokinin	ACE inhibitor	5	RL	[241-242],[486-487],[535-536],[685-686],[711-712]
3258	beta-lactokinin	ACE inhibitor	2	IR	[468-469],[551-552]
3380	ACE inhibitor	ACE inhibitor	1	RY	[271-272]
3384	ACE inhibitor	ACE inhibitor	1	VF	[519-520]
3489	ACE inhibitor from sake lees	ACE inhibitor	4	RF	[182-183],[402-403],[538-539],[647-648]
3492	ACE inhibitor from sake	ACE inhibitor	1	VY	[639-640]
3494	ACE inhibitor from sake	ACE inhibitor	1	HY	[630-631]
3507	ACE inhibitor (beta-LG fr. 78-80)	ACE inhibitor	1	IPA	[694-696]
3518	ACE inhibitor	ACE inhibitor	1	VAA	[526-528]
3537	ACE inhibitor	ACE inhibitor	11	PR	[92-93],[95-96],[111-112],[148-149],[151-152],[207-208],[210-211],[265-266],[362-363],[532-533],[749-750]
3542	ACE inhibitor	ACE inhibitor	1	LQP	[493-495]
3544	ACE inhibitor	ACE inhibitor	1	LNP	[36-38]
3547	ACE inhibitor	ACE inhibitor	1	IRA	[551-553]
3551	ACE inhibitor (from bovine beta-Lg)	ACE inhibitor	3	LF	[9-10],[11-12],[16-17]
3553	ACE inhibitor	ACE inhibitor	1	YG	[464-465]
7512	ACE inhibitor from Alaskan pollack skin	ACE inhibitor	2	GP	[356-357],[580-581]
7513	ACE inhibitor from Alaskan pollack skin	ACE inhibitor	2	PL	[8-9],[781-782]
7558	ACE inhibitor from buckwheat	ACE inhibitor	2	VK	[397-398],[490-491]
7562	ACE inhibitor from soy hydrolysate	ACE inhibitor	1	IA	[700-701]
7580	ACE inhibitor	ACE inhibitor	1	RW	[46-47]
7581	ACE inhibitor	ACE inhibitor	2	IP	[7-8],[694-695]
7582	ACE inhibitor	ACE inhibitor	1	RP	[571-572]
7585	ACE inhibitor	ACE inhibitor	5	LA	[18-19],[25-26],[686-687],[727-728],[782-783]
7586	ACE inhibitor	ACE inhibitor	1	KR	[622-623]
7587	ACE inhibitor	ACE inhibitor	3	VP	[470-471],[628-629],[766-767]
7588	ACE inhibitor	ACE inhibitor	3	RA	[446-447],[552-553],[559-560]

7589	ACE inhibitor	ACE inhibitor	2	YA	[506-507],[618-619]
7590	ACE inhibitor	ACE inhibitor	2	AA	[507-508],[527-528]
7591	ACE inhibitor	ACE inhibitor	2	GF	[714-715],[790-791]
7592	ACE inhibitor	ACE inhibitor	1	FR	[502-503]
7593	ACE inhibitor	ACE inhibitor	2	IF	[691-692],[756-757]
7594	ACE inhibitor	ACE inhibitor	1	VG	[713-714]
7596	ACE inhibitor	ACE inhibitor	2	GI	[412-413],[716-717]
7598	ACE inhibitor	ACE inhibitor	3	GA	[473-474],[509-510],[624-625]
7599	ACE inhibitor	ACE inhibitor	1	GL	[385-386]
7600	ACE inhibitor	ACE inhibitor	6	AG	[472-473],[508-509],[568-569],[696-697],[728-729],[789-790]
7601	ACE inhibitor	ACE inhibitor	1	GH	[697-698]
7603	ACE inhibitor	ACE inhibitor	11	GR	[139-140],[198-199],[257-258],[309-310],[352-353],[368-369],[445-446],[574-575],[646-647],[665-666],[777-778]
7604	ACE inhibitor	ACE inhibitor	1	KG	[664-665]
7605	ACE inhibitor	ACE inhibitor	2	FG	[595-596],[715-716]
7606	ACE inhibitor	ACE inhibitor	1	DA	[435-436]
7608	ACE inhibitor	ACE inhibitor	1	GV	[548-549]
7610	ACE inhibitor	ACE inhibitor	12	GQ	[62-63],[166-167],[168-169],[225-226],[227-228],[285-286],[500-501],[569-570],[596-597],[677-678],[729-730],[775-776]
7611	ACE inhibitor	ACE inhibitor	1	GK	[719-720]
7612	ACE inhibitor	ACE inhibitor	1	GT	[644-645]
7613	ACE inhibitor	ACE inhibitor	2	WG	[355-356],[573-574]
7615	ACE inhibitor	ACE inhibitor	2	GE	[393-394],[395-396]
7616	ACE inhibitor	ACE inhibitor	1	GG	[579-580]
7617	ACE inhibitor	ACE inhibitor	7	QG	[165-166],[167-168],[224-225],[226-227],[351-352],[384-385],[776-777]
7618	ACE inhibitor	ACE inhibitor	2	SG	[392-393],[578-579]
7620	ACE inhibitor	ACE inhibitor	2	GD	[465-466],[689-690]
7621	ACE inhibitor	ACE inhibitor	2	TG	[645-646],[676-677]
7622	ACE inhibitor	ACE inhibitor	3	EG	[394-395],[547-548],[643-644]
7623	ACE inhibitor	ACE inhibitor	3	EA	[422-423],[600-601],[740-741]
7624	ACE inhibitor	ACE inhibitor	1	NG	[718-719]
7625	ACE inhibitor	ACE inhibitor	1	PG	[499-500]
7638	ACE inhibitor from tuna muscles	ACE inhibitor	1	LTF	[449-451]

7680	ACE inhibitor from pea vicilin	ACE inhibitor	1	QK	[680-681]
7682	ACE inhibitor from garlic	ACE inhibitor	2	NY	[415-416],[617-618]
7683	ACE inhibitor from garlic	ACE inhibitor	1	NF	[725-726]
7684	ACE inhibitor from garlic	ACE inhibitor	4	SY	[515-516],[590-591],[660-661],[763-764]
7685	ACE inhibitor from garlic	ACE inhibitor	2	SF	[459-460],[745-746]
7691	ACE inhibitor from wakame	ACE inhibitor	1	KY	[398-399]
7693	ACE inhibitor from wakame	ACE inhibitor	2	KL	[491-492],[557-558]
7697	ACE inhibitor from wakame	ACE inhibitor	2	YK	[347-348],[663-664]
7741	ACE inhibitor	ACE inhibitor	21	RR	[71-72],[119-120],[133-134],[136-137],[137-138],[163-164],[188-189],[195-196],[196-197],[222-223],[254-255],[255-256],[283-284],[292-293],[293-294],[324-325],[363-364],[456-457],[575-576],[666-667],[773-774]
7742	ACE inhibitor	ACE inhibitor	2	AR	[684-685],[687-688]
7743	ACE inhibitor	ACE inhibitor	2	KA	[4-5],[634-635]
7752	ACE inhibitor from shark meat hydrolysate	ACE inhibitor	4	EY	[463-464],[476-477],[504-505],[662-663]
7826	ACE inhibitor	ACE inhibitor	4	EI	[88-89],[620-621],[752-753],[755-756]
7827	ACE inhibitor	ACE inhibitor	2	IE	[413-414],[753-754]
7828	ACE inhibitor	ACE inhibitor	1	EV	[396-397]
7829	ACE inhibitor	ACE inhibitor	2	VE	[642-643],[761-762]
7830	ACE inhibitor	ACE inhibitor	5	TE	[29-30],[404-405],[407-408],[475-476],[513-514]
7831	ACE inhibitor	ACE inhibitor	2	LQ	[493-494],[609-610]
7832	ACE inhibitor	ACE inhibitor	2	LN	[36-37],[529-530]
7834	ACE inhibitor	ACE inhibitor	1	TQ	[328-329]
7837	ACE inhibitor	ACE inhibitor	2	PQ	[115-116],[315-316]
7838	ACE inhibitor	ACE inhibitor	1	EW	[354-355]
7839	ACE inhibitor	ACE inhibitor	1	ME	[768-769]
7840	ACE inhibitor	ACE inhibitor	2	EK	[107-108],[556-557]
7841	ACE inhibitor	ACE inhibitor	3	KE	[145-146],[348-349],[742-743]
7842	ACE inhibitor	ACE inhibitor	2	HP	[698-699],[780-781]
7843	ACE inhibitor	ACE inhibitor	3	PH	[38-39],[629-630],[653-654]
7844	ACE inhibitor	ACE inhibitor	1	HK	[433-434]
8185	ACE inhibitor	ACE inhibitor	2	TF	[427-428],[450-451]
8193	ACE inhibitor	ACE inhibitor	1	AI	[701-702]
8513	ACE inhibitor from soya milk	ACE inhibitor	1	FVP	[765-767]

9031	ACE inhibitor	ACE inhibitor	1	LEE	[242-244]
9068	ACE inhibitor	ACE inhibitor	1	VTR	[442-444]
9073	ACE inhibitor	ACE inhibitor	1	TP	[531-532]
9074	ACE inhibitor	ACE inhibitor	1	DF	[787-788]
9076	ACE inhibitor	ACE inhibitor	3	FQ	[49-50],[125-126],[679-680]
9077	ACE inhibitor	ACE inhibitor	2	YV	[477-478],[640-641]
9078	ACE inhibitor	ACE inhibitor	3	YE	[99-100],[272-273],[661-662]
9079	ACE inhibitor	ACE inhibitor	3	IL	[420-421],[524-525],[785-786]
9085	ACE inhibitor	ACE inhibitor	1	MM	[626-627]
9087	ACE inhibitor	ACE inhibitor	2	YH	[319-320],[432-433]
9173	ACE inhibitor	ACE inhibitor	12	RG	[61-62],[138-139],[197-198],[256-257],[284-285],[308-309],[367-368],[411-412],[444-445],[623-624],[688-689],[774-775]
9183	ACE inhibitor	ACE inhibitor	1	GTG	[644-646]
9184	ACE inhibitor	ACE inhibitor	2	ST	[512-513],[675-676]
9185	ACE inhibitor	ACE inhibitor	1	YN	[631-632]
9213	ACE inhibitor	ACE inhibitor	6	LR	[77-78],[386-387],[410-411],[517-518],[558-559],[710-711]
9309	ACE inhibitor	ACE inhibitor	4	RRR	[136-138],[195-197],[254-256],[292-294]
9566	ACE inhibitor	ACE inhibitor	2	QP	[114-115],[494-495]
9730	ACE inhibitor	ACE inhibitor	3	NPR	[147-149],[206-208],[264-266]
9743	ACE inhibitor	ACE inhibitor	4	EQR	[186-188],[245-247],[269-271],[544-546]
9754	ACE inhibitor	ACE inhibitor	1	NLR	[709-711]
3283	Antithrombotic peptide	antithrombotic	2	GP	[356-357],[580-581]
3285	Antithrombotic peptide	antithrombotic	1	PG	[499-500]
3354	Antithrombotic peptide	antithrombotic	1	DEE	[203-205]
9660	Antithrombotic peptide	antithrombotic	1	RGD	[688-690]
2882	Immunostimulating peptide	immunomodulating	1	YG	[464-465]
3351	Stimulating vasoactive substance release	stimulating	5	EEE	[184-186],[243-245],[370-372],[670-672],[671-673]
8320	Glucose uptake stimulating peptide	stimulating	2	VL	[429-430],[614-615]
8321	Glucose uptake stimulating peptide	stimulating	4	LV	[20-21],[525-526],[615-616],[712-713]
8322	Glucose uptake stimulating peptide	stimulating	2	IV	[89-90],[440-441]
8323	Glucose uptake stimulating peptide	stimulating	3	IL	[420-421],[524-525],[785-786]
8325	Glucose uptake stimulating peptide	stimulating	2	II	[550-551],[733-734]

8326	Glucose uptake stimulating peptide	stimulating	2	LL	[409-410],[492-493]
8329	Stimulating vasoactive substance release	stimulating	22	EE	[59-60],[74-75],[87-88],[159-160],[180-181],[184-185],[185-186],[191-192],[204-205],[239-240],[243-244],[244-245],[250-251],[302-303],[370-371],[371-372],[604-605],[670-671],[671-672],[672-673],[751-752],[754-755]
2890	neuropeptide	neuropeptide	12	GQ	[62-63],[166-167],[168-169],[225-226],[227-228],[285-286],[500-501],[569-570],[596-597],[677-678],[729-730],[775-776]
8310	Anxiolytic peptide	neuropeptide	2	YL	[399-400],[516-517]
9534	Kyotorphin	neuropeptide	3	YR	[155-156],[214-215],[416-417]
2753	peptide regulating the stomach mucosal membrane activity	regulating	2	GP	[356-357],[580-581]
2754	peptide regulating the stomach mucosal membrane activity	regulating	1	PG	[499-500]
8318	Dvl protein binding	anticancer	1	VVV	[637-639]
3300		antioxidative	1	PHH	[38-40]
3307		antioxidative	1	PYY	[378-380]
3319		antioxidative	1	HH	[39-40]
7888	antioxidative peptide	antioxidative	3	EL	[35-36],[408-409],[743-744]
7927	synthetic peptide	antioxidative	1	EYY	[504-506]
7928	synthetic peptide	antioxidative	1	YEY	[661-663]
7939	synthetic peptide	antioxidative	1	YYA	[505-507]
7943	synthetic peptide	antioxidative	1	YYF	[379-381]
8023	synthetic peptide	antioxidative	1	PHD	[653-655]
8038	synthetic peptide	antioxidative	1	PHY	[629-631]
8042	synthetic peptide	antioxidative	1	PWG	[572-574]
8056	synthetic peptide	antioxidative	1	RHE	[389-391]
8063	synthetic peptide	antioxidative	1	RHN	[375-377]
8072	synthetic peptide	antioxidative	1	RWE	[46-48]
8103	peptide derived from dried bonito	antioxidative	1	VKL	[490-492]
8130	peptide derived from dried bonito	antioxidative	1	EAK	[740-742]
8134	peptide derived from dried bonito	antioxidative	1	KD	[434-435]
8190	peptide from buckwheat	antioxidative	1	PW	[572-573]
8214	Antioxidative peptide	antioxidative	1	RW	[46-47]
8215	Antioxidative peptide	antioxidative	2	IR	[468-469],[551-552]
8217	Antioxidative peptide	antioxidative	1	LK	[584-585]

8224	antioxidative peptide	antioxidative	1	VY	[639-640]
8484	Antioxidant peptide from as1-CN (98-100)	antioxidative	1	LLR	[409-411]
9082	Antioxidative peptide	antioxidative	2	WG	[355-356],[573-574]
9086	Antioxidative peptide	antioxidative	1	MM	[626-627]
9361	Antioxidative peptide	antioxidative	1	VYV	[639-641]
9363	Antioxidative peptide	antioxidative	1	NEN	[707-709]
9366	Antioxidative peptide	antioxidative	1	KYL	[398-400]
9368	Antioxidative peptide	antioxidative	2	EQC	[100-102],[273-275]
3164	laminin-like peptide	embryotoxic	1	RGD	[688-690]
4005		activating ubiquitin-mediated proteolysis	3	RA	[446-447],[552-553],[559-560]
4006	Ubiqitin-mediated proteolysis activating peptide	activating ubiquitin-mediated proteolysis	5	LA	[18-19],[25-26],[686-687],[727-728],[782-783]
9580	Hypolipidemic peptide	hypolipidemic	1	EF	[48-49]
9650	Alpha-glucosidase inhibitor	alpha-glucosidase inhibitor	3	EA	[422-423],[600-601],[740-741]
9693	Alpha-glucosidase inhibitor	alpha-glucosidase inhibitor	2	VE	[642-643],[761-762]
9694	Alpha-glucosidase inhibitor	alpha-glucosidase inhibitor	4	PE	[42-43],[268-269],[333-334],[603-604]
3169	dipeptidyl peptidase IV inhibitor (DPP IV inhibitor)	dipeptidyl peptidase IV inhibitor	2	GP	[356-357],[580-581]
3171	dipeptidyl peptidase IV inhibitor (DPP IV inhibitor)	dipeptidyl peptidase IV inhibitor	1	MP	[748-749]
3172	dipeptidyl peptidase IV inhibitor (DPP IV inhibitor)	dipeptidyl peptidase IV inhibitor	1	VA	[526-527]
3173	dipeptidyl peptidase IV inhibitor (DPP IV inhibitor)	dipeptidyl peptidase IV inhibitor	1	MA	[650-651]
3174	dipeptidyl peptidase IV inhibitor (DPP IV inhibitor)	dipeptidyl peptidase IV inhibitor	2	KA	[4-5],[634-635]
3175	dipeptidyl peptidase IV inhibitor (DPP IV inhibitor)	dipeptidyl peptidase IV inhibitor	5	LA	[18-19],[25-26],[686-687],[727-728],[782-783]
3176	dipeptidyl peptidase IV inhibitor (DPP IV inhibitor)	dipeptidyl peptidase IV inhibitor	1	FA	[788-789]
3179	dipeptidyl peptidase IV inhibitor (DPP IV inhibitor)	dipeptidyl peptidase IV inhibitor	2	PA	[471-472],[695-696]
3180	dipeptidyl peptidase IV inhibitor (DPP IV inhibitor)	dipeptidyl peptidase IV inhibitor	1	LP	[430-431]

3181	dipeptidyl peptidase IV inhibitor (DPP IV inhibitor)	dipeptidyl peptidase IV inhibitor	3	VP	[470-471],[628-629],[766-767]
3182	dipeptidyl peptidase IV inhibitor (DPP IV inhibitor)	dipeptidyl peptidase IV inhibitor	2	LL	[409-410],[492-493]
3183	dipeptidyl peptidase IV inhibitor (DPP IV inhibitor)	dipeptidyl peptidase IV inhibitor	5	VV	[418-419],[441-442],[637-638],[638-639],[641-642]
3184	dipeptidyl peptidase IV inhibitor (DPP IV inhibitor)	dipeptidyl peptidase IV inhibitor	1	HA	[564-565]
8304	Dipeptidyl peptidase IV inhibitor (DPP IV inhibitor)	dipeptidyl peptidase IV inhibitor	1	IPA	[694-696]
8501	Dipeptidyl peptidase IV inhibitor (DPP IV inhibitor)	dipeptidyl peptidase IV inhibitor	2	IP	[7-8],[694-695]
8503	Dipeptidyl peptidase IV inhibitor (DPP IV inhibitor)	dipeptidyl peptidase IV inhibitor	1	TP	[531-532]
8505	Dipeptidyl peptidase IV inhibitor (DPP IV inhibitor)	dipeptidyl peptidase IV inhibitor	2	SP	[332-333],[361-362]
8518	dipeptidyl peptidase IV inhibitor (DPP IV inhibitor)	dipeptidyl peptidase IV inhibitor	1	RP	[571-572]
8520	dipeptidyl peptidase IV inhibitor (DPP IV inhibitor)	dipeptidyl peptidase IV inhibitor	2	HP	[698-699],[780-781]
8524	dipeptidyl peptidase IV inhibitor (DPP IV inhibitor)	dipeptidyl peptidase IV inhibitor	3	GA	[473-474],[509-510],[624-625]
8525	dipeptidyl peptidase IV inhibitor (DPP IV inhibitor)	dipeptidyl peptidase IV inhibitor	1	IA	[700-701]
8526	dipeptidyl peptidase IV inhibitor (DPP IV inhibitor)	dipeptidyl peptidase IV inhibitor	3	RA	[446-447],[552-553],[559-560]
8529	dipeptidyl peptidase IV inhibitor (DPP IV inhibitor)	dipeptidyl peptidase IV inhibitor	1	EP	[94-95]
8530	dipeptidyl peptidase IV inhibitor (DPP IV inhibitor)	dipeptidyl peptidase IV inhibitor	7	NP	[37-38],[147-148],[206-207],[264-265],[377-378],[424-425],[498-499]
8531	dipeptidyl peptidase IV inhibitor (DPP IV inhibitor)	dipeptidyl peptidase IV inhibitor	2	TA	[683-684],[703-704]
8532	dipeptidyl peptidase IV inhibitor (DPP IV inhibitor)	dipeptidyl peptidase IV inhibitor	2	QP	[114-115],[494-495]
8555	dipeptidyl peptidase IV inhibitor (DPP IV inhibitor)	dipeptidyl peptidase IV inhibitor	4	FL	[10-11],[12-13],[17-18],[726-727]
8558	dipeptidyl peptidase IV inhibitor (DPP IV inhibitor)	dipeptidyl peptidase IV inhibitor	2	EK	[107-108],[556-557]

8559	dipeptidyl peptidase IV inhibitor (DPP IV inhibitor)	dipeptidyl peptidase IV inhibitor	5	AL	[15-16],[19-20],[26-27],[528-529],[560-561]
8560	dipeptidyl peptidase IV inhibitor (DPP IV inhibitor)	dipeptidyl peptidase IV inhibitor	2	SL	[24-25],[583-584]
8561	dipeptidyl peptidase IV inhibitor (DPP IV inhibitor)	dipeptidyl peptidase IV inhibitor	1	GL	[385-386]
8637	dipeptidyl peptidase IV inhibitor (DPP IV inhibitor)	dipeptidyl peptidase IV inhibitor	2	AA	[507-508],[527-528]
8638	dipeptidyl peptidase IV inhibitor (DPP IV inhibitor)	dipeptidyl peptidase IV inhibitor	2	PL	[8-9],[781-782]
8689	dipeptidyl peptidase IV inhibitor (DPP IV inhibitor)	dipeptidyl peptidase IV inhibitor	1	LQP	[493-495]
8691	dipeptidyl peptidase IV inhibitor (DPP IV inhibitor)	dipeptidyl peptidase IV inhibitor	1	WE	[47-48]
8697	dipeptidyl peptidase IV inhibitor (DPP IV inhibitor)	dipeptidyl peptidase IV inhibitor	2	WG	[355-356],[573-574]
8758	dipeptidyl peptidase IV inhibitor (DPP IV inhibitor)	dipeptidyl peptidase IV inhibitor	2	AE	[436-437],[619-620]
8760	dipeptidyl peptidase IV inhibitor (DPP IV inhibitor)	dipeptidyl peptidase IV inhibitor	6	AG	[472-473],[508-509],[568-569],[696-697],[728-729],[789-790]
8762	dipeptidyl peptidase IV inhibitor (DPP IV inhibitor)	dipeptidyl peptidase IV inhibitor	4	AS	[360-361],[553-554],[704-705],[783-784]
8763	dipeptidyl peptidase IV inhibitor (DPP IV inhibitor)	dipeptidyl peptidase IV inhibitor	3	AT	[447-448],[474-475],[635-636]
8766	dipeptidyl peptidase IV inhibitor (DPP IV inhibitor)	dipeptidyl peptidase IV inhibitor	1	DN	[200-201]
8767	dipeptidyl peptidase IV inhibitor (DPP IV inhibitor)	dipeptidyl peptidase IV inhibitor	7	DP	[41-42],[91-92],[110-111],[150-151],[209-210],[267-268],[314-315]
8768	dipeptidyl peptidase IV inhibitor (DPP IV inhibitor)	dipeptidyl peptidase IV inhibitor	3	DQ	[262-263],[358-359],[541-542]
8769	dipeptidyl peptidase IV inhibitor (DPP IV inhibitor)	dipeptidyl peptidase IV inhibitor	2	DR	[311-312],[534-535]
8770	dipeptidyl peptidase IV inhibitor (DPP IV inhibitor)	dipeptidyl peptidase IV inhibitor	3	EG	[394-395],[547-548],[643-644]
8771	dipeptidyl peptidase IV inhibitor (DPP IV inhibitor)	dipeptidyl peptidase IV inhibitor	3	EH	[160-161],[219-220],[605-606]
8772	dipeptidyl peptidase IV inhibitor (DPP IV inhibitor)	dipeptidyl peptidase IV inhibitor	4	EI	[88-89],[620-621],[752-753],[755-756]

8773	dipeptidyl peptidase IV inhibitor (DPP IV inhibitor)	dipeptidyl peptidase IV inhibitor	8	ES	[43-44],[365-366],[391-392],[437-438],[458-459],[514-515],[673-674],[762-763]
8774	dipeptidyl peptidase IV inhibitor (DPP IV inhibitor)	dipeptidyl peptidase IV inhibitor	2	ET	[28-29],[104-105]
8775	dipeptidyl peptidase IV inhibitor (DPP IV inhibitor)	dipeptidyl peptidase IV inhibitor	1	EV	[396-397]
8776	dipeptidyl peptidase IV inhibitor (DPP IV inhibitor)	dipeptidyl peptidase IV inhibitor	1	EW	[354-355]
8777	dipeptidyl peptidase IV inhibitor (DPP IV inhibitor)	dipeptidyl peptidase IV inhibitor	4	EY	[463-464],[476-477],[504-505],[662-663]
8778	dipeptidyl peptidase IV inhibitor (DPP IV inhibitor)	dipeptidyl peptidase IV inhibitor	2	FN	[460-461],[746-747]
8779	dipeptidyl peptidase IV inhibitor (DPP IV inhibitor)	dipeptidyl peptidase IV inhibitor	3	FQ	[49-50],[125-126],[679-680]
8780	dipeptidyl peptidase IV inhibitor (DPP IV inhibitor)	dipeptidyl peptidase IV inhibitor	1	FR	[502-503]
8781	dipeptidyl peptidase IV inhibitor (DPP IV inhibitor)	dipeptidyl peptidase IV inhibitor	2	GE	[393-394],[395-396]
8782	dipeptidyl peptidase IV inhibitor (DPP IV inhibitor)	dipeptidyl peptidase IV inhibitor	2	GF	[714-715],[790-791]
8783	dipeptidyl peptidase IV inhibitor (DPP IV inhibitor)	dipeptidyl peptidase IV inhibitor	1	GG	[579-580]
8784	dipeptidyl peptidase IV inhibitor (DPP IV inhibitor)	dipeptidyl peptidase IV inhibitor	1	GH	[697-698]
8785	dipeptidyl peptidase IV inhibitor (DPP IV inhibitor)	dipeptidyl peptidase IV inhibitor	2	GI	[412-413],[716-717]
8786	dipeptidyl peptidase IV inhibitor (DPP IV inhibitor)	dipeptidyl peptidase IV inhibitor	1	GV	[548-549]
8789	dipeptidyl peptidase IV inhibitor (DPP IV inhibitor)	dipeptidyl peptidase IV inhibitor	2	HD	[40-41],[654-655]
8790	dipeptidyl peptidase IV inhibitor (DPP IV inhibitor)	dipeptidyl peptidase IV inhibitor	2	HE	[58-59],[390-391]
8792	dipeptidyl peptidase IV inhibitor (DPP IV inhibitor)	dipeptidyl peptidase IV inhibitor	1	HH	[39-40]
8794	dipeptidyl peptidase IV inhibitor (DPP IV inhibitor)	dipeptidyl peptidase IV inhibitor	3	HR	[45-46],[320-321],[606-607]
8795	dipeptidyl peptidase IV inhibitor (DPP IV inhibitor)	dipeptidyl peptidase IV inhibitor	1	HS	[382-383]

8799	dipeptidyl peptidase IV inhibitor (DPP IV inhibitor)	dipeptidyl peptidase IV inhibitor	1	HY	[630-631]
8801	dipeptidyl peptidase IV inhibitor (DPP IV inhibitor)	dipeptidyl peptidase IV inhibitor	2	II	[550-551],[733-734]
8802	dipeptidyl peptidase IV inhibitor (DPP IV inhibitor)	dipeptidyl peptidase IV inhibitor	3	IL	[420-421],[524-525],[785-786]
8804	dipeptidyl peptidase IV inhibitor (DPP IV inhibitor)	dipeptidyl peptidase IV inhibitor	3	IN	[479-480],[717-718],[734-735]
8806	dipeptidyl peptidase IV inhibitor (DPP IV inhibitor)	dipeptidyl peptidase IV inhibitor	2	IR	[468-469],[551-552]
8808	dipeptidyl peptidase IV inhibitor (DPP IV inhibitor)	dipeptidyl peptidase IV inhibitor	3	KE	[145-146],[348-349],[742-743]
8810	dipeptidyl peptidase IV inhibitor (DPP IV inhibitor)	dipeptidyl peptidase IV inhibitor	1	KG	[664-665]
8812	dipeptidyl peptidase IV inhibitor (DPP IV inhibitor)	dipeptidyl peptidase IV inhibitor	1	KI	[6-7]
8814	dipeptidyl peptidase IV inhibitor (DPP IV inhibitor)	dipeptidyl peptidase IV inhibitor	1	KR	[622-623]
8815	dipeptidyl peptidase IV inhibitor (DPP IV inhibitor)	dipeptidyl peptidase IV inhibitor	1	KS	[585-586]
8817	dipeptidyl peptidase IV inhibitor (DPP IV inhibitor)	dipeptidyl peptidase IV inhibitor	1	KV	[681-682]
8819	dipeptidyl peptidase IV inhibitor (DPP IV inhibitor)	dipeptidyl peptidase IV inhibitor	1	KY	[398-399]
8823	dipeptidyl peptidase IV inhibitor (DPP IV inhibitor)	dipeptidyl peptidase IV inhibitor	2	LN	[36-37],[529-530]
8824	dipeptidyl peptidase IV inhibitor (DPP IV inhibitor)	dipeptidyl peptidase IV inhibitor	1	LT	[449-450]
8825	dipeptidyl peptidase IV inhibitor (DPP IV inhibitor)	dipeptidyl peptidase IV inhibitor	4	LV	[20-21],[525-526],[615-616],[712-713]
8826	dipeptidyl peptidase IV inhibitor (DPP IV inhibitor)	dipeptidyl peptidase IV inhibitor	1	ME	[768-769]
8833	dipeptidyl peptidase IV inhibitor (DPP IV inhibitor)	dipeptidyl peptidase IV inhibitor	1	MM	[626-627]
8837	dipeptidyl peptidase IV inhibitor (DPP IV inhibitor)	dipeptidyl peptidase IV inhibitor	3	MV	[1-2],[489-490],[627-628]
8840	dipeptidyl peptidase IV inhibitor (DPP IV inhibitor)	dipeptidyl peptidase IV inhibitor	2	ND	[201-202],[522-523]

8841	dipeptidyl peptidase IV inhibitor (DPP IV inhibitor)	dipeptidyl peptidase IV inhibitor	3	NE	[34-35],[484-485],[707-708]
8842	dipeptidyl peptidase IV inhibitor (DPP IV inhibitor)	dipeptidyl peptidase IV inhibitor	1	NF	[725-726]
8843	dipeptidyl peptidase IV inhibitor (DPP IV inhibitor)	dipeptidyl peptidase IV inhibitor	1	NG	[718-719]
8845	dipeptidyl peptidase IV inhibitor (DPP IV inhibitor)	dipeptidyl peptidase IV inhibitor	2	NL	[461-462],[709-710]
8846	dipeptidyl peptidase IV inhibitor (DPP IV inhibitor)	dipeptidyl peptidase IV inhibitor	1	NM	[747-748]
8847	dipeptidyl peptidase IV inhibitor (DPP IV inhibitor)	dipeptidyl peptidase IV inhibitor	3	NN	[497-498],[721-722],[731-732]
8848	dipeptidyl peptidase IV inhibitor (DPP IV inhibitor)	dipeptidyl peptidase IV inhibitor	4	NQ	[480-481],[593-594],[722-723],[735-736]
8850	dipeptidyl peptidase IV inhibitor (DPP IV inhibitor)	dipeptidyl peptidase IV inhibitor	2	NT	[426-427],[530-531]
8853	dipeptidyl peptidase IV inhibitor (DPP IV inhibitor)	dipeptidyl peptidase IV inhibitor	2	NY	[415-416],[617-618]
8855	dipeptidyl peptidase IV inhibitor (DPP IV inhibitor)	dipeptidyl peptidase IV inhibitor	1	PG	[499-500]
8856	dipeptidyl peptidase IV inhibitor (DPP IV inhibitor)	dipeptidyl peptidase IV inhibitor	3	PH	[38-39],[629-630],[653-654]
8857	dipeptidyl peptidase IV inhibitor (DPP IV inhibitor)	dipeptidyl peptidase IV inhibitor	2	PI	[581-582],[699-700]
8859	dipeptidyl peptidase IV inhibitor (DPP IV inhibitor)	dipeptidyl peptidase IV inhibitor	1	PM	[767-768]
8860	dipeptidyl peptidase IV inhibitor (DPP IV inhibitor)	dipeptidyl peptidase IV inhibitor	1	PN	[425-426]
8861	dipeptidyl peptidase IV inhibitor (DPP IV inhibitor)	dipeptidyl peptidase IV inhibitor	2	PQ	[115-116],[315-316]
8864	dipeptidyl peptidase IV inhibitor (DPP IV inhibitor)	dipeptidyl peptidase IV inhibitor	1	PV	[495-496]
8865	dipeptidyl peptidase IV inhibitor (DPP IV inhibitor)	dipeptidyl peptidase IV inhibitor	1	PW	[572-573]
8866	dipeptidyl peptidase IV inhibitor (DPP IV inhibitor)	dipeptidyl peptidase IV inhibitor	2	PY	[378-379],[431-432]
8867	dipeptidyl peptidase IV inhibitor (DPP IV inhibitor)	dipeptidyl peptidase IV inhibitor	2	QA	[65-66],[359-360]

8868	dipeptidyl peptidase IV inhibitor (DPP IV inhibitor)	dipeptidyl peptidase IV inhibitor	3	QD	[109-110],[313-314],[481-482]
8869	dipeptidyl peptidase IV inhibitor (DPP IV inhibitor)	dipeptidyl peptidase IV inhibitor	14	QE	[53-54],[126-127],[129-130],[190-191],[218-219],[249-250],[286-287],[301-302],[329-330],[454-455],[543-544],[555-556],[610-611],[669-670]
8870	dipeptidyl peptidase IV inhibitor (DPP IV inhibitor)	dipeptidyl peptidase IV inhibitor	5	QF	[124-125],[501-502],[594-595],[597-598],[678-679]
8871	dipeptidyl peptidase IV inhibitor (DPP IV inhibitor)	dipeptidyl peptidase IV inhibitor	7	QG	[165-166],[167-168],[224-225],[226-227],[351-352],[384-385],[776-777]
8872	dipeptidyl peptidase IV inhibitor (DPP IV inhibitor)	dipeptidyl peptidase IV inhibitor	2	QH	[57-58],[563-564]
8874	dipeptidyl peptidase IV inhibitor (DPP IV inhibitor)	dipeptidyl peptidase IV inhibitor	4	QL	[76-77],[289-290],[608-609],[736-737]
8875	dipeptidyl peptidase IV inhibitor (DPP IV inhibitor)	dipeptidyl peptidase IV inhibitor	3	QN	[263-264],[706-707],[730-731]
8876	dipeptidyl peptidase IV inhibitor (DPP IV inhibitor)	dipeptidyl peptidase IV inhibitor	21	QQ	[50-51],[67-68],[113-114],[128-129],[171-172],[172-173],[173-174],[230-231],[231-232],[232-233],[276-277],[277-278],[288-289],[297-298],[300-301],[316-317],[317-318],[340-341],[350-351],[373-374],[542-543]
8877	dipeptidyl peptidase IV inhibitor (DPP IV inhibitor)	dipeptidyl peptidase IV inhibitor	6	QS	[176-177],[235-236],[331-332],[511-512],[659-660],[771-772]
8878	dipeptidyl peptidase IV inhibitor (DPP IV inhibitor)	dipeptidyl peptidase IV inhibitor	1	QT	[327-328]
8879	dipeptidyl peptidase IV inhibitor (DPP IV inhibitor)	dipeptidyl peptidase IV inhibitor	1	QV	[760-761]
8881	dipeptidyl peptidase IV inhibitor (DPP IV inhibitor)	dipeptidyl peptidase IV inhibitor	5	QY	[98-99],[154-155],[213-214],[318-319],[346-347]
8882	dipeptidyl peptidase IV inhibitor (DPP IV inhibitor)	dipeptidyl peptidase IV inhibitor	12	RG	[61-62],[138-139],[197-198],[256-257],[284-285],[308-309],[367-368],[411-412],[444-445],[623-624],[688-689],[774-775]
8883	dipeptidyl peptidase IV inhibitor (DPP IV inhibitor)	dipeptidyl peptidase IV inhibitor	2	RH	[375-376],[389-390]
8885	dipeptidyl peptidase IV inhibitor (DPP IV inhibitor)	dipeptidyl peptidase IV inhibitor	2	RK	[96-97],[247-248]
8886	dipeptidyl peptidase IV inhibitor (DPP IV inhibitor)	dipeptidyl peptidase IV inhibitor	5	RL	[241-242],[486-487],[535-536],[685-686],[711-712]
8888	dipeptidyl peptidase IV inhibitor (DPP IV inhibitor)	dipeptidyl peptidase IV inhibitor	1	RN	[724-725]
8889	dipeptidyl peptidase IV inhibitor (DPP IV inhibitor)	dipeptidyl peptidase IV inhibitor	21	RR	[71-72],[119-120],[133-134],[136-137],[137-138],[163-164],[188-189],[195-196],[196-197],[222-223],[254-255],[255-256],[283-284],[292-293],[293-294],[324-325],[363-364],[456-457],[575-576],[666-667],[773-774]
8890	dipeptidyl peptidase IV inhibitor (DPP IV inhibitor)	dipeptidyl peptidase IV inhibitor	1	RW	[46-47]

8891	dipeptidyl peptidase IV inhibitor (DPP IV inhibitor)	dipeptidyl peptidase IV inhibitor	2	SF	[459-460],[745-746]
8892	dipeptidyl peptidase IV inhibitor (DPP IV inhibitor)	dipeptidyl peptidase IV inhibitor	1	SH	[44-45]
8893	dipeptidyl peptidase IV inhibitor (DPP IV inhibitor)	dipeptidyl peptidase IV inhibitor	1	SI	[784-785]
8894	dipeptidyl peptidase IV inhibitor (DPP IV inhibitor)	dipeptidyl peptidase IV inhibitor	1	SK	[633-634]
8895	dipeptidyl peptidase IV inhibitor (DPP IV inhibitor)	dipeptidyl peptidase IV inhibitor	1	SV	[438-439]
8897	dipeptidyl peptidase IV inhibitor (DPP IV inhibitor)	dipeptidyl peptidase IV inhibitor	4	SY	[515-516],[590-591],[660-661],[763-764]
8899	dipeptidyl peptidase IV inhibitor (DPP IV inhibitor)	dipeptidyl peptidase IV inhibitor	5	TE	[29-30],[404-405],[407-408],[475-476],[513-514]
8900	dipeptidyl peptidase IV inhibitor (DPP IV inhibitor)	dipeptidyl peptidase IV inhibitor	2	TF	[427-428],[450-451]
8901	dipeptidyl peptidase IV inhibitor (DPP IV inhibitor)	dipeptidyl peptidase IV inhibitor	2	TG	[645-646],[676-677]
8904	dipeptidyl peptidase IV inhibitor (DPP IV inhibitor)	dipeptidyl peptidase IV inhibitor	1	TK	[3-4]
8905	dipeptidyl peptidase IV inhibitor (DPP IV inhibitor)	dipeptidyl peptidase IV inhibitor	1	TL	[448-449]
8908	dipeptidyl peptidase IV inhibitor (DPP IV inhibitor)	dipeptidyl peptidase IV inhibitor	1	TQ	[328-329]
8909	dipeptidyl peptidase IV inhibitor (DPP IV inhibitor)	dipeptidyl peptidase IV inhibitor	1	TR	[443-444]
8912	dipeptidyl peptidase IV inhibitor (DPP IV inhibitor)	dipeptidyl peptidase IV inhibitor	1	TV	[636-637]
8915	dipeptidyl peptidase IV inhibitor (DPP IV inhibitor)	dipeptidyl peptidase IV inhibitor	1	VD	[90-91]
8916	dipeptidyl peptidase IV inhibitor (DPP IV inhibitor)	dipeptidyl peptidase IV inhibitor	2	VE	[642-643],[761-762]
8917	dipeptidyl peptidase IV inhibitor (DPP IV inhibitor)	dipeptidyl peptidase IV inhibitor	1	VF	[519-520]
8918	dipeptidyl peptidase IV inhibitor (DPP IV inhibitor)	dipeptidyl peptidase IV inhibitor	1	VG	[713-714]
8920	dipeptidyl peptidase IV inhibitor (DPP IV inhibitor)	dipeptidyl peptidase IV inhibitor	6	VI	[419-420],[439-440],[467-468],[478-479],[549-550],[693-694]

8921	dipeptidyl peptidase IV inhibitor (DPP IV inhibitor)	dipeptidyl peptidase IV inhibitor	2	VK	[397-398],[490-491]
8922	dipeptidyl peptidase IV inhibitor (DPP IV inhibitor)	dipeptidyl peptidase IV inhibitor	2	VL	[429-430],[614-615]
8924	dipeptidyl peptidase IV inhibitor (DPP IV inhibitor)	dipeptidyl peptidase IV inhibitor	2	VN	[496-497],[616-617]
8926	dipeptidyl peptidase IV inhibitor (DPP IV inhibitor)	dipeptidyl peptidase IV inhibitor	2	VS	[452-453],[656-657]
8927	dipeptidyl peptidase IV inhibitor (DPP IV inhibitor)	dipeptidyl peptidase IV inhibitor	3	VT	[2-3],[442-443],[682-683]
8929	dipeptidyl peptidase IV inhibitor (DPP IV inhibitor)	dipeptidyl peptidase IV inhibitor	1	VY	[639-640]
8932	dipeptidyl peptidase IV inhibitor (DPP IV inhibitor)	dipeptidyl peptidase IV inhibitor	2	YA	[506-507],[618-619]
8934	dipeptidyl peptidase IV inhibitor (DPP IV inhibitor)	dipeptidyl peptidase IV inhibitor	3	YE	[99-100],[272-273],[661-662]
8935	dipeptidyl peptidase IV inhibitor (DPP IV inhibitor)	dipeptidyl peptidase IV inhibitor	2	YF	[380-381],[764-765]
8936	dipeptidyl peptidase IV inhibitor (DPP IV inhibitor)	dipeptidyl peptidase IV inhibitor	1	YG	[464-465]
8937	dipeptidyl peptidase IV inhibitor (DPP IV inhibitor)	dipeptidyl peptidase IV inhibitor	2	YH	[319-320],[432-433]
8939	dipeptidyl peptidase IV inhibitor (DPP IV inhibitor)	dipeptidyl peptidase IV inhibitor	2	YK	[347-348],[663-664]
8940	dipeptidyl peptidase IV inhibitor (DPP IV inhibitor)	dipeptidyl peptidase IV inhibitor	2	YL	[399-400],[516-517]
8942	dipeptidyl peptidase IV inhibitor (DPP IV inhibitor)	dipeptidyl peptidase IV inhibitor	1	YN	[631-632]
8944	dipeptidyl peptidase IV inhibitor (DPP IV inhibitor)	dipeptidyl peptidase IV inhibitor	3	YR	[155-156],[214-215],[416-417]
8945	dipeptidyl peptidase IV inhibitor (DPP IV inhibitor)	dipeptidyl peptidase IV inhibitor	1	YS	[591-592]
8946	dipeptidyl peptidase IV inhibitor (DPP IV inhibitor)	dipeptidyl peptidase IV inhibitor	2	YV	[477-478],[640-641]
8948	dipeptidyl peptidase IV inhibitor (DPP IV inhibitor)	dipeptidyl peptidase IV inhibitor	2	YY	[379-380],[505-506]
9476	DPP-III inhibitor	dipeptidyl peptidase III inhibitor	2	YY	[379-380],[505-506]

9477	DPP-III inhibitor	dipeptidyl peptidase III inhibitor	1	RW	[46-47]
9478	DPP-III inhibitor	dipeptidyl peptidase III inhibitor	6	LR	[77-78],[386-387],[410-411],[517-518],[558-559],[710-711]
9480	DPP-III inhibitor	dipeptidyl peptidase III inhibitor	2	YF	[380-381],[764-765]
9481	DPP-III inhibitor	dipeptidyl peptidase III inhibitor	2	YH	[319-320],[432-433]
9482	DPP-III inhibitor	dipeptidyl peptidase III inhibitor	2	YL	[399-400],[516-517]
9483	DPP-III inhibitor	dipeptidyl peptidase III inhibitor	2	YK	[347-348],[663-664]
9484	DPP-III inhibitor	dipeptidyl peptidase III inhibitor	3	YR	[155-156],[214-215],[416-417]
9485	DPP-III inhibitor	dipeptidyl peptidase III inhibitor	21	RR	[71-72],[119-120],[133-134],[136-137],[137-138],[163-164],[188-189],[195-196],[196-197],[222-223],[254-255],[255-256],[283-284],[292-293],[293-294],[324-325],[363-364],[456-457],[575-576],[666-667],[773-774]
9486	DPP-III inhibitor	dipeptidyl peptidase III inhibitor	2	TF	[427-428],[450-451]
9487	DPP-III inhibitor	dipeptidyl peptidase III inhibitor	2	GE	[393-394],[395-396]
9488	DPP-III inhibitor	dipeptidyl peptidase III inhibitor	2	GF	[714-715],[790-791]
9489	DPP-III inhibitor	dipeptidyl peptidase III inhibitor	11	PR	[92-93],[95-96],[111-112],[148-149],[151-152],[207-208],[210-211],[265-266],[362-363],[532-533],[749-750]
9490	DPP-III inhibitor	dipeptidyl peptidase III inhibitor	4	RF	[182-183],[402-403],[538-539],[647-648]
9491	DPP-III inhibitor	dipeptidyl peptidase III inhibitor	3	RV	[417-418],[469-470],[518-519]
9492	DPP-III inhibitor	dipeptidyl peptidase III inhibitor	1	DA	[435-436]
9494	DPP-III inhibitor	dipeptidyl peptidase III inhibitor	1	HK	[433-434]
9496	DPP-III inhibitor	dipeptidyl peptidase III inhibitor	2	HP	[698-699],[780-781]
9499	DPP-III inhibitor	dipeptidyl peptidase III inhibitor	5	LA	[18-19],[25-26],[686-687],[727-728],[782-783]
9500	DPP-III inhibitor	dipeptidyl peptidase III inhibitor	1	FA	[788-789]

9501	DPP-III inhibitor	dipeptidyl peptidase III inhibitor	1	FR	[502-503]
9502	DPP-III inhibitor	dipeptidyl peptidase III inhibitor	4	FL	[10-11],[12-13],[17-18],[726-727]
9504	DPP-III inhibitor	dipeptidyl peptidase III inhibitor	4	PE	[42-43],[268-269],[333-334],[603-604]
9508	DPP-III inhibitor	dipeptidyl peptidase III inhibitor	1	YG	[464-465]
9509	DPP-III inhibitor	dipeptidyl peptidase III inhibitor	1	VY	[639-640]
9511	DPP-III inhibitor	dipeptidyl peptidase III inhibitor	2	KA	[4-5],[634-635]
8247	CaMPDE inhibitor	CaMPDE inhibitor	2	IR	[468-469],[551-552]
8250	CaMPDE inhibitor	CaMPDE inhibitor	1	EF	[48-49]
2835	Renin inhibitor	renin inhibitor	1	FT	[403-404]
2842	Renin inhibitor	renin inhibitor	6	LR	[77-78],[386-387],[410-411],[517-518],[558-559],[710-711]
8246	renin inhibitor	renin inhibitor	2	IR	[468-469],[551-552]
8251	Renin inhibitor	renin inhibitor	1	EF	[48-49]
9431	Renin inhibitor	renin inhibitor	5	QF	[124-125],[501-502],[594-595],[597-598],[678-679]
9432	Renin inhibitor	renin inhibitor	2	SF	[459-460],[745-746]
9433	Renin inhibitor	renin inhibitor	2	YA	[506-507],[618-619]
9471	Renin inhibitor	renin inhibitor	2	TF	[427-428],[450-451]

Table S12. Profile of potential biological activity of fragments of protein Car i 4.0101.

ID	Name of peptide	Activity	Number	Sequence	Location
3460	Prolyl endopeptidase inhibitor	antiamnestic	1	PG	[111-112]
3257	beta-lactokinin	ACE inhibitor	3	RL	[41-42],[256-257],[479-480]
3258	beta-lactokinin	ACE inhibitor	3	IR	[140-141],[278-279],[458-459]
3381	ACE inhibitor	ACE inhibitor	2	LY	[370-371],[375-376]
3383	ACE inhibitor	ACE inhibitor	2	IY	[9-10],[339-340]
3384	ACE inhibitor	ACE inhibitor	2	VF	[234-235],[407-408]
3492	ACE inhibitor from sake	ACE inhibitor	2	VY	[97-98],[388-389]
3494	ACE inhibitor from sake	ACE inhibitor	1	HY	[89-90]
3502	ACE inhibitor (BSA fr. 221-222)	ACE inhibitor	2	FP	[110-111],[151-152]
3528	ACE inhibitor	ACE inhibitor	1	LVR	[489-491]
3537	ACE inhibitor	ACE inhibitor	3	PR	[186-187],[280-281],[473-474]
3538	ACE inhibitor	ACE inhibitor	1	VSP	[448-450]
3547	ACE inhibitor	ACE inhibitor	1	IRA	[458-460]
3551	ACE inhibitor (from bovine beta-Lg)	ACE inhibitor	2	LF	[18-19],[109-110]
3556	ACE inhibitor	ACE inhibitor	1	FY	[189-190]
3573		ACE inhibitor	1	AFP	[150-152]
3713	ACE inhibitor from alpha-zein	ACE inhibitor	1	LLP	[86-88]
7414	ACE inhibitor	ACE inhibitor	1	LRW	[358-360]
7502	ACE inhibitor	ACE inhibitor	1	IVR	[267-269]
7513	ACE inhibitor from Alaskan pollack skin	ACE inhibitor	1	PL	[450-451]
7547	ACE inhibitor	ACE inhibitor	1	IRP	[278-280]
7558	ACE inhibitor from buckwheat	ACE inhibitor	1	VK	[427-428]
7562	ACE inhibitor from soy hydrolysate	ACE inhibitor	2	IA	[99-100],[149-150]
7580	ACE inhibitor	ACE inhibitor	2	RW	[281-282],[359-360]
7581	ACE inhibitor	ACE inhibitor	2	IP	[420-421],[472-473]
7582	ACE inhibitor	ACE inhibitor	2	RP	[200-201],[279-280]
7583	ACE inhibitor	ACE inhibitor	3	AF	[150-151],[246-247],[469-470]
7584	ACE inhibitor	ACE inhibitor	1	AP	[93-94]
7585	ACE inhibitor	ACE inhibitor	4	LA	[23-24],[191-192],[243-244],[451-452]
7586	ACE inhibitor	ACE inhibitor	1	KR	[428-429]
7587	ACE inhibitor	ACE inhibitor	1	VP	[377-378]
7588	ACE inhibitor	ACE inhibitor	5	RA	[336-337],[394-395],[429-430],[459-460],[502-503]
7589	ACE inhibitor	ACE inhibitor	1	YA	[389-390]

7591	ACE inhibitor	ACE inhibitor	2	GF	[237-238],[434-435]
7592	ACE inhibitor	ACE inhibitor	2	FR	[143-144],[199-200]
7593	ACE inhibitor	ACE inhibitor	1	IF	[170-171]
7595	ACE inhibitor	ACE inhibitor	1	IG	[331-332]
7596	ACE inhibitor	ACE inhibitor	1	GI	[104-105]
7598	ACE inhibitor	ACE inhibitor	1	GA	[368-369]
7599	ACE inhibitor	ACE inhibitor	2	GL	[84-85],[317-318]
7600	ACE inhibitor	ACE inhibitor	6	AG	[55-56],[71-72],[153-154],[192-193],[344-345],[452-453]
7603	ACE inhibitor	ACE inhibitor	7	GR	[28-29],[102-103],[272-273],[312-313],[345-346],[393-394],[453-454]
7605	ACE inhibitor	ACE inhibitor	2	FG	[34-35],[403-404]
7606	ACE inhibitor	ACE inhibitor	5	DA	[43-44],[239-240],[245-246],[373-374],[476-477]
7607	ACE inhibitor	ACE inhibitor	2	GS	[163-164],[265-266]
7608	ACE inhibitor	ACE inhibitor	4	GV	[56-57],[72-73],[107-108],[154-155]
7610	ACE inhibitor	ACE inhibitor	5	GQ	[35-36],[127-128],[203-204],[404-405],[415-416]
7614	ACE inhibitor	ACE inhibitor	1	HG	[223-224]
7615	ACE inhibitor	ACE inhibitor	2	GE	[221-222],[224-225]
7616	ACE inhibitor	ACE inhibitor	2	GG	[27-28],[311-312]
7617	ACE inhibitor	ACE inhibitor	2	QG	[126-127],[202-203]
7618	ACE inhibitor	ACE inhibitor	2	SG	[26-27],[236-237]
7619	ACE inhibitor	ACE inhibitor	1	LG	[230-231]
7620	ACE inhibitor	ACE inhibitor	2	GD	[146-147],[332-333]
7621	ACE inhibitor	ACE inhibitor	1	TG	[106-107]
7622	ACE inhibitor	ACE inhibitor	4	EG	[145-146],[271-272],[414-415],[433-434]
7623	ACE inhibitor	ACE inhibitor	3	EA	[52-53],[54-55],[343-344]
7624	ACE inhibitor	ACE inhibitor	3	NG	[20-21],[83-84],[316-317]
7625	ACE inhibitor	ACE inhibitor	1	PG	[111-112]
7628	ACE inhibitor from k-CN (fr. 67-68)	ACE inhibitor	3	VR	[76-77],[268-269],[490-491]
7634	ACE inhibitor from k-CN (fr. 21-23)	ACE inhibitor	1	YVP	[376-378]
7635	ACE inhibitor from k-CN (fr. 51-53)	ACE inhibitor	1	VAV	[73-75]
7680	ACE inhibitor from pea vicilin	ACE inhibitor	1	QK	[138-139]
7681	ACE inhibitor from soy	ACE inhibitor	1	DG	[162-163]
7683	ACE inhibitor from garlic	ACE inhibitor	3	NF	[188-189],[402-403],[423-424]
7685	ACE inhibitor from garlic	ACE inhibitor	1	SF	[439-440]
7692	ACE inhibitor	ACE inhibitor	2	KF	[33-34],[481-482]
7741	ACE inhibitor	ACE inhibitor	9	RR	[77-78],[129-130],[211-212],[219-220],[255-256],[305-306],[309-310],[478-479],[501-502]

7742	ACE inhibitor	ACE inhibitor	4	AR	[100-101],[254-255],[430-431],[477-478]
7752	ACE inhibitor from shark meat hydrolysate	ACE inhibitor	1	EY	[206-207]
7810	ACE inhibitor from anchovy and bonito	ACE inhibitor	1	KP	[3-4]
7827	ACE inhibitor	ACE inhibitor	3	IE	[51-52],[58-59],[80-81]
7828	ACE inhibitor	ACE inhibitor	3	EV	[396-397],[464-465],[504-505]
7829	ACE inhibitor	ACE inhibitor	1	VE	[270-271]
7830	ACE inhibitor	ACE inhibitor	2	TE	[251-252],[341-342]
7831	ACE inhibitor	ACE inhibitor	4	LQ	[68-69],[257-258],[275-276],[361-362]
7832	ACE inhibitor	ACE inhibitor	2	LN	[39-40],[382-383]
7833	ACE inhibitor	ACE inhibitor	1	PT	[47-48]
7835	ACE inhibitor	ACE inhibitor	2	AH	[156-157],[384-385]
7837	ACE inhibitor	ACE inhibitor	3	PQ	[94-95],[201-202],[421-422]
7838	ACE inhibitor	ACE inhibitor	1	EW	[436-437]
7841	ACE inhibitor	ACE inhibitor	1	KE	[293-294]
7843	ACE inhibitor	ACE inhibitor	2	PH	[88-89],[378-379]
7844	ACE inhibitor	ACE inhibitor	1	HK	[32-33]
7859	ACE inhibitor	ACE inhibitor	1	IEP	[80-82]
8182	ACE Inhibitor	ACE inhibitor	1	ALEP	[44-47]
8185	ACE inhibitor	ACE inhibitor	1	TF	[116-117]
8193	ACE inhibitor	ACE inhibitor	2	AI	[169-170],[457-458]
8402	ACE inhibitor	ACE inhibitor	1	LVY	[96-98]
8951	ACE inhibitor	ACE inhibitor	2	AV	[74-75],[425-426]
9029	ACE inhibitor	ACE inhibitor	1	ALP	[460-462]
9031	ACE inhibitor	ACE inhibitor	1	LEE	[318-320]
9039	ACE inhibitor	ACE inhibitor	1	IFL	[170-172]
9046	ACE inhibitor	ACE inhibitor	1	VQV	[397-399]
9076	ACE inhibitor	ACE inhibitor	2	FQ	[132-133],[470-471]
9077	ACE inhibitor	ACE inhibitor	1	YV	[376-377]
9078	ACE inhibitor	ACE inhibitor	1	YE	[207-208]
9079	ACE inhibitor	ACE inhibitor	2	IL	[5-6],[357-358]
9107	ACE inhibitor	ACE inhibitor	1	WL	[360-361]
9160	ACE inhibitor	ACE inhibitor	1	TLS	[324-326]
9173	ACE inhibitor	ACE inhibitor	7	RG	[101-102],[103-104],[220-221],[264-265],[310-311],[367-368],[392-393]
9184	ACE inhibitor	ACE inhibitor	2	ST	[348-349],[487-488]
9185	ACE inhibitor	ACE inhibitor	1	YN	[160-161]
9196	ACE inhibitor	ACE inhibitor	2	AVV	[74-76],[425-427]

9213	ACE inhibitor	ACE inhibitor	4	LR	[327-328],[358-359],[391-392],[412-413]
9265	ACE inhibitor	ACE inhibitor	1	TLS	[324-326]
9468	ACE inhibitor	ACE inhibitor	1	RALP	[459-462]
9708	ACE inhibitor	ACE inhibitor	1	LPILR	[355-359]
9729	ACE inhibitor	ACE inhibitor	1	VVR	[75-77]
9730	ACE inhibitor	ACE inhibitor	1	NPR	[185-187]
3285	Antithrombotic peptide	antithrombotic	1	PG	[111-112]
3356	Stimulating vasoactive substance release	stimulating	1	LLL	[85-87]
8320	Glucose uptake stimulating peptide	stimulating	2	VL	[108-109],[465-466]
8321	Glucose uptake stimulating peptide	stimulating	3	LV	[96-97],[466-467],[489-490]
8322	Glucose uptake stimulating peptide	stimulating	2	IV	[15-16],[267-268]
8323	Glucose uptake stimulating peptide	stimulating	2	IL	[5-6],[357-358]
8324	Glucose uptake stimulating peptide	stimulating	1	LI	[13-14]
8325	Glucose uptake stimulating peptide	stimulating	2	II	[14-15],[148-149]
8326	Glucose uptake stimulating peptide	stimulating	5	LL	[6-7],[85-86],[86-87],[172-173],[417-418]
8329	Stimulating vasoactive substance release	stimulating	6	EE	[118-119],[285-286],[290-291],[319-320],[342-343],[463-464]
8330	Stimulating vasoactive substance release	stimulating	4	SE	[259-260],[301-302],[303-304],[499-500]
3066	Immunostimulating peptide	immunostimulating	1	EAE	[52-54]
2890	neuropeptide	neuropeptide	5	GQ	[35-36],[127-128],[203-204],[404-405],[415-416]
8310	Anxiolytic peptide	neuropeptide	2	YL	[10-11],[190-191]
2754	peptide regulating the stomach mucosal membrane activity	regulating	1	PG	[111-112]
3314		antioxidative	1	LLPH	[86-89]
7872	peptide from soybean protein isolates: beta-conglycinin and glycinin	antioxidative	2	LY	[370-371],[375-376]
7873	peptide from soybean protein isolates: beta-conglycinin and glycinin	antioxidative	2	IY	[9-10],[339-340]
7886	peptide derived from egg white albumin	antioxidative	2	AH	[156-157],[384-385]
7888	antioxidative peptide	antioxidative	1	EL	[411-412]
8037	synthetic peptide	antioxidative	1	PHW	[378-380]
8038	synthetic peptide	antioxidative	1	PHY	[88-90]
8057	synthetic peptide	antioxidative	1	RHF	[141-143]
8064	synthetic peptide	antioxidative	1	RHQ	[136-138]
8076	synthetic peptide	antioxidative	1	RWL	[359-361]
8214	Antioxidative peptide	antioxidative	2	RW	[281-282],[359-360]
8215	Antioxidative peptide	antioxidative	3	IR	[140-141],[278-279],[458-459]
8217	Antioxidative peptide	antioxidative	1	LK	[480-481]

8218	Antioxidative peptide	antioxidative	1	KP	[3-4]
8220	antioxidative peptide	antioxidative	1	TFE	[116-118]
8224	antioxidative peptide	antioxidative	2	VY	[97-98],[388-389]
9363	Antioxidative peptide	antioxidative	1	NEN	[443-445]
9716	Antioxidative peptide	antioxidative	1	LPILR	[355-359]
8661	Hypotensive peptide	hypotensive	1	LPILR	[355-359]
4005		activating ubiquitin-mediated proteolysis	5	RA	[336-337],[394-395],[429-430],[459-460],[502-503]
4006	Ubiqitin-mediated proteolysis activating peptide	activating ubiquitin-mediated proteolysis	4	LA	[23-24],[191-192],[243-244],[451-452]
9580	Hypolipidemic peptide	hypolipidemic	3	EF	[131-132],[198-199],[241-242]
9650	Alpha-glucosidase inhibitor	alpha-glucosidase inhibitor	3	EA	[52-53],[54-55],[343-344]
9693	Alpha-glucosidase inhibitor	alpha-glucosidase inhibitor	1	VE	[270-271]
9694	Alpha-glucosidase inhibitor	alpha-glucosidase inhibitor	2	PE	[114-115],[462-463]
9695	Alpha-glucosidase inhibitor	alpha-glucosidase inhibitor	2	AD	[244-245],[337-338]
3172	dipeptidyl peptidase IV inhibitor (DPP IV inhibitor)	dipeptidyl peptidase IV inhibitor	4	VA	[16-17],[73-74],[155-156],[168-169]
3173	dipeptidyl peptidase IV inhibitor (DPP IV inhibitor)	dipeptidyl peptidase IV inhibitor	1	MA	[1-2]
3175	dipeptidyl peptidase IV inhibitor (DPP IV inhibitor)	dipeptidyl peptidase IV inhibitor	4	LA	[23-24],[191-192],[243-244],[451-452]
3176	dipeptidyl peptidase IV inhibitor (DPP IV inhibitor)	dipeptidyl peptidase IV inhibitor	1	FA	[424-425]
3177	dipeptidyl peptidase IV inhibitor (DPP IV inhibitor)	dipeptidyl peptidase IV inhibitor	1	AP	[93-94]
3179	dipeptidyl peptidase IV inhibitor (DPP IV inhibitor)	dipeptidyl peptidase IV inhibitor	1	PA	[152-153]
3180	dipeptidyl peptidase IV inhibitor (DPP IV inhibitor)	dipeptidyl peptidase IV inhibitor	3	LP	[87-88],[355-356],[461-462]
3181	dipeptidyl peptidase IV inhibitor (DPP IV inhibitor)	dipeptidyl peptidase IV inhibitor	1	VP	[377-378]
3182	dipeptidyl peptidase IV inhibitor (DPP IV inhibitor)	dipeptidyl peptidase IV inhibitor	5	LL	[6-7],[85-86],[86-87],[172-173],[417-418]
3183	dipeptidyl peptidase IV inhibitor (DPP IV inhibitor)	dipeptidyl peptidase IV inhibitor	5	VV	[75-76],[167-168],[387-388],[399-400],[426-427]
8501	Dipeptidyl peptidase IV inhibitor (DPP IV inhibitor)	dipeptidyl peptidase IV inhibitor	2	IP	[420-421],[472-473]
8505	Dipeptidyl peptidase IV inhibitor (DPP IV inhibitor)	dipeptidyl peptidase IV inhibitor	2	SP	[165-166],[449-450]
8506	dipeptidyl peptidase IV inhibitor (DPP IV inhibitor)	dipeptidyl peptidase IV inhibitor	2	FP	[110-111],[151-152]
8518	dipeptidyl peptidase IV inhibitor (DPP IV inhibitor)	dipeptidyl peptidase IV inhibitor	2	RP	[200-201],[279-280]
8519	dipeptidyl peptidase IV inhibitor (DPP IV inhibitor)	dipeptidyl peptidase IV inhibitor	1	KP	[3-4]
8524	dipeptidyl peptidase IV inhibitor (DPP IV inhibitor)	dipeptidyl peptidase IV inhibitor	1	GA	[368-369]
8525	dipeptidyl peptidase IV inhibitor (DPP IV inhibitor)	dipeptidyl peptidase IV inhibitor	2	IA	[99-100],[149-150]
8526	dipeptidyl peptidase IV inhibitor (DPP IV inhibitor)	dipeptidyl peptidase IV inhibitor	5	RA	[336-337],[394-395],[429-430],[459-460],[502-503]
8529	dipeptidyl peptidase IV inhibitor (DPP IV inhibitor)	dipeptidyl peptidase IV inhibitor	2	EP	[46-47],[81-82]
8530	dipeptidyl peptidase IV inhibitor (DPP IV inhibitor)	dipeptidyl peptidase IV inhibitor	2	NP	[185-186],[194-195]
8531	dipeptidyl peptidase IV inhibitor (DPP IV inhibitor)	dipeptidyl peptidase IV inhibitor	1	TA	[253-254]

8555	dipeptidyl peptidase IV inhibitor (DPP IV inhibitor)	dipeptidyl peptidase IV inhibitor	2	FL	[171-172],[242-243]
8556	dipeptidyl peptidase IV inhibitor (DPP IV inhibitor)	dipeptidyl peptidase IV inhibitor	1	WV	[437-438]
8559	dipeptidyl peptidase IV inhibitor (DPP IV inhibitor)	dipeptidyl peptidase IV inhibitor	6	AL	[17-18],[44-45],[369-370],[374-375],[390-391],[460-461]
8560	dipeptidyl peptidase IV inhibitor (DPP IV inhibitor)	dipeptidyl peptidase IV inhibitor	1	SL	[326-327]
8561	dipeptidyl peptidase IV inhibitor (DPP IV inhibitor)	dipeptidyl peptidase IV inhibitor	2	GL	[84-85],[317-318]
8594	dipeptidyl peptidase IV inhibitor (DPP IV inhibitor)	dipeptidyl peptidase IV inhibitor	3	VR	[76-77],[268-269],[490-491]
8638	dipeptidyl peptidase IV inhibitor (DPP IV inhibitor)	dipeptidyl peptidase IV inhibitor	1	PL	[450-451]
8677	dipeptidyl peptidase IV inhibitor (DPP IV inhibitor)	dipeptidyl peptidase IV inhibitor	1	WL	[360-361]
8680	dipeptidyl peptidase IV inhibitor (DPP IV inhibitor)	dipeptidyl peptidase IV inhibitor	1	WN	[380-381]
8684	dipeptidyl peptidase IV inhibitor (DPP IV inhibitor)	dipeptidyl peptidase IV inhibitor	1	WC	[158-159]
8687	dipeptidyl peptidase IV inhibitor (DPP IV inhibitor)	dipeptidyl peptidase IV inhibitor	1	WS	[282-283]
8696	dipeptidyl peptidase IV inhibitor (DPP IV inhibitor)	dipeptidyl peptidase IV inhibitor	1	YT	[340-341]
8757	dipeptidyl peptidase IV inhibitor (DPP IV inhibitor)	dipeptidyl peptidase IV inhibitor	2	AD	[244-245],[337-338]
8758	dipeptidyl peptidase IV inhibitor (DPP IV inhibitor)	dipeptidyl peptidase IV inhibitor	5	AE	[53-54],[240-241],[365-366],[395-396],[503-504]
8759	dipeptidyl peptidase IV inhibitor (DPP IV inhibitor)	dipeptidyl peptidase IV inhibitor	3	AF	[150-151],[246-247],[469-470]
8760	dipeptidyl peptidase IV inhibitor (DPP IV inhibitor)	dipeptidyl peptidase IV inhibitor	6	AG	[55-56],[71-72],[153-154],[192-193],[344-345],[452-453]
8761	dipeptidyl peptidase IV inhibitor (DPP IV inhibitor)	dipeptidyl peptidase IV inhibitor	2	AH	[156-157],[384-385]
8764	dipeptidyl peptidase IV inhibitor (DPP IV inhibitor)	dipeptidyl peptidase IV inhibitor	2	AV	[74-75],[425-426]
8766	dipeptidyl peptidase IV inhibitor (DPP IV inhibitor)	dipeptidyl peptidase IV inhibitor	2	DN	[315-316],[401-402]
8767	dipeptidyl peptidase IV inhibitor (DPP IV inhibitor)	dipeptidyl peptidase IV inhibitor	2	DP	[62-63],[333-334]
8768	dipeptidyl peptidase IV inhibitor (DPP IV inhibitor)	dipeptidyl peptidase IV inhibitor	1	DQ	[183-184]
8769	dipeptidyl peptidase IV inhibitor (DPP IV inhibitor)	dipeptidyl peptidase IV inhibitor	1	DR	[135-136]
8770	dipeptidyl peptidase IV inhibitor (DPP IV inhibitor)	dipeptidyl peptidase IV inhibitor	4	EG	[145-146],[271-272],[414-415],[433-434]
8771	dipeptidyl peptidase IV inhibitor (DPP IV inhibitor)	dipeptidyl peptidase IV inhibitor	2	EH	[222-223],[288-289]
8773	dipeptidyl peptidase IV inhibitor (DPP IV inhibitor)	dipeptidyl peptidase IV inhibitor	5	ES	[59-60],[119-120],[300-301],[302-303],[486-487]
8774	dipeptidyl peptidase IV inhibitor (DPP IV inhibitor)	dipeptidyl peptidase IV inhibitor	3	ET	[115-116],[252-253],[320-321]
8775	dipeptidyl peptidase IV inhibitor (DPP IV inhibitor)	dipeptidyl peptidase IV inhibitor	3	EV	[396-397],[464-465],[504-505]
8776	dipeptidyl peptidase IV inhibitor (DPP IV inhibitor)	dipeptidyl peptidase IV inhibitor	1	EW	[436-437]
8777	dipeptidyl peptidase IV inhibitor (DPP IV inhibitor)	dipeptidyl peptidase IV inhibitor	1	EY	[206-207]
8778	dipeptidyl peptidase IV inhibitor (DPP IV inhibitor)	dipeptidyl peptidase IV inhibitor	3	FN	[19-20],[247-248],[482-483]
8779	dipeptidyl peptidase IV inhibitor (DPP IV inhibitor)	dipeptidyl peptidase IV inhibitor	2	FQ	[132-133],[470-471]
8780	dipeptidyl peptidase IV inhibitor (DPP IV inhibitor)	dipeptidyl peptidase IV inhibitor	2	FR	[143-144],[199-200]
8781	dipeptidyl peptidase IV inhibitor (DPP IV inhibitor)	dipeptidyl peptidase IV inhibitor	2	GE	[221-222],[224-225]
8782	dipeptidyl peptidase IV inhibitor (DPP IV inhibitor)	dipeptidyl peptidase IV inhibitor	2	GF	[237-238],[434-435]
8783	dipeptidyl peptidase IV inhibitor (DPP IV inhibitor)	dipeptidyl peptidase IV inhibitor	2	GG	[27-28],[311-312]
8785	dipeptidyl peptidase IV inhibitor (DPP IV inhibitor)	dipeptidyl peptidase IV inhibitor	1	GI	[104-105]

8786	dipeptidyl peptidase IV inhibitor (DPP IV inhibitor)	dipeptidyl peptidase IV inhibitor	4	GV	[56-57],[72-73],[107-108],[154-155]
8790	dipeptidyl peptidase IV inhibitor (DPP IV inhibitor)	dipeptidyl peptidase IV inhibitor	1	HE	[289-290]
8791	dipeptidyl peptidase IV inhibitor (DPP IV inhibitor)	dipeptidyl peptidase IV inhibitor	1	HF	[142-143]
8794	dipeptidyl peptidase IV inhibitor (DPP IV inhibitor)	dipeptidyl peptidase IV inhibitor	2	HR	[210-211],[263-264]
8795	dipeptidyl peptidase IV inhibitor (DPP IV inhibitor)	dipeptidyl peptidase IV inhibitor	1	HS	[385-386]
8798	dipeptidyl peptidase IV inhibitor (DPP IV inhibitor)	dipeptidyl peptidase IV inhibitor	2	HW	[157-158],[379-380]
8799	dipeptidyl peptidase IV inhibitor (DPP IV inhibitor)	dipeptidyl peptidase IV inhibitor	1	HY	[89-90]
8801	dipeptidyl peptidase IV inhibitor (DPP IV inhibitor)	dipeptidyl peptidase IV inhibitor	2	II	[14-15],[148-149]
8802	dipeptidyl peptidase IV inhibitor (DPP IV inhibitor)	dipeptidyl peptidase IV inhibitor	2	IL	[5-6],[357-358]
8806	dipeptidyl peptidase IV inhibitor (DPP IV inhibitor)	dipeptidyl peptidase IV inhibitor	3	IR	[140-141],[278-279],[458-459]
8808	dipeptidyl peptidase IV inhibitor (DPP IV inhibitor)	dipeptidyl peptidase IV inhibitor	1	KE	[293-294]
8809	dipeptidyl peptidase IV inhibitor (DPP IV inhibitor)	dipeptidyl peptidase IV inhibitor	2	KF	[33-34],[481-482]
8812	dipeptidyl peptidase IV inhibitor (DPP IV inhibitor)	dipeptidyl peptidase IV inhibitor	1	KI	[139-140]
8814	dipeptidyl peptidase IV inhibitor (DPP IV inhibitor)	dipeptidyl peptidase IV inhibitor	1	KR	[428-429]
8816	dipeptidyl peptidase IV inhibitor (DPP IV inhibitor)	dipeptidyl peptidase IV inhibitor	1	KT	[441-442]
8821	dipeptidyl peptidase IV inhibitor (DPP IV inhibitor)	dipeptidyl peptidase IV inhibitor	1	LI	[13-14]
8823	dipeptidyl peptidase IV inhibitor (DPP IV inhibitor)	dipeptidyl peptidase IV inhibitor	2	LN	[39-40],[382-383]
8824	dipeptidyl peptidase IV inhibitor (DPP IV inhibitor)	dipeptidyl peptidase IV inhibitor	1	LT	[418-419]
8825	dipeptidyl peptidase IV inhibitor (DPP IV inhibitor)	dipeptidyl peptidase IV inhibitor	3	LV	[96-97],[466-467],[489-490]
8837	dipeptidyl peptidase IV inhibitor (DPP IV inhibitor)	dipeptidyl peptidase IV inhibitor	1	MV	[447-448]
8839	dipeptidyl peptidase IV inhibitor (DPP IV inhibitor)	dipeptidyl peptidase IV inhibitor	5	NA	[92-93],[178-179],[383-384],[445-446],[468-469]
8840	dipeptidyl peptidase IV inhibitor (DPP IV inhibitor)	dipeptidyl peptidase IV inhibitor	2	ND	[161-162],[261-262]
8841	dipeptidyl peptidase IV inhibitor (DPP IV inhibitor)	dipeptidyl peptidase IV inhibitor	1	NE	[443-444]
8842	dipeptidyl peptidase IV inhibitor (DPP IV inhibitor)	dipeptidyl peptidase IV inhibitor	3	NF	[188-189],[402-403],[423-424]
8843	dipeptidyl peptidase IV inhibitor (DPP IV inhibitor)	dipeptidyl peptidase IV inhibitor	3	NG	[20-21],[83-84],[316-317]
8844	dipeptidyl peptidase IV inhibitor (DPP IV inhibitor)	dipeptidyl peptidase IV inhibitor	1	NH	[64-65]
8845	dipeptidyl peptidase IV inhibitor (DPP IV inhibitor)	dipeptidyl peptidase IV inhibitor	2	NL	[354-355],[381-382]
8847	dipeptidyl peptidase IV inhibitor (DPP IV inhibitor)	dipeptidyl peptidase IV inhibitor	2	NN	[177-178],[232-233]
8848	dipeptidyl peptidase IV inhibitor (DPP IV inhibitor)	dipeptidyl peptidase IV inhibitor	1	NQ	[180-181]
8849	dipeptidyl peptidase IV inhibitor (DPP IV inhibitor)	dipeptidyl peptidase IV inhibitor	3	NR	[40-41],[49-50],[483-484]
8851	dipeptidyl peptidase IV inhibitor (DPP IV inhibitor)	dipeptidyl peptidase IV inhibitor	2	NV	[233-234],[248-249]
8855	dipeptidyl peptidase IV inhibitor (DPP IV inhibitor)	dipeptidyl peptidase IV inhibitor	1	PG	[111-112]
8856	dipeptidyl peptidase IV inhibitor (DPP IV inhibitor)	dipeptidyl peptidase IV inhibitor	2	PH	[88-89],[378-379]
8857	dipeptidyl peptidase IV inhibitor (DPP IV inhibitor)	dipeptidyl peptidase IV inhibitor	2	PI	[4-5],[356-357]
8860	dipeptidyl peptidase IV inhibitor (DPP IV inhibitor)	dipeptidyl peptidase IV inhibitor	2	PN	[63-64],[82-83]
8861	dipeptidyl peptidase IV inhibitor (DPP IV inhibitor)	dipeptidyl peptidase IV inhibitor	3	PQ	[94-95],[201-202],[421-422]

8862	dipeptidyl peptidase IV inhibitor (DPP IV inhibitor)	dipeptidyl peptidase IV inhibitor	1	PS	[334-335]
8863	dipeptidyl peptidase IV inhibitor (DPP IV inhibitor)	dipeptidyl peptidase IV inhibitor	1	PT	[47-48]
8864	dipeptidyl peptidase IV inhibitor (DPP IV inhibitor)	dipeptidyl peptidase IV inhibitor	1	PV	[166-167]
8868	dipeptidyl peptidase IV inhibitor (DPP IV inhibitor)	dipeptidyl peptidase IV inhibitor	1	QD	[134-135]
8869	dipeptidyl peptidase IV inhibitor (DPP IV inhibitor)	dipeptidyl peptidase IV inhibitor	3	QE	[205-206],[287-288],[485-486]
8871	dipeptidyl peptidase IV inhibitor (DPP IV inhibitor)	dipeptidyl peptidase IV inhibitor	2	QG	[126-127],[202-203]
8872	dipeptidyl peptidase IV inhibitor (DPP IV inhibitor)	dipeptidyl peptidase IV inhibitor	3	QH	[31-32],[209-210],[215-216]
8873	dipeptidyl peptidase IV inhibitor (DPP IV inhibitor)	dipeptidyl peptidase IV inhibitor	1	QI	[471-472]
8874	dipeptidyl peptidase IV inhibitor (DPP IV inhibitor)	dipeptidyl peptidase IV inhibitor	7	QL	[38-39],[67-68],[95-96],[181-182],[274-275],[362-363],[416-417]
8875	dipeptidyl peptidase IV inhibitor (DPP IV inhibitor)	dipeptidyl peptidase IV inhibitor	2	QN	[184-185],[422-423]
8876	dipeptidyl peptidase IV inhibitor (DPP IV inhibitor)	dipeptidyl peptidase IV inhibitor	9	QQ	[30-31],[66-67],[125-126],[133-134],[204-205],[213-214],[214-215],[217-218],[226-227]
8877	dipeptidyl peptidase IV inhibitor (DPP IV inhibitor)	dipeptidyl peptidase IV inhibitor	4	QS	[25-26],[123-124],[258-259],[307-308]
8878	dipeptidyl peptidase IV inhibitor (DPP IV inhibitor)	dipeptidyl peptidase IV inhibitor	1	QT	[405-406]
8879	dipeptidyl peptidase IV inhibitor (DPP IV inhibitor)	dipeptidyl peptidase IV inhibitor	2	QV	[276-277],[398-399]
8882	dipeptidyl peptidase IV inhibitor (DPP IV inhibitor)	dipeptidyl peptidase IV inhibitor	7	RG	[101-102],[103-104],[220-221],[264-265],[310-311],[367-368],[392-393]
8883	dipeptidyl peptidase IV inhibitor (DPP IV inhibitor)	dipeptidyl peptidase IV inhibitor	2	RH	[136-137],[141-142]
8884	dipeptidyl peptidase IV inhibitor (DPP IV inhibitor)	dipeptidyl peptidase IV inhibitor	2	RI	[50-51],[346-347]
8885	dipeptidyl peptidase IV inhibitor (DPP IV inhibitor)	dipeptidyl peptidase IV inhibitor	1	RK	[292-293]
8886	dipeptidyl peptidase IV inhibitor (DPP IV inhibitor)	dipeptidyl peptidase IV inhibitor	3	RL	[41-42],[256-257],[479-480]
8888	dipeptidyl peptidase IV inhibitor (DPP IV inhibitor)	dipeptidyl peptidase IV inhibitor	1	RN	[187-188]
8889	dipeptidyl peptidase IV inhibitor (DPP IV inhibitor)	dipeptidyl peptidase IV inhibitor	9	RR	[77-78],[129-130],[211-212],[219-220],[255-256],[305-306],[309-310],[478-479],[501-502]
8890	dipeptidyl peptidase IV inhibitor (DPP IV inhibitor)	dipeptidyl peptidase IV inhibitor	2	RW	[281-282],[359-360]
8891	dipeptidyl peptidase IV inhibitor (DPP IV inhibitor)	dipeptidyl peptidase IV inhibitor	1	SF	[439-440]
8892	dipeptidyl peptidase IV inhibitor (DPP IV inhibitor)	dipeptidyl peptidase IV inhibitor	1	SH	[352-353]
8893	dipeptidyl peptidase IV inhibitor (DPP IV inhibitor)	dipeptidyl peptidase IV inhibitor	2	SI	[8-9],[266-267]
8895	dipeptidyl peptidase IV inhibitor (DPP IV inhibitor)	dipeptidyl peptidase IV inhibitor	1	SV	[386-387]
8896	dipeptidyl peptidase IV inhibitor (DPP IV inhibitor)	dipeptidyl peptidase IV inhibitor	1	SW	[60-61]
8899	dipeptidyl peptidase IV inhibitor (DPP IV inhibitor)	dipeptidyl peptidase IV inhibitor	2	TE	[251-252],[341-342]
8900	dipeptidyl peptidase IV inhibitor (DPP IV inhibitor)	dipeptidyl peptidase IV inhibitor	1	TF	[116-117]
8901	dipeptidyl peptidase IV inhibitor (DPP IV inhibitor)	dipeptidyl peptidase IV inhibitor	1	TG	[106-107]
8902	dipeptidyl peptidase IV inhibitor (DPP IV inhibitor)	dipeptidyl peptidase IV inhibitor	1	TH	[175-176]
8903	dipeptidyl peptidase IV inhibitor (DPP IV inhibitor)	dipeptidyl peptidase IV inhibitor	3	TI	[79-80],[321-322],[419-420]
8905	dipeptidyl peptidase IV inhibitor (DPP IV inhibitor)	dipeptidyl peptidase IV inhibitor	2	TL	[324-325],[488-489]
8907	dipeptidyl peptidase IV inhibitor (DPP IV inhibitor)	dipeptidyl peptidase IV inhibitor	2	TN	[48-49],[442-443]

8910	dipeptidyl peptidase IV inhibitor (DPP IV inhibitor)	dipeptidyl peptidase IV inhibitor	1	TS	[455-456]
8912	dipeptidyl peptidase IV inhibitor (DPP IV inhibitor)	dipeptidyl peptidase IV inhibitor	2	TV	[349-350],[406-407]
8915	dipeptidyl peptidase IV inhibitor (DPP IV inhibitor)	dipeptidyl peptidase IV inhibitor	2	VD	[249-250],[400-401]
8916	dipeptidyl peptidase IV inhibitor (DPP IV inhibitor)	dipeptidyl peptidase IV inhibitor	1	VE	[270-271]
8917	dipeptidyl peptidase IV inhibitor (DPP IV inhibitor)	dipeptidyl peptidase IV inhibitor	2	VF	[234-235],[407-408]
8920	dipeptidyl peptidase IV inhibitor (DPP IV inhibitor)	dipeptidyl peptidase IV inhibitor	2	VI	[57-58],[277-278]
8921	dipeptidyl peptidase IV inhibitor (DPP IV inhibitor)	dipeptidyl peptidase IV inhibitor	1	VK	[427-428]
8922	dipeptidyl peptidase IV inhibitor (DPP IV inhibitor)	dipeptidyl peptidase IV inhibitor	2	VL	[108-109],[465-466]
8924	dipeptidyl peptidase IV inhibitor (DPP IV inhibitor)	dipeptidyl peptidase IV inhibitor	2	VN	[350-351],[467-468]
8925	dipeptidyl peptidase IV inhibitor (DPP IV inhibitor)	dipeptidyl peptidase IV inhibitor	1	VQ	[397-398]
8926	dipeptidyl peptidase IV inhibitor (DPP IV inhibitor)	dipeptidyl peptidase IV inhibitor	2	VS	[438-439],[448-449]
8929	dipeptidyl peptidase IV inhibitor (DPP IV inhibitor)	dipeptidyl peptidase IV inhibitor	2	VY	[97-98],[388-389]
8930	dipeptidyl peptidase IV inhibitor (DPP IV inhibitor)	dipeptidyl peptidase IV inhibitor	1	WD	[61-62]
8932	dipeptidyl peptidase IV inhibitor (DPP IV inhibitor)	dipeptidyl peptidase IV inhibitor	1	YA	[389-390]
8934	dipeptidyl peptidase IV inhibitor (DPP IV inhibitor)	dipeptidyl peptidase IV inhibitor	1	YE	[207-208]
8938	dipeptidyl peptidase IV inhibitor (DPP IV inhibitor)	dipeptidyl peptidase IV inhibitor	1	YI	[98-99]
8940	dipeptidyl peptidase IV inhibitor (DPP IV inhibitor)	dipeptidyl peptidase IV inhibitor	2	YL	[10-11],[190-191]
8942	dipeptidyl peptidase IV inhibitor (DPP IV inhibitor)	dipeptidyl peptidase IV inhibitor	1	YN	[160-161]
8945	dipeptidyl peptidase IV inhibitor (DPP IV inhibitor)	dipeptidyl peptidase IV inhibitor	2	YS	[90-91],[371-372]
8946	dipeptidyl peptidase IV inhibitor (DPP IV inhibitor)	dipeptidyl peptidase IV inhibitor	1	YV	[376-377]
9477	DPP-III inhibitor	dipeptidyl peptidase III inhibitor	2	RW	[281-282],[359-360]
9478	DPP-III inhibitor	dipeptidyl peptidase III inhibitor	4	LR	[327-328],[358-359],[391-392],[412-413]
9482	DPP-III inhibitor	dipeptidyl peptidase III inhibitor	2	YL	[10-11],[190-191]
9485	DPP-III inhibitor	dipeptidyl peptidase III inhibitor	9	RR	[77-78],[129-130],[211-212],[219-220],[255-256],[305-306],[309-310],[478-479],[501-502]
9486	DPP-III inhibitor	dipeptidyl peptidase III inhibitor	1	TF	[116-117]
9487	DPP-III inhibitor	dipeptidyl peptidase III inhibitor	2	GE	[221-222],[224-225]
9488	DPP-III inhibitor	dipeptidyl peptidase III inhibitor	2	GF	[237-238],[434-435]
9489	DPP-III inhibitor	dipeptidyl peptidase III inhibitor	3	PR	[186-187],[280-281],[473-474]
9491	DPP-III inhibitor	dipeptidyl peptidase III inhibitor	1	RV	[269-270]
9492	DPP-III inhibitor	dipeptidyl peptidase III inhibitor	5	DA	[43-44],[239-240],[245-246],[373-374],[476-477]
9494	DPP-III inhibitor	dipeptidyl peptidase III inhibitor	1	HK	[32-33]
9495	DPP-III inhibitor	dipeptidyl peptidase III inhibitor	1	HF	[142-143]
9499	DPP-III inhibitor	dipeptidyl peptidase III inhibitor	4	LA	[23-24],[191-192],[243-244],[451-452]
9500	DPP-III inhibitor	dipeptidyl peptidase III inhibitor	1	FA	[424-425]
9501	DPP-III inhibitor	dipeptidyl peptidase III inhibitor	2	FR	[143-144],[199-200]

9502	DPP-III inhibitor	dipeptidyl peptidase III inhibitor	2	FL	[171-172],[242-243]
9504	DPP-III inhibitor	dipeptidyl peptidase III inhibitor	2	PE	[114-115],[462-463]
9509	DPP-III inhibitor	dipeptidyl peptidase III inhibitor	2	VY	[97-98],[388-389]
9510	DPP-III inhibitor	dipeptidyl peptidase III inhibitor	1	YI	[98-99]
8247	CaMPDE inhibitor	CaMPDE inhibitor	3	IR	[140-141],[278-279],[458-459]
8249	CaMPDE inhibitor	CaMPDE inhibitor	2	KF	[33-34],[481-482]
8250	CaMPDE inhibitor	CaMPDE inhibitor	3	EF	[131-132],[198-199],[241-242]
2842	Renin inhibitor	renin inhibitor	4	LR	[327-328],[358-359],[391-392],[412-413]
8246	renin inhibitor	renin inhibitor	3	IR	[140-141],[278-279],[458-459]
8248	Renin inhibitor	renin inhibitor	2	KF	[33-34],[481-482]
8251	Renin inhibitor	renin inhibitor	3	EF	[131-132],[198-199],[241-242]
9430	Renin inhibitor	renin inhibitor	3	NR	[40-41],[49-50],[483-484]
9432	Renin inhibitor	renin inhibitor	1	SF	[439-440]
9433	Renin inhibitor	renin inhibitor	1	YA	[389-390]
9469	Renin inhibitor	renin inhibitor	1	RALP	[459-462]
9470	Renin inhibitor	renin inhibitor	2	LY	[370-371],[375-376]
9471	Renin inhibitor	renin inhibitor	1	TF	[116-117]

Table S13. Profile of potential biological activity of fragments of protein Jug r 1.0101.

ID	Name of peptide	Activity	Number	Sequence	Location
3258	beta-lactokinin	ACE inhibitor	1	IR	[134-135]
3515	ACE inhibitor	ACE inhibitor	1	GGY	[63-65]
3532	ACE inhibitor	ACE inhibitor	1	GY	[64-65]
3537	ACE inhibitor	ACE inhibitor	1	PR	[32-33]
3551	ACE inhibitor (from bovine beta-Lg)	ACE inhibitor	1	LF	[8-9]
7542	ACE inhibitor	ACE inhibitor	1	DLP	[120-122]
7583	ACE inhibitor	ACE inhibitor	1	AF	[15-16]
7590	ACE inhibitor	ACE inhibitor	3	AA	[1-2],[13-14],[14-15]
7592	ACE inhibitor	ACE inhibitor	2	FR	[16-17],[74-75]
7596	ACE inhibitor	ACE inhibitor	1	GI	[126-127]
7599	ACE inhibitor	ACE inhibitor	2	GL	[92-93],[105-106]
7615	ACE inhibitor	ACE inhibitor	2	GE	[36-37],[108-109]
7616	ACE inhibitor	ACE inhibitor	1	GG	[63-64]
7617	ACE inhibitor	ACE inhibitor	1	QG	[104-105]
7618	ACE inhibitor	ACE inhibitor	1	SG	[62-63]
7622	ACE inhibitor	ACE inhibitor	2	EG	[37-38],[91-92]
7628	ACE inhibitor from k-CN (fr. 67-68)	ACE inhibitor	1	VR	[97-98]
7644	ACE inhibitor from porcine myosin (306-308)	ACE inhibitor	1	ITT	[20-22]
7741	ACE inhibitor	ACE inhibitor	4	RR	[33-34],[34-35],[98-99],[135-136]
7742	ACE inhibitor	ACE inhibitor	1	AR	[118-119]
7807	ACE inhibitor from caprine b-Lg	ACE inhibitor	1	LLF	[7-9]
7826	ACE inhibitor	ACE inhibitor	2	EI	[24-25],[133-134]
7832	ACE inhibitor	ACE inhibitor	1	LN	[49-50]
7839	ACE inhibitor	ACE inhibitor	2	ME	[23-24],[111-112]
9173	ACE inhibitor	ACE inhibitor	2	RG	[35-36],[107-108]
9213	ACE inhibitor	ACE inhibitor	3	LR	[56-57],[93-94],[106-107]
9309	ACE inhibitor	ACE inhibitor	1	RRR	[33-35]
9729	ACE inhibitor	ACE inhibitor	1	VVR	[96-98]
9730	ACE inhibitor	ACE inhibitor	1	NPR	[31-33]
8321	Glucose uptake stimulating peptide	stimulating	1	LV	[4-5]
8326	Glucose uptake stimulating peptide	stimulating	2	LL	[3-4],[7-8]
8329	Stimulating vasoactive substance release	stimulating	2	EE	[109-110],[112-113]
8310	Anxiolytic peptide	neuropeptide	1	YL	[55-56]

7941	synthetic peptide	antioxidative	1	YYL	[54-56]
7966	synthetic peptide	antioxidative	1	QYY	[53-55]
8215	Antioxidative peptide	antioxidative	1	IR	[134-135]
9357	Antioxidative peptide	antioxidative	1	CQC	[88-90]
9368	Antioxidative peptide	antioxidative	1	EQC	[86-88]
3172	dipeptidyl peptidase IV inhibitor (DPP IV inhibitor)	dipeptidyl peptidase IV inhibitor	2	VA	[5-6],[10-11]
3180	dipeptidyl peptidase IV inhibitor (DPP IV inhibitor)	dipeptidyl peptidase IV inhibitor	1	LP	[121-122]
3182	dipeptidyl peptidase IV inhibitor (DPP IV inhibitor)	dipeptidyl peptidase IV inhibitor	2	LL	[3-4],[7-8]
3183	dipeptidyl peptidase IV inhibitor (DPP IV inhibitor)	dipeptidyl peptidase IV inhibitor	1	VV	[96-97]
8530	dipeptidyl peptidase IV inhibitor (DPP IV inhibitor)	dipeptidyl peptidase IV inhibitor	1	NP	[31-32]
8559	dipeptidyl peptidase IV inhibitor (DPP IV inhibitor)	dipeptidyl peptidase IV inhibitor	2	AL	[2-3],[6-7]
8561	dipeptidyl peptidase IV inhibitor (DPP IV inhibitor)	dipeptidyl peptidase IV inhibitor	2	GL	[92-93],[105-106]
8594	dipeptidyl peptidase IV inhibitor (DPP IV inhibitor)	dipeptidyl peptidase IV inhibitor	1	VR	[97-98]
8637	dipeptidyl peptidase IV inhibitor (DPP IV inhibitor)	dipeptidyl peptidase IV inhibitor	3	AA	[1-2],[13-14],[14-15]
8692	dipeptidyl peptidase IV inhibitor (DPP IV inhibitor)	dipeptidyl peptidase IV inhibitor	1	WF	[138-139]
8759	dipeptidyl peptidase IV inhibitor (DPP IV inhibitor)	dipeptidyl peptidase IV inhibitor	1	AF	[15-16]
8766	dipeptidyl peptidase IV inhibitor (DPP IV inhibitor)	dipeptidyl peptidase IV inhibitor	2	DN	[30-31],[68-69]
8770	dipeptidyl peptidase IV inhibitor (DPP IV inhibitor)	dipeptidyl peptidase IV inhibitor	2	EG	[37-38],[91-92]
8772	dipeptidyl peptidase IV inhibitor (DPP IV inhibitor)	dipeptidyl peptidase IV inhibitor	2	EI	[24-25],[133-134]
8780	dipeptidyl peptidase IV inhibitor (DPP IV inhibitor)	dipeptidyl peptidase IV inhibitor	2	FR	[16-17],[74-75]
8781	dipeptidyl peptidase IV inhibitor (DPP IV inhibitor)	dipeptidyl peptidase IV inhibitor	2	GE	[36-37],[108-109]
8783	dipeptidyl peptidase IV inhibitor (DPP IV inhibitor)	dipeptidyl peptidase IV inhibitor	1	GG	[63-64]
8785	dipeptidyl peptidase IV inhibitor (DPP IV inhibitor)	dipeptidyl peptidase IV inhibitor	1	GI	[126-127]
8788	dipeptidyl peptidase IV inhibitor (DPP IV inhibitor)	dipeptidyl peptidase IV inhibitor	1	GY	[64-65]
8791	dipeptidyl peptidase IV inhibitor (DPP IV inhibitor)	dipeptidyl peptidase IV inhibitor	1	HF	[73-74]
8805	dipeptidyl peptidase IV inhibitor (DPP IV inhibitor)	dipeptidyl peptidase IV inhibitor	1	IQ	[43-44]
8806	dipeptidyl peptidase IV inhibitor (DPP IV inhibitor)	dipeptidyl peptidase IV inhibitor	1	IR	[134-135]
8823	dipeptidyl peptidase IV inhibitor (DPP IV inhibitor)	dipeptidyl peptidase IV inhibitor	1	LN	[49-50]
8825	dipeptidyl peptidase IV inhibitor (DPP IV inhibitor)	dipeptidyl peptidase IV inhibitor	1	LV	[4-5]
8826	dipeptidyl peptidase IV inhibitor (DPP IV inhibitor)	dipeptidyl peptidase IV inhibitor	2	ME	[23-24],[111-112]
8837	dipeptidyl peptidase IV inhibitor (DPP IV inhibitor)	dipeptidyl peptidase IV inhibitor	1	MV	[114-115]
8839	dipeptidyl peptidase IV inhibitor (DPP IV inhibitor)	dipeptidyl peptidase IV inhibitor	1	NA	[12-13]
8841	dipeptidyl peptidase IV inhibitor (DPP IV inhibitor)	dipeptidyl peptidase IV inhibitor	1	NE	[123-124]
8844	dipeptidyl peptidase IV inhibitor (DPP IV inhibitor)	dipeptidyl peptidase IV inhibitor	1	NH	[50-51]
8845	dipeptidyl peptidase IV inhibitor (DPP IV inhibitor)	dipeptidyl peptidase IV inhibitor	1	NL	[48-49]
8848	dipeptidyl peptidase IV inhibitor (DPP IV inhibitor)	dipeptidyl peptidase IV inhibitor	1	NQ	[69-70]

8860	dipeptidyl peptidase IV inhibitor (DPP IV inhibitor)	dipeptidyl peptidase IV inhibitor	1	PN	[122-123]
8871	dipeptidyl peptidase IV inhibitor (DPP IV inhibitor)	dipeptidyl peptidase IV inhibitor	1	QG	[104-105]
8872	dipeptidyl peptidase IV inhibitor (DPP IV inhibitor)	dipeptidyl peptidase IV inhibitor	1	QH	[72-73]
8873	dipeptidyl peptidase IV inhibitor (DPP IV inhibitor)	dipeptidyl peptidase IV inhibitor	1	QI	[42-43]
8874	dipeptidyl peptidase IV inhibitor (DPP IV inhibitor)	dipeptidyl peptidase IV inhibitor	1	QL	[80-81]
8875	dipeptidyl peptidase IV inhibitor (DPP IV inhibitor)	dipeptidyl peptidase IV inhibitor	1	QN	[47-48]
8876	dipeptidyl peptidase IV inhibitor (DPP IV inhibitor)	dipeptidyl peptidase IV inhibitor	7	QQ	[46-47],[58-59],[79-80],[100-101],[101-102],[102-103],[103-104]
8877	dipeptidyl peptidase IV inhibitor (DPP IV inhibitor)	dipeptidyl peptidase IV inhibitor	2	QS	[59-60],[116-117]
8879	dipeptidyl peptidase IV inhibitor (DPP IV inhibitor)	dipeptidyl peptidase IV inhibitor	1	QV	[95-96]
8881	dipeptidyl peptidase IV inhibitor (DPP IV inhibitor)	dipeptidyl peptidase IV inhibitor	1	QY	[53-54]
8882	dipeptidyl peptidase IV inhibitor (DPP IV inhibitor)	dipeptidyl peptidase IV inhibitor	2	RG	[35-36],[107-108]
8889	dipeptidyl peptidase IV inhibitor (DPP IV inhibitor)	dipeptidyl peptidase IV inhibitor	4	RR	[33-34],[34-35],[98-99],[135-136]
8896	dipeptidyl peptidase IV inhibitor (DPP IV inhibitor)	dipeptidyl peptidase IV inhibitor	1	SW	[137-138]
8903	dipeptidyl peptidase IV inhibitor (DPP IV inhibitor)	dipeptidyl peptidase IV inhibitor	1	TI	[19-20]
8906	dipeptidyl peptidase IV inhibitor (DPP IV inhibitor)	dipeptidyl peptidase IV inhibitor	1	TM	[22-23]
8911	dipeptidyl peptidase IV inhibitor (DPP IV inhibitor)	dipeptidyl peptidase IV inhibitor	2	TT	[18-19],[21-22]
8925	dipeptidyl peptidase IV inhibitor (DPP IV inhibitor)	dipeptidyl peptidase IV inhibitor	1	VQ	[115-116]
8933	dipeptidyl peptidase IV inhibitor (DPP IV inhibitor)	dipeptidyl peptidase IV inhibitor	1	YD	[65-66]
8940	dipeptidyl peptidase IV inhibitor (DPP IV inhibitor)	dipeptidyl peptidase IV inhibitor	1	YL	[55-56]
8948	dipeptidyl peptidase IV inhibitor (DPP IV inhibitor)	dipeptidyl peptidase IV inhibitor	1	YY	[54-55]
9476	DPP-III inhibitor	dipeptidyl peptidase III inhibitor	1	YY	[54-55]
9478	DPP-III inhibitor	dipeptidyl peptidase III inhibitor	3	LR	[56-57],[93-94],[106-107]
9482	DPP-III inhibitor	dipeptidyl peptidase III inhibitor	1	YL	[55-56]
9485	DPP-III inhibitor	dipeptidyl peptidase III inhibitor	4	RR	[33-34],[34-35],[98-99],[135-136]
9487	DPP-III inhibitor	dipeptidyl peptidase III inhibitor	2	GE	[36-37],[108-109]
9489	DPP-III inhibitor	dipeptidyl peptidase III inhibitor	1	PR	[32-33]
9495	DPP-III inhibitor	dipeptidyl peptidase III inhibitor	1	HF	[73-74]
9501	DPP-III inhibitor	dipeptidyl peptidase III inhibitor	2	FR	[16-17],[74-75]
8247	CaMPDE inhibitor	CaMPDE inhibitor	1	IR	[134-135]
2842	Renin inhibitor	renin inhibitor	3	LR	[56-57],[93-94],[106-107]
8246	renin inhibitor	renin inhibitor	1	IR	[134-135]

Table S14. Profile of potential biological activity of fragments of protein Jug r 2.0101.

ID	Name of peptide	Activity	Number	Sequence	Location
3460	Prolyl endopeptidase inhibitor	antiamnestic	1	PG	[302-303]
3461	Prolyl endopeptidase inhibitor	antiamnestic	2	GP	[160-161],[384-385]
3257	beta-lactokinin	ACE inhibitor	5	RL	[45-46],[289-290],[339-340],[487-488],[513-514]
3258	beta-lactokinin	ACE inhibitor	4	IR	[40-41],[189-190],[271-272],[355-356]
3380	ACE inhibitor	ACE inhibitor	2	RY	[75-76],[451-452]
3384	ACE inhibitor	ACE inhibitor	1	VF	[323-324]
3489	ACE inhibitor from sake lees	ACE inhibitor	3	RF	[205-206],[342-343],[480-481]
3492	ACE inhibitor from sake	ACE inhibitor	2	VY	[279-280],[443-444]
3494	ACE inhibitor from sake	ACE inhibitor	1	HY	[434-435]
3507	ACE inhibitor (beta-LG fr. 78-80)	ACE inhibitor	1	IPA	[496-498]
3518	ACE inhibitor	ACE inhibitor	1	VAA	[330-332]
3537	ACE inhibitor	ACE inhibitor	6	PR	[11-12],[14-15],[69-70],[165-166],[336-337],[551-552]
3542	ACE inhibitor	ACE inhibitor	1	LQP	[296-298]
3547	ACE inhibitor	ACE inhibitor	1	IRA	[355-357]
7498	ACE inhibitor from as2-CN	ACE inhibitor	1	TVY	[278-280]
7512	ACE inhibitor from Alaskan pollack skin	ACE inhibitor	2	GP	[160-161],[384-385]
7513	ACE inhibitor from Alaskan pollack skin	ACE inhibitor	1	PL	[583-584]
7558	ACE inhibitor from buckwheat	ACE inhibitor	2	VK	[200-201],[293-294]
7559	ACE inhibitor from buckwheat	ACE inhibitor	1	PSY	[393-395]
7562	ACE inhibitor from soy hydrolysate	ACE inhibitor	1	IA	[502-503]
7581	ACE inhibitor	ACE inhibitor	1	IP	[496-497]
7582	ACE inhibitor	ACE inhibitor	1	RP	[375-376]
7583	ACE inhibitor	ACE inhibitor	1	AF	[591-592]
7585	ACE inhibitor	ACE inhibitor	3	LA	[488-489],[529-530],[584-585]
7586	ACE inhibitor	ACE inhibitor	1	KR	[426-427]
7587	ACE inhibitor	ACE inhibitor	3	VP	[273-274],[432-433],[568-569]
7588	ACE inhibitor	ACE inhibitor	3	RA	[249-250],[356-357],[363-364]
7589	ACE inhibitor	ACE inhibitor	2	YA	[309-310],[422-423]
7590	ACE inhibitor	ACE inhibitor	2	AA	[310-311],[331-332]

7591	ACE inhibitor	ACE inhibitor	1	GF	[516-517]
7592	ACE inhibitor	ACE inhibitor	1	FR	[305-306]
7593	ACE inhibitor	ACE inhibitor	2	IF	[493-494],[558-559]
7596	ACE inhibitor	ACE inhibitor	1	GI	[215-216]
7598	ACE inhibitor	ACE inhibitor	3	GA	[276-277],[312-313],[428-429]
7600	ACE inhibitor	ACE inhibitor	5	AG	[275-276],[311-312],[372-373],[498-499],[530-531]
7601	ACE inhibitor	ACE inhibitor	1	GH	[499-500]
7603	ACE inhibitor	ACE inhibitor	11	GR	[2-3],[61-62],[113-114],[156-157],[171-172],[248-249],[378-379],[450-451],[468-469],[479-480],[579-580]
7605	ACE inhibitor	ACE inhibitor	1	FG	[399-400]
7606	ACE inhibitor	ACE inhibitor	2	DA	[225-226],[238-239]
7608	ACE inhibitor	ACE inhibitor	1	GV	[352-353]
7610	ACE inhibitor	ACE inhibitor	9	GQ	[29-30],[31-32],[89-90],[303-304],[373-374],[400-401],[466-467],[531-532],[577-578]
7612	ACE inhibitor	ACE inhibitor	1	GT	[448-449]
7613	ACE inhibitor	ACE inhibitor	1	WG	[377-378]
7615	ACE inhibitor	ACE inhibitor	2	GE	[198-199],[521-522]
7616	ACE inhibitor	ACE inhibitor	1	GG	[383-384]
7617	ACE inhibitor	ACE inhibitor	5	QG	[28-29],[30-31],[155-156],[467-468],[578-579]
7618	ACE inhibitor	ACE inhibitor	1	SG	[382-383]
7619	ACE inhibitor	ACE inhibitor	1	LG	[515-516]
7620	ACE inhibitor	ACE inhibitor	2	GD	[268-269],[491-492]
7621	ACE inhibitor	ACE inhibitor	2	TG	[449-450],[478-479]
7622	ACE inhibitor	ACE inhibitor	4	EG	[197-198],[351-352],[447-448],[465-466]
7623	ACE inhibitor	ACE inhibitor	3	EA	[162-163],[404-405],[542-543]
7624	ACE inhibitor	ACE inhibitor	1	NG	[520-521]
7625	ACE inhibitor	ACE inhibitor	1	PG	[302-303]
7635	ACE inhibitor from k-CN (fr. 51-53)	ACE inhibitor	1	VAV	[242-244]
7680	ACE inhibitor from pea vicilin	ACE inhibitor	1	QK	[482-483]
7682	ACE inhibitor from garlic	ACE inhibitor	2	NY	[218-219],[421-422]
7684	ACE inhibitor from garlic	ACE inhibitor	4	SY	[319-320],[394-395],[463-464],[565-566]
7685	ACE inhibitor from garlic	ACE inhibitor	2	SF	[262-263],[547-548]
7691	ACE inhibitor from wakame	ACE inhibitor	1	KY	[201-202]

7693	ACE inhibitor from wakame	ACE inhibitor	2	KL	[294-295],[361-362]
7697	ACE inhibitor from wakame	ACE inhibitor	1	YK	[151-152]
7741	ACE inhibitor	ACE inhibitor	12	RR	[26-27],[58-59],[59-60],[87-88],[96-97],[97-98],[102-103],[128-129],[166-167],[379-380],[469-470],[575-576]
7742	ACE inhibitor	ACE inhibitor	2	AR	[486-487],[489-490]
7743	ACE inhibitor	ACE inhibitor	1	KA	[438-439]
7752	ACE inhibitor from shark meat hydrolysate	ACE inhibitor	2	EY	[23-24],[307-308]
7826	ACE inhibitor	ACE inhibitor	3	EI	[424-425],[554-555],[557-558]
7827	ACE inhibitor	ACE inhibitor	2	IE	[216-217],[555-556]
7828	ACE inhibitor	ACE inhibitor	1	EV	[199-200]
7829	ACE inhibitor	ACE inhibitor	1	VE	[446-447]
7830	ACE inhibitor	ACE inhibitor	3	TE	[207-208],[210-211],[570-571]
7831	ACE inhibitor	ACE inhibitor	2	LQ	[296-297],[413-414]
7832	ACE inhibitor	ACE inhibitor	1	LN	[333-334]
7833	ACE inhibitor	ACE inhibitor	1	PT	[569-570]
7837	ACE inhibitor	ACE inhibitor	1	PQ	[119-120]
7839	ACE inhibitor	ACE inhibitor	1	ME	[563-564]
7840	ACE inhibitor	ACE inhibitor	1	EK	[360-361]
7841	ACE inhibitor	ACE inhibitor	2	KE	[152-153],[544-545]
7842	ACE inhibitor	ACE inhibitor	2	HP	[500-501],[582-583]
7843	ACE inhibitor	ACE inhibitor	3	PH	[234-235],[433-434],[457-458]
7844	ACE inhibitor	ACE inhibitor	1	HK	[236-237]
8193	ACE inhibitor	ACE inhibitor	1	AI	[503-504]
8513	ACE inhibitor from soya milk	ACE inhibitor	1	FVP	[567-569]
8951	ACE inhibitor	ACE inhibitor	1	AV	[243-244]
9031	ACE inhibitor	ACE inhibitor	1	LEE	[46-48]
9068	ACE inhibitor	ACE inhibitor	1	VTR	[245-247]
9073	ACE inhibitor	ACE inhibitor	1	TP	[335-336]
9074	ACE inhibitor	ACE inhibitor	2	DF	[527-528],[589-590]
9076	ACE inhibitor	ACE inhibitor	1	FQ	[481-482]
9077	ACE inhibitor	ACE inhibitor	2	YV	[280-281],[444-445]
9078	ACE inhibitor	ACE inhibitor	3	YE	[76-77],[452-453],[464-465]

9079	ACE inhibitor	ACE inhibitor	3	IL	[223-224],[328-329],[587-588]
9085	ACE inhibitor	ACE inhibitor	1	MM	[430-431]
9087	ACE inhibitor	ACE inhibitor	1	YH	[123-124]
9173	ACE inhibitor	ACE inhibitor	11	RG	[1-2],[60-61],[88-89],[112-113],[159-160],[170-171],[214-215],[247-248],[427-428],[490-491],[576-577]
9183	ACE inhibitor	ACE inhibitor	1	GTG	[448-450]
9184	ACE inhibitor	ACE inhibitor	1	ST	[477-478]
9185	ACE inhibitor	ACE inhibitor	1	YN	[435-436]
9196	ACE inhibitor	ACE inhibitor	1	AVV	[243-245]
9213	ACE inhibitor	ACE inhibitor	4	LR	[213-214],[321-322],[362-363],[512-513]
9309	ACE inhibitor	ACE inhibitor	2	RRR	[58-60],[96-98]
9566	ACE inhibitor	ACE inhibitor	1	QP	[297-298]
9730	ACE inhibitor	ACE inhibitor	2	NPR	[10-12],[68-70]
9743	ACE inhibitor	ACE inhibitor	3	EQR	[73-75],[100-102],[348-350]
9754	ACE inhibitor	ACE inhibitor	1	NLR	[511-513]
3283	Antithrombotic peptide	antithrombotic	2	GP	[160-161],[384-385]
3285	Antithrombotic peptide	antithrombotic	1	PG	[302-303]
3354	Antithrombotic peptide	antithrombotic	1	DEE	[7-9]
9660	Antithrombotic peptide	antithrombotic	1	RGD	[490-492]
3351	Stimulating vasoactive substance release	stimulating	3	EEE	[173-175],[473-475],[474-476]
8320	Glucose uptake stimulating peptide	stimulating	1	VL	[418-419]
8321	Glucose uptake stimulating peptide	stimulating	3	LV	[254-255],[329-330],[419-420]
8323	Glucose uptake stimulating peptide	stimulating	3	IL	[223-224],[328-329],[587-588]
8325	Glucose uptake stimulating peptide	stimulating	2	II	[354-355],[535-536]
8326	Glucose uptake stimulating peptide	stimulating	3	LL	[212-213],[295-296],[514-515]
8329	Stimulating vasoactive substance release	stimulating	14	EE	[8-9],[43-44],[47-48],[54-55],[106-107],[173-174],[174-175],[196-197],[408-409],[473-474],[474-475],[475-476],[553-554],[556-557]
8330	Stimulating vasoactive substance release	stimulating	2	SE	[195-196],[390-391]
2890	neuropeptide	neuropeptide	9	GQ	[29-30],[31-32],[89-90],[303-304],[373-374],[400-401],[466-467],[531-532],[577-578]
8310	Anxiolytic peptide	neuropeptide	2	YL	[202-203],[320-321]
9534	Kyotorphin	neuropeptide	2	YR	[18-19],[219-220]
2753	peptide regulating the stomach mucosal membrane activity	regulating	2	GP	[160-161],[384-385]

2754	peptide regulating the stomach mucosal membrane activity	regulating	1	PG	[302-303]
8318	Dvl protein binding	anticancer	1	VVV	[441-443]
3300		antioxidative	1	PHH	[234-236]
3307		antioxidative	1	PYY	[181-183]
3318		antioxidative	1	LPHH	[233-236]
3319		antioxidative	1	HH	[235-236]
7888	antioxidative peptide	antioxidative	2	EL	[211-212],[545-546]
7927	synthetic peptide	antioxidative	1	EYY	[307-309]
7939	synthetic peptide	antioxidative	1	YYA	[308-310]
7943	synthetic peptide	antioxidative	1	YYF	[182-184]
8036	synthetic peptide	antioxidative	1	PHV	[457-459]
8038	synthetic peptide	antioxidative	1	PHY	[433-435]
8042	synthetic peptide	antioxidative	1	PWG	[376-378]
8056	synthetic peptide	antioxidative	1	RHE	[192-194]
8063	synthetic peptide	antioxidative	1	RHN	[178-180]
8103	peptide derived from dried bonito	antioxidative	1	VKL	[293-295]
8130	peptide derived from dried bonito	antioxidative	1	EAK	[542-544]
8134	peptide derived from dried bonito	antioxidative	1	KD	[237-238]
8190	peptide from buckwheat	antioxidative	1	PW	[376-377]
8215	Antioxidative peptide	antioxidative	4	IR	[40-41],[189-190],[271-272],[355-356]
8217	Antioxidative peptide	antioxidative	1	LK	[388-389]
8224	antioxidative peptide	antioxidative	2	VY	[279-280],[443-444]
8484	Antioxidant peptide from as1-CN (98-100)	antioxidative	1	LLR	[212-214]
9082	Antioxidative peptide	antioxidative	1	WG	[377-378]
9086	Antioxidative peptide	antioxidative	1	MM	[430-431]
9361	Antioxidative peptide	antioxidative	2	VYV	[279-281],[443-445]
9363	Antioxidative peptide	antioxidative	1	NEN	[509-511]
9366	Antioxidative peptide	antioxidative	1	KYL	[201-203]
9368	Antioxidative peptide	antioxidative	1	EQC	[77-79]
3164	laminin-like peptide	embryotoxic	1	RGD	[490-492]
4005		activating ubiquitin-mediated proteolysis	3	RA	[249-250],[356-357],[363-364]

4006	Ubiqitin-mediated proteolysis activating peptide	activating ubiquitin-mediated proteolysis	3	LA	[488-489],[529-530],[584-585]
9650	Alpha-glucosidase inhibitor	alpha-glucosidase inhibitor	3	EA	[162-163],[404-405],[542-543]
9693	Alpha-glucosidase inhibitor	alpha-glucosidase inhibitor	1	VE	[446-447]
9694	Alpha-glucosidase inhibitor	alpha-glucosidase inhibitor	4	PE	[72-73],[137-138],[161-162],[407-408]
3169	dipeptidyl peptidase IV inhibitor (DPP IV inhibitor)	dipeptidyl peptidase IV inhibitor	2	GP	[160-161],[384-385]
3171	dipeptidyl peptidase IV inhibitor (DPP IV inhibitor)	dipeptidyl peptidase IV inhibitor	1	MP	[550-551]
3172	dipeptidyl peptidase IV inhibitor (DPP IV inhibitor)	dipeptidyl peptidase IV inhibitor	2	VA	[242-243],[330-331]
3173	dipeptidyl peptidase IV inhibitor (DPP IV inhibitor)	dipeptidyl peptidase IV inhibitor	1	MA	[454-455]
3174	dipeptidyl peptidase IV inhibitor (DPP IV inhibitor)	dipeptidyl peptidase IV inhibitor	1	KA	[438-439]
3175	dipeptidyl peptidase IV inhibitor (DPP IV inhibitor)	dipeptidyl peptidase IV inhibitor	3	LA	[488-489],[529-530],[584-585]
3176	dipeptidyl peptidase IV inhibitor (DPP IV inhibitor)	dipeptidyl peptidase IV inhibitor	1	FA	[590-591]
3179	dipeptidyl peptidase IV inhibitor (DPP IV inhibitor)	dipeptidyl peptidase IV inhibitor	2	PA	[274-275],[497-498]
3180	dipeptidyl peptidase IV inhibitor (DPP IV inhibitor)	dipeptidyl peptidase IV inhibitor	1	LP	[233-234]
3181	dipeptidyl peptidase IV inhibitor (DPP IV inhibitor)	dipeptidyl peptidase IV inhibitor	3	VP	[273-274],[432-433],[568-569]
3182	dipeptidyl peptidase IV inhibitor (DPP IV inhibitor)	dipeptidyl peptidase IV inhibitor	3	LL	[212-213],[295-296],[514-515]
3183	dipeptidyl peptidase IV inhibitor (DPP IV inhibitor)	dipeptidyl peptidase IV inhibitor	5	VV	[221-222],[244-245],[441-442],[442-443],[445-446]
3184	dipeptidyl peptidase IV inhibitor (DPP IV inhibitor)	dipeptidyl peptidase IV inhibitor	1	HA	[368-369]
8304	Dipeptidyl peptidase IV inhibitor (DPP IV inhibitor)	dipeptidyl peptidase IV inhibitor	1	IPA	[496-498]
8501	Dipeptidyl peptidase IV inhibitor (DPP IV inhibitor)	dipeptidyl peptidase IV inhibitor	1	IP	[496-497]
8503	Dipeptidyl peptidase IV inhibitor (DPP IV inhibitor)	dipeptidyl peptidase IV inhibitor	1	TP	[335-336]

8505	Dipeptidyl peptidase IV inhibitor (DPP IV inhibitor)	dipeptidyl peptidase IV inhibitor	4	SP	[136-137],[164-165],[315-316],[392-393]
8518	dipeptidyl peptidase IV inhibitor (DPP IV inhibitor)	dipeptidyl peptidase IV inhibitor	1	RP	[375-376]
8520	dipeptidyl peptidase IV inhibitor (DPP IV inhibitor)	dipeptidyl peptidase IV inhibitor	2	HP	[500-501],[582-583]
8524	dipeptidyl peptidase IV inhibitor (DPP IV inhibitor)	dipeptidyl peptidase IV inhibitor	3	GA	[276-277],[312-313],[428-429]
8525	dipeptidyl peptidase IV inhibitor (DPP IV inhibitor)	dipeptidyl peptidase IV inhibitor	1	IA	[502-503]
8526	dipeptidyl peptidase IV inhibitor (DPP IV inhibitor)	dipeptidyl peptidase IV inhibitor	3	RA	[249-250],[356-357],[363-364]
8530	dipeptidyl peptidase IV inhibitor (DPP IV inhibitor)	dipeptidyl peptidase IV inhibitor	5	NP	[10-11],[68-69],[180-181],[227-228],[301-302]
8531	dipeptidyl peptidase IV inhibitor (DPP IV inhibitor)	dipeptidyl peptidase IV inhibitor	2	TA	[485-486],[505-506]
8532	dipeptidyl peptidase IV inhibitor (DPP IV inhibitor)	dipeptidyl peptidase IV inhibitor	1	QP	[297-298]
8555	dipeptidyl peptidase IV inhibitor (DPP IV inhibitor)	dipeptidyl peptidase IV inhibitor	1	FL	[528-529]
8558	dipeptidyl peptidase IV inhibitor (DPP IV inhibitor)	dipeptidyl peptidase IV inhibitor	1	EK	[360-361]
8559	dipeptidyl peptidase IV inhibitor (DPP IV inhibitor)	dipeptidyl peptidase IV inhibitor	2	AL	[332-333],[364-365]
8560	dipeptidyl peptidase IV inhibitor (DPP IV inhibitor)	dipeptidyl peptidase IV inhibitor	1	SL	[387-388]
8637	dipeptidyl peptidase IV inhibitor (DPP IV inhibitor)	dipeptidyl peptidase IV inhibitor	2	AA	[310-311],[331-332]
8638	dipeptidyl peptidase IV inhibitor (DPP IV inhibitor)	dipeptidyl peptidase IV inhibitor	1	PL	[583-584]
8689	dipeptidyl peptidase IV inhibitor (DPP IV inhibitor)	dipeptidyl peptidase IV inhibitor	1	LQP	[296-298]
8697	dipeptidyl peptidase IV inhibitor (DPP IV inhibitor)	dipeptidyl peptidase IV inhibitor	1	WG	[377-378]
8758	dipeptidyl peptidase IV inhibitor (DPP IV inhibitor)	dipeptidyl peptidase IV inhibitor	2	AE	[239-240],[423-424]
8759	dipeptidyl peptidase IV inhibitor (DPP IV inhibitor)	dipeptidyl peptidase IV inhibitor	1	AF	[591-592]

8760	dipeptidyl peptidase IV inhibitor (DPP IV inhibitor)	dipeptidyl peptidase IV inhibitor	5	AG	[275-276],[311-312],[372-373],[498-499],[530-531]
8762	dipeptidyl peptidase IV inhibitor (DPP IV inhibitor)	dipeptidyl peptidase IV inhibitor	4	AS	[163-164],[357-358],[506-507],[585-586]
8763	dipeptidyl peptidase IV inhibitor (DPP IV inhibitor)	dipeptidyl peptidase IV inhibitor	3	AT	[250-251],[277-278],[439-440]
8764	dipeptidyl peptidase IV inhibitor (DPP IV inhibitor)	dipeptidyl peptidase IV inhibitor	1	AV	[243-244]
8767	dipeptidyl peptidase IV inhibitor (DPP IV inhibitor)	dipeptidyl peptidase IV inhibitor	3	DP	[13-14],[71-72],[118-119]
8768	dipeptidyl peptidase IV inhibitor (DPP IV inhibitor)	dipeptidyl peptidase IV inhibitor	4	DQ	[49-50],[66-67],[317-318],[345-346]
8769	dipeptidyl peptidase IV inhibitor (DPP IV inhibitor)	dipeptidyl peptidase IV inhibitor	2	DR	[115-116],[338-339]
8770	dipeptidyl peptidase IV inhibitor (DPP IV inhibitor)	dipeptidyl peptidase IV inhibitor	4	EG	[197-198],[351-352],[447-448],[465-466]
8771	dipeptidyl peptidase IV inhibitor (DPP IV inhibitor)	dipeptidyl peptidase IV inhibitor	1	EH	[409-410]
8772	dipeptidyl peptidase IV inhibitor (DPP IV inhibitor)	dipeptidyl peptidase IV inhibitor	3	EI	[424-425],[554-555],[557-558]
8773	dipeptidyl peptidase IV inhibitor (DPP IV inhibitor)	dipeptidyl peptidase IV inhibitor	8	ES	[168-169],[194-195],[240-241],[261-262],[391-392],[476-477],[560-561],[564-565]
8774	dipeptidyl peptidase IV inhibitor (DPP IV inhibitor)	dipeptidyl peptidase IV inhibitor	1	ET	[258-259]
8775	dipeptidyl peptidase IV inhibitor (DPP IV inhibitor)	dipeptidyl peptidase IV inhibitor	1	EV	[199-200]
8777	dipeptidyl peptidase IV inhibitor (DPP IV inhibitor)	dipeptidyl peptidase IV inhibitor	2	EY	[23-24],[307-308]
8778	dipeptidyl peptidase IV inhibitor (DPP IV inhibitor)	dipeptidyl peptidase IV inhibitor	2	FN	[263-264],[548-549]
8779	dipeptidyl peptidase IV inhibitor (DPP IV inhibitor)	dipeptidyl peptidase IV inhibitor	1	FQ	[481-482]
8780	dipeptidyl peptidase IV inhibitor (DPP IV inhibitor)	dipeptidyl peptidase IV inhibitor	1	FR	[305-306]
8781	dipeptidyl peptidase IV inhibitor (DPP IV inhibitor)	dipeptidyl peptidase IV inhibitor	2	GE	[198-199],[521-522]
8782	dipeptidyl peptidase IV inhibitor (DPP IV inhibitor)	dipeptidyl peptidase IV inhibitor	1	GF	[516-517]

8783	dipeptidyl peptidase IV inhibitor (DPP IV inhibitor)	dipeptidyl peptidase IV inhibitor	1	GG	[383-384]
8784	dipeptidyl peptidase IV inhibitor (DPP IV inhibitor)	dipeptidyl peptidase IV inhibitor	1	GH	[499-500]
8785	dipeptidyl peptidase IV inhibitor (DPP IV inhibitor)	dipeptidyl peptidase IV inhibitor	1	GI	[215-216]
8786	dipeptidyl peptidase IV inhibitor (DPP IV inhibitor)	dipeptidyl peptidase IV inhibitor	1	GV	[352-353]
8790	dipeptidyl peptidase IV inhibitor (DPP IV inhibitor)	dipeptidyl peptidase IV inhibitor	1	HE	[193-194]
8792	dipeptidyl peptidase IV inhibitor (DPP IV inhibitor)	dipeptidyl peptidase IV inhibitor	1	HH	[235-236]
8794	dipeptidyl peptidase IV inhibitor (DPP IV inhibitor)	dipeptidyl peptidase IV inhibitor	2	HR	[124-125],[410-411]
8795	dipeptidyl peptidase IV inhibitor (DPP IV inhibitor)	dipeptidyl peptidase IV inhibitor	1	HS	[185-186]
8797	dipeptidyl peptidase IV inhibitor (DPP IV inhibitor)	dipeptidyl peptidase IV inhibitor	1	HV	[458-459]
8799	dipeptidyl peptidase IV inhibitor (DPP IV inhibitor)	dipeptidyl peptidase IV inhibitor	1	HY	[434-435]
8801	dipeptidyl peptidase IV inhibitor (DPP IV inhibitor)	dipeptidyl peptidase IV inhibitor	2	II	[354-355],[535-536]
8802	dipeptidyl peptidase IV inhibitor (DPP IV inhibitor)	dipeptidyl peptidase IV inhibitor	3	IL	[223-224],[328-329],[587-588]
8804	dipeptidyl peptidase IV inhibitor (DPP IV inhibitor)	dipeptidyl peptidase IV inhibitor	3	IN	[282-283],[519-520],[536-537]
8805	dipeptidyl peptidase IV inhibitor (DPP IV inhibitor)	dipeptidyl peptidase IV inhibitor	1	IQ	[132-133]
8806	dipeptidyl peptidase IV inhibitor (DPP IV inhibitor)	dipeptidyl peptidase IV inhibitor	4	IR	[40-41],[189-190],[271-272],[355-356]
8808	dipeptidyl peptidase IV inhibitor (DPP IV inhibitor)	dipeptidyl peptidase IV inhibitor	2	KE	[152-153],[544-545]
8814	dipeptidyl peptidase IV inhibitor (DPP IV inhibitor)	dipeptidyl peptidase IV inhibitor	1	KR	[426-427]
8815	dipeptidyl peptidase IV inhibitor (DPP IV inhibitor)	dipeptidyl peptidase IV inhibitor	2	KS	[314-315],[389-390]
8817	dipeptidyl peptidase IV inhibitor (DPP IV inhibitor)	dipeptidyl peptidase IV inhibitor	1	KV	[483-484]

8819	dipeptidyl peptidase IV inhibitor (DPP IV inhibitor)	dipeptidyl peptidase IV inhibitor	1	KY	[201-202]
8823	dipeptidyl peptidase IV inhibitor (DPP IV inhibitor)	dipeptidyl peptidase IV inhibitor	1	LN	[333-334]
8824	dipeptidyl peptidase IV inhibitor (DPP IV inhibitor)	dipeptidyl peptidase IV inhibitor	1	LT	[252-253]
8825	dipeptidyl peptidase IV inhibitor (DPP IV inhibitor)	dipeptidyl peptidase IV inhibitor	3	LV	[254-255],[329-330],[419-420]
8826	dipeptidyl peptidase IV inhibitor (DPP IV inhibitor)	dipeptidyl peptidase IV inhibitor	1	ME	[563-564]
8832	dipeptidyl peptidase IV inhibitor (DPP IV inhibitor)	dipeptidyl peptidase IV inhibitor	1	ML	[232-233]
8833	dipeptidyl peptidase IV inhibitor (DPP IV inhibitor)	dipeptidyl peptidase IV inhibitor	1	MM	[430-431]
8837	dipeptidyl peptidase IV inhibitor (DPP IV inhibitor)	dipeptidyl peptidase IV inhibitor	2	MV	[292-293],[431-432]
8840	dipeptidyl peptidase IV inhibitor (DPP IV inhibitor)	dipeptidyl peptidase IV inhibitor	1	ND	[326-327]
8841	dipeptidyl peptidase IV inhibitor (DPP IV inhibitor)	dipeptidyl peptidase IV inhibitor	2	NE	[287-288],[509-510]
8843	dipeptidyl peptidase IV inhibitor (DPP IV inhibitor)	dipeptidyl peptidase IV inhibitor	1	NG	[520-521]
8845	dipeptidyl peptidase IV inhibitor (DPP IV inhibitor)	dipeptidyl peptidase IV inhibitor	2	NL	[264-265],[511-512]
8846	dipeptidyl peptidase IV inhibitor (DPP IV inhibitor)	dipeptidyl peptidase IV inhibitor	1	NM	[549-550]
8847	dipeptidyl peptidase IV inhibitor (DPP IV inhibitor)	dipeptidyl peptidase IV inhibitor	3	NN	[300-301],[523-524],[533-534]
8848	dipeptidyl peptidase IV inhibitor (DPP IV inhibitor)	dipeptidyl peptidase IV inhibitor	4	NQ	[283-284],[397-398],[524-525],[537-538]
8850	dipeptidyl peptidase IV inhibitor (DPP IV inhibitor)	dipeptidyl peptidase IV inhibitor	2	NT	[229-230],[334-335]
8853	dipeptidyl peptidase IV inhibitor (DPP IV inhibitor)	dipeptidyl peptidase IV inhibitor	2	NY	[218-219],[421-422]
8855	dipeptidyl peptidase IV inhibitor (DPP IV inhibitor)	dipeptidyl peptidase IV inhibitor	1	PG	[302-303]
8856	dipeptidyl peptidase IV inhibitor (DPP IV inhibitor)	dipeptidyl peptidase IV inhibitor	3	PH	[234-235],[433-434],[457-458]

8857	dipeptidyl peptidase IV inhibitor (DPP IV inhibitor)	dipeptidyl peptidase IV inhibitor	2	PI	[385-386],[501-502]
8860	dipeptidyl peptidase IV inhibitor (DPP IV inhibitor)	dipeptidyl peptidase IV inhibitor	1	PN	[228-229]
8861	dipeptidyl peptidase IV inhibitor (DPP IV inhibitor)	dipeptidyl peptidase IV inhibitor	1	PQ	[119-120]
8862	dipeptidyl peptidase IV inhibitor (DPP IV inhibitor)	dipeptidyl peptidase IV inhibitor	1	PS	[393-394]
8863	dipeptidyl peptidase IV inhibitor (DPP IV inhibitor)	dipeptidyl peptidase IV inhibitor	1	PT	[569-570]
8864	dipeptidyl peptidase IV inhibitor (DPP IV inhibitor)	dipeptidyl peptidase IV inhibitor	1	PV	[298-299]
8865	dipeptidyl peptidase IV inhibitor (DPP IV inhibitor)	dipeptidyl peptidase IV inhibitor	1	PW	[376-377]
8866	dipeptidyl peptidase IV inhibitor (DPP IV inhibitor)	dipeptidyl peptidase IV inhibitor	1	PY	[181-182]
8868	dipeptidyl peptidase IV inhibitor (DPP IV inhibitor)	dipeptidyl peptidase IV inhibitor	2	QD	[117-118],[284-285]
8869	dipeptidyl peptidase IV inhibitor (DPP IV inhibitor)	dipeptidyl peptidase IV inhibitor	10	QE	[22-23],[53-54],[90-91],[105-106],[133-134],[257-258],[347-348],[359-360],[414-415],[472-473]
8870	dipeptidyl peptidase IV inhibitor (DPP IV inhibitor)	dipeptidyl peptidase IV inhibitor	3	QF	[304-305],[398-399],[401-402]
8871	dipeptidyl peptidase IV inhibitor (DPP IV inhibitor)	dipeptidyl peptidase IV inhibitor	5	QG	[28-29],[30-31],[155-156],[467-468],[578-579]
8872	dipeptidyl peptidase IV inhibitor (DPP IV inhibitor)	dipeptidyl peptidase IV inhibitor	1	QH	[367-368]
8873	dipeptidyl peptidase IV inhibitor (DPP IV inhibitor)	dipeptidyl peptidase IV inhibitor	2	QI	[39-40],[131-132]
8874	dipeptidyl peptidase IV inhibitor (DPP IV inhibitor)	dipeptidyl peptidase IV inhibitor	2	QL	[412-413],[538-539]
8875	dipeptidyl peptidase IV inhibitor (DPP IV inhibitor)	dipeptidyl peptidase IV inhibitor	3	QN	[67-68],[508-509],[532-533]
8876	dipeptidyl peptidase IV inhibitor (DPP IV inhibitor)	dipeptidyl peptidase IV inhibitor	12	QQ	[34-35],[35-36],[36-37],[80-81],[81-82],[104-105],[120-121],[121-122],[144-145],[154-155],[176-177],[346-347]
8877	dipeptidyl peptidase IV inhibitor (DPP IV inhibitor)	dipeptidyl peptidase IV inhibitor	5	QS	[135-136],[187-188],[318-319],[462-463],[573-574]
8878	dipeptidyl peptidase IV inhibitor (DPP IV inhibitor)	dipeptidyl peptidase IV inhibitor	1	QT	[92-93]

8881	dipeptidyl peptidase IV inhibitor (DPP IV inhibitor)	dipeptidyl peptidase IV inhibitor	3	QY	[17-18],[122-123],[150-151]
8882	dipeptidyl peptidase IV inhibitor (DPP IV inhibitor)	dipeptidyl peptidase IV inhibitor	11	RG	[1-2],[60-61],[88-89],[112-113],[159-160],[170-171],[214-215],[247-248],[427-428],[490-491],[576-577]
8883	dipeptidyl peptidase IV inhibitor (DPP IV inhibitor)	dipeptidyl peptidase IV inhibitor	2	RH	[178-179],[192-193]
8886	dipeptidyl peptidase IV inhibitor (DPP IV inhibitor)	dipeptidyl peptidase IV inhibitor	5	RL	[45-46],[289-290],[339-340],[487-488],[513-514]
8889	dipeptidyl peptidase IV inhibitor (DPP IV inhibitor)	dipeptidyl peptidase IV inhibitor	12	RR	[26-27],[58-59],[59-60],[87-88],[96-97],[97-98],[102-103],[128-129],[166-167],[379-380],[469-470],[575-576]
8891	dipeptidyl peptidase IV inhibitor (DPP IV inhibitor)	dipeptidyl peptidase IV inhibitor	2	SF	[262-263],[547-548]
8893	dipeptidyl peptidase IV inhibitor (DPP IV inhibitor)	dipeptidyl peptidase IV inhibitor	2	SI	[188-189],[586-587]
8894	dipeptidyl peptidase IV inhibitor (DPP IV inhibitor)	dipeptidyl peptidase IV inhibitor	1	SK	[437-438]
8895	dipeptidyl peptidase IV inhibitor (DPP IV inhibitor)	dipeptidyl peptidase IV inhibitor	1	SV	[241-242]
8897	dipeptidyl peptidase IV inhibitor (DPP IV inhibitor)	dipeptidyl peptidase IV inhibitor	4	SY	[319-320],[394-395],[463-464],[565-566]
8899	dipeptidyl peptidase IV inhibitor (DPP IV inhibitor)	dipeptidyl peptidase IV inhibitor	3	TE	[207-208],[210-211],[570-571]
8901	dipeptidyl peptidase IV inhibitor (DPP IV inhibitor)	dipeptidyl peptidase IV inhibitor	2	TG	[449-450],[478-479]
8905	dipeptidyl peptidase IV inhibitor (DPP IV inhibitor)	dipeptidyl peptidase IV inhibitor	3	TL	[93-94],[251-252],[253-254]
8909	dipeptidyl peptidase IV inhibitor (DPP IV inhibitor)	dipeptidyl peptidase IV inhibitor	2	TR	[246-247],[259-260]
8910	dipeptidyl peptidase IV inhibitor (DPP IV inhibitor)	dipeptidyl peptidase IV inhibitor	1	TS	[230-231]
8912	dipeptidyl peptidase IV inhibitor (DPP IV inhibitor)	dipeptidyl peptidase IV inhibitor	2	TV	[278-279],[440-441]
8915	dipeptidyl peptidase IV inhibitor (DPP IV inhibitor)	dipeptidyl peptidase IV inhibitor	1	VD	[64-65]
8916	dipeptidyl peptidase IV inhibitor (DPP IV inhibitor)	dipeptidyl peptidase IV inhibitor	1	VE	[446-447]
8917	dipeptidyl peptidase IV inhibitor (DPP IV inhibitor)	dipeptidyl peptidase IV inhibitor	1	VF	[323-324]

8920	dipeptidyl peptidase IV inhibitor (DPP IV inhibitor)	dipeptidyl peptidase IV inhibitor	5	VI	[222-223],[270-271],[281-282],[353-354],[495-496]
8921	dipeptidyl peptidase IV inhibitor (DPP IV inhibitor)	dipeptidyl peptidase IV inhibitor	2	VK	[200-201],[293-294]
8922	dipeptidyl peptidase IV inhibitor (DPP IV inhibitor)	dipeptidyl peptidase IV inhibitor	1	VL	[418-419]
8924	dipeptidyl peptidase IV inhibitor (DPP IV inhibitor)	dipeptidyl peptidase IV inhibitor	2	VN	[299-300],[420-421]
8926	dipeptidyl peptidase IV inhibitor (DPP IV inhibitor)	dipeptidyl peptidase IV inhibitor	2	VS	[255-256],[459-460]
8927	dipeptidyl peptidase IV inhibitor (DPP IV inhibitor)	dipeptidyl peptidase IV inhibitor	2	VT	[245-246],[484-485]
8929	dipeptidyl peptidase IV inhibitor (DPP IV inhibitor)	dipeptidyl peptidase IV inhibitor	2	VY	[279-280],[443-444]
8932	dipeptidyl peptidase IV inhibitor (DPP IV inhibitor)	dipeptidyl peptidase IV inhibitor	2	YA	[309-310],[422-423]
8934	dipeptidyl peptidase IV inhibitor (DPP IV inhibitor)	dipeptidyl peptidase IV inhibitor	3	YE	[76-77],[452-453],[464-465]
8935	dipeptidyl peptidase IV inhibitor (DPP IV inhibitor)	dipeptidyl peptidase IV inhibitor	2	YF	[183-184],[566-567]
8937	dipeptidyl peptidase IV inhibitor (DPP IV inhibitor)	dipeptidyl peptidase IV inhibitor	1	YH	[123-124]
8939	dipeptidyl peptidase IV inhibitor (DPP IV inhibitor)	dipeptidyl peptidase IV inhibitor	1	YK	[151-152]
8940	dipeptidyl peptidase IV inhibitor (DPP IV inhibitor)	dipeptidyl peptidase IV inhibitor	2	YL	[202-203],[320-321]
8942	dipeptidyl peptidase IV inhibitor (DPP IV inhibitor)	dipeptidyl peptidase IV inhibitor	1	YN	[435-436]
8944	dipeptidyl peptidase IV inhibitor (DPP IV inhibitor)	dipeptidyl peptidase IV inhibitor	2	YR	[18-19],[219-220]
8945	dipeptidyl peptidase IV inhibitor (DPP IV inhibitor)	dipeptidyl peptidase IV inhibitor	1	YS	[395-396]
8946	dipeptidyl peptidase IV inhibitor (DPP IV inhibitor)	dipeptidyl peptidase IV inhibitor	2	YV	[280-281],[444-445]
8948	dipeptidyl peptidase IV inhibitor (DPP IV inhibitor)	dipeptidyl peptidase IV inhibitor	2	YY	[182-183],[308-309]
9476	DPP-III inhibitor	dipeptidyl peptidase III inhibitor	2	YY	[182-183],[308-309]
9478	DPP-III inhibitor	dipeptidyl peptidase III inhibitor	4	LR	[213-214],[321-322],[362-363],[512-513]

9480	DPP-III inhibitor	dipeptidyl peptidase III inhibitor	2	YF	[183-184],[566-567]
9481	DPP-III inhibitor	dipeptidyl peptidase III inhibitor	1	YH	[123-124]
9482	DPP-III inhibitor	dipeptidyl peptidase III inhibitor	2	YL	[202-203],[320-321]
9483	DPP-III inhibitor	dipeptidyl peptidase III inhibitor	1	YK	[151-152]
9484	DPP-III inhibitor	dipeptidyl peptidase III inhibitor	2	YR	[18-19],[219-220]
9485	DPP-III inhibitor	dipeptidyl peptidase III inhibitor	12	RR	[26-27],[58-59],[59-60],[87-88],[96-97],[97-98],[102-103],[128-129],[166-167],[379-380],[469-470],[575-576]
9487	DPP-III inhibitor	dipeptidyl peptidase III inhibitor	2	GE	[198-199],[521-522]
9488	DPP-III inhibitor	dipeptidyl peptidase III inhibitor	1	GF	[516-517]
9489	DPP-III inhibitor	dipeptidyl peptidase III inhibitor	6	PR	[11-12],[14-15],[69-70],[165-166],[336-337],[551-552]
9490	DPP-III inhibitor	dipeptidyl peptidase III inhibitor	3	RF	[205-206],[342-343],[480-481]
9491	DPP-III inhibitor	dipeptidyl peptidase III inhibitor	3	RV	[220-221],[272-273],[322-323]
9492	DPP-III inhibitor	dipeptidyl peptidase III inhibitor	2	DA	[225-226],[238-239]
9494	DPP-III inhibitor	dipeptidyl peptidase III inhibitor	1	HK	[236-237]
9496	DPP-III inhibitor	dipeptidyl peptidase III inhibitor	2	HP	[500-501],[582-583]
9499	DPP-III inhibitor	dipeptidyl peptidase III inhibitor	3	LA	[488-489],[529-530],[584-585]
9500	DPP-III inhibitor	dipeptidyl peptidase III inhibitor	1	FA	[590-591]
9501	DPP-III inhibitor	dipeptidyl peptidase III inhibitor	1	FR	[305-306]
9502	DPP-III inhibitor	dipeptidyl peptidase III inhibitor	1	FL	[528-529]
9504	DPP-III inhibitor	dipeptidyl peptidase III inhibitor	4	PE	[72-73],[137-138],[161-162],[407-408]
9507	DPP-III inhibitor	dipeptidyl peptidase III inhibitor	1	SM	[231-232]
9509	DPP-III inhibitor	dipeptidyl peptidase III inhibitor	2	VY	[279-280],[443-444]
9511	DPP-III inhibitor	dipeptidyl peptidase III inhibitor	1	KA	[438-439]
8247	CaMPDE inhibitor	CaMPDE inhibitor	4	IR	[40-41],[189-190],[271-272],[355-356]
2835	Renin inhibitor	renin inhibitor	1	FT	[206-207]
2842	Renin inhibitor	renin inhibitor	4	LR	[213-214],[321-322],[362-363],[512-513]
8246	renin inhibitor	renin inhibitor	4	IR	[40-41],[189-190],[271-272],[355-356]
9431	Renin inhibitor	renin inhibitor	3	QF	[304-305],[398-399],[401-402]
9432	Renin inhibitor	renin inhibitor	2	SF	[262-263],[547-548]
9433	Renin inhibitor	renin inhibitor	2	YA	[309-310],[422-423]

Table S15. Profile of potential biological activity of fragments of protein Jug r 3.

ID	Name of peptide	Activity	Number	Sequence	Location
3460	Prolyl endopeptidase inhibitor	antiamnestic	3	PG	[87-88],[91-92],[98-99]
3361	ACE inhibitor from sardine	ACE inhibitor	1	LKL	[7-9]
3375	ACE inhibitor	ACE inhibitor	1	AAP	[20-22]
3421	ACE inhibitor	ACE inhibitor	1	LVL	[5-7]
3518	ACE inhibitor	ACE inhibitor	1	VAA	[19-21]
3524	ACE inhibitor (from bovine beta-CN)	ACE inhibitor	1	VPP	[51-53]
3532	ACE inhibitor	ACE inhibitor	1	GY	[42-43]
3539	ACE inhibitor from alpha-zein	ACE inhibitor	1	LAA	[93-95]
3544	ACE inhibitor	ACE inhibitor	1	LNP	[89-91]
7507	ACE inhibitor from Alaskan pollack skin	ACE inhibitor	2	PGL	[87-89],[91-93]
7509	ACE inhibitor from Alaskan pollack skin	ACE inhibitor	1	GLP	[96-98]
7511	ACE inhibitor from Alaskan pollack skin	ACE inhibitor	1	LPG	[97-99]
7558	ACE inhibitor from buckwheat	ACE inhibitor	2	VK	[59-60],[118-119]
7581	ACE inhibitor	ACE inhibitor	1	IP	[86-87]
7584	ACE inhibitor	ACE inhibitor	1	AP	[21-22]
7585	ACE inhibitor	ACE inhibitor	1	LA	[93-94]
7587	ACE inhibitor	ACE inhibitor	3	VP	[48-49],[51-52],[105-106]
7590	ACE inhibitor	ACE inhibitor	5	AA	[20-21],[65-66],[66-67],[74-75],[94-95]
7594	ACE inhibitor	ACE inhibitor	1	VG	[37-38]
7595	ACE inhibitor	ACE inhibitor	1	IG	[41-42]
7597	ACE inhibitor	ACE inhibitor	1	GM	[11-12]
7599	ACE inhibitor	ACE inhibitor	3	GL	[88-89],[92-93],[96-97]
7600	ACE inhibitor	ACE inhibitor	1	AG	[95-96]
7607	ACE inhibitor	ACE inhibitor	3	GS	[3-4],[38-39],[84-85]
7608	ACE inhibitor	ACE inhibitor	2	GV	[58-59],[102-103]
7610	ACE inhibitor	ACE inhibitor	1	GQ	[31-32]
7611	ACE inhibitor	ACE inhibitor	1	GK	[99-100]
7612	ACE inhibitor	ACE inhibitor	1	GT	[46-47]
7618	ACE inhibitor	ACE inhibitor	2	SG	[10-11],[83-84]

7621	ACE inhibitor	ACE inhibitor	1	TG	[2-3]
7623	ACE inhibitor	ACE inhibitor	1	EA	[25-26]
7624	ACE inhibitor	ACE inhibitor	1	NG	[57-58]
7625	ACE inhibitor	ACE inhibitor	3	PG	[87-88],[91-92],[98-99]
7693	ACE inhibitor from wakame	ACE inhibitor	1	KL	[8-9]
7697	ACE inhibitor from wakame	ACE inhibitor	1	YK	[107-108]
7698	ACE inhibitor from wakame	ACE inhibitor	1	NK	[63-64]
7743	ACE inhibitor	ACE inhibitor	2	KA	[64-65],[116-117]
7832	ACE inhibitor	ACE inhibitor	2	LN	[62-63],[89-90]
7833	ACE inhibitor	ACE inhibitor	1	PT	[49-50]
7836	ACE inhibitor	ACE inhibitor	1	PP	[52-53]
8951	ACE inhibitor	ACE inhibitor	2	AV	[26-27],[117-118]
9173	ACE inhibitor	ACE inhibitor	1	RG	[45-46]
9184	ACE inhibitor	ACE inhibitor	2	ST	[110-111],[112-113]
9213	ACE inhibitor	ACE inhibitor	1	LR	[44-45]
9756	ACE inhibitor	ACE inhibitor	1	PYK	[106-108]
3285	Antithrombotic peptide	antithrombotic	3	PG	[87-88],[91-92],[98-99]
8320	Glucose uptake stimulating peptide	stimulating	2	VL	[6-7],[13-14]
8321	Glucose uptake stimulating peptide	stimulating	1	LV	[5-6]
8326	Glucose uptake stimulating peptide	stimulating	1	LL	[14-15]
2890	neuropeptide	neuropeptide	1	GQ	[31-32]
8310	Anxiolytic peptide	neuropeptide	1	YL	[43-44]
2754	peptide regulating the stomach mucosal membrane activity	regulating	3	PG	[87-88],[91-92],[98-99]
8217	Antioxidative peptide	antioxidative	2	LK	[7-8],[79-80]
3751		bacterial permease ligand	1	KK	[80-81]
9538	Antinflammatory peptide	anti inflammatory	1	VPP	[51-53]
4006	Ubiqitin-mediated proteolysis activating peptide	activating ubiquitin-mediated proteolysis	1	LA	[93-94]
9650	Alpha-glucosidase inhibitor	alpha-glucosidase inhibitor	1	EA	[25-26]
9651	Alpha-glucosidase inhibitor	alpha-glucosidase inhibitor	1	PP	[52-53]
9695	Alpha-glucosidase inhibitor	alpha-glucosidase inhibitor	1	AD	[70-71]
3170	dipeptidyl peptidase IV inhibitor (DPP IV inhibitor)	dipeptidyl peptidase IV inhibitor	1	PP	[52-53]
3172	dipeptidyl peptidase IV inhibitor (DPP IV inhibitor)	dipeptidyl peptidase IV inhibitor	3	VA	[19-20],[23-24],[33-34]

3174	dipeptidyl peptidase IV inhibitor (DPP IV inhibitor)	dipeptidyl peptidase IV inhibitor	2	KA	[64-65],[116-117]
3175	dipeptidyl peptidase IV inhibitor (DPP IV inhibitor)	dipeptidyl peptidase IV inhibitor	1	LA	[93-94]
3177	dipeptidyl peptidase IV inhibitor (DPP IV inhibitor)	dipeptidyl peptidase IV inhibitor	1	AP	[21-22]
3180	dipeptidyl peptidase IV inhibitor (DPP IV inhibitor)	dipeptidyl peptidase IV inhibitor	1	LP	[97-98]
3181	dipeptidyl peptidase IV inhibitor (DPP IV inhibitor)	dipeptidyl peptidase IV inhibitor	3	VP	[48-49],[51-52],[105-106]
3182	dipeptidyl peptidase IV inhibitor (DPP IV inhibitor)	dipeptidyl peptidase IV inhibitor	1	LL	[14-15]
3183	dipeptidyl peptidase IV inhibitor (DPP IV inhibitor)	dipeptidyl peptidase IV inhibitor	1	VV	[18-19]
8501	Dipeptidyl peptidase IV inhibitor (DPP IV inhibitor)	dipeptidyl peptidase IV inhibitor	1	IP	[86-87]
8530	dipeptidyl peptidase IV inhibitor (DPP IV inhibitor)	dipeptidyl peptidase IV inhibitor	1	NP	[90-91]
8531	dipeptidyl peptidase IV inhibitor (DPP IV inhibitor)	dipeptidyl peptidase IV inhibitor	1	TA	[69-70]
8560	dipeptidyl peptidase IV inhibitor (DPP IV inhibitor)	dipeptidyl peptidase IV inhibitor	2	SL	[4-5],[61-62]
8561	dipeptidyl peptidase IV inhibitor (DPP IV inhibitor)	dipeptidyl peptidase IV inhibitor	3	GL	[88-89],[92-93],[96-97]
8637	dipeptidyl peptidase IV inhibitor (DPP IV inhibitor)	dipeptidyl peptidase IV inhibitor	5	AA	[20-21],[65-66],[66-67],[74-75],[94-95]
8757	dipeptidyl peptidase IV inhibitor (DPP IV inhibitor)	dipeptidyl peptidase IV inhibitor	1	AD	[70-71]
8758	dipeptidyl peptidase IV inhibitor (DPP IV inhibitor)	dipeptidyl peptidase IV inhibitor	1	AE	[24-25]
8760	dipeptidyl peptidase IV inhibitor (DPP IV inhibitor)	dipeptidyl peptidase IV inhibitor	1	AG	[95-96]
8762	dipeptidyl peptidase IV inhibitor (DPP IV inhibitor)	dipeptidyl peptidase IV inhibitor	1	AS	[34-35]
8763	dipeptidyl peptidase IV inhibitor (DPP IV inhibitor)	dipeptidyl peptidase IV inhibitor	1	AT	[67-68]
8764	dipeptidyl peptidase IV inhibitor (DPP IV inhibitor)	dipeptidyl peptidase IV inhibitor	2	AV	[26-27],[117-118]
8769	dipeptidyl peptidase IV inhibitor (DPP IV inhibitor)	dipeptidyl peptidase IV inhibitor	1	DR	[71-72]
8786	dipeptidyl peptidase IV inhibitor (DPP IV inhibitor)	dipeptidyl peptidase IV inhibitor	2	GV	[58-59],[102-103]
8788	dipeptidyl peptidase IV inhibitor (DPP IV inhibitor)	dipeptidyl peptidase IV inhibitor	1	GY	[42-43]
8812	dipeptidyl peptidase IV inhibitor (DPP IV inhibitor)	dipeptidyl peptidase IV inhibitor	1	KI	[108-109]
8813	dipeptidyl peptidase IV inhibitor (DPP IV inhibitor)	dipeptidyl peptidase IV inhibitor	1	KK	[80-81]
8815	dipeptidyl peptidase IV inhibitor (DPP IV inhibitor)	dipeptidyl peptidase IV inhibitor	1	KS	[60-61]
8816	dipeptidyl peptidase IV inhibitor (DPP IV inhibitor)	dipeptidyl peptidase IV inhibitor	1	KT	[81-82]
8823	dipeptidyl peptidase IV inhibitor (DPP IV inhibitor)	dipeptidyl peptidase IV inhibitor	2	LN	[62-63],[89-90]
8825	dipeptidyl peptidase IV inhibitor (DPP IV inhibitor)	dipeptidyl peptidase IV inhibitor	1	LV	[5-6]
8837	dipeptidyl peptidase IV inhibitor (DPP IV inhibitor)	dipeptidyl peptidase IV inhibitor	2	MV	[12-13],[17-18]
8843	dipeptidyl peptidase IV inhibitor (DPP IV inhibitor)	dipeptidyl peptidase IV inhibitor	1	NG	[57-58]
8855	dipeptidyl peptidase IV inhibitor (DPP IV inhibitor)	dipeptidyl peptidase IV inhibitor	3	PG	[87-88],[91-92],[98-99]
8862	dipeptidyl peptidase IV inhibitor (DPP IV inhibitor)	dipeptidyl peptidase IV inhibitor	1	PS	[53-54]

8863	dipeptidyl peptidase IV inhibitor (DPP IV inhibitor)	dipeptidyl peptidase IV inhibitor	1	PT	[49-50]
8864	dipeptidyl peptidase IV inhibitor (DPP IV inhibitor)	dipeptidyl peptidase IV inhibitor	1	PV	[22-23]
8866	dipeptidyl peptidase IV inhibitor (DPP IV inhibitor)	dipeptidyl peptidase IV inhibitor	1	PY	[106-107]
8867	dipeptidyl peptidase IV inhibitor (DPP IV inhibitor)	dipeptidyl peptidase IV inhibitor	1	QA	[73-74]
8879	dipeptidyl peptidase IV inhibitor (DPP IV inhibitor)	dipeptidyl peptidase IV inhibitor	1	QV	[32-33]
8882	dipeptidyl peptidase IV inhibitor (DPP IV inhibitor)	dipeptidyl peptidase IV inhibitor	1	RG	[45-46]
8893	dipeptidyl peptidase IV inhibitor (DPP IV inhibitor)	dipeptidyl peptidase IV inhibitor	1	SI	[85-86]
8895	dipeptidyl peptidase IV inhibitor (DPP IV inhibitor)	dipeptidyl peptidase IV inhibitor	2	SV	[36-37],[104-105]
8901	dipeptidyl peptidase IV inhibitor (DPP IV inhibitor)	dipeptidyl peptidase IV inhibitor	1	TG	[2-3]
8907	dipeptidyl peptidase IV inhibitor (DPP IV inhibitor)	dipeptidyl peptidase IV inhibitor	1	TN	[113-114]
8910	dipeptidyl peptidase IV inhibitor (DPP IV inhibitor)	dipeptidyl peptidase IV inhibitor	2	TS	[82-83],[111-112]
8911	dipeptidyl peptidase IV inhibitor (DPP IV inhibitor)	dipeptidyl peptidase IV inhibitor	1	TT	[68-69]
8912	dipeptidyl peptidase IV inhibitor (DPP IV inhibitor)	dipeptidyl peptidase IV inhibitor	2	TV	[47-48],[50-51]
8918	dipeptidyl peptidase IV inhibitor (DPP IV inhibitor)	dipeptidyl peptidase IV inhibitor	1	VG	[37-38]
8920	dipeptidyl peptidase IV inhibitor (DPP IV inhibitor)	dipeptidyl peptidase IV inhibitor	1	VI	[27-28]
8921	dipeptidyl peptidase IV inhibitor (DPP IV inhibitor)	dipeptidyl peptidase IV inhibitor	2	VK	[59-60],[118-119]
8922	dipeptidyl peptidase IV inhibitor (DPP IV inhibitor)	dipeptidyl peptidase IV inhibitor	2	VL	[6-7],[13-14]
8926	dipeptidyl peptidase IV inhibitor (DPP IV inhibitor)	dipeptidyl peptidase IV inhibitor	1	VS	[103-104]
8939	dipeptidyl peptidase IV inhibitor (DPP IV inhibitor)	dipeptidyl peptidase IV inhibitor	1	YK	[107-108]
8940	dipeptidyl peptidase IV inhibitor (DPP IV inhibitor)	dipeptidyl peptidase IV inhibitor	1	YL	[43-44]
9478	DPP-III inhibitor	dipeptidyl peptidase III inhibitor	1	LR	[44-45]
9482	DPP-III inhibitor	dipeptidyl peptidase III inhibitor	1	YL	[43-44]
9483	DPP-III inhibitor	dipeptidyl peptidase III inhibitor	1	YK	[107-108]
9499	DPP-III inhibitor	dipeptidyl peptidase III inhibitor	1	LA	[93-94]
9511	DPP-III inhibitor	dipeptidyl peptidase III inhibitor	2	KA	[64-65],[116-117]
2842	Renin inhibitor	renin inhibitor	1	LR	[44-45]

Table S16. Profile of potential biological activity of fragments of protein Jug r 4.0101.

ID	Name of peptide	Activity	Number	Sequence	Location
3460	Prolyl endopeptidase inhibitor	antiamnestic	2	PG	[110-111],[219-220]
3257	beta-lactokinin	ACE inhibitor	4	RL	[40-41],[255-256],[325-326],[478-479]
3258	beta-lactokinin	ACE inhibitor	3	IR	[139-140],[277-278],[457-458]
3381	ACE inhibitor	ACE inhibitor	2	LY	[369-370],[374-375]
3383	ACE inhibitor	ACE inhibitor	2	IY	[9-10],[338-339]
3384	ACE inhibitor	ACE inhibitor	2	VF	[233-234],[406-407]
3492	ACE inhibitor from sake	ACE inhibitor	2	VY	[96-97],[387-388]
3502	ACE inhibitor (BSA fr. 221-222)	ACE inhibitor	2	FP	[109-110],[150-151]
3528	ACE inhibitor	ACE inhibitor	1	LVR	[488-490]
3537	ACE inhibitor	ACE inhibitor	3	PR	[185-186],[279-280],[472-473]
3538	ACE inhibitor	ACE inhibitor	1	VSP	[447-449]
3547	ACE inhibitor	ACE inhibitor	1	IRA	[457-459]
3551	ACE inhibitor (from bovine beta-Lg)	ACE inhibitor	3	LF	[11-12],[17-18],[108-109]
3556	ACE inhibitor	ACE inhibitor	1	FY	[188-189]
3573		ACE inhibitor	1	AFP	[149-151]
3713	ACE inhibitor from alpha-zein	ACE inhibitor	1	LLP	[85-87]
7414	ACE inhibitor	ACE inhibitor	1	LRW	[357-359]
7502	ACE inhibitor	ACE inhibitor	1	IVR	[266-268]
7513	ACE inhibitor from Alaskan pollack skin	ACE inhibitor	1	PL	[449-450]
7547	ACE inhibitor	ACE inhibitor	1	IRP	[277-279]
7558	ACE inhibitor from buckwheat	ACE inhibitor	1	VK	[426-427]
7562	ACE inhibitor from soy hydrolysate	ACE inhibitor	2	IA	[98-99],[148-149]
7580	ACE inhibitor	ACE inhibitor	2	RW	[280-281],[358-359]
7581	ACE inhibitor	ACE inhibitor	2	IP	[419-420],[471-472]
7582	ACE inhibitor	ACE inhibitor	4	RP	[199-200],[218-219],[278-279],[492-493]
7583	ACE inhibitor	ACE inhibitor	3	AF	[149-150],[245-246],[468-469]
7584	ACE inhibitor	ACE inhibitor	1	AP	[92-93]
7585	ACE inhibitor	ACE inhibitor	5	LA	[22-23],[190-191],[242-243],[450-451],[465-466]
7586	ACE inhibitor	ACE inhibitor	1	KR	[427-428]

7587	ACE inhibitor	ACE inhibitor	1	VP	[376-377]
7588	ACE inhibitor	ACE inhibitor	5	RA	[335-336],[393-394],[428-429],[458-459],[504-505]
7589	ACE inhibitor	ACE inhibitor	1	YA	[388-389]
7591	ACE inhibitor	ACE inhibitor	2	GF	[236-237],[433-434]
7592	ACE inhibitor	ACE inhibitor	2	FR	[142-143],[198-199]
7595	ACE inhibitor	ACE inhibitor	1	IG	[330-331]
7596	ACE inhibitor	ACE inhibitor	1	GI	[103-104]
7598	ACE inhibitor	ACE inhibitor	1	GA	[367-368]
7599	ACE inhibitor	ACE inhibitor	3	GL	[83-84],[228-229],[316-317]
7600	ACE inhibitor	ACE inhibitor	6	AG	[54-55],[70-71],[152-153],[191-192],[343-344],[451-452]
7603	ACE inhibitor	ACE inhibitor	7	GR	[27-28],[101-102],[271-272],[311-312],[344-345],[392-393],[452-453]
7605	ACE inhibitor	ACE inhibitor	2	FG	[33-34],[402-403]
7606	ACE inhibitor	ACE inhibitor	5	DA	[42-43],[238-239],[244-245],[372-373],[475-476]
7607	ACE inhibitor	ACE inhibitor	1	GS	[162-163]
7608	ACE inhibitor	ACE inhibitor	4	GV	[55-56],[71-72],[106-107],[153-154]
7610	ACE inhibitor	ACE inhibitor	6	GQ	[34-35],[126-127],[202-203],[223-224],[403-404],[414-415]
7614	ACE inhibitor	ACE inhibitor	1	HG	[222-223]
7615	ACE inhibitor	ACE inhibitor	1	GE	[220-221]
7616	ACE inhibitor	ACE inhibitor	2	GG	[26-27],[310-311]
7617	ACE inhibitor	ACE inhibitor	2	QG	[125-126],[201-202]
7618	ACE inhibitor	ACE inhibitor	2	SG	[25-26],[235-236]
7619	ACE inhibitor	ACE inhibitor	1	LG	[229-230]
7620	ACE inhibitor	ACE inhibitor	2	GD	[145-146],[331-332]
7621	ACE inhibitor	ACE inhibitor	1	TG	[105-106]
7622	ACE inhibitor	ACE inhibitor	4	EG	[144-145],[270-271],[413-414],[432-433]
7623	ACE inhibitor	ACE inhibitor	3	EA	[51-52],[53-54],[342-343]
7624	ACE inhibitor	ACE inhibitor	3	NG	[19-20],[82-83],[315-316]
7625	ACE inhibitor	ACE inhibitor	2	PG	[110-111],[219-220]
7628	ACE inhibitor from k-CN (fr. 67-68)	ACE inhibitor	3	VR	[75-76],[267-268],[489-490]
7634	ACE inhibitor from k-CN (fr. 21-23)	ACE inhibitor	1	YVP	[375-377]
7635	ACE inhibitor from k-CN (fr. 51-53)	ACE inhibitor	1	VAV	[72-74]
7680	ACE inhibitor from pea vicilin	ACE inhibitor	1	QK	[137-138]

7681	ACE inhibitor from soy	ACE inhibitor	1	DG	[161-162]
7683	ACE inhibitor from garlic	ACE inhibitor	3	NF	[187-188],[401-402],[422-423]
7684	ACE inhibitor from garlic	ACE inhibitor	1	SY	[158-159]
7685	ACE inhibitor from garlic	ACE inhibitor	1	SF	[438-439]
7692	ACE inhibitor	ACE inhibitor	1	KF	[480-481]
7741	ACE inhibitor	ACE inhibitor	8	RR	[76-77],[210-211],[254-255],[263-264],[304-305],[308-309],[477-478],[503-504]
7742	ACE inhibitor	ACE inhibitor	4	AR	[99-100],[253-254],[429-430],[476-477]
7752	ACE inhibitor from shark meat hydrolysate	ACE inhibitor	1	EY	[205-206]
7810	ACE inhibitor from anchovy and bonito	ACE inhibitor	1	KP	[3-4]
7827	ACE inhibitor	ACE inhibitor	3	IE	[50-51],[57-58],[79-80]
7828	ACE inhibitor	ACE inhibitor	3	EV	[395-396],[463-464],[506-507]
7829	ACE inhibitor	ACE inhibitor	1	VE	[269-270]
7830	ACE inhibitor	ACE inhibitor	2	TE	[250-251],[340-341]
7831	ACE inhibitor	ACE inhibitor	3	LQ	[256-257],[274-275],[360-361]
7832	ACE inhibitor	ACE inhibitor	2	LN	[38-39],[381-382]
7833	ACE inhibitor	ACE inhibitor	1	PT	[46-47]
7835	ACE inhibitor	ACE inhibitor	2	AH	[155-156],[383-384]
7837	ACE inhibitor	ACE inhibitor	4	PQ	[87-88],[93-94],[200-201],[420-421]
7838	ACE inhibitor	ACE inhibitor	1	EW	[435-436]
7841	ACE inhibitor	ACE inhibitor	1	KE	[292-293]
7843	ACE inhibitor	ACE inhibitor	1	PH	[377-378]
7859	ACE inhibitor	ACE inhibitor	1	IEP	[79-81]
8182	ACE Inhibitor	ACE inhibitor	1	ALEP	[43-46]
8185	ACE inhibitor	ACE inhibitor	1	TF	[115-116]
8193	ACE inhibitor	ACE inhibitor	2	AI	[168-169],[456-457]
8402	ACE inhibitor	ACE inhibitor	1	LVY	[95-97]
8951	ACE inhibitor	ACE inhibitor	2	AV	[73-74],[424-425]
9029	ACE inhibitor	ACE inhibitor	1	ALP	[459-461]
9031	ACE inhibitor	ACE inhibitor	1	LEE	[317-319]
9046	ACE inhibitor	ACE inhibitor	1	VQV	[396-398]
9074	ACE inhibitor	ACE inhibitor	1	DF	[240-241]
9076	ACE inhibitor	ACE inhibitor	3	FQ	[67-68],[131-132],[469-470]

9077	ACE inhibitor	ACE inhibitor	1	YV	[375-376]
9078	ACE inhibitor	ACE inhibitor	1	YE	[206-207]
9079	ACE inhibitor	ACE inhibitor	1	IL	[5-6]
9107	ACE inhibitor	ACE inhibitor	1	WL	[359-360]
9173	ACE inhibitor	ACE inhibitor	6	RG	[100-101],[102-103],[227-228],[309-310],[366-367],[391-392]
9184	ACE inhibitor	ACE inhibitor	2	ST	[347-348],[486-487]
9185	ACE inhibitor	ACE inhibitor	1	YN	[159-160]
9196	ACE inhibitor	ACE inhibitor	2	AVV	[73-75],[424-426]
9213	ACE inhibitor	ACE inhibitor	5	LR	[324-325],[326-327],[357-358],[390-391],[411-412]
9468	ACE inhibitor	ACE inhibitor	1	RALP	[458-461]
9729	ACE inhibitor	ACE inhibitor	1	VVR	[74-76]
9730	ACE inhibitor	ACE inhibitor	1	NPR	[184-186]
3285	Antithrombotic peptide	antithrombotic	2	PG	[110-111],[219-220]
3356	Stimulating vasoactive substance release	stimulating	1	LLL	[84-86]
8320	Glucose uptake stimulating peptide	stimulating	3	VL	[107-108],[356-357],[464-465]
8321	Glucose uptake stimulating peptide	stimulating	2	LV	[95-96],[488-489]
8322	Glucose uptake stimulating peptide	stimulating	2	IV	[14-15],[266-267]
8323	Glucose uptake stimulating peptide	stimulating	1	IL	[5-6]
8324	Glucose uptake stimulating peptide	stimulating	1	LI	[13-14]
8325	Glucose uptake stimulating peptide	stimulating	1	II	[147-148]
8326	Glucose uptake stimulating peptide	stimulating	5	LL	[6-7],[84-85],[85-86],[171-172],[416-417]
8329	Stimulating vasoactive substance release	stimulating	6	EE	[117-118],[284-285],[289-290],[318-319],[341-342],[462-463]
8330	Stimulating vasoactive substance release	stimulating	4	SE	[258-259],[300-301],[302-303],[501-502]
3066	Immunostimulating peptide	immunostimulating	1	EAE	[51-53]
2890	neuropeptide	neuropeptide	6	GQ	[34-35],[126-127],[202-203],[223-224],[403-404],[414-415]
8310	Anxiolytic peptide	neuropeptide	2	YL	[10-11],[189-190]
2754	peptide regulating the stomach mucosal membrane activity	regulating	2	PG	[110-111],[219-220]
7868	peptide from Okara protein	antioxidative	1	ADF	[239-241]
7872	peptide from soybean protein isolates: beta-conglycinin and glycinin	antioxidative	2	LY	[369-370],[374-375]
7873	peptide from soybean protein isolates: beta-conglycinin and glycinin	antioxidative	2	IY	[9-10],[338-339]

7886	peptide derived from egg white albumin	antioxidative	2	AH	[155-156],[383-384]
7888	antioxidative peptide	antioxidative	1	EL	[410-411]
8037	synthetic peptide	antioxidative	1	PHW	[377-379]
8057	synthetic peptide	antioxidative	1	RHF	[140-142]
8064	synthetic peptide	antioxidative	1	RHQ	[135-137]
8076	synthetic peptide	antioxidative	1	RWL	[358-360]
8214	Antioxidative peptide	antioxidative	2	RW	[280-281],[358-359]
8215	Antioxidative peptide	antioxidative	3	IR	[139-140],[277-278],[457-458]
8217	Antioxidative peptide	antioxidative	1	LK	[479-480]
8218	Antioxidative peptide	antioxidative	1	KP	[3-4]
8220	antioxidative peptide	antioxidative	1	TFE	[115-117]
8224	antioxidative peptide	antioxidative	2	VY	[96-97],[387-388]
9363	Antioxidative peptide	antioxidative	1	NEN	[442-444]
4005		activating ubiquitin-mediated proteolysis	5	RA	[335-336],[393-394],[428-429],[458-459],[504-505]
4006	Ubiqitin-mediated proteolysis activating peptide	activating ubiquitin-mediated proteolysis	5	LA	[22-23],[190-191],[242-243],[450-451],[465-466]
9580	Hypolipidemic peptide	hypolipidemic	2	EF	[130-131],[197-198]
9650	Alpha-glucosidase inhibitor	alpha-glucosidase inhibitor	3	EA	[51-52],[53-54],[342-343]
9693	Alpha-glucosidase inhibitor	alpha-glucosidase inhibitor	1	VE	[269-270]
9694	Alpha-glucosidase inhibitor	alpha-glucosidase inhibitor	2	PE	[113-114],[461-462]
9695	Alpha-glucosidase inhibitor	alpha-glucosidase inhibitor	3	AD	[239-240],[243-244],[336-337]
3172	dipeptidyl peptidase IV inhibitor (DPP IV inhibitor)	dipeptidyl peptidase IV inhibitor	4	VA	[15-16],[72-73],[154-155],[167-168]
3173	dipeptidyl peptidase IV inhibitor (DPP IV inhibitor)	dipeptidyl peptidase IV inhibitor	1	MA	[1-2]
3175	dipeptidyl peptidase IV inhibitor (DPP IV inhibitor)	dipeptidyl peptidase IV inhibitor	5	LA	[22-23],[190-191],[242-243],[450-451],[465-466]
3176	dipeptidyl peptidase IV inhibitor (DPP IV inhibitor)	dipeptidyl peptidase IV inhibitor	1	FA	[423-424]
3177	dipeptidyl peptidase IV inhibitor (DPP IV inhibitor)	dipeptidyl peptidase IV inhibitor	1	AP	[92-93]
3179	dipeptidyl peptidase IV inhibitor (DPP IV inhibitor)	dipeptidyl peptidase IV inhibitor	1	PA	[151-152]
3180	dipeptidyl peptidase IV inhibitor (DPP IV inhibitor)	dipeptidyl peptidase IV inhibitor	3	LP	[86-87],[354-355],[460-461]
3181	dipeptidyl peptidase IV inhibitor (DPP IV inhibitor)	dipeptidyl peptidase IV inhibitor	1	VP	[376-377]
3182	dipeptidyl peptidase IV inhibitor (DPP IV inhibitor)	dipeptidyl peptidase IV inhibitor	5	LL	[6-7],[84-85],[85-86],[171-172],[416-417]
3183	dipeptidyl peptidase IV inhibitor (DPP IV inhibitor)	dipeptidyl peptidase IV inhibitor	5	VV	[74-75],[166-167],[386-387],[398-399],[425-426]
8501	Dipeptidyl peptidase IV inhibitor (DPP IV inhibitor)	dipeptidyl peptidase IV inhibitor	2	IP	[419-420],[471-472]

8505	Dipeptidyl peptidase IV inhibitor (DPP IV inhibitor)	dipeptidyl peptidase IV inhibitor	1	SP	[448-449]
8506	dipeptidyl peptidase IV inhibitor (DPP IV inhibitor)	dipeptidyl peptidase IV inhibitor	2	FP	[109-110],[150-151]
8518	dipeptidyl peptidase IV inhibitor (DPP IV inhibitor)	dipeptidyl peptidase IV inhibitor	4	RP	[199-200],[218-219],[278-279],[492-493]
8519	dipeptidyl peptidase IV inhibitor (DPP IV inhibitor)	dipeptidyl peptidase IV inhibitor	1	KP	[3-4]
8524	dipeptidyl peptidase IV inhibitor (DPP IV inhibitor)	dipeptidyl peptidase IV inhibitor	1	GA	[367-368]
8525	dipeptidyl peptidase IV inhibitor (DPP IV inhibitor)	dipeptidyl peptidase IV inhibitor	2	IA	[98-99],[148-149]
8526	dipeptidyl peptidase IV inhibitor (DPP IV inhibitor)	dipeptidyl peptidase IV inhibitor	5	RA	[335-336],[393-394],[428-429],[458-459],[504-505]
8529	dipeptidyl peptidase IV inhibitor (DPP IV inhibitor)	dipeptidyl peptidase IV inhibitor	2	EP	[45-46],[80-81]
8530	dipeptidyl peptidase IV inhibitor (DPP IV inhibitor)	dipeptidyl peptidase IV inhibitor	3	NP	[164-165],[184-185],[193-194]
8531	dipeptidyl peptidase IV inhibitor (DPP IV inhibitor)	dipeptidyl peptidase IV inhibitor	2	TA	[252-253],[467-468]
8555	dipeptidyl peptidase IV inhibitor (DPP IV inhibitor)	dipeptidyl peptidase IV inhibitor	2	FL	[12-13],[241-242]
8556	dipeptidyl peptidase IV inhibitor (DPP IV inhibitor)	dipeptidyl peptidase IV inhibitor	1	WV	[436-437]
8559	dipeptidyl peptidase IV inhibitor (DPP IV inhibitor)	dipeptidyl peptidase IV inhibitor	6	AL	[16-17],[43-44],[368-369],[373-374],[389-390],[459-460]
8560	dipeptidyl peptidase IV inhibitor (DPP IV inhibitor)	dipeptidyl peptidase IV inhibitor	1	SL	[170-171]
8561	dipeptidyl peptidase IV inhibitor (DPP IV inhibitor)	dipeptidyl peptidase IV inhibitor	3	GL	[83-84],[228-229],[316-317]
8594	dipeptidyl peptidase IV inhibitor (DPP IV inhibitor)	dipeptidyl peptidase IV inhibitor	3	VR	[75-76],[267-268],[489-490]
8638	dipeptidyl peptidase IV inhibitor (DPP IV inhibitor)	dipeptidyl peptidase IV inhibitor	1	PL	[449-450]
8677	dipeptidyl peptidase IV inhibitor (DPP IV inhibitor)	dipeptidyl peptidase IV inhibitor	1	WL	[359-360]
8680	dipeptidyl peptidase IV inhibitor (DPP IV inhibitor)	dipeptidyl peptidase IV inhibitor	1	WN	[379-380]
8687	dipeptidyl peptidase IV inhibitor (DPP IV inhibitor)	dipeptidyl peptidase IV inhibitor	2	WS	[157-158],[281-282]
8696	dipeptidyl peptidase IV inhibitor (DPP IV inhibitor)	dipeptidyl peptidase IV inhibitor	1	YT	[339-340]
8757	dipeptidyl peptidase IV inhibitor (DPP IV inhibitor)	dipeptidyl peptidase IV inhibitor	3	AD	[239-240],[243-244],[336-337]
8758	dipeptidyl peptidase IV inhibitor (DPP IV inhibitor)	dipeptidyl peptidase IV inhibitor	4	AE	[52-53],[364-365],[394-395],[505-506]
8759	dipeptidyl peptidase IV inhibitor (DPP IV inhibitor)	dipeptidyl peptidase IV inhibitor	3	AF	[149-150],[245-246],[468-469]
8760	dipeptidyl peptidase IV inhibitor (DPP IV inhibitor)	dipeptidyl peptidase IV inhibitor	6	AG	[54-55],[70-71],[152-153],[191-192],[343-344],[451-452]
8761	dipeptidyl peptidase IV inhibitor (DPP IV inhibitor)	dipeptidyl peptidase IV inhibitor	2	AH	[155-156],[383-384]
8763	dipeptidyl peptidase IV inhibitor (DPP IV inhibitor)	dipeptidyl peptidase IV inhibitor	1	AT	[466-467]
8764	dipeptidyl peptidase IV inhibitor (DPP IV inhibitor)	dipeptidyl peptidase IV inhibitor	2	AV	[73-74],[424-425]
8766	dipeptidyl peptidase IV inhibitor (DPP IV inhibitor)	dipeptidyl peptidase IV inhibitor	2	DN	[314-315],[400-401]
8767	dipeptidyl peptidase IV inhibitor (DPP IV inhibitor)	dipeptidyl peptidase IV inhibitor	2	DP	[61-62],[332-333]
8768	dipeptidyl peptidase IV inhibitor (DPP IV inhibitor)	dipeptidyl peptidase IV inhibitor	1	DQ	[182-183]
8769	dipeptidyl peptidase IV inhibitor (DPP IV inhibitor)	dipeptidyl peptidase IV inhibitor	1	DR	[134-135]

8770	dipeptidyl peptidase IV inhibitor (DPP IV inhibitor)	dipeptidyl peptidase IV inhibitor	4	EG	[144-145],[270-271],[413-414],[432-433]
8771	dipeptidyl peptidase IV inhibitor (DPP IV inhibitor)	dipeptidyl peptidase IV inhibitor	1	EH	[221-222]
8773	dipeptidyl peptidase IV inhibitor (DPP IV inhibitor)	dipeptidyl peptidase IV inhibitor	5	ES	[58-59],[118-119],[299-300],[301-302],[485-486]
8774	dipeptidyl peptidase IV inhibitor (DPP IV inhibitor)	dipeptidyl peptidase IV inhibitor	3	ET	[114-115],[251-252],[319-320]
8775	dipeptidyl peptidase IV inhibitor (DPP IV inhibitor)	dipeptidyl peptidase IV inhibitor	3	EV	[395-396],[463-464],[506-507]
8776	dipeptidyl peptidase IV inhibitor (DPP IV inhibitor)	dipeptidyl peptidase IV inhibitor	1	EW	[435-436]
8777	dipeptidyl peptidase IV inhibitor (DPP IV inhibitor)	dipeptidyl peptidase IV inhibitor	1	EY	[205-206]
8778	dipeptidyl peptidase IV inhibitor (DPP IV inhibitor)	dipeptidyl peptidase IV inhibitor	3	FN	[18-19],[246-247],[481-482]
8779	dipeptidyl peptidase IV inhibitor (DPP IV inhibitor)	dipeptidyl peptidase IV inhibitor	3	FQ	[67-68],[131-132],[469-470]
8780	dipeptidyl peptidase IV inhibitor (DPP IV inhibitor)	dipeptidyl peptidase IV inhibitor	2	FR	[142-143],[198-199]
8781	dipeptidyl peptidase IV inhibitor (DPP IV inhibitor)	dipeptidyl peptidase IV inhibitor	1	GE	[220-221]
8782	dipeptidyl peptidase IV inhibitor (DPP IV inhibitor)	dipeptidyl peptidase IV inhibitor	2	GF	[236-237],[433-434]
8783	dipeptidyl peptidase IV inhibitor (DPP IV inhibitor)	dipeptidyl peptidase IV inhibitor	2	GG	[26-27],[310-311]
8785	dipeptidyl peptidase IV inhibitor (DPP IV inhibitor)	dipeptidyl peptidase IV inhibitor	1	GI	[103-104]
8786	dipeptidyl peptidase IV inhibitor (DPP IV inhibitor)	dipeptidyl peptidase IV inhibitor	4	GV	[55-56],[71-72],[106-107],[153-154]
8791	dipeptidyl peptidase IV inhibitor (DPP IV inhibitor)	dipeptidyl peptidase IV inhibitor	1	HF	[141-142]
8794	dipeptidyl peptidase IV inhibitor (DPP IV inhibitor)	dipeptidyl peptidase IV inhibitor	2	HR	[209-210],[262-263]
8795	dipeptidyl peptidase IV inhibitor (DPP IV inhibitor)	dipeptidyl peptidase IV inhibitor	1	HS	[384-385]
8796	dipeptidyl peptidase IV inhibitor (DPP IV inhibitor)	dipeptidyl peptidase IV inhibitor	1	HT	[352-353]
8798	dipeptidyl peptidase IV inhibitor (DPP IV inhibitor)	dipeptidyl peptidase IV inhibitor	2	HW	[156-157],[378-379]
8801	dipeptidyl peptidase IV inhibitor (DPP IV inhibitor)	dipeptidyl peptidase IV inhibitor	1	II	[147-148]
8802	dipeptidyl peptidase IV inhibitor (DPP IV inhibitor)	dipeptidyl peptidase IV inhibitor	1	IL	[5-6]
8806	dipeptidyl peptidase IV inhibitor (DPP IV inhibitor)	dipeptidyl peptidase IV inhibitor	3	IR	[139-140],[277-278],[457-458]
8808	dipeptidyl peptidase IV inhibitor (DPP IV inhibitor)	dipeptidyl peptidase IV inhibitor	1	KE	[292-293]
8809	dipeptidyl peptidase IV inhibitor (DPP IV inhibitor)	dipeptidyl peptidase IV inhibitor	1	KF	[480-481]
8812	dipeptidyl peptidase IV inhibitor (DPP IV inhibitor)	dipeptidyl peptidase IV inhibitor	1	KI	[138-139]
8814	dipeptidyl peptidase IV inhibitor (DPP IV inhibitor)	dipeptidyl peptidase IV inhibitor	1	KR	[427-428]
8816	dipeptidyl peptidase IV inhibitor (DPP IV inhibitor)	dipeptidyl peptidase IV inhibitor	1	KT	[440-441]
8821	dipeptidyl peptidase IV inhibitor (DPP IV inhibitor)	dipeptidyl peptidase IV inhibitor	1	LI	[13-14]
8823	dipeptidyl peptidase IV inhibitor (DPP IV inhibitor)	dipeptidyl peptidase IV inhibitor	2	LN	[38-39],[381-382]
8824	dipeptidyl peptidase IV inhibitor (DPP IV inhibitor)	dipeptidyl peptidase IV inhibitor	1	LT	[417-418]
8825	dipeptidyl peptidase IV inhibitor (DPP IV inhibitor)	dipeptidyl peptidase IV inhibitor	2	LV	[95-96],[488-489]

8837	dipeptidyl peptidase IV inhibitor (DPP IV inhibitor)	dipeptidyl peptidase IV inhibitor	1	MV	[446-447]
8839	dipeptidyl peptidase IV inhibitor (DPP IV inhibitor)	dipeptidyl peptidase IV inhibitor	4	NA	[91-92],[177-178],[382-383],[444-445]
8840	dipeptidyl peptidase IV inhibitor (DPP IV inhibitor)	dipeptidyl peptidase IV inhibitor	2	ND	[160-161],[260-261]
8841	dipeptidyl peptidase IV inhibitor (DPP IV inhibitor)	dipeptidyl peptidase IV inhibitor	2	NE	[431-432],[442-443]
8842	dipeptidyl peptidase IV inhibitor (DPP IV inhibitor)	dipeptidyl peptidase IV inhibitor	3	NF	[187-188],[401-402],[422-423]
8843	dipeptidyl peptidase IV inhibitor (DPP IV inhibitor)	dipeptidyl peptidase IV inhibitor	3	NG	[19-20],[82-83],[315-316]
8845	dipeptidyl peptidase IV inhibitor (DPP IV inhibitor)	dipeptidyl peptidase IV inhibitor	1	NL	[380-381]
8847	dipeptidyl peptidase IV inhibitor (DPP IV inhibitor)	dipeptidyl peptidase IV inhibitor	4	NN	[63-64],[175-176],[176-177],[231-232]
8848	dipeptidyl peptidase IV inhibitor (DPP IV inhibitor)	dipeptidyl peptidase IV inhibitor	2	NQ	[64-65],[179-180]
8849	dipeptidyl peptidase IV inhibitor (DPP IV inhibitor)	dipeptidyl peptidase IV inhibitor	3	NR	[39-40],[48-49],[482-483]
8851	dipeptidyl peptidase IV inhibitor (DPP IV inhibitor)	dipeptidyl peptidase IV inhibitor	2	NV	[232-233],[247-248]
8855	dipeptidyl peptidase IV inhibitor (DPP IV inhibitor)	dipeptidyl peptidase IV inhibitor	2	PG	[110-111],[219-220]
8856	dipeptidyl peptidase IV inhibitor (DPP IV inhibitor)	dipeptidyl peptidase IV inhibitor	1	PH	[377-378]
8857	dipeptidyl peptidase IV inhibitor (DPP IV inhibitor)	dipeptidyl peptidase IV inhibitor	1	PI	[4-5]
8860	dipeptidyl peptidase IV inhibitor (DPP IV inhibitor)	dipeptidyl peptidase IV inhibitor	2	PN	[62-63],[81-82]
8861	dipeptidyl peptidase IV inhibitor (DPP IV inhibitor)	dipeptidyl peptidase IV inhibitor	4	PQ	[87-88],[93-94],[200-201],[420-421]
8862	dipeptidyl peptidase IV inhibitor (DPP IV inhibitor)	dipeptidyl peptidase IV inhibitor	2	PS	[333-334],[493-494]
8863	dipeptidyl peptidase IV inhibitor (DPP IV inhibitor)	dipeptidyl peptidase IV inhibitor	1	PT	[46-47]
8864	dipeptidyl peptidase IV inhibitor (DPP IV inhibitor)	dipeptidyl peptidase IV inhibitor	2	PV	[165-166],[355-356]
8868	dipeptidyl peptidase IV inhibitor (DPP IV inhibitor)	dipeptidyl peptidase IV inhibitor	1	QD	[133-134]
8869	dipeptidyl peptidase IV inhibitor (DPP IV inhibitor)	dipeptidyl peptidase IV inhibitor	3	QE	[204-205],[286-287],[484-485]
8870	dipeptidyl peptidase IV inhibitor (DPP IV inhibitor)	dipeptidyl peptidase IV inhibitor	2	QF	[32-33],[66-67]
8871	dipeptidyl peptidase IV inhibitor (DPP IV inhibitor)	dipeptidyl peptidase IV inhibitor	2	QG	[125-126],[201-202]
8872	dipeptidyl peptidase IV inhibitor (DPP IV inhibitor)	dipeptidyl peptidase IV inhibitor	1	QH	[208-209]
8873	dipeptidyl peptidase IV inhibitor (DPP IV inhibitor)	dipeptidyl peptidase IV inhibitor	1	QI	[470-471]
8874	dipeptidyl peptidase IV inhibitor (DPP IV inhibitor)	dipeptidyl peptidase IV inhibitor	6	QL	[37-38],[94-95],[180-181],[273-274],[361-362],[415-416]
8875	dipeptidyl peptidase IV inhibitor (DPP IV inhibitor)	dipeptidyl peptidase IV inhibitor	2	QN	[183-184],[421-422]
8876	dipeptidyl peptidase IV inhibitor (DPP IV inhibitor)	dipeptidyl peptidase IV inhibitor	12	QQ	[29-30],[30-31],[31-32],[65-66],[124-125],[132-133],[203-204],[212-213],[213-214],[216-217],[224-225],[225-226]
8877	dipeptidyl peptidase IV inhibitor (DPP IV inhibitor)	dipeptidyl peptidase IV inhibitor	5	QS	[24-25],[122-123],[127-128],[257-258],[306-307]
8878	dipeptidyl peptidase IV inhibitor (DPP IV inhibitor)	dipeptidyl peptidase IV inhibitor	1	QT	[404-405]
8879	dipeptidyl peptidase IV inhibitor (DPP IV inhibitor)	dipeptidyl peptidase IV inhibitor	2	QV	[275-276],[397-398]

8881	dipeptidyl peptidase IV inhibitor (DPP IV inhibitor)	dipeptidyl peptidase IV inhibitor	1	QY	[88-89]
8882	dipeptidyl peptidase IV inhibitor (DPP IV inhibitor)	dipeptidyl peptidase IV inhibitor	6	RG	[100-101],[102-103],[227-228],[309-310],[366-367],[391-392]
8883	dipeptidyl peptidase IV inhibitor (DPP IV inhibitor)	dipeptidyl peptidase IV inhibitor	2	RH	[135-136],[140-141]
8884	dipeptidyl peptidase IV inhibitor (DPP IV inhibitor)	dipeptidyl peptidase IV inhibitor	2	RI	[49-50],[345-346]
8885	dipeptidyl peptidase IV inhibitor (DPP IV inhibitor)	dipeptidyl peptidase IV inhibitor	1	RK	[291-292]
8886	dipeptidyl peptidase IV inhibitor (DPP IV inhibitor)	dipeptidyl peptidase IV inhibitor	4	RL	[40-41],[255-256],[325-326],[478-479]
8888	dipeptidyl peptidase IV inhibitor (DPP IV inhibitor)	dipeptidyl peptidase IV inhibitor	2	RN	[186-187],[430-431]
8889	dipeptidyl peptidase IV inhibitor (DPP IV inhibitor)	dipeptidyl peptidase IV inhibitor	8	RR	[76-77],[210-211],[254-255],[263-264],[304-305],[308-309],[477-478],[503-504]
8890	dipeptidyl peptidase IV inhibitor (DPP IV inhibitor)	dipeptidyl peptidase IV inhibitor	2	RW	[280-281],[358-359]
8891	dipeptidyl peptidase IV inhibitor (DPP IV inhibitor)	dipeptidyl peptidase IV inhibitor	1	SF	[438-439]
8892	dipeptidyl peptidase IV inhibitor (DPP IV inhibitor)	dipeptidyl peptidase IV inhibitor	1	SH	[351-352]
8893	dipeptidyl peptidase IV inhibitor (DPP IV inhibitor)	dipeptidyl peptidase IV inhibitor	2	SI	[8-9],[265-266]
8895	dipeptidyl peptidase IV inhibitor (DPP IV inhibitor)	dipeptidyl peptidase IV inhibitor	1	SV	[385-386]
8896	dipeptidyl peptidase IV inhibitor (DPP IV inhibitor)	dipeptidyl peptidase IV inhibitor	1	SW	[59-60]
8897	dipeptidyl peptidase IV inhibitor (DPP IV inhibitor)	dipeptidyl peptidase IV inhibitor	1	SY	[158-159]
8899	dipeptidyl peptidase IV inhibitor (DPP IV inhibitor)	dipeptidyl peptidase IV inhibitor	2	TE	[250-251],[340-341]
8900	dipeptidyl peptidase IV inhibitor (DPP IV inhibitor)	dipeptidyl peptidase IV inhibitor	1	TF	[115-116]
8901	dipeptidyl peptidase IV inhibitor (DPP IV inhibitor)	dipeptidyl peptidase IV inhibitor	1	TG	[105-106]
8903	dipeptidyl peptidase IV inhibitor (DPP IV inhibitor)	dipeptidyl peptidase IV inhibitor	3	TI	[78-79],[320-321],[418-419]
8905	dipeptidyl peptidase IV inhibitor (DPP IV inhibitor)	dipeptidyl peptidase IV inhibitor	3	TL	[323-324],[353-354],[487-488]
8907	dipeptidyl peptidase IV inhibitor (DPP IV inhibitor)	dipeptidyl peptidase IV inhibitor	3	TN	[47-48],[174-175],[441-442]
8910	dipeptidyl peptidase IV inhibitor (DPP IV inhibitor)	dipeptidyl peptidase IV inhibitor	1	TS	[454-455]
8912	dipeptidyl peptidase IV inhibitor (DPP IV inhibitor)	dipeptidyl peptidase IV inhibitor	2	TV	[348-349],[405-406]
8915	dipeptidyl peptidase IV inhibitor (DPP IV inhibitor)	dipeptidyl peptidase IV inhibitor	2	VD	[248-249],[399-400]
8916	dipeptidyl peptidase IV inhibitor (DPP IV inhibitor)	dipeptidyl peptidase IV inhibitor	1	VE	[269-270]
8917	dipeptidyl peptidase IV inhibitor (DPP IV inhibitor)	dipeptidyl peptidase IV inhibitor	2	VF	[233-234],[406-407]
8920	dipeptidyl peptidase IV inhibitor (DPP IV inhibitor)	dipeptidyl peptidase IV inhibitor	2	VI	[56-57],[276-277]
8921	dipeptidyl peptidase IV inhibitor (DPP IV inhibitor)	dipeptidyl peptidase IV inhibitor	1	VK	[426-427]
8922	dipeptidyl peptidase IV inhibitor (DPP IV inhibitor)	dipeptidyl peptidase IV inhibitor	3	VL	[107-108],[356-357],[464-465]
8924	dipeptidyl peptidase IV inhibitor (DPP IV inhibitor)	dipeptidyl peptidase IV inhibitor	1	VN	[349-350]
8925	dipeptidyl peptidase IV inhibitor (DPP IV inhibitor)	dipeptidyl peptidase IV inhibitor	1	VQ	[396-397]
8926	dipeptidyl peptidase IV inhibitor (DPP IV inhibitor)	dipeptidyl peptidase IV inhibitor	2	VS	[437-438],[447-448]

8929	dipeptidyl peptidase IV inhibitor (DPP IV inhibitor)	dipeptidyl peptidase IV inhibitor	2	VY	[96-97],[387-388]
8930	dipeptidyl peptidase IV inhibitor (DPP IV inhibitor)	dipeptidyl peptidase IV inhibitor	1	WD	[60-61]
8932	dipeptidyl peptidase IV inhibitor (DPP IV inhibitor)	dipeptidyl peptidase IV inhibitor	1	YA	[388-389]
8934	dipeptidyl peptidase IV inhibitor (DPP IV inhibitor)	dipeptidyl peptidase IV inhibitor	1	YE	[206-207]
8938	dipeptidyl peptidase IV inhibitor (DPP IV inhibitor)	dipeptidyl peptidase IV inhibitor	1	YI	[97-98]
8940	dipeptidyl peptidase IV inhibitor (DPP IV inhibitor)	dipeptidyl peptidase IV inhibitor	2	YL	[10-11],[189-190]
8942	dipeptidyl peptidase IV inhibitor (DPP IV inhibitor)	dipeptidyl peptidase IV inhibitor	1	YN	[159-160]
8945	dipeptidyl peptidase IV inhibitor (DPP IV inhibitor)	dipeptidyl peptidase IV inhibitor	2	YS	[89-90],[370-371]
8946	dipeptidyl peptidase IV inhibitor (DPP IV inhibitor)	dipeptidyl peptidase IV inhibitor	1	YV	[375-376]
9339	Dipeptidyl peptidase IV inhibitor	dipeptidyl peptidase IV inhibitor	1	LPQ	[86-88]
9477	DPP-III inhibitor	dipeptidyl peptidase III inhibitor	2	RW	[280-281],[358-359]
9478	DPP-III inhibitor	dipeptidyl peptidase III inhibitor	5	LR	[324-325],[326-327],[357-358],[390-391],[411-412]
9482	DPP-III inhibitor	dipeptidyl peptidase III inhibitor	2	YL	[10-11],[189-190]
9485	DPP-III inhibitor	dipeptidyl peptidase III inhibitor	8	RR	[76-77],[210-211],[254-255],[263-264],[304-305],[308-309],[477-478],[503-504]
9486	DPP-III inhibitor	dipeptidyl peptidase III inhibitor	1	TF	[115-116]
9487	DPP-III inhibitor	dipeptidyl peptidase III inhibitor	1	GE	[220-221]
9488	DPP-III inhibitor	dipeptidyl peptidase III inhibitor	2	GF	[236-237],[433-434]
9489	DPP-III inhibitor	dipeptidyl peptidase III inhibitor	3	PR	[185-186],[279-280],[472-473]
9491	DPP-III inhibitor	dipeptidyl peptidase III inhibitor	1	RV	[268-269]
9492	DPP-III inhibitor	dipeptidyl peptidase III inhibitor	5	DA	[42-43],[238-239],[244-245],[372-373],[475-476]
9495	DPP-III inhibitor	dipeptidyl peptidase III inhibitor	1	HF	[141-142]
9499	DPP-III inhibitor	dipeptidyl peptidase III inhibitor	5	LA	[22-23],[190-191],[242-243],[450-451],[465-466]
9500	DPP-III inhibitor	dipeptidyl peptidase III inhibitor	1	FA	[423-424]
9501	DPP-III inhibitor	dipeptidyl peptidase III inhibitor	2	FR	[142-143],[198-199]
9502	DPP-III inhibitor	dipeptidyl peptidase III inhibitor	2	FL	[12-13],[241-242]
9504	DPP-III inhibitor	dipeptidyl peptidase III inhibitor	2	PE	[113-114],[461-462]
9509	DPP-III inhibitor	dipeptidyl peptidase III inhibitor	2	VY	[96-97],[387-388]
9510	DPP-III inhibitor	dipeptidyl peptidase III inhibitor	1	YI	[97-98]
8247	CaMPDE inhibitor	CaMPDE inhibitor	3	IR	[139-140],[277-278],[457-458]
8249	CaMPDE inhibitor	CaMPDE inhibitor	1	KF	[480-481]
8250	CaMPDE inhibitor	CaMPDE inhibitor	2	EF	[130-131],[197-198]
2842	Renin inhibitor	renin inhibitor	5	LR	[324-325],[326-327],[357-358],[390-391],[411-412]

8246	renin inhibitor	renin inhibitor	3	IR	[139-140],[277-278],[457-458]
8248	Renin inhibitor	renin inhibitor	1	KF	[480-481]
8251	Renin inhibitor	renin inhibitor	2	EF	[130-131],[197-198]
9430	Renin inhibitor	renin inhibitor	3	NR	[39-40],[48-49],[482-483]
9431	Renin inhibitor	renin inhibitor	2	QF	[32-33],[66-67]
9432	Renin inhibitor	renin inhibitor	1	SF	[438-439]
9433	Renin inhibitor	renin inhibitor	1	YA	[388-389]
9469	Renin inhibitor	renin inhibitor	1	RALP	[458-461]
9470	Renin inhibitor	renin inhibitor	2	LY	[369-370],[374-375]
9471	Renin inhibitor	renin inhibitor	1	TF	[115-116]

Table S17. Profile of potential biological activity of fragments of protein Pis v 1.0101.

ID	Name of peptide	Activity	Number	Sequence	Location
3381	ACE inhibitor	ACE inhibitor	1	LY	[121-122]
3383	ACE inhibitor	ACE inhibitor	1	IY	[22-23]
3388	ACE inhibitor	ACE inhibitor	1	MY	[55-56]
3421	ACE inhibitor	ACE inhibitor	1	LVL	[4-6]
3488	ACE inhibitor from sake lees	ACE inhibitor	1	YW	[146-147]
3537	ACE inhibitor	ACE inhibitor	1	PR	[129-130]
3539	ACE inhibitor from alpha-zein	ACE inhibitor	1	LAA	[16-18]
7558	ACE inhibitor from buckwheat	ACE inhibitor	1	VK	[104-105]
7583	ACE inhibitor	ACE inhibitor	2	AF	[10-11],[12-13]
7585	ACE inhibitor	ACE inhibitor	1	LA	[16-17]
7586	ACE inhibitor	ACE inhibitor	1	KR	[105-106]
7588	ACE inhibitor	ACE inhibitor	1	RA	[24-25]
7590	ACE inhibitor	ACE inhibitor	1	AA	[17-18]
7592	ACE inhibitor	ACE inhibitor	1	FR	[113-114]
7601	ACE inhibitor	ACE inhibitor	1	GH	[67-68]
7610	ACE inhibitor	ACE inhibitor	2	GQ	[37-38],[111-112]
7615	ACE inhibitor	ACE inhibitor	2	GE	[31-32],[115-116]
7617	ACE inhibitor	ACE inhibitor	2	QG	[110-111],[138-139]
7618	ACE inhibitor	ACE inhibitor	1	SG	[36-37]
7622	ACE inhibitor	ACE inhibitor	1	EG	[30-31]
7680	ACE inhibitor from pea vicilin	ACE inhibitor	3	QK	[41-42],[48-49],[62-63]
7681	ACE inhibitor from soy	ACE inhibitor	1	DG	[66-67]
7684	ACE inhibitor from garlic	ACE inhibitor	1	SY	[148-149]
7692	ACE inhibitor	ACE inhibitor	1	KF	[49-50]
7693	ACE inhibitor from wakame	ACE inhibitor	2	KL	[3-4],[117-118]
7742	ACE inhibitor	ACE inhibitor	1	AR	[72-73]
7751	ACE inhibitor from shark meat hydrolysate	ACE inhibitor	1	CF	[80-81]
7828	ACE inhibitor	ACE inhibitor	3	EV	[28-29],[60-61],[90-91]
7829	ACE inhibitor	ACE inhibitor	2	VE	[27-28],[29-30]

7831	ACE inhibitor	ACE inhibitor	2	LQ	[88-89],[118-119]
7840	ACE inhibitor	ACE inhibitor	1	EK	[116-117]
9042	ACE inhibitor	ACE inhibitor	1	AFL	[12-14]
9077	ACE inhibitor	ACE inhibitor	1	YV	[56-57]
9078	ACE inhibitor	ACE inhibitor	1	YE	[122-123]
9079	ACE inhibitor	ACE inhibitor	1	IL	[15-16]
9173	ACE inhibitor	ACE inhibitor	1	RG	[114-115]
9473	ACE inhibitor	ACE inhibitor	1	GHS	[67-69]
3356	Stimulating vasoactive substance release	stimulating	1	LLL	[6-8]
8320	Glucose uptake stimulating peptide	stimulating	1	VL	[5-6]
8321	Glucose uptake stimulating peptide	stimulating	1	LV	[4-5]
8323	Glucose uptake stimulating peptide	stimulating	1	IL	[15-16]
8324	Glucose uptake stimulating peptide	stimulating	1	LI	[14-15]
8326	Glucose uptake stimulating peptide	stimulating	2	LL	[6-7],[7-8]
8329	Stimulating vasoactive substance release	stimulating	1	EE	[45-46]
8330	Stimulating vasoactive substance release	stimulating	1	SE	[126-127]
2890	neuropeptide	neuropeptide	2	GQ	[37-38],[111-112]
9534	Kyotorphin	neuropeptide	1	YR	[23-24]
7872	peptide from soybean protein isolates: beta-conglycinin and glycinin	antioxidative	1	LY	[121-122]
7873	peptide from soybean protein isolates: beta-conglycinin and glycinin	antioxidative	1	IY	[22-23]
7888	antioxidative peptide	antioxidative	3	EL	[87-88],[120-121],[127-128]
8090	peptide derived from sardine muscle	antioxidative	1	MY	[55-56]
3751		bacterial permease ligand	1	KK	[93-94]
9472	Renin inhibitor	hypotensive	1	GHS	[67-69]
4005		activating ubiquitin-mediated proteolysis	1	RA	[24-25]
4006	Ubiqitin-mediated proteolysis activating peptide	activating ubiquitin-mediated proteolysis	1	LA	[16-17]
9693	Alpha-glucosidase inhibitor	alpha-glucosidase inhibitor	2	VE	[27-28],[29-30]
3173	dipeptidyl peptidase IV inhibitor (DPP IV inhibitor)	dipeptidyl peptidase IV inhibitor	1	MA	[1-2]
3175	dipeptidyl peptidase IV inhibitor (DPP IV inhibitor)	dipeptidyl peptidase IV inhibitor	1	LA	[16-17]
3176	dipeptidyl peptidase IV inhibitor (DPP IV inhibitor)	dipeptidyl peptidase IV inhibitor	1	FA	[11-12]
3180	dipeptidyl peptidase IV inhibitor (DPP IV inhibitor)	dipeptidyl peptidase IV inhibitor	1	LP	[128-129]
3182	dipeptidyl peptidase IV inhibitor (DPP IV inhibitor)	dipeptidyl peptidase IV inhibitor	2	LL	[6-7],[7-8]

8505	Dipeptidyl peptidase IV inhibitor (DPP IV inhibitor)	dipeptidyl peptidase IV inhibitor	2	SP	[135-136],[144-145]
8526	dipeptidyl peptidase IV inhibitor (DPP IV inhibitor)	dipeptidyl peptidase IV inhibitor	1	RA	[24-25]
8531	dipeptidyl peptidase IV inhibitor (DPP IV inhibitor)	dipeptidyl peptidase IV inhibitor	2	TA	[71-72],[124-125]
8555	dipeptidyl peptidase IV inhibitor (DPP IV inhibitor)	dipeptidyl peptidase IV inhibitor	1	FL	[13-14]
8558	dipeptidyl peptidase IV inhibitor (DPP IV inhibitor)	dipeptidyl peptidase IV inhibitor	1	EK	[116-117]
8560	dipeptidyl peptidase IV inhibitor (DPP IV inhibitor)	dipeptidyl peptidase IV inhibitor	1	SL	[69-70]
8637	dipeptidyl peptidase IV inhibitor (DPP IV inhibitor)	dipeptidyl peptidase IV inhibitor	1	AA	[17-18]
8687	dipeptidyl peptidase IV inhibitor (DPP IV inhibitor)	dipeptidyl peptidase IV inhibitor	1	WS	[147-148]
8759	dipeptidyl peptidase IV inhibitor (DPP IV inhibitor)	dipeptidyl peptidase IV inhibitor	2	AF	[10-11],[12-13]
8762	dipeptidyl peptidase IV inhibitor (DPP IV inhibitor)	dipeptidyl peptidase IV inhibitor	2	AS	[20-21],[125-126]
8763	dipeptidyl peptidase IV inhibitor (DPP IV inhibitor)	dipeptidyl peptidase IV inhibitor	1	AT	[25-26]
8770	dipeptidyl peptidase IV inhibitor (DPP IV inhibitor)	dipeptidyl peptidase IV inhibitor	1	EG	[30-31]
8774	dipeptidyl peptidase IV inhibitor (DPP IV inhibitor)	dipeptidyl peptidase IV inhibitor	1	ET	[123-124]
8775	dipeptidyl peptidase IV inhibitor (DPP IV inhibitor)	dipeptidyl peptidase IV inhibitor	3	EV	[28-29],[60-61],[90-91]
8780	dipeptidyl peptidase IV inhibitor (DPP IV inhibitor)	dipeptidyl peptidase IV inhibitor	1	FR	[113-114]
8781	dipeptidyl peptidase IV inhibitor (DPP IV inhibitor)	dipeptidyl peptidase IV inhibitor	2	GE	[31-32],[115-116]
8784	dipeptidyl peptidase IV inhibitor (DPP IV inhibitor)	dipeptidyl peptidase IV inhibitor	1	GH	[67-68]
8795	dipeptidyl peptidase IV inhibitor (DPP IV inhibitor)	dipeptidyl peptidase IV inhibitor	1	HS	[68-69]
8802	dipeptidyl peptidase IV inhibitor (DPP IV inhibitor)	dipeptidyl peptidase IV inhibitor	1	IL	[15-16]
8804	dipeptidyl peptidase IV inhibitor (DPP IV inhibitor)	dipeptidyl peptidase IV inhibitor	1	IN	[74-75]
8809	dipeptidyl peptidase IV inhibitor (DPP IV inhibitor)	dipeptidyl peptidase IV inhibitor	1	KF	[49-50]
8811	dipeptidyl peptidase IV inhibitor (DPP IV inhibitor)	dipeptidyl peptidase IV inhibitor	1	KH	[51-52]
8813	dipeptidyl peptidase IV inhibitor (DPP IV inhibitor)	dipeptidyl peptidase IV inhibitor	1	KK	[93-94]
8814	dipeptidyl peptidase IV inhibitor (DPP IV inhibitor)	dipeptidyl peptidase IV inhibitor	1	KR	[105-106]
8815	dipeptidyl peptidase IV inhibitor (DPP IV inhibitor)	dipeptidyl peptidase IV inhibitor	1	KS	[63-64]
8821	dipeptidyl peptidase IV inhibitor (DPP IV inhibitor)	dipeptidyl peptidase IV inhibitor	1	LI	[14-15]
8824	dipeptidyl peptidase IV inhibitor (DPP IV inhibitor)	dipeptidyl peptidase IV inhibitor	1	LT	[70-71]
8825	dipeptidyl peptidase IV inhibitor (DPP IV inhibitor)	dipeptidyl peptidase IV inhibitor	1	LV	[4-5]
8837	dipeptidyl peptidase IV inhibitor (DPP IV inhibitor)	dipeptidyl peptidase IV inhibitor	1	MV	[103-104]
8838	dipeptidyl peptidase IV inhibitor (DPP IV inhibitor)	dipeptidyl peptidase IV inhibitor	1	MY	[55-56]
8839	dipeptidyl peptidase IV inhibitor (DPP IV inhibitor)	dipeptidyl peptidase IV inhibitor	1	NA	[19-20]
8845	dipeptidyl peptidase IV inhibitor (DPP IV inhibitor)	dipeptidyl peptidase IV inhibitor	2	NL	[33-34],[99-100]

8848	dipeptidyl peptidase IV inhibitor (DPP IV inhibitor)	dipeptidyl peptidase IV inhibitor	1	NQ	[75-76]
8862	dipeptidyl peptidase IV inhibitor (DPP IV inhibitor)	dipeptidyl peptidase IV inhibitor	1	PS	[136-137]
8866	dipeptidyl peptidase IV inhibitor (DPP IV inhibitor)	dipeptidyl peptidase IV inhibitor	1	PY	[145-146]
8868	dipeptidyl peptidase IV inhibitor (DPP IV inhibitor)	dipeptidyl peptidase IV inhibitor	1	QD	[65-66]
8869	dipeptidyl peptidase IV inhibitor (DPP IV inhibitor)	dipeptidyl peptidase IV inhibitor	4	QE	[59-60],[86-87],[89-90],[119-120]
8870	dipeptidyl peptidase IV inhibitor (DPP IV inhibitor)	dipeptidyl peptidase IV inhibitor	3	QF	[43-44],[112-113],[141-142]
8871	dipeptidyl peptidase IV inhibitor (DPP IV inhibitor)	dipeptidyl peptidase IV inhibitor	2	QG	[110-111],[138-139]
8875	dipeptidyl peptidase IV inhibitor (DPP IV inhibitor)	dipeptidyl peptidase IV inhibitor	1	QN	[98-99]
8876	dipeptidyl peptidase IV inhibitor (DPP IV inhibitor)	dipeptidyl peptidase IV inhibitor	6	QQ	[47-48],[58-59],[78-79],[107-108],[108-109],[109-110]
8877	dipeptidyl peptidase IV inhibitor (DPP IV inhibitor)	dipeptidyl peptidase IV inhibitor	1	QS	[38-39]
8882	dipeptidyl peptidase IV inhibitor (DPP IV inhibitor)	dipeptidyl peptidase IV inhibitor	1	RG	[114-115]
8884	dipeptidyl peptidase IV inhibitor (DPP IV inhibitor)	dipeptidyl peptidase IV inhibitor	1	RI	[73-74]
8887	dipeptidyl peptidase IV inhibitor (DPP IV inhibitor)	dipeptidyl peptidase IV inhibitor	1	RM	[130-131]
8893	dipeptidyl peptidase IV inhibitor (DPP IV inhibitor)	dipeptidyl peptidase IV inhibitor	1	SI	[21-22]
8897	dipeptidyl peptidase IV inhibitor (DPP IV inhibitor)	dipeptidyl peptidase IV inhibitor	1	SY	[148-149]
8912	dipeptidyl peptidase IV inhibitor (DPP IV inhibitor)	dipeptidyl peptidase IV inhibitor	1	TV	[26-27]
8915	dipeptidyl peptidase IV inhibitor (DPP IV inhibitor)	dipeptidyl peptidase IV inhibitor	1	VD	[91-92]
8916	dipeptidyl peptidase IV inhibitor (DPP IV inhibitor)	dipeptidyl peptidase IV inhibitor	2	VE	[27-28],[29-30]
8921	dipeptidyl peptidase IV inhibitor (DPP IV inhibitor)	dipeptidyl peptidase IV inhibitor	1	VK	[104-105]
8922	dipeptidyl peptidase IV inhibitor (DPP IV inhibitor)	dipeptidyl peptidase IV inhibitor	1	VL	[5-6]
8925	dipeptidyl peptidase IV inhibitor (DPP IV inhibitor)	dipeptidyl peptidase IV inhibitor	2	VQ	[57-58],[61-62]
8934	dipeptidyl peptidase IV inhibitor (DPP IV inhibitor)	dipeptidyl peptidase IV inhibitor	1	YE	[122-123]
8944	dipeptidyl peptidase IV inhibitor (DPP IV inhibitor)	dipeptidyl peptidase IV inhibitor	1	YR	[23-24]
8946	dipeptidyl peptidase IV inhibitor (DPP IV inhibitor)	dipeptidyl peptidase IV inhibitor	1	YV	[56-57]
8947	dipeptidyl peptidase IV inhibitor (DPP IV inhibitor)	dipeptidyl peptidase IV inhibitor	1	YW	[146-147]
9484	DPP-III inhibitor	dipeptidyl peptidase III inhibitor	1	YR	[23-24]
9487	DPP-III inhibitor	dipeptidyl peptidase III inhibitor	2	GE	[31-32],[115-116]
9489	DPP-III inhibitor	dipeptidyl peptidase III inhibitor	1	PR	[129-130]
9499	DPP-III inhibitor	dipeptidyl peptidase III inhibitor	1	LA	[16-17]
9500	DPP-III inhibitor	dipeptidyl peptidase III inhibitor	1	FA	[11-12]
9501	DPP-III inhibitor	dipeptidyl peptidase III inhibitor	1	FR	[113-114]
9502	DPP-III inhibitor	dipeptidyl peptidase III inhibitor	1	FL	[13-14]

8249	CaMPDE inhibitor	CaMPDE inhibitor	1	KF	[49-50]
8248	Renin inhibitor	renin inhibitor	1	KF	[49-50]
9431	Renin inhibitor	renin inhibitor	3	QF	[43-44],[112-113],[141-142]
9470	Renin inhibitor	renin inhibitor	1	LY	[121-122]

Table S18. Profile of potential biological activity of fragments of protein Pis v 2.0101.

ID	Name of peptide	Activity	Number	Sequence	Location
3460	Prolyl endopeptidase inhibitor	antiamnestic	2	PG	[117-118],[472-473]
3257	beta-lactokinin	ACE inhibitor	2	RL	[254-255],[459-460]
3258	beta-lactokinin	ACE inhibitor	1	IR	[392-393]
3379	ACE inhibitor	ACE inhibitor	1	AKK	[345-347]
3383	ACE inhibitor	ACE inhibitor	1	IY	[165-166]
3384	ACE inhibitor	ACE inhibitor	4	VF	[81-82],[115-116],[387-388],[437-438]
3393	ACE inhibitor	ACE inhibitor	1	FAP	[470-472]
3404	ACE inhibitor	ACE inhibitor	1	VRP	[146-148]
3421	ACE inhibitor	ACE inhibitor	1	LVL	[173-175]
3489	ACE inhibitor from sake lees	ACE inhibitor	2	RF	[38-39],[339-340]
3492	ACE inhibitor from sake	ACE inhibitor	3	VY	[103-104],[319-320],[368-369]
3502	ACE inhibitor (BSA fr. 221-222)	ACE inhibitor	1	FP	[116-117]
3532	ACE inhibitor	ACE inhibitor	1	GY	[2-3]
3537	ACE inhibitor	ACE inhibitor	1	PR	[322-323]
3541	ACE inhibitor	ACE inhibitor	1	LSP	[275-277]
3551	ACE inhibitor (from bovine beta-Lg)	ACE inhibitor	1	LF	[16-17]
7502	ACE inhibitor	ACE inhibitor	1	IVR	[265-267]
7513	ACE inhibitor from Alaskan pollack skin	ACE inhibitor	1	PL	[442-443]
7541	ACE inhibitor from wheat germ hydrolysate	ACE inhibitor	1	IVY	[367-369]
7558	ACE inhibitor from buckwheat	ACE inhibitor	1	VK	[407-408]
7559	ACE inhibitor from buckwheat	ACE inhibitor	1	PSY	[94-96]
7562	ACE inhibitor from soy hydrolysate	ACE inhibitor	1	IA	[155-156]
7570	ACE inhibitor from chicken muscle	ACE inhibitor	1	LAP	[356-358]
7582	ACE inhibitor	ACE inhibitor	1	RP	[147-148]
7583	ACE inhibitor	ACE inhibitor	1	AF	[235-236]
7584	ACE inhibitor	ACE inhibitor	3	AP	[99-100],[357-358],[471-472]
7585	ACE inhibitor	ACE inhibitor	5	LA	[178-179],[241-242],[251-252],[356-357],[431-432]
7586	ACE inhibitor	ACE inhibitor	3	KR	[54-55],[259-260],[408-409]
7587	ACE inhibitor	ACE inhibitor	2	VP	[93-94],[400-401]

7588	ACE inhibitor	ACE inhibitor	3	RA	[316-317],[409-410],[439-440]
7591	ACE inhibitor	ACE inhibitor	1	GF	[12-13]
7592	ACE inhibitor	ACE inhibitor	2	FR	[82-83],[438-439]
7593	ACE inhibitor	ACE inhibitor	1	IF	[469-470]
7594	ACE inhibitor	ACE inhibitor	1	VG	[181-182]
7596	ACE inhibitor	ACE inhibitor	2	GI	[110-111],[263-264]
7598	ACE inhibitor	ACE inhibitor	1	GA	[113-114]
7599	ACE inhibitor	ACE inhibitor	3	GL	[90-91],[297-298],[424-425]
7600	ACE inhibitor	ACE inhibitor	3	AG	[61-62],[159-160],[432-433]
7603	ACE inhibitor	ACE inhibitor	3	GR	[325-326],[375-376],[433-434]
7604	ACE inhibitor	ACE inhibitor	1	KG	[347-348]
7606	ACE inhibitor	ACE inhibitor	2	DA	[353-354],[456-457]
7607	ACE inhibitor	ACE inhibitor	4	GS	[108-109],[199-200],[207-208],[473-474]
7608	ACE inhibitor	ACE inhibitor	3	GV	[62-63],[160-161],[348-349]
7609	ACE inhibitor	ACE inhibitor	1	MG	[1-2]
7610	ACE inhibitor	ACE inhibitor	5	GQ	[169-170],[209-210],[223-224],[226-227],[395-396]
7614	ACE inhibitor	ACE inhibitor	1	HG	[112-113]
7615	ACE inhibitor	ACE inhibitor	2	GE	[140-141],[384-385]
7616	ACE inhibitor	ACE inhibitor	2	GG	[198-199],[324-325]
7617	ACE inhibitor	ACE inhibitor	3	QG	[107-108],[206-207],[225-226]
7618	ACE inhibitor	ACE inhibitor	3	SG	[109-110],[139-140],[208-209]
7619	ACE inhibitor	ACE inhibitor	2	LG	[11-12],[197-198]
7620	ACE inhibitor	ACE inhibitor	1	GD	[152-153]
7622	ACE inhibitor	ACE inhibitor	2	EG	[151-152],[394-395]
7623	ACE inhibitor	ACE inhibitor	1	EA	[60-61]
7624	ACE inhibitor	ACE inhibitor	5	NG	[168-169],[296-297],[374-375],[383-384],[423-424]
7625	ACE inhibitor	ACE inhibitor	2	PG	[117-118],[472-473]
7628	ACE inhibitor from k-CN (fr. 67-68)	ACE inhibitor	2	VR	[146-147],[266-267]
7635	ACE inhibitor from k-CN (fr. 51-53)	ACE inhibitor	1	VAV	[79-81]
7680	ACE inhibitor from pea vicilin	ACE inhibitor	2	QK	[144-145],[256-257]
7683	ACE inhibitor from garlic	ACE inhibitor	1	NF	[403-404]
7684	ACE inhibitor from garlic	ACE inhibitor	1	SY	[95-96]

7685	ACE inhibitor from garlic	ACE inhibitor	5	SF	[8-9],[20-21],[244-245],[419-420],[449-450]
7691	ACE inhibitor from wakame	ACE inhibitor	1	KY	[308-309]
7692	ACE inhibitor	ACE inhibitor	2	KF	[194-195],[414-415]
7693	ACE inhibitor from wakame	ACE inhibitor	1	KL	[172-173]
7741	ACE inhibitor	ACE inhibitor	5	RR	[55-56],[221-222],[253-254],[292-293],[458-459]
7742	ACE inhibitor	ACE inhibitor	2	AR	[252-253],[457-458]
7752	ACE inhibitor from shark meat hydrolysate	ACE inhibitor	1	EY	[285-286]
7807	ACE inhibitor from caprine b-Lg	ACE inhibitor	1	LLF	[15-17]
7826	ACE inhibitor	ACE inhibitor	4	EI	[204-205],[239-240],[391-392],[494-495]
7827	ACE inhibitor	ACE inhibitor	2	IE	[24-25],[57-58]
7828	ACE inhibitor	ACE inhibitor	1	EV	[273-274]
7830	ACE inhibitor	ACE inhibitor	1	TE	[64-65]
7831	ACE inhibitor	ACE inhibitor	3	LQ	[74-75],[255-256],[341-342]
7832	ACE inhibitor	ACE inhibitor	2	LN	[48-49],[333-334]
7834	ACE inhibitor	ACE inhibitor	2	TQ	[41-42],[249-250]
7835	ACE inhibitor	ACE inhibitor	2	AH	[162-163],[364-365]
7837	ACE inhibitor	ACE inhibitor	2	PQ	[201-202],[401-402]
7838	ACE inhibitor	ACE inhibitor	1	EW	[416-417]
7840	ACE inhibitor	ACE inhibitor	1	EK	[258-259]
7841	ACE inhibitor	ACE inhibitor	2	KE	[257-258],[461-462]
7843	ACE inhibitor	ACE inhibitor	2	PH	[277-278],[358-359]
8182	ACE Inhibitor	ACE inhibitor	1	ALEP	[50-53]
8185	ACE inhibitor	ACE inhibitor	2	TF	[122-123],[301-302]
8193	ACE inhibitor	ACE inhibitor	1	AI	[354-355]
8402	ACE inhibitor	ACE inhibitor	1	LVY	[102-104]
8951	ACE inhibitor	ACE inhibitor	3	AV	[80-81],[114-115],[405-406]
9029	ACE inhibitor	ACE inhibitor	2	ALP	[156-158],[440-442]
9031	ACE inhibitor	ACE inhibitor	1	LEE	[298-300]
9076	ACE inhibitor	ACE inhibitor	2	FQ	[39-40],[123-124]
9077	ACE inhibitor	ACE inhibitor	1	YV	[104-105]
9078	ACE inhibitor	ACE inhibitor	1	YE	[286-287]
9079	ACE inhibitor	ACE inhibitor	4	IL	[232-233],[240-241],[337-338],[355-356]

9173	ACE inhibitor	ACE inhibitor	5	RG	[89-90],[222-223],[262-263],[323-324],[372-373]
9185	ACE inhibitor	ACE inhibitor	3	YN	[166-167],[309-310],[320-321]
9196	ACE inhibitor	ACE inhibitor	1	AVV	[405-407]
9213	ACE inhibitor	ACE inhibitor	2	LR	[192-193],[338-339]
9468	ACE inhibitor	ACE inhibitor	1	RALP	[439-442]
9708	ACE inhibitor	ACE inhibitor	1	LPILR	[335-339]
9730	ACE inhibitor	ACE inhibitor	1	NPR	[321-323]
9742	ACE inhibitor	ACE inhibitor	1	EKR	[258-260]
3285	Antithrombotic peptide	antithrombotic	2	PG	[117-118],[472-473]
3354	Antithrombotic peptide	antithrombotic	2	DEE	[237-239],[389-391]
3351	Stimulating vasoactive substance release	stimulating	1	EEE	[287-289]
3355	Stimulating vasoactive substance release	stimulating	1	SSS	[474-476]
3356	Stimulating vasoactive substance release	stimulating	1	LLL	[14-16]
8320	Glucose uptake stimulating peptide	stimulating	4	VL	[174-175],[196-197],[274-275],[349-350]
8321	Glucose uptake stimulating peptide	stimulating	5	LV	[92-93],[102-103],[173-174],[175-176],[397-398]
8322	Glucose uptake stimulating peptide	stimulating	3	IV	[265-266],[367-368],[379-380]
8323	Glucose uptake stimulating peptide	stimulating	4	IL	[232-233],[240-241],[337-338],[355-356]
8325	Glucose uptake stimulating peptide	stimulating	1	II	[264-265]
8326	Glucose uptake stimulating peptide	stimulating	4	LL	[6-7],[14-15],[15-16],[91-92]
8329	Stimulating vasoactive substance release	stimulating	8	EE	[71-72],[125-126],[238-239],[287-288],[288-289],[299-300],[390-391],[491-492]
8330	Stimulating vasoactive substance release	stimulating	6	SE	[59-60],[133-134],[184-185],[381-382],[465-466],[482-483]
3061	Immunostimulating peptide	immunostimulating	1	GFL	[12-14]
2890	neuropeptide	neuropeptide	5	GQ	[169-170],[209-210],[223-224],[226-227],[395-396]
8310	Anxiolytic peptide	neuropeptide	1	YL	[191-192]
2737	Peptide regulating phosphoinositol metabolism	regulating	1	GFL	[12-14]
2754	peptide regulating the stomach mucosal membrane activity	regulating	2	PG	[117-118],[472-473]
8318	Dvl protein binding	anticancer	1	VVV	[398-400]
3305		antioxidative	1	LH	[350-351]
7873	peptide from soybean protein isolates: beta-conglycinin and glycinin	antioxidative	1	IY	[165-166]
7886	peptide derived from egg white albumin	antioxidative	2	AH	[162-163],[364-365]
7888	antioxidative peptide	antioxidative	1	EL	[101-102]

7999	synthetic peptide	antioxidative	1	LHR	[350-352]
8033	synthetic peptide	antioxidative	1	PHR	[277-279]
8037	synthetic peptide	antioxidative	1	PHW	[358-360]
8067	synthetic peptide	antioxidative	1	RHT	[83-85]
8139	synthetic peptide	antioxidative	1	PEL	[100-102]
8215	Antioxidative peptide	antioxidative	1	IR	[392-393]
8217	Antioxidative peptide	antioxidative	2	LK	[307-308],[460-461]
8224	antioxidative peptide	antioxidative	3	VY	[103-104],[319-320],[368-369]
9342	Antioxidative peptide	antioxidative	1	FC	[302-303]
9361	Antioxidative peptide	antioxidative	1	VYV	[103-105]
9716	Antioxidative peptide	antioxidative	1	LPILR	[335-339]
3751		bacterial permease ligand	1	KK	[346-347]
8661	Hypotensive peptide	hypotensive	1	LPILR	[335-339]
4005		activating ubiquitin-mediated proteolysis	3	RA	[316-317],[409-410],[439-440]
4006	Ubiqitin-mediated proteolysis activating peptide	activating ubiquitin-mediated proteolysis	5	LA	[178-179],[241-242],[251-252],[356-357],[431-432]
9580	Hypolipidemic peptide	hypolipidemic	1	EF	[65-66]
9650	Alpha-glucosidase inhibitor	alpha-glucosidase inhibitor	1	EA	[60-61]
9694	Alpha-glucosidase inhibitor	alpha-glucosidase inhibitor	2	PE	[100-101],[120-121]
9695	Alpha-glucosidase inhibitor	alpha-glucosidase inhibitor	2	AD	[179-180],[317-318]
3172	dipeptidyl peptidase IV inhibitor (DPP IV inhibitor)	dipeptidyl peptidase IV inhibitor	3	VA	[79-80],[161-162],[176-177]
3175	dipeptidyl peptidase IV inhibitor (DPP IV inhibitor)	dipeptidyl peptidase IV inhibitor	5	LA	[178-179],[241-242],[251-252],[356-357],[431-432]
3176	dipeptidyl peptidase IV inhibitor (DPP IV inhibitor)	dipeptidyl peptidase IV inhibitor	3	FA	[21-22],[404-405],[470-471]
3177	dipeptidyl peptidase IV inhibitor (DPP IV inhibitor)	dipeptidyl peptidase IV inhibitor	3	AP	[99-100],[357-358],[471-472]
3179	dipeptidyl peptidase IV inhibitor (DPP IV inhibitor)	dipeptidyl peptidase IV inhibitor	1	PA	[158-159]
3180	dipeptidyl peptidase IV inhibitor (DPP IV inhibitor)	dipeptidyl peptidase IV inhibitor	3	LP	[157-158],[335-336],[441-442]
3181	dipeptidyl peptidase IV inhibitor (DPP IV inhibitor)	dipeptidyl peptidase IV inhibitor	2	VP	[93-94],[400-401]
3182	dipeptidyl peptidase IV inhibitor (DPP IV inhibitor)	dipeptidyl peptidase IV inhibitor	4	LL	[6-7],[14-15],[15-16],[91-92]
3183	dipeptidyl peptidase IV inhibitor (DPP IV inhibitor)	dipeptidyl peptidase IV inhibitor	5	VV	[27-28],[105-106],[398-399],[399-400],[406-407]
8500	dipeptidyl peptidase IV inhibitor (DPP IV inhibitor)	dipeptidyl peptidase IV inhibitor	1	APG	[471-473]
8505	Dipeptidyl peptidase IV inhibitor (DPP IV inhibitor)	dipeptidyl peptidase IV inhibitor	2	SP	[200-201],[276-277]
8506	dipeptidyl peptidase IV inhibitor (DPP IV inhibitor)	dipeptidyl peptidase IV inhibitor	1	FP	[116-117]

8518	dipeptidyl peptidase IV inhibitor (DPP IV inhibitor)	dipeptidyl peptidase IV inhibitor	1	RP	[147-148]
8524	dipeptidyl peptidase IV inhibitor (DPP IV inhibitor)	dipeptidyl peptidase IV inhibitor	1	GA	[113-114]
8525	dipeptidyl peptidase IV inhibitor (DPP IV inhibitor)	dipeptidyl peptidase IV inhibitor	1	IA	[155-156]
8526	dipeptidyl peptidase IV inhibitor (DPP IV inhibitor)	dipeptidyl peptidase IV inhibitor	3	RA	[316-317],[409-410],[439-440]
8529	dipeptidyl peptidase IV inhibitor (DPP IV inhibitor)	dipeptidyl peptidase IV inhibitor	1	EP	[52-53]
8530	dipeptidyl peptidase IV inhibitor (DPP IV inhibitor)	dipeptidyl peptidase IV inhibitor	1	NP	[321-322]
8555	dipeptidyl peptidase IV inhibitor (DPP IV inhibitor)	dipeptidyl peptidase IV inhibitor	2	FL	[13-14],[340-341]
8556	dipeptidyl peptidase IV inhibitor (DPP IV inhibitor)	dipeptidyl peptidase IV inhibitor	1	WV	[417-418]
8558	dipeptidyl peptidase IV inhibitor (DPP IV inhibitor)	dipeptidyl peptidase IV inhibitor	1	EK	[258-259]
8559	dipeptidyl peptidase IV inhibitor (DPP IV inhibitor)	dipeptidyl peptidase IV inhibitor	5	AL	[50-51],[156-157],[177-178],[332-333],[440-441]
8560	dipeptidyl peptidase IV inhibitor (DPP IV inhibitor)	dipeptidyl peptidase IV inhibitor	2	SL	[5-6],[10-11]
8561	dipeptidyl peptidase IV inhibitor (DPP IV inhibitor)	dipeptidyl peptidase IV inhibitor	3	GL	[90-91],[297-298],[424-425]
8594	dipeptidyl peptidase IV inhibitor (DPP IV inhibitor)	dipeptidyl peptidase IV inhibitor	2	VR	[146-147],[266-267]
8616	dipeptidyl peptidase IV inhibitor (DPP IV inhibitor)	dipeptidyl peptidase IV inhibitor	1	LPL	[441-443]
8638	dipeptidyl peptidase IV inhibitor (DPP IV inhibitor)	dipeptidyl peptidase IV inhibitor	1	PL	[442-443]
8647	dipeptidyl peptidase IV inhibitor (DPP IV inhibitor)	dipeptidyl peptidase IV inhibitor	1	ILAP	[355-358]
8679	dipeptidyl peptidase IV inhibitor (DPP IV inhibitor)	dipeptidyl peptidase IV inhibitor	1	WI	[164-165]
8680	dipeptidyl peptidase IV inhibitor (DPP IV inhibitor)	dipeptidyl peptidase IV inhibitor	1	WN	[360-361]
8757	dipeptidyl peptidase IV inhibitor (DPP IV inhibitor)	dipeptidyl peptidase IV inhibitor	2	AD	[179-180],[317-318]
8759	dipeptidyl peptidase IV inhibitor (DPP IV inhibitor)	dipeptidyl peptidase IV inhibitor	1	AF	[235-236]
8760	dipeptidyl peptidase IV inhibitor (DPP IV inhibitor)	dipeptidyl peptidase IV inhibitor	3	AG	[61-62],[159-160],[432-433]
8761	dipeptidyl peptidase IV inhibitor (DPP IV inhibitor)	dipeptidyl peptidase IV inhibitor	2	AH	[162-163],[364-365]
8762	dipeptidyl peptidase IV inhibitor (DPP IV inhibitor)	dipeptidyl peptidase IV inhibitor	1	AS	[410-411]
8764	dipeptidyl peptidase IV inhibitor (DPP IV inhibitor)	dipeptidyl peptidase IV inhibitor	3	AV	[80-81],[114-115],[405-406]
8766	dipeptidyl peptidase IV inhibitor (DPP IV inhibitor)	dipeptidyl peptidase IV inhibitor	1	DN	[97-98]
8767	dipeptidyl peptidase IV inhibitor (DPP IV inhibitor)	dipeptidyl peptidase IV inhibitor	1	DP	[313-314]
8768	dipeptidyl peptidase IV inhibitor (DPP IV inhibitor)	dipeptidyl peptidase IV inhibitor	2	DQ	[68-69],[189-190]
8770	dipeptidyl peptidase IV inhibitor (DPP IV inhibitor)	dipeptidyl peptidase IV inhibitor	2	EG	[151-152],[394-395]
8772	dipeptidyl peptidase IV inhibitor (DPP IV inhibitor)	dipeptidyl peptidase IV inhibitor	4	EI	[204-205],[239-240],[391-392],[494-495]
8773	dipeptidyl peptidase IV inhibitor (DPP IV inhibitor)	dipeptidyl peptidase IV inhibitor	4	ES	[58-59],[126-127],[385-386],[462-463]
8774	dipeptidyl peptidase IV inhibitor (DPP IV inhibitor)	dipeptidyl peptidase IV inhibitor	3	ET	[121-122],[300-301],[466-467]
8775	dipeptidyl peptidase IV inhibitor (DPP IV inhibitor)	dipeptidyl peptidase IV inhibitor	1	EV	[273-274]

8776	dipeptidyl peptidase IV inhibitor (DPP IV inhibitor)	dipeptidyl peptidase IV inhibitor	1	EW	[416-417]
8777	dipeptidyl peptidase IV inhibitor (DPP IV inhibitor)	dipeptidyl peptidase IV inhibitor	1	EY	[285-286]
8778	dipeptidyl peptidase IV inhibitor (DPP IV inhibitor)	dipeptidyl peptidase IV inhibitor	1	FN	[245-246]
8779	dipeptidyl peptidase IV inhibitor (DPP IV inhibitor)	dipeptidyl peptidase IV inhibitor	2	FQ	[39-40],[123-124]
8780	dipeptidyl peptidase IV inhibitor (DPP IV inhibitor)	dipeptidyl peptidase IV inhibitor	2	FR	[82-83],[438-439]
8781	dipeptidyl peptidase IV inhibitor (DPP IV inhibitor)	dipeptidyl peptidase IV inhibitor	2	GE	[140-141],[384-385]
8782	dipeptidyl peptidase IV inhibitor (DPP IV inhibitor)	dipeptidyl peptidase IV inhibitor	1	GF	[12-13]
8783	dipeptidyl peptidase IV inhibitor (DPP IV inhibitor)	dipeptidyl peptidase IV inhibitor	2	GG	[198-199],[324-325]
8785	dipeptidyl peptidase IV inhibitor (DPP IV inhibitor)	dipeptidyl peptidase IV inhibitor	2	GI	[110-111],[263-264]
8786	dipeptidyl peptidase IV inhibitor (DPP IV inhibitor)	dipeptidyl peptidase IV inhibitor	3	GV	[62-63],[160-161],[348-349]
8788	dipeptidyl peptidase IV inhibitor (DPP IV inhibitor)	dipeptidyl peptidase IV inhibitor	1	GY	[2-3]
8794	dipeptidyl peptidase IV inhibitor (DPP IV inhibitor)	dipeptidyl peptidase IV inhibitor	2	HR	[278-279],[351-352]
8795	dipeptidyl peptidase IV inhibitor (DPP IV inhibitor)	dipeptidyl peptidase IV inhibitor	1	HS	[365-366]
8796	dipeptidyl peptidase IV inhibitor (DPP IV inhibitor)	dipeptidyl peptidase IV inhibitor	1	HT	[84-85]
8798	dipeptidyl peptidase IV inhibitor (DPP IV inhibitor)	dipeptidyl peptidase IV inhibitor	2	HW	[163-164],[359-360]
8800	dipeptidyl peptidase IV inhibitor (DPP IV inhibitor)	dipeptidyl peptidase IV inhibitor	2	IH	[111-112],[495-496]
8801	dipeptidyl peptidase IV inhibitor (DPP IV inhibitor)	dipeptidyl peptidase IV inhibitor	1	II	[264-265]
8802	dipeptidyl peptidase IV inhibitor (DPP IV inhibitor)	dipeptidyl peptidase IV inhibitor	4	IL	[232-233],[240-241],[337-338],[355-356]
8804	dipeptidyl peptidase IV inhibitor (DPP IV inhibitor)	dipeptidyl peptidase IV inhibitor	1	IN	[311-312]
8805	dipeptidyl peptidase IV inhibitor (DPP IV inhibitor)	dipeptidyl peptidase IV inhibitor	5	IQ	[45-46],[86-87],[149-150],[205-206],[377-378]
8806	dipeptidyl peptidase IV inhibitor (DPP IV inhibitor)	dipeptidyl peptidase IV inhibitor	1	IR	[392-393]
8808	dipeptidyl peptidase IV inhibitor (DPP IV inhibitor)	dipeptidyl peptidase IV inhibitor	2	KE	[257-258],[461-462]
8809	dipeptidyl peptidase IV inhibitor (DPP IV inhibitor)	dipeptidyl peptidase IV inhibitor	2	KF	[194-195],[414-415]
8810	dipeptidyl peptidase IV inhibitor (DPP IV inhibitor)	dipeptidyl peptidase IV inhibitor	1	KG	[347-348]
8813	dipeptidyl peptidase IV inhibitor (DPP IV inhibitor)	dipeptidyl peptidase IV inhibitor	1	KK	[346-347]
8814	dipeptidyl peptidase IV inhibitor (DPP IV inhibitor)	dipeptidyl peptidase IV inhibitor	3	KR	[54-55],[259-260],[408-409]
8815	dipeptidyl peptidase IV inhibitor (DPP IV inhibitor)	dipeptidyl peptidase IV inhibitor	1	KS	[479-480]
8816	dipeptidyl peptidase IV inhibitor (DPP IV inhibitor)	dipeptidyl peptidase IV inhibitor	1	KT	[421-422]
8817	dipeptidyl peptidase IV inhibitor (DPP IV inhibitor)	dipeptidyl peptidase IV inhibitor	1	KV	[145-146]
8819	dipeptidyl peptidase IV inhibitor (DPP IV inhibitor)	dipeptidyl peptidase IV inhibitor	1	KY	[308-309]
8820	dipeptidyl peptidase IV inhibitor (DPP IV inhibitor)	dipeptidyl peptidase IV inhibitor	1	LH	[350-351]
8823	dipeptidyl peptidase IV inhibitor (DPP IV inhibitor)	dipeptidyl peptidase IV inhibitor	2	LN	[48-49],[333-334]

8825	dipeptidyl peptidase IV inhibitor (DPP IV inhibitor)	dipeptidyl peptidase IV inhibitor	5	LV	[92-93],[102-103],[173-174],[175-176],[397-398]
8828	dipeptidyl peptidase IV inhibitor (DPP IV inhibitor)	dipeptidyl peptidase IV inhibitor	1	MG	[1-2]
8839	dipeptidyl peptidase IV inhibitor (DPP IV inhibitor)	dipeptidyl peptidase IV inhibitor	4	NA	[49-50],[98-99],[331-332],[363-364]
8840	dipeptidyl peptidase IV inhibitor (DPP IV inhibitor)	dipeptidyl peptidase IV inhibitor	1	ND	[312-313]
8841	dipeptidyl peptidase IV inhibitor (DPP IV inhibitor)	dipeptidyl peptidase IV inhibitor	1	NE	[70-71]
8842	dipeptidyl peptidase IV inhibitor (DPP IV inhibitor)	dipeptidyl peptidase IV inhibitor	1	NF	[403-404]
8843	dipeptidyl peptidase IV inhibitor (DPP IV inhibitor)	dipeptidyl peptidase IV inhibitor	5	NG	[168-169],[296-297],[374-375],[383-384],[423-424]
8845	dipeptidyl peptidase IV inhibitor (DPP IV inhibitor)	dipeptidyl peptidase IV inhibitor	2	NL	[47-48],[334-335]
8847	dipeptidyl peptidase IV inhibitor (DPP IV inhibitor)	dipeptidyl peptidase IV inhibitor	2	NN	[167-168],[230-231]
8848	dipeptidyl peptidase IV inhibitor (DPP IV inhibitor)	dipeptidyl peptidase IV inhibitor	1	NQ	[186-187]
8851	dipeptidyl peptidase IV inhibitor (DPP IV inhibitor)	dipeptidyl peptidase IV inhibitor	2	NV	[78-79],[361-362]
8855	dipeptidyl peptidase IV inhibitor (DPP IV inhibitor)	dipeptidyl peptidase IV inhibitor	2	PG	[117-118],[472-473]
8856	dipeptidyl peptidase IV inhibitor (DPP IV inhibitor)	dipeptidyl peptidase IV inhibitor	2	PH	[277-278],[358-359]
8857	dipeptidyl peptidase IV inhibitor (DPP IV inhibitor)	dipeptidyl peptidase IV inhibitor	2	PI	[148-149],[336-337]
8858	dipeptidyl peptidase IV inhibitor (DPP IV inhibitor)	dipeptidyl peptidase IV inhibitor	1	PK	[53-54]
8861	dipeptidyl peptidase IV inhibitor (DPP IV inhibitor)	dipeptidyl peptidase IV inhibitor	2	PQ	[201-202],[401-402]
8862	dipeptidyl peptidase IV inhibitor (DPP IV inhibitor)	dipeptidyl peptidase IV inhibitor	2	PS	[94-95],[314-315]
8869	dipeptidyl peptidase IV inhibitor (DPP IV inhibitor)	dipeptidyl peptidase IV inhibitor	7	QE	[124-125],[150-151],[203-204],[269-270],[280-281],[282-283],[284-285]
8871	dipeptidyl peptidase IV inhibitor (DPP IV inhibitor)	dipeptidyl peptidase IV inhibitor	3	QG	[107-108],[206-207],[225-226]
8872	dipeptidyl peptidase IV inhibitor (DPP IV inhibitor)	dipeptidyl peptidase IV inhibitor	1	QH	[142-143]
8873	dipeptidyl peptidase IV inhibitor (DPP IV inhibitor)	dipeptidyl peptidase IV inhibitor	3	QI	[23-24],[44-45],[378-379]
8874	dipeptidyl peptidase IV inhibitor (DPP IV inhibitor)	dipeptidyl peptidase IV inhibitor	6	QL	[73-74],[187-188],[250-251],[342-343],[396-397],[430-431]
8875	dipeptidyl peptidase IV inhibitor (DPP IV inhibitor)	dipeptidyl peptidase IV inhibitor	3	QN	[46-47],[69-70],[402-403]
8876	dipeptidyl peptidase IV inhibitor (DPP IV inhibitor)	dipeptidyl peptidase IV inhibitor	8	QQ	[31-32],[34-35],[35-36],[36-37],[137-138],[202-203],[224-225],[227-228]
8877	dipeptidyl peptidase IV inhibitor (DPP IV inhibitor)	dipeptidyl peptidase IV inhibitor	10	QS	[87-88],[128-129],[138-139],[170-171],[210-211],[216-217],[218-219],[228-229],[243-244],[481-482]
8878	dipeptidyl peptidase IV inhibitor (DPP IV inhibitor)	dipeptidyl peptidase IV inhibitor	2	QT	[40-41],[427-428]
8879	dipeptidyl peptidase IV inhibitor (DPP IV inhibitor)	dipeptidyl peptidase IV inhibitor	1	QV	[26-27]
8881	dipeptidyl peptidase IV inhibitor (DPP IV inhibitor)	dipeptidyl peptidase IV inhibitor	1	QY	[190-191]
8882	dipeptidyl peptidase IV inhibitor (DPP IV inhibitor)	dipeptidyl peptidase IV inhibitor	5	RG	[89-90],[222-223],[262-263],[323-324],[372-373]
8883	dipeptidyl peptidase IV inhibitor (DPP IV inhibitor)	dipeptidyl peptidase IV inhibitor	1	RH	[83-84]
8884	dipeptidyl peptidase IV inhibitor (DPP IV inhibitor)	dipeptidyl peptidase IV inhibitor	2	RI	[56-57],[376-377]

8885	dipeptidyl peptidase IV inhibitor (DPP IV inhibitor)	dipeptidyl peptidase IV inhibitor	2	RK	[193-194],[478-479]
8886	dipeptidyl peptidase IV inhibitor (DPP IV inhibitor)	dipeptidyl peptidase IV inhibitor	2	RL	[254-255],[459-460]
8888	dipeptidyl peptidase IV inhibitor (DPP IV inhibitor)	dipeptidyl peptidase IV inhibitor	1	RN	[295-296]
8889	dipeptidyl peptidase IV inhibitor (DPP IV inhibitor)	dipeptidyl peptidase IV inhibitor	5	RR	[55-56],[221-222],[253-254],[292-293],[458-459]
8891	dipeptidyl peptidase IV inhibitor (DPP IV inhibitor)	dipeptidyl peptidase IV inhibitor	5	SF	[8-9],[20-21],[244-245],[419-420],[449-450]
8893	dipeptidyl peptidase IV inhibitor (DPP IV inhibitor)	dipeptidyl peptidase IV inhibitor	1	SI	[366-367]
8894	dipeptidyl peptidase IV inhibitor (DPP IV inhibitor)	dipeptidyl peptidase IV inhibitor	1	SK	[171-172]
8895	dipeptidyl peptidase IV inhibitor (DPP IV inhibitor)	dipeptidyl peptidase IV inhibitor	3	SV	[329-330],[386-387],[436-437]
8897	dipeptidyl peptidase IV inhibitor (DPP IV inhibitor)	dipeptidyl peptidase IV inhibitor	1	SY	[95-96]
8899	dipeptidyl peptidase IV inhibitor (DPP IV inhibitor)	dipeptidyl peptidase IV inhibitor	1	TE	[64-65]
8900	dipeptidyl peptidase IV inhibitor (DPP IV inhibitor)	dipeptidyl peptidase IV inhibitor	2	TF	[122-123],[301-302]
8903	dipeptidyl peptidase IV inhibitor (DPP IV inhibitor)	dipeptidyl peptidase IV inhibitor	2	TI	[85-86],[468-469]
8905	dipeptidyl peptidase IV inhibitor (DPP IV inhibitor)	dipeptidyl peptidase IV inhibitor	1	TL	[306-307]
8906	dipeptidyl peptidase IV inhibitor (DPP IV inhibitor)	dipeptidyl peptidase IV inhibitor	1	TM	[304-305]
8907	dipeptidyl peptidase IV inhibitor (DPP IV inhibitor)	dipeptidyl peptidase IV inhibitor	1	TN	[422-423]
8908	dipeptidyl peptidase IV inhibitor (DPP IV inhibitor)	dipeptidyl peptidase IV inhibitor	2	TQ	[41-42],[249-250]
8909	dipeptidyl peptidase IV inhibitor (DPP IV inhibitor)	dipeptidyl peptidase IV inhibitor	1	TR	[371-372]
8910	dipeptidyl peptidase IV inhibitor (DPP IV inhibitor)	dipeptidyl peptidase IV inhibitor	1	TS	[428-429]
8911	dipeptidyl peptidase IV inhibitor (DPP IV inhibitor)	dipeptidyl peptidase IV inhibitor	1	TT	[467-468]
8917	dipeptidyl peptidase IV inhibitor (DPP IV inhibitor)	dipeptidyl peptidase IV inhibitor	4	VF	[81-82],[115-116],[387-388],[437-438]
8918	dipeptidyl peptidase IV inhibitor (DPP IV inhibitor)	dipeptidyl peptidase IV inhibitor	1	VG	[181-182]
8920	dipeptidyl peptidase IV inhibitor (DPP IV inhibitor)	dipeptidyl peptidase IV inhibitor	2	VI	[154-155],[445-446]
8921	dipeptidyl peptidase IV inhibitor (DPP IV inhibitor)	dipeptidyl peptidase IV inhibitor	1	VK	[407-408]
8922	dipeptidyl peptidase IV inhibitor (DPP IV inhibitor)	dipeptidyl peptidase IV inhibitor	4	VL	[174-175],[196-197],[274-275],[349-350]
8924	dipeptidyl peptidase IV inhibitor (DPP IV inhibitor)	dipeptidyl peptidase IV inhibitor	3	VN	[28-29],[330-331],[362-363]
8925	dipeptidyl peptidase IV inhibitor (DPP IV inhibitor)	dipeptidyl peptidase IV inhibitor	2	VQ	[106-107],[268-269]
8926	dipeptidyl peptidase IV inhibitor (DPP IV inhibitor)	dipeptidyl peptidase IV inhibitor	4	VS	[327-328],[380-381],[418-419],[435-436]
8927	dipeptidyl peptidase IV inhibitor (DPP IV inhibitor)	dipeptidyl peptidase IV inhibitor	1	VT	[63-64]
8929	dipeptidyl peptidase IV inhibitor (DPP IV inhibitor)	dipeptidyl peptidase IV inhibitor	3	VY	[103-104],[319-320],[368-369]
8930	dipeptidyl peptidase IV inhibitor (DPP IV inhibitor)	dipeptidyl peptidase IV inhibitor	1	WD	[67-68]
8933	dipeptidyl peptidase IV inhibitor (DPP IV inhibitor)	dipeptidyl peptidase IV inhibitor	1	YD	[96-97]
8934	dipeptidyl peptidase IV inhibitor (DPP IV inhibitor)	dipeptidyl peptidase IV inhibitor	1	YE	[286-287]

8938	dipeptidyl peptidase IV inhibitor (DPP IV inhibitor)	dipeptidyl peptidase IV inhibitor	1	YI	[369-370]
8940	dipeptidyl peptidase IV inhibitor (DPP IV inhibitor)	dipeptidyl peptidase IV inhibitor	1	YL	[191-192]
8942	dipeptidyl peptidase IV inhibitor (DPP IV inhibitor)	dipeptidyl peptidase IV inhibitor	3	YN	[166-167],[309-310],[320-321]
8945	dipeptidyl peptidase IV inhibitor (DPP IV inhibitor)	dipeptidyl peptidase IV inhibitor	1	YS	[3-4]
8946	dipeptidyl peptidase IV inhibitor (DPP IV inhibitor)	dipeptidyl peptidase IV inhibitor	1	YV	[104-105]
9478	DPP-III inhibitor	dipeptidyl peptidase III inhibitor	2	LR	[192-193],[338-339]
9482	DPP-III inhibitor	dipeptidyl peptidase III inhibitor	1	YL	[191-192]
9485	DPP-III inhibitor	dipeptidyl peptidase III inhibitor	5	RR	[55-56],[221-222],[253-254],[292-293],[458-459]
9486	DPP-III inhibitor	dipeptidyl peptidase III inhibitor	2	TF	[122-123],[301-302]
9487	DPP-III inhibitor	dipeptidyl peptidase III inhibitor	2	GE	[140-141],[384-385]
9488	DPP-III inhibitor	dipeptidyl peptidase III inhibitor	1	GF	[12-13]
9489	DPP-III inhibitor	dipeptidyl peptidase III inhibitor	1	PR	[322-323]
9490	DPP-III inhibitor	dipeptidyl peptidase III inhibitor	2	RF	[38-39],[339-340]
9491	DPP-III inhibitor	dipeptidyl peptidase III inhibitor	3	RV	[267-268],[326-327],[434-435]
9492	DPP-III inhibitor	dipeptidyl peptidase III inhibitor	2	DA	[353-354],[456-457]
9497	DPP-III inhibitor	dipeptidyl peptidase III inhibitor	2	IH	[111-112],[495-496]
9499	DPP-III inhibitor	dipeptidyl peptidase III inhibitor	5	LA	[178-179],[241-242],[251-252],[356-357],[431-432]
9500	DPP-III inhibitor	dipeptidyl peptidase III inhibitor	3	FA	[21-22],[404-405],[470-471]
9501	DPP-III inhibitor	dipeptidyl peptidase III inhibitor	2	FR	[82-83],[438-439]
9502	DPP-III inhibitor	dipeptidyl peptidase III inhibitor	2	FL	[13-14],[340-341]
9504	DPP-III inhibitor	dipeptidyl peptidase III inhibitor	2	PE	[100-101],[120-121]
9509	DPP-III inhibitor	dipeptidyl peptidase III inhibitor	3	VY	[103-104],[319-320],[368-369]
9510	DPP-III inhibitor	dipeptidyl peptidase III inhibitor	1	YI	[369-370]
9512	DPP-III inhibitor	dipeptidyl peptidase III inhibitor	1	GFL	[12-14]
8247	CaMPDE inhibitor	CaMPDE inhibitor	1	IR	[392-393]
8249	CaMPDE inhibitor	CaMPDE inhibitor	2	KF	[194-195],[414-415]
8250	CaMPDE inhibitor	CaMPDE inhibitor	1	EF	[65-66]
2842	Renin inhibitor	renin inhibitor	2	LR	[192-193],[338-339]
8246	renin inhibitor	renin inhibitor	1	IR	[392-393]
8248	Renin inhibitor	renin inhibitor	2	KF	[194-195],[414-415]
8251	Renin inhibitor	renin inhibitor	1	EF	[65-66]
9432	Renin inhibitor	renin inhibitor	5	SF	[8-9],[20-21],[244-245],[419-420],[449-450]

9469	Renin inhibitor	renin inhibitor	1	RALP	[439-442]
9471	Renin inhibitor	renin inhibitor	2	TF	[122-123],[301-302]

Table S19. Profile of potential biological activity of fragments of protein Pis v 2.0201.

ID	Name of peptide	Activity	Number	Sequence	Location
3460	Prolyl endopeptidase inhibitor	antiamnestic	2	PG	[117-118],[463-464]
3258	beta-lactokinin	ACE inhibitor	2	IR	[154-155],[383-384]
3361	ACE inhibitor from sardine	ACE inhibitor	1	LKL	[298-300]
3373	ACE inhibitor from beta-CN	ACE inhibitor	1	PQR	[277-279]
3381	ACE inhibitor	ACE inhibitor	1	LY	[341-342]
3383	ACE inhibitor	ACE inhibitor	1	IY	[170-171]
3384	ACE inhibitor	ACE inhibitor	3	VF	[81-82],[115-116],[378-379]
3393	ACE inhibitor	ACE inhibitor	1	FAP	[461-463]
3421	ACE inhibitor	ACE inhibitor	1	LVL	[178-180]
3463	ACE inhibitor	ACE inhibitor	1	HIR	[153-155]
3489	ACE inhibitor from sake lees	ACE inhibitor	2	RF	[38-39],[330-331]
3492	ACE inhibitor from sake	ACE inhibitor	3	VY	[103-104],[310-311],[359-360]
3502	ACE inhibitor (BSA fr. 221-222)	ACE inhibitor	1	FP	[116-117]
3532	ACE inhibitor	ACE inhibitor	1	GY	[2-3]
3537	ACE inhibitor	ACE inhibitor	1	PR	[313-314]
3541	ACE inhibitor	ACE inhibitor	1	LSP	[275-277]
3551	ACE inhibitor (from bovine beta-Lg)	ACE inhibitor	1	LF	[16-17]
7502	ACE inhibitor	ACE inhibitor	1	IVR	[265-267]
7509	ACE inhibitor from Alaskan pollack skin	ACE inhibitor	1	GLP	[431-433]
7513	ACE inhibitor from Alaskan pollack skin	ACE inhibitor	1	PL	[433-434]
7541	ACE inhibitor from wheat germ hydrolysate	ACE inhibitor	1	IVY	[358-360]
7543	ACE inhibitor	ACE inhibitor	1	AW	[448-449]
7558	ACE inhibitor from buckwheat	ACE inhibitor	3	VK	[252-253],[268-269],[398-399]
7559	ACE inhibitor from buckwheat	ACE inhibitor	1	PSY	[94-96]
7562	ACE inhibitor from soy hydrolysate	ACE inhibitor	1	IA	[160-161]
7583	ACE inhibitor	ACE inhibitor	1	AF	[235-236]
7584	ACE inhibitor	ACE inhibitor	3	AP	[99-100],[348-349],[462-463]
7585	ACE inhibitor	ACE inhibitor	4	LA	[183-184],[241-242],[416-417],[422-423]
7586	ACE inhibitor	ACE inhibitor	2	KR	[54-55],[399-400]

7587	ACE inhibitor	ACE inhibitor	2	VP	[93-94],[391-392]
7588	ACE inhibitor	ACE inhibitor	2	RA	[307-308],[400-401]
7591	ACE inhibitor	ACE inhibitor	2	GF	[12-13],[405-406]
7592	ACE inhibitor	ACE inhibitor	1	FR	[82-83]
7593	ACE inhibitor	ACE inhibitor	1	IF	[460-461]
7594	ACE inhibitor	ACE inhibitor	1	VG	[186-187]
7596	ACE inhibitor	ACE inhibitor	2	GI	[110-111],[263-264]
7598	ACE inhibitor	ACE inhibitor	1	GA	[113-114]
7599	ACE inhibitor	ACE inhibitor	4	GL	[90-91],[288-289],[415-416],[431-432]
7600	ACE inhibitor	ACE inhibitor	3	AG	[61-62],[164-165],[423-424]
7603	ACE inhibitor	ACE inhibitor	3	GR	[316-317],[366-367],[424-425]
7604	ACE inhibitor	ACE inhibitor	2	KG	[225-226],[338-339]
7606	ACE inhibitor	ACE inhibitor	1	DA	[447-448]
7607	ACE inhibitor	ACE inhibitor	3	GS	[108-109],[204-205],[464-465]
7608	ACE inhibitor	ACE inhibitor	3	GV	[62-63],[165-166],[339-340]
7609	ACE inhibitor	ACE inhibitor	1	MG	[1-2]
7610	ACE inhibitor	ACE inhibitor	4	GQ	[174-175],[214-215],[226-227],[386-387]
7614	ACE inhibitor	ACE inhibitor	1	HG	[112-113]
7615	ACE inhibitor	ACE inhibitor	2	GE	[145-146],[375-376]
7616	ACE inhibitor	ACE inhibitor	4	GG	[203-204],[212-213],[213-214],[315-316]
7617	ACE inhibitor	ACE inhibitor	2	QG	[107-108],[211-212]
7618	ACE inhibitor	ACE inhibitor	2	SG	[109-110],[144-145]
7619	ACE inhibitor	ACE inhibitor	2	LG	[11-12],[202-203]
7620	ACE inhibitor	ACE inhibitor	1	GD	[157-158]
7622	ACE inhibitor	ACE inhibitor	2	EG	[156-157],[385-386]
7623	ACE inhibitor	ACE inhibitor	1	EA	[60-61]
7624	ACE inhibitor	ACE inhibitor	5	NG	[173-174],[287-288],[365-366],[374-375],[414-415]
7625	ACE inhibitor	ACE inhibitor	2	PG	[117-118],[463-464]
7628	ACE inhibitor from k-CN (fr. 67-68)	ACE inhibitor	2	VR	[151-152],[266-267]
7635	ACE inhibitor from k-CN (fr. 51-53)	ACE inhibitor	1	VAV	[79-81]
7680	ACE inhibitor from pea vicilin	ACE inhibitor	1	QK	[149-150]
7681	ACE inhibitor from soy	ACE inhibitor	1	DG	[404-405]

7683	ACE inhibitor from garlic	ACE inhibitor	1	NF	[394-395]
7684	ACE inhibitor from garlic	ACE inhibitor	1	SY	[95-96]
7685	ACE inhibitor from garlic	ACE inhibitor	5	SF	[8-9],[20-21],[244-245],[410-411],[440-441]
7692	ACE inhibitor	ACE inhibitor	1	KF	[199-200]
7693	ACE inhibitor from wakame	ACE inhibitor	3	KL	[177-178],[254-255],[299-300]
7741	ACE inhibitor	ACE inhibitor	1	RR	[55-56]
7752	ACE inhibitor from shark meat hydrolysate	ACE inhibitor	1	EY	[283-284]
7807	ACE inhibitor from caprine b-Lg	ACE inhibitor	1	LLF	[15-17]
7826	ACE inhibitor	ACE inhibitor	3	EI	[209-210],[239-240],[382-383]
7827	ACE inhibitor	ACE inhibitor	2	IE	[24-25],[57-58]
7829	ACE inhibitor	ACE inhibitor	1	VE	[336-337]
7830	ACE inhibitor	ACE inhibitor	1	TE	[64-65]
7831	ACE inhibitor	ACE inhibitor	4	LQ	[74-75],[255-256],[272-273],[332-333]
7832	ACE inhibitor	ACE inhibitor	3	LN	[48-49],[300-301],[324-325]
7834	ACE inhibitor	ACE inhibitor	2	TQ	[41-42],[249-250]
7835	ACE inhibitor	ACE inhibitor	2	AH	[167-168],[355-356]
7837	ACE inhibitor	ACE inhibitor	3	PQ	[206-207],[277-278],[392-393]
7838	ACE inhibitor	ACE inhibitor	1	EW	[407-408]
7840	ACE inhibitor	ACE inhibitor	3	EK	[259-260],[281-282],[337-338]
7841	ACE inhibitor	ACE inhibitor	3	KE	[269-270],[282-283],[452-453]
7843	ACE inhibitor	ACE inhibitor	1	PH	[349-350]
8182	ACE Inhibitor	ACE inhibitor	1	ALEP	[50-53]
8185	ACE inhibitor	ACE inhibitor	2	TF	[122-123],[292-293]
8193	ACE inhibitor	ACE inhibitor	1	AI	[345-346]
8402	ACE inhibitor	ACE inhibitor	1	LVY	[102-104]
8951	ACE inhibitor	ACE inhibitor	3	AV	[80-81],[114-115],[396-397]
9029	ACE inhibitor	ACE inhibitor	1	ALP	[161-163]
9031	ACE inhibitor	ACE inhibitor	1	LEE	[289-291]
9050	ACE inhibitor	ACE inhibitor	1	VLY	[340-342]
9076	ACE inhibitor	ACE inhibitor	2	FQ	[39-40],[123-124]
9077	ACE inhibitor	ACE inhibitor	1	YV	[104-105]
9079	ACE inhibitor	ACE inhibitor	3	IL	[232-233],[240-241],[328-329]

9173	ACE inhibitor	ACE inhibitor	5	RG	[89-90],[262-263],[314-315],[363-364],[430-431]
9185	ACE inhibitor	ACE inhibitor	3	YN	[96-97],[171-172],[311-312]
9196	ACE inhibitor	ACE inhibitor	1	AVV	[396-398]
9213	ACE inhibitor	ACE inhibitor	2	LR	[197-198],[329-330]
9241	ACE inhibitor	ACE inhibitor	1	MAP	[347-349]
9325	ACE inhibitor	ACE inhibitor	1	GVLY	[339-342]
9708	ACE inhibitor	ACE inhibitor	1	LPILR	[326-330]
9730	ACE inhibitor	ACE inhibitor	1	NPR	[312-314]
3285	Antithrombotic peptide	antithrombotic	2	PG	[117-118],[463-464]
3354	Antithrombotic peptide	antithrombotic	2	DEE	[237-239],[380-382]
3356	Stimulating vasoactive substance release	stimulating	1	LLL	[14-16]
8320	Glucose uptake stimulating peptide	stimulating	4	VL	[179-180],[201-202],[274-275],[340-341]
8321	Glucose uptake stimulating peptide	stimulating	6	LV	[92-93],[102-103],[178-179],[180-181],[251-252],[388-389]
8322	Glucose uptake stimulating peptide	stimulating	3	IV	[265-266],[358-359],[370-371]
8323	Glucose uptake stimulating peptide	stimulating	3	IL	[232-233],[240-241],[328-329]
8325	Glucose uptake stimulating peptide	stimulating	2	II	[159-160],[264-265]
8326	Glucose uptake stimulating peptide	stimulating	4	LL	[6-7],[14-15],[15-16],[91-92]
8329	Stimulating vasoactive substance release	stimulating	6	EE	[71-72],[125-126],[238-239],[258-259],[290-291],[381-382]
8330	Stimulating vasoactive substance release	stimulating	5	SE	[59-60],[138-139],[189-190],[372-373],[456-457]
3061	Immunostimulating peptide	immunostimulating	1	GFL	[12-14]
2890	neuropeptide	neuropeptide	4	GQ	[174-175],[214-215],[226-227],[386-387]
8310	Anxiolytic peptide	neuropeptide	1	YL	[196-197]
2737	Peptide regulating phosphoinositol metabolism	regulating	1	GFL	[12-14]
2754	peptide regulating the stomach mucosal membrane activity	regulating	2	PG	[117-118],[463-464]
8318	Dvl protein binding	anticancer	1	VVV	[389-391]
7872	peptide from soybean protein isolates: beta-conglycinin and glycinin	antioxidative	1	LY	[341-342]
7873	peptide from soybean protein isolates: beta-conglycinin and glycinin	antioxidative	1	IY	[170-171]
7886	peptide derived from egg white albumin	antioxidative	2	AH	[167-168],[355-356]
7888	antioxidative peptide	antioxidative	1	EL	[101-102]
8037	synthetic peptide	antioxidative	1	PHW	[349-351]
8059	synthetic peptide	antioxidative	1	RHI	[152-154]

8067	synthetic peptide	antioxidative	1	RHT	[83-85]
8139	synthetic peptide	antioxidative	1	PEL	[100-102]
8215	Antioxidative peptide	antioxidative	2	IR	[154-155],[383-384]
8217	Antioxidative peptide	antioxidative	2	LK	[298-299],[451-452]
8224	antioxidative peptide	antioxidative	3	VY	[103-104],[310-311],[359-360]
8460	Antioxidant peptide from marine bivalve (Mactra veneriformis)	antioxidative	1	AW	[448-449]
9342	Antioxidative peptide	antioxidative	1	FC	[293-294]
9361	Antioxidative peptide	antioxidative	1	VYV	[103-105]
9716	Antioxidative peptide	antioxidative	1	LPILR	[326-330]
3751		bacterial permease ligand	1	KK	[253-254]
8661	Hypotensive peptide	hypotensive	1	LPILR	[326-330]
4005		activating ubiquitin-mediated proteolysis	2	RA	[307-308],[400-401]
4006	Ubiqitin-mediated proteolysis activating peptide	activating ubiquitin-mediated proteolysis	4	LA	[183-184],[241-242],[416-417],[422-423]
9580	Hypolipidemic peptide	hypolipidemic	1	EF	[65-66]
9650	Alpha-glucosidase inhibitor	alpha-glucosidase inhibitor	1	EA	[60-61]
9693	Alpha-glucosidase inhibitor	alpha-glucosidase inhibitor	1	VE	[336-337]
9694	Alpha-glucosidase inhibitor	alpha-glucosidase inhibitor	2	PE	[100-101],[120-121]
9695	Alpha-glucosidase inhibitor	alpha-glucosidase inhibitor	2	AD	[184-185],[308-309]
3172	dipeptidyl peptidase IV inhibitor (DPP IV inhibitor)	dipeptidyl peptidase IV inhibitor	3	VA	[79-80],[166-167],[181-182]
3173	dipeptidyl peptidase IV inhibitor (DPP IV inhibitor)	dipeptidyl peptidase IV inhibitor	1	MA	[347-348]
3175	dipeptidyl peptidase IV inhibitor (DPP IV inhibitor)	dipeptidyl peptidase IV inhibitor	4	LA	[183-184],[241-242],[416-417],[422-423]
3176	dipeptidyl peptidase IV inhibitor (DPP IV inhibitor)	dipeptidyl peptidase IV inhibitor	3	FA	[21-22],[395-396],[461-462]
3177	dipeptidyl peptidase IV inhibitor (DPP IV inhibitor)	dipeptidyl peptidase IV inhibitor	3	AP	[99-100],[348-349],[462-463]
3179	dipeptidyl peptidase IV inhibitor (DPP IV inhibitor)	dipeptidyl peptidase IV inhibitor	1	PA	[163-164]
3180	dipeptidyl peptidase IV inhibitor (DPP IV inhibitor)	dipeptidyl peptidase IV inhibitor	3	LP	[162-163],[326-327],[432-433]
3181	dipeptidyl peptidase IV inhibitor (DPP IV inhibitor)	dipeptidyl peptidase IV inhibitor	2	VP	[93-94],[391-392]
3182	dipeptidyl peptidase IV inhibitor (DPP IV inhibitor)	dipeptidyl peptidase IV inhibitor	4	LL	[6-7],[14-15],[15-16],[91-92]
3183	dipeptidyl peptidase IV inhibitor (DPP IV inhibitor)	dipeptidyl peptidase IV inhibitor	5	VV	[27-28],[105-106],[389-390],[390-391],[397-398]
8500	dipeptidyl peptidase IV inhibitor (DPP IV inhibitor)	dipeptidyl peptidase IV inhibitor	1	APG	[462-464]
8505	Dipeptidyl peptidase IV inhibitor (DPP IV inhibitor)	dipeptidyl peptidase IV inhibitor	2	SP	[205-206],[276-277]
8506	dipeptidyl peptidase IV inhibitor (DPP IV inhibitor)	dipeptidyl peptidase IV inhibitor	1	FP	[116-117]

8524	dipeptidyl peptidase IV inhibitor (DPP IV inhibitor)	dipeptidyl peptidase IV inhibitor	1	GA	[113-114]
8525	dipeptidyl peptidase IV inhibitor (DPP IV inhibitor)	dipeptidyl peptidase IV inhibitor	1	IA	[160-161]
8526	dipeptidyl peptidase IV inhibitor (DPP IV inhibitor)	dipeptidyl peptidase IV inhibitor	2	RA	[307-308],[400-401]
8529	dipeptidyl peptidase IV inhibitor (DPP IV inhibitor)	dipeptidyl peptidase IV inhibitor	1	EP	[52-53]
8530	dipeptidyl peptidase IV inhibitor (DPP IV inhibitor)	dipeptidyl peptidase IV inhibitor	1	NP	[312-313]
8555	dipeptidyl peptidase IV inhibitor (DPP IV inhibitor)	dipeptidyl peptidase IV inhibitor	2	FL	[13-14],[331-332]
8556	dipeptidyl peptidase IV inhibitor (DPP IV inhibitor)	dipeptidyl peptidase IV inhibitor	1	WV	[408-409]
8558	dipeptidyl peptidase IV inhibitor (DPP IV inhibitor)	dipeptidyl peptidase IV inhibitor	3	EK	[259-260],[281-282],[337-338]
8559	dipeptidyl peptidase IV inhibitor (DPP IV inhibitor)	dipeptidyl peptidase IV inhibitor	4	AL	[50-51],[161-162],[182-183],[323-324]
8560	dipeptidyl peptidase IV inhibitor (DPP IV inhibitor)	dipeptidyl peptidase IV inhibitor	2	SL	[5-6],[10-11]
8561	dipeptidyl peptidase IV inhibitor (DPP IV inhibitor)	dipeptidyl peptidase IV inhibitor	4	GL	[90-91],[288-289],[415-416],[431-432]
8594	dipeptidyl peptidase IV inhibitor (DPP IV inhibitor)	dipeptidyl peptidase IV inhibitor	2	VR	[151-152],[266-267]
8616	dipeptidyl peptidase IV inhibitor (DPP IV inhibitor)	dipeptidyl peptidase IV inhibitor	1	LPL	[432-434]
8638	dipeptidyl peptidase IV inhibitor (DPP IV inhibitor)	dipeptidyl peptidase IV inhibitor	1	PL	[433-434]
8679	dipeptidyl peptidase IV inhibitor (DPP IV inhibitor)	dipeptidyl peptidase IV inhibitor	1	WI	[169-170]
8680	dipeptidyl peptidase IV inhibitor (DPP IV inhibitor)	dipeptidyl peptidase IV inhibitor	2	WN	[351-352],[449-450]
8687	dipeptidyl peptidase IV inhibitor (DPP IV inhibitor)	dipeptidyl peptidase IV inhibitor	1	WS	[217-218]
8695	dipeptidyl peptidase IV inhibitor (DPP IV inhibitor)	dipeptidyl peptidase IV inhibitor	1	AW	[448-449]
8757	dipeptidyl peptidase IV inhibitor (DPP IV inhibitor)	dipeptidyl peptidase IV inhibitor	2	AD	[184-185],[308-309]
8759	dipeptidyl peptidase IV inhibitor (DPP IV inhibitor)	dipeptidyl peptidase IV inhibitor	1	AF	[235-236]
8760	dipeptidyl peptidase IV inhibitor (DPP IV inhibitor)	dipeptidyl peptidase IV inhibitor	3	AG	[61-62],[164-165],[423-424]
8761	dipeptidyl peptidase IV inhibitor (DPP IV inhibitor)	dipeptidyl peptidase IV inhibitor	2	AH	[167-168],[355-356]
8762	dipeptidyl peptidase IV inhibitor (DPP IV inhibitor)	dipeptidyl peptidase IV inhibitor	1	AS	[401-402]
8764	dipeptidyl peptidase IV inhibitor (DPP IV inhibitor)	dipeptidyl peptidase IV inhibitor	3	AV	[80-81],[114-115],[396-397]
8766	dipeptidyl peptidase IV inhibitor (DPP IV inhibitor)	dipeptidyl peptidase IV inhibitor	1	DN	[286-287]
8767	dipeptidyl peptidase IV inhibitor (DPP IV inhibitor)	dipeptidyl peptidase IV inhibitor	1	DP	[304-305]
8768	dipeptidyl peptidase IV inhibitor (DPP IV inhibitor)	dipeptidyl peptidase IV inhibitor	2	DQ	[68-69],[194-195]
8770	dipeptidyl peptidase IV inhibitor (DPP IV inhibitor)	dipeptidyl peptidase IV inhibitor	2	EG	[156-157],[385-386]
8772	dipeptidyl peptidase IV inhibitor (DPP IV inhibitor)	dipeptidyl peptidase IV inhibitor	3	EI	[209-210],[239-240],[382-383]
8773	dipeptidyl peptidase IV inhibitor (DPP IV inhibitor)	dipeptidyl peptidase IV inhibitor	4	ES	[58-59],[126-127],[376-377],[453-454]
8774	dipeptidyl peptidase IV inhibitor (DPP IV inhibitor)	dipeptidyl peptidase IV inhibitor	2	ET	[121-122],[291-292]
8776	dipeptidyl peptidase IV inhibitor (DPP IV inhibitor)	dipeptidyl peptidase IV inhibitor	1	EW	[407-408]

8777	dipeptidyl peptidase IV inhibitor (DPP IV inhibitor)	dipeptidyl peptidase IV inhibitor	1	EY	[283-284]
8778	dipeptidyl peptidase IV inhibitor (DPP IV inhibitor)	dipeptidyl peptidase IV inhibitor	1	FN	[245-246]
8779	dipeptidyl peptidase IV inhibitor (DPP IV inhibitor)	dipeptidyl peptidase IV inhibitor	2	FQ	[39-40],[123-124]
8780	dipeptidyl peptidase IV inhibitor (DPP IV inhibitor)	dipeptidyl peptidase IV inhibitor	1	FR	[82-83]
8781	dipeptidyl peptidase IV inhibitor (DPP IV inhibitor)	dipeptidyl peptidase IV inhibitor	2	GE	[145-146],[375-376]
8782	dipeptidyl peptidase IV inhibitor (DPP IV inhibitor)	dipeptidyl peptidase IV inhibitor	2	GF	[12-13],[405-406]
8783	dipeptidyl peptidase IV inhibitor (DPP IV inhibitor)	dipeptidyl peptidase IV inhibitor	4	GG	[203-204],[212-213],[213-214],[315-316]
8785	dipeptidyl peptidase IV inhibitor (DPP IV inhibitor)	dipeptidyl peptidase IV inhibitor	2	GI	[110-111],[263-264]
8786	dipeptidyl peptidase IV inhibitor (DPP IV inhibitor)	dipeptidyl peptidase IV inhibitor	3	GV	[62-63],[165-166],[339-340]
8788	dipeptidyl peptidase IV inhibitor (DPP IV inhibitor)	dipeptidyl peptidase IV inhibitor	1	GY	[2-3]
8793	dipeptidyl peptidase IV inhibitor (DPP IV inhibitor)	dipeptidyl peptidase IV inhibitor	1	HI	[153-154]
8795	dipeptidyl peptidase IV inhibitor (DPP IV inhibitor)	dipeptidyl peptidase IV inhibitor	2	HS	[135-136],[356-357]
8796	dipeptidyl peptidase IV inhibitor (DPP IV inhibitor)	dipeptidyl peptidase IV inhibitor	1	HT	[84-85]
8798	dipeptidyl peptidase IV inhibitor (DPP IV inhibitor)	dipeptidyl peptidase IV inhibitor	2	HW	[168-169],[350-351]
8800	dipeptidyl peptidase IV inhibitor (DPP IV inhibitor)	dipeptidyl peptidase IV inhibitor	1	IH	[111-112]
8801	dipeptidyl peptidase IV inhibitor (DPP IV inhibitor)	dipeptidyl peptidase IV inhibitor	2	II	[159-160],[264-265]
8802	dipeptidyl peptidase IV inhibitor (DPP IV inhibitor)	dipeptidyl peptidase IV inhibitor	3	IL	[232-233],[240-241],[328-329]
8803	dipeptidyl peptidase IV inhibitor (DPP IV inhibitor)	dipeptidyl peptidase IV inhibitor	1	IM	[346-347]
8804	dipeptidyl peptidase IV inhibitor (DPP IV inhibitor)	dipeptidyl peptidase IV inhibitor	2	IN	[302-303],[321-322]
8805	dipeptidyl peptidase IV inhibitor (DPP IV inhibitor)	dipeptidyl peptidase IV inhibitor	4	IQ	[45-46],[86-87],[210-211],[437-438]
8806	dipeptidyl peptidase IV inhibitor (DPP IV inhibitor)	dipeptidyl peptidase IV inhibitor	2	IR	[154-155],[383-384]
8808	dipeptidyl peptidase IV inhibitor (DPP IV inhibitor)	dipeptidyl peptidase IV inhibitor	3	KE	[269-270],[282-283],[452-453]
8809	dipeptidyl peptidase IV inhibitor (DPP IV inhibitor)	dipeptidyl peptidase IV inhibitor	1	KF	[199-200]
8810	dipeptidyl peptidase IV inhibitor (DPP IV inhibitor)	dipeptidyl peptidase IV inhibitor	2	KG	[225-226],[338-339]
8812	dipeptidyl peptidase IV inhibitor (DPP IV inhibitor)	dipeptidyl peptidase IV inhibitor	1	KI	[418-419]
8813	dipeptidyl peptidase IV inhibitor (DPP IV inhibitor)	dipeptidyl peptidase IV inhibitor	1	KK	[253-254]
8814	dipeptidyl peptidase IV inhibitor (DPP IV inhibitor)	dipeptidyl peptidase IV inhibitor	2	KR	[54-55],[399-400]
8816	dipeptidyl peptidase IV inhibitor (DPP IV inhibitor)	dipeptidyl peptidase IV inhibitor	1	KT	[412-413]
8817	dipeptidyl peptidase IV inhibitor (DPP IV inhibitor)	dipeptidyl peptidase IV inhibitor	1	KV	[150-151]
8823	dipeptidyl peptidase IV inhibitor (DPP IV inhibitor)	dipeptidyl peptidase IV inhibitor	3	LN	[48-49],[300-301],[324-325]
8825	dipeptidyl peptidase IV inhibitor (DPP IV inhibitor)	dipeptidyl peptidase IV inhibitor	6	LV	[92-93],[102-103],[178-179],[180-181],[251-252],[388-389]
8828	dipeptidyl peptidase IV inhibitor (DPP IV inhibitor)	dipeptidyl peptidase IV inhibitor	1	MG	[1-2]

8834	dipeptidyl peptidase IV inhibitor (DPP IV inhibitor)	dipeptidyl peptidase IV inhibitor	1	MN	[353-354]
8835	dipeptidyl peptidase IV inhibitor (DPP IV inhibitor)	dipeptidyl peptidase IV inhibitor	1	MQ	[368-369]
8836	dipeptidyl peptidase IV inhibitor (DPP IV inhibitor)	dipeptidyl peptidase IV inhibitor	1	MR	[429-430]
8839	dipeptidyl peptidase IV inhibitor (DPP IV inhibitor)	dipeptidyl peptidase IV inhibitor	5	NA	[49-50],[98-99],[322-323],[344-345],[354-355]
8840	dipeptidyl peptidase IV inhibitor (DPP IV inhibitor)	dipeptidyl peptidase IV inhibitor	1	ND	[303-304]
8841	dipeptidyl peptidase IV inhibitor (DPP IV inhibitor)	dipeptidyl peptidase IV inhibitor	1	NE	[70-71]
8842	dipeptidyl peptidase IV inhibitor (DPP IV inhibitor)	dipeptidyl peptidase IV inhibitor	1	NF	[394-395]
8843	dipeptidyl peptidase IV inhibitor (DPP IV inhibitor)	dipeptidyl peptidase IV inhibitor	5	NG	[173-174],[287-288],[365-366],[374-375],[414-415]
8845	dipeptidyl peptidase IV inhibitor (DPP IV inhibitor)	dipeptidyl peptidase IV inhibitor	3	NL	[47-48],[325-326],[450-451]
8846	dipeptidyl peptidase IV inhibitor (DPP IV inhibitor)	dipeptidyl peptidase IV inhibitor	1	NM	[352-353]
8847	dipeptidyl peptidase IV inhibitor (DPP IV inhibitor)	dipeptidyl peptidase IV inhibitor	3	NN	[97-98],[172-173],[230-231]
8848	dipeptidyl peptidase IV inhibitor (DPP IV inhibitor)	dipeptidyl peptidase IV inhibitor	1	NQ	[191-192]
8851	dipeptidyl peptidase IV inhibitor (DPP IV inhibitor)	dipeptidyl peptidase IV inhibitor	1	NV	[78-79]
8855	dipeptidyl peptidase IV inhibitor (DPP IV inhibitor)	dipeptidyl peptidase IV inhibitor	2	PG	[117-118],[463-464]
8856	dipeptidyl peptidase IV inhibitor (DPP IV inhibitor)	dipeptidyl peptidase IV inhibitor	1	PH	[349-350]
8857	dipeptidyl peptidase IV inhibitor (DPP IV inhibitor)	dipeptidyl peptidase IV inhibitor	1	PI	[327-328]
8858	dipeptidyl peptidase IV inhibitor (DPP IV inhibitor)	dipeptidyl peptidase IV inhibitor	1	PK	[53-54]
8861	dipeptidyl peptidase IV inhibitor (DPP IV inhibitor)	dipeptidyl peptidase IV inhibitor	3	PQ	[206-207],[277-278],[392-393]
8862	dipeptidyl peptidase IV inhibitor (DPP IV inhibitor)	dipeptidyl peptidase IV inhibitor	2	PS	[94-95],[305-306]
8869	dipeptidyl peptidase IV inhibitor (DPP IV inhibitor)	dipeptidyl peptidase IV inhibitor	3	QE	[124-125],[208-209],[280-281]
8871	dipeptidyl peptidase IV inhibitor (DPP IV inhibitor)	dipeptidyl peptidase IV inhibitor	2	QG	[107-108],[211-212]
8872	dipeptidyl peptidase IV inhibitor (DPP IV inhibitor)	dipeptidyl peptidase IV inhibitor	2	QH	[134-135],[147-148]
8873	dipeptidyl peptidase IV inhibitor (DPP IV inhibitor)	dipeptidyl peptidase IV inhibitor	3	QI	[23-24],[44-45],[369-370]
8874	dipeptidyl peptidase IV inhibitor (DPP IV inhibitor)	dipeptidyl peptidase IV inhibitor	6	QL	[73-74],[192-193],[250-251],[333-334],[387-388],[421-422]
8875	dipeptidyl peptidase IV inhibitor (DPP IV inhibitor)	dipeptidyl peptidase IV inhibitor	5	QN	[46-47],[69-70],[343-344],[393-394],[438-439]
8876	dipeptidyl peptidase IV inhibitor (DPP IV inhibitor)	dipeptidyl peptidase IV inhibitor	7	QQ	[31-32],[34-35],[35-36],[36-37],[142-143],[207-208],[227-228]
8877	dipeptidyl peptidase IV inhibitor (DPP IV inhibitor)	dipeptidyl peptidase IV inhibitor	9	QS	[87-88],[128-129],[130-131],[143-144],[175-176],[215-216],[219-220],[228-229],[243-244]
8878	dipeptidyl peptidase IV inhibitor (DPP IV inhibitor)	dipeptidyl peptidase IV inhibitor	1	QT	[40-41]
8879	dipeptidyl peptidase IV inhibitor (DPP IV inhibitor)	dipeptidyl peptidase IV inhibitor	2	QV	[26-27],[273-274]
8881	dipeptidyl peptidase IV inhibitor (DPP IV inhibitor)	dipeptidyl peptidase IV inhibitor	1	QY	[195-196]
8882	dipeptidyl peptidase IV inhibitor (DPP IV inhibitor)	dipeptidyl peptidase IV inhibitor	5	RG	[89-90],[262-263],[314-315],[363-364],[430-431]

8883	dipeptidyl peptidase IV inhibitor (DPP IV inhibitor)	dipeptidyl peptidase IV inhibitor	2	RH	[83-84],[152-153]
8884	dipeptidyl peptidase IV inhibitor (DPP IV inhibitor)	dipeptidyl peptidase IV inhibitor	2	RI	[56-57],[425-426]
8885	dipeptidyl peptidase IV inhibitor (DPP IV inhibitor)	dipeptidyl peptidase IV inhibitor	2	RK	[198-199],[224-225]
8887	dipeptidyl peptidase IV inhibitor (DPP IV inhibitor)	dipeptidyl peptidase IV inhibitor	1	RM	[367-368]
8888	dipeptidyl peptidase IV inhibitor (DPP IV inhibitor)	dipeptidyl peptidase IV inhibitor	1	RN	[471-472]
8889	dipeptidyl peptidase IV inhibitor (DPP IV inhibitor)	dipeptidyl peptidase IV inhibitor	1	RR	[55-56]
8891	dipeptidyl peptidase IV inhibitor (DPP IV inhibitor)	dipeptidyl peptidase IV inhibitor	5	SF	[8-9],[20-21],[244-245],[410-411],[440-441]
8893	dipeptidyl peptidase IV inhibitor (DPP IV inhibitor)	dipeptidyl peptidase IV inhibitor	2	SI	[320-321],[357-358]
8894	dipeptidyl peptidase IV inhibitor (DPP IV inhibitor)	dipeptidyl peptidase IV inhibitor	1	SK	[176-177]
8895	dipeptidyl peptidase IV inhibitor (DPP IV inhibitor)	dipeptidyl peptidase IV inhibitor	3	SV	[335-336],[377-378],[427-428]
8896	dipeptidyl peptidase IV inhibitor (DPP IV inhibitor)	dipeptidyl peptidase IV inhibitor	1	SW	[216-217]
8897	dipeptidyl peptidase IV inhibitor (DPP IV inhibitor)	dipeptidyl peptidase IV inhibitor	1	SY	[95-96]
8899	dipeptidyl peptidase IV inhibitor (DPP IV inhibitor)	dipeptidyl peptidase IV inhibitor	1	TE	[64-65]
8900	dipeptidyl peptidase IV inhibitor (DPP IV inhibitor)	dipeptidyl peptidase IV inhibitor	2	TF	[122-123],[292-293]
8903	dipeptidyl peptidase IV inhibitor (DPP IV inhibitor)	dipeptidyl peptidase IV inhibitor	2	TI	[85-86],[459-460]
8905	dipeptidyl peptidase IV inhibitor (DPP IV inhibitor)	dipeptidyl peptidase IV inhibitor	1	TL	[297-298]
8906	dipeptidyl peptidase IV inhibitor (DPP IV inhibitor)	dipeptidyl peptidase IV inhibitor	1	TM	[295-296]
8907	dipeptidyl peptidase IV inhibitor (DPP IV inhibitor)	dipeptidyl peptidase IV inhibitor	1	TN	[413-414]
8908	dipeptidyl peptidase IV inhibitor (DPP IV inhibitor)	dipeptidyl peptidase IV inhibitor	2	TQ	[41-42],[249-250]
8909	dipeptidyl peptidase IV inhibitor (DPP IV inhibitor)	dipeptidyl peptidase IV inhibitor	1	TR	[362-363]
8910	dipeptidyl peptidase IV inhibitor (DPP IV inhibitor)	dipeptidyl peptidase IV inhibitor	1	TS	[319-320]
8916	dipeptidyl peptidase IV inhibitor (DPP IV inhibitor)	dipeptidyl peptidase IV inhibitor	1	VE	[336-337]
8917	dipeptidyl peptidase IV inhibitor (DPP IV inhibitor)	dipeptidyl peptidase IV inhibitor	3	VF	[81-82],[115-116],[378-379]
8918	dipeptidyl peptidase IV inhibitor (DPP IV inhibitor)	dipeptidyl peptidase IV inhibitor	1	VG	[186-187]
8920	dipeptidyl peptidase IV inhibitor (DPP IV inhibitor)	dipeptidyl peptidase IV inhibitor	1	VI	[436-437]
8921	dipeptidyl peptidase IV inhibitor (DPP IV inhibitor)	dipeptidyl peptidase IV inhibitor	3	VK	[252-253],[268-269],[398-399]
8922	dipeptidyl peptidase IV inhibitor (DPP IV inhibitor)	dipeptidyl peptidase IV inhibitor	4	VL	[179-180],[201-202],[274-275],[340-341]
8923	dipeptidyl peptidase IV inhibitor (DPP IV inhibitor)	dipeptidyl peptidase IV inhibitor	1	VM	[428-429]
8924	dipeptidyl peptidase IV inhibitor (DPP IV inhibitor)	dipeptidyl peptidase IV inhibitor	1	VN	[28-29]
8925	dipeptidyl peptidase IV inhibitor (DPP IV inhibitor)	dipeptidyl peptidase IV inhibitor	1	VQ	[106-107]
8926	dipeptidyl peptidase IV inhibitor (DPP IV inhibitor)	dipeptidyl peptidase IV inhibitor	2	VS	[371-372],[409-410]
8927	dipeptidyl peptidase IV inhibitor (DPP IV inhibitor)	dipeptidyl peptidase IV inhibitor	2	VT	[63-64],[318-319]

8929	dipeptidyl peptidase IV inhibitor (DPP IV inhibitor)	dipeptidyl peptidase IV inhibitor	3	VY	[103-104],[310-311],[359-360]
8930	dipeptidyl peptidase IV inhibitor (DPP IV inhibitor)	dipeptidyl peptidase IV inhibitor	1	WD	[67-68]
8938	dipeptidyl peptidase IV inhibitor (DPP IV inhibitor)	dipeptidyl peptidase IV inhibitor	1	YI	[360-361]
8940	dipeptidyl peptidase IV inhibitor (DPP IV inhibitor)	dipeptidyl peptidase IV inhibitor	1	YL	[196-197]
8942	dipeptidyl peptidase IV inhibitor (DPP IV inhibitor)	dipeptidyl peptidase IV inhibitor	3	YN	[96-97],[171-172],[311-312]
8943	dipeptidyl peptidase IV inhibitor (DPP IV inhibitor)	dipeptidyl peptidase IV inhibitor	1	YQ	[342-343]
8945	dipeptidyl peptidase IV inhibitor (DPP IV inhibitor)	dipeptidyl peptidase IV inhibitor	2	YS	[3-4],[284-285]
8946	dipeptidyl peptidase IV inhibitor (DPP IV inhibitor)	dipeptidyl peptidase IV inhibitor	1	YV	[104-105]
9478	DPP-III inhibitor	dipeptidyl peptidase III inhibitor	2	LR	[197-198],[329-330]
9479	DPP-III inhibitor	dipeptidyl peptidase III inhibitor	1	MR	[429-430]
9482	DPP-III inhibitor	dipeptidyl peptidase III inhibitor	1	YL	[196-197]
9485	DPP-III inhibitor	dipeptidyl peptidase III inhibitor	1	RR	[55-56]
9486	DPP-III inhibitor	dipeptidyl peptidase III inhibitor	2	TF	[122-123],[292-293]
9487	DPP-III inhibitor	dipeptidyl peptidase III inhibitor	2	GE	[145-146],[375-376]
9488	DPP-III inhibitor	dipeptidyl peptidase III inhibitor	2	GF	[12-13],[405-406]
9489	DPP-III inhibitor	dipeptidyl peptidase III inhibitor	1	PR	[313-314]
9490	DPP-III inhibitor	dipeptidyl peptidase III inhibitor	2	RF	[38-39],[330-331]
9491	DPP-III inhibitor	dipeptidyl peptidase III inhibitor	2	RV	[267-268],[317-318]
9492	DPP-III inhibitor	dipeptidyl peptidase III inhibitor	1	DA	[447-448]
9497	DPP-III inhibitor	dipeptidyl peptidase III inhibitor	1	IH	[111-112]
9499	DPP-III inhibitor	dipeptidyl peptidase III inhibitor	4	LA	[183-184],[241-242],[416-417],[422-423]
9500	DPP-III inhibitor	dipeptidyl peptidase III inhibitor	3	FA	[21-22],[395-396],[461-462]
9501	DPP-III inhibitor	dipeptidyl peptidase III inhibitor	1	FR	[82-83]
9502	DPP-III inhibitor	dipeptidyl peptidase III inhibitor	2	FL	[13-14],[331-332]
9504	DPP-III inhibitor	dipeptidyl peptidase III inhibitor	2	PE	[100-101],[120-121]
9509	DPP-III inhibitor	dipeptidyl peptidase III inhibitor	3	VY	[103-104],[310-311],[359-360]
9510	DPP-III inhibitor	dipeptidyl peptidase III inhibitor	1	YI	[360-361]
9512	DPP-III inhibitor	dipeptidyl peptidase III inhibitor	1	GFL	[12-14]
8247	CaMPDE inhibitor	CaMPDE inhibitor	2	IR	[154-155],[383-384]
8249	CaMPDE inhibitor	CaMPDE inhibitor	1	KF	[199-200]
8250	CaMPDE inhibitor	CaMPDE inhibitor	1	EF	[65-66]
2842	Renin inhibitor	renin inhibitor	2	LR	[197-198],[329-330]

8246	renin inhibitor	renin inhibitor	2	IR	[154-155],[383-384]
8248	Renin inhibitor	renin inhibitor	1	KF	[199-200]
8251	Renin inhibitor	renin inhibitor	1	EF	[65-66]
9432	Renin inhibitor	renin inhibitor	5	SF	[8-9],[20-21],[244-245],[410-411],[440-441]
9470	Renin inhibitor	renin inhibitor	1	LY	[341-342]
9471	Renin inhibitor	renin inhibitor	2	TF	[122-123],[292-293]

Table S20. Profile of potential biological activity of fragments of protein Pis v 3.0101.

ID	Name of peptide	Activity	Number	Sequence	Location
3458	Prolyl endopeptidase inhibitor	antiamnestic	1	GPGG	[257-260]
3460	Prolyl endopeptidase inhibitor	antiamnestic	3	PG	[66-67],[249-250],[258-259]
3461	Prolyl endopeptidase inhibitor	antiamnestic	5	GP	[257-258],[315-316],[375-376],[414-415],[506-507]
2653	ACE inhibitor	ACE inhibitor	1	VLP	[149-151]
3257	beta-lactokinin	ACE inhibitor	3	RL	[70-71],[167-168],[394-395]
3258	beta-lactokinin	ACE inhibitor	6	IR	[202-203],[218-219],[220-221],[306-307],[424-425],[461-462]
3377	ACE inhibitor (from bovine as1-CN)	ACE inhibitor	1	FGK	[495-497]
3380	ACE inhibitor	ACE inhibitor	1	RY	[462-463]
3381	ACE inhibitor	ACE inhibitor	1	LY	[237-238]
3384	ACE inhibitor	ACE inhibitor	4	VF	[130-131],[254-255],[429-430],[494-495]
3386	ACE inhibitor	ACE inhibitor	1	KW	[508-509]
3489	ACE inhibitor from sake lees	ACE inhibitor	1	RF	[91-92]
3494	ACE inhibitor from sake	ACE inhibitor	1	HY	[251-252]
3537	ACE inhibitor	ACE inhibitor	1	PR	[282-283]
3542	ACE inhibitor	ACE inhibitor	1	LQP	[243-245]
3547	ACE inhibitor	ACE inhibitor	2	IRA	[220-222],[306-308]
3551	ACE inhibitor (from bovine beta-Lg)	ACE inhibitor	3	LF	[289-290],[331-332],[345-346]
3553	ACE inhibitor	ACE inhibitor	1	YG	[342-343]
3556	ACE inhibitor	ACE inhibitor	3	FY	[226-227],[266-267],[377-378]
3666	ACE inhibitor	ACE inhibitor	1	YP	[353-354]
7511	ACE inhibitor from Alaskan pollack skin	ACE inhibitor	1	LPG	[248-250]
7512	ACE inhibitor from Alaskan pollack skin	ACE inhibitor	5	GP	[257-258],[315-316],[375-376],[414-415],[506-507]
7513	ACE inhibitor from Alaskan pollack skin	ACE inhibitor	1	PL	[355-356]
7544	ACE inhibitor	ACE inhibitor	1	IW	[318-319]
7558	ACE inhibitor from buckwheat	ACE inhibitor	4	VK	[140-141],[212-213],[240-241],[299-300]
7559	ACE inhibitor from buckwheat	ACE inhibitor	1	PSY	[415-417]
7562	ACE inhibitor from soy hydrolysate	ACE inhibitor	2	IA	[385-386],[397-398]
7583	ACE inhibitor	ACE inhibitor	4	AF	[169-170],[177-178],[269-270],[483-484]
7585	ACE inhibitor	ACE inhibitor	3	LA	[168-169],[465-466],[482-483]

7586	ACE inhibitor	ACE inhibitor	3	KR	[69-70],[155-156],[206-207]
7587	ACE inhibitor	ACE inhibitor	2	VP	[180-181],[432-433]
7588	ACE inhibitor	ACE inhibitor	5	RA	[221-222],[268-269],[307-308],[381-382],[516-517]
7590	ACE inhibitor	ACE inhibitor	1	AA	[277-278]
7592	ACE inhibitor	ACE inhibitor	1	FR	[92-93]
7593	ACE inhibitor	ACE inhibitor	1	IF	[189-190]
7597	ACE inhibitor	ACE inhibitor	1	GM	[372-373]
7598	ACE inhibitor	ACE inhibitor	1	GA	[296-297]
7599	ACE inhibitor	ACE inhibitor	1	GL	[162-163]
7600	ACE inhibitor	ACE inhibitor	3	AG	[222-223],[434-435],[466-467]
7601	ACE inhibitor	ACE inhibitor	2	GH	[250-251],[435-436]
7602	ACE inhibitor	ACE inhibitor	1	HL	[401-402]
7603	ACE inhibitor	ACE inhibitor	6	GR	[40-41],[54-55],[56-57],[195-196],[393-394],[515-516]
7604	ACE inhibitor	ACE inhibitor	4	KG	[29-30],[39-40],[370-371],[487-488]
7605	ACE inhibitor	ACE inhibitor	1	FG	[495-496]
7606	ACE inhibitor	ACE inhibitor	1	DA	[185-186]
7607	ACE inhibitor	ACE inhibitor	1	GS	[52-53]
7610	ACE inhibitor	ACE inhibitor	3	GQ	[84-85],[343-344],[409-410]
7611	ACE inhibitor	ACE inhibitor	4	GK	[145-146],[323-324],[467-468],[496-497]
7612	ACE inhibitor	ACE inhibitor	3	GT	[197-198],[223-224],[327-328]
7613	ACE inhibitor	ACE inhibitor	2	WG	[51-52],[194-195]
7614	ACE inhibitor	ACE inhibitor	1	HG	[256-257]
7615	ACE inhibitor	ACE inhibitor	5	GE	[67-68],[260-261],[313-314],[391-392],[488-489]
7616	ACE inhibitor	ACE inhibitor	3	GG	[83-84],[259-260],[371-372]
7617	ACE inhibitor	ACE inhibitor	4	QG	[144-145],[214-215],[505-506],[514-515]
7618	ACE inhibitor	ACE inhibitor	6	SG	[53-54],[374-375],[390-391],[408-409],[413-414],[443-444]
7620	ACE inhibitor	ACE inhibitor	3	GD	[58-59],[117-118],[215-216]
7621	ACE inhibitor	ACE inhibitor	2	TG	[322-323],[326-327]
7622	ACE inhibitor	ACE inhibitor	5	EG	[116-117],[295-296],[314-315],[392-393],[458-459]
7623	ACE inhibitor	ACE inhibitor	2	EA	[276-277],[478-479]
7625	ACE inhibitor	ACE inhibitor	3	PG	[66-67],[249-250],[258-259]
7653	ACE inhibitor from wakame	ACE inhibitor	1	KYY	[33-35]

7680	ACE inhibitor from pea vicilin	ACE inhibitor	2	QK	[23-24],[86-87]
7681	ACE inhibitor from soy	ACE inhibitor	1	DG	[82-83]
7682	ACE inhibitor from garlic	ACE inhibitor	1	NY	[341-342]
7684	ACE inhibitor from garlic	ACE inhibitor	3	SY	[106-107],[364-365],[416-417]
7685	ACE inhibitor from garlic	ACE inhibitor	1	SF	[265-266]
7691	ACE inhibitor from wakame	ACE inhibitor	3	KY	[33-34],[97-98],[165-166]
7692	ACE inhibitor	ACE inhibitor	1	KF	[152-153]
7693	ACE inhibitor from wakame	ACE inhibitor	6	KL	[158-159],[236-237],[241-242],[285-286],[288-289],[419-420]
7697	ACE inhibitor from wakame	ACE inhibitor	3	YK	[35-36],[98-99],[417-418]
7698	ACE inhibitor from wakame	ACE inhibitor	1	NK	[205-206]
7741	ACE inhibitor	ACE inhibitor	2	RR	[102-103],[311-312]
7743	ACE inhibitor	ACE inhibitor	1	KA	[300-301]
7751	ACE inhibitor from shark meat hydrolysate	ACE inhibitor	1	CF	[452-453]
7826	ACE inhibitor	ACE inhibitor	2	EI	[396-397],[449-450]
7827	ACE inhibitor	ACE inhibitor	1	IE	[472-473]
7828	ACE inhibitor	ACE inhibitor	5	EV	[253-254],[273-274],[454-455],[473-474],[490-491]
7830	ACE inhibitor	ACE inhibitor	1	TE	[142-143]
7831	ACE inhibitor	ACE inhibitor	2	LQ	[243-244],[356-357]
7836	ACE inhibitor	ACE inhibitor	1	PP	[354-355]
7837	ACE inhibitor	ACE inhibitor	1	PQ	[175-176]
7838	ACE inhibitor	ACE inhibitor	1	EW	[50-51]
7839	ACE inhibitor	ACE inhibitor	1	ME	[475-476]
7840	ACE inhibitor	ACE inhibitor	10	EK	[32-33],[37-38],[68-69],[96-97],[164-165],[235-236],[287-288],[291-292],[411-412],[476-477]
7841	ACE inhibitor	ACE inhibitor	6	KE	[24-25],[36-37],[100-101],[303-304],[477-478],[480-481]
7842	ACE inhibitor	ACE inhibitor	1	HP	[436-437]
7843	ACE inhibitor	ACE inhibitor	1	PH	[400-401]
8185	ACE inhibitor	ACE inhibitor	1	TF	[328-329]
8193	ACE inhibitor	ACE inhibitor	2	AI	[297-298],[386-387]
9037	ACE inhibitor	ACE inhibitor	1	GKV	[145-147]
9042	ACE inhibitor	ACE inhibitor	1	AFL	[169-171]
9053	ACE inhibitor	ACE inhibitor	1	FYN	[377-379]
9064	ACE inhibitor	ACE inhibitor	2	LEK	[163-165],[286-288]

9072	ACE inhibitor	ACE inhibitor	1	DY	[352-353]
9073	ACE inhibitor	ACE inhibitor	2	TP	[224-225],[281-282]
9076	ACE inhibitor	ACE inhibitor	1	FQ	[504-505]
9077	ACE inhibitor	ACE inhibitor	2	YV	[129-130],[365-366]
9078	ACE inhibitor	ACE inhibitor	1	YE	[252-253]
9079	ACE inhibitor	ACE inhibitor	1	IL	[450-451]
9145	ACE inhibitor	ACE inhibitor	2	SGP	[374-376],[413-415]
9146	ACE inhibitor	ACE inhibitor	1	QGP	[505-507]
9173	ACE inhibitor	ACE inhibitor	5	RG	[55-56],[57-58],[161-162],[196-197],[312-313]
9184	ACE inhibitor	ACE inhibitor	2	ST	[62-63],[325-326]
9185	ACE inhibitor	ACE inhibitor	1	YN	[378-379]
9213	ACE inhibitor	ACE inhibitor	1	LR	[160-161]
9566	ACE inhibitor	ACE inhibitor	1	QP	[244-245]
9731	ACE inhibitor	ACE inhibitor	1	VVL	[148-150]
9742	ACE inhibitor	ACE inhibitor	1	EKR	[68-70]
3283	Antithrombotic peptide	antithrombotic	5	GP	[257-258],[315-316],[375-376],[414-415],[506-507]
3285	Antithrombotic peptide	antithrombotic	3	PG	[66-67],[249-250],[258-259]
3354	Antithrombotic peptide	antithrombotic	4	DEE	[20-22],[110-112],[118-120],[499-501]
3462		antithrombotic	1	GPGG	[257-260]
9660	Antithrombotic peptide	antithrombotic	1	RGD	[57-59]
2882	Immunostimulating peptide	immunomodulating	1	YG	[342-343]
3351	Stimulating vasoactive substance release	stimulating	10	EEE	[44-46],[45-47],[46-48],[47-49],[48-50],[111-113],[112-114],[113-115],[114-116],[122-124]
3355	Stimulating vasoactive substance release	stimulating	2	SSS	[403-405],[421-423]
8320	Glucose uptake stimulating peptide	stimulating	2	VL	[149-150],[274-275]
8321	Glucose uptake stimulating peptide	stimulating	1	LV	[171-172]
8322	Glucose uptake stimulating peptide	stimulating	4	IV	[228-229],[239-240],[298-299],[387-388]
8323	Glucose uptake stimulating peptide	stimulating	1	IL	[450-451]
8325	Glucose uptake stimulating peptide	stimulating	2	II	[217-218],[471-472]
8326	Glucose uptake stimulating peptide	stimulating	2	LL	[159-160],[242-243]
8329	Stimulating vasoactive substance release	stimulating	17	EE	[21-22],[44-45],[45-46],[46-47],[47-48],[48-49],[49-50],[111-112],[112-113],[113-114],[114-115],[115-116],[119-120],[122-123],[123-124],[489-490],[500-501]
8330	Stimulating vasoactive substance release	stimulating	1	SE	[348-349]

2890	neuropeptide	neuropeptide	3	GQ	[84-85],[343-344],[409-410]
9534	Kyotorphin	neuropeptide	2	YR	[166-167],[267-268]
2749	peptide regulating ion flow	regulating	1	DY	[352-353]
2753	peptide regulating the stomach mucosal membrane activity	regulating	5	GP	[257-258],[315-316],[375-376],[414-415],[506-507]
2754	peptide regulating the stomach mucosal membrane activity	regulating	3	PG	[66-67],[249-250],[258-259]
2755	peptide regulating the stomach mucosal membrane activity	regulating	1	GPGG	[257-260]
8318	Dvl protein binding	anticancer	1	VVV	[147-149]
3317		antioxidative	1	HL	[401-402]
7863	peptide from prawn muscle (Penaeus japonicus)	antioxidative	1	FKK	[332-334]
7872	peptide from soybean protein isolates: beta-conglycinin and glycinin	antioxidative	1	LY	[237-238]
7888	antioxidative peptide	antioxidative	3	EL	[5-6],[358-359],[481-482]
7931	synthetic peptide	antioxidative	1	KYY	[33-35]
7937	synthetic peptide	antioxidative	1	YYK	[34-36]
8029	synthetic peptide	antioxidative	1	PHL	[400-402]
8103	peptide derived from dried bonito	antioxidative	1	VKL	[240-242]
8114	peptide derived from sardinelle by-products proteins (Sardinella aurita)	antioxidative	1	GGE	[259-261]
8130	peptide derived from dried bonito	antioxidative	1	EAK	[478-480]
8134	peptide derived from dried bonito	antioxidative	2	KD	[334-335],[351-352]
8139	synthetic peptide	antioxidative	1	PEL	[4-6]
8215	Antioxidative peptide	antioxidative	6	IR	[202-203],[218-219],[220-221],[306-307],[424-425],[461-462]
8217	Antioxidative peptide	antioxidative	2	LK	[6-7],[279-280]
8484	Antioxidant peptide from as1-CN (98-100)	antioxidative	1	LLR	[159-161]
9082	Antioxidative peptide	antioxidative	2	WG	[51-52],[194-195]
9368	Antioxidative peptide	antioxidative	1	EQC	[25-27]
3751		bacterial permease ligand	5	KK	[38-39],[99-100],[333-334],[418-419],[468-469]
3164	laminin-like peptide	embryotoxic	1	RGD	[57-59]
4005		activating ubiquitin-mediated proteolysis	5	RA	[221-222],[268-269],[307-308],[381-382],[516-517]
4006	Ubiqitin-mediated proteolysis activating peptide	activating ubiquitin-mediated proteolysis	3	LA	[168-169],[465-466],[482-483]

9580	Hypolipidemic peptide	hypolipidemic	3	EF	[60-61],[349-350],[501-502]
9548	Alpha-glucosidase inhibitor	alpha-glucosidase inhibitor	1	YP	[353-354]
9650	Alpha-glucosidase inhibitor	alpha-glucosidase inhibitor	2	EA	[276-277],[478-479]
9651	Alpha-glucosidase inhibitor	alpha-glucosidase inhibitor	1	PP	[354-355]
9694	Alpha-glucosidase inhibitor	alpha-glucosidase inhibitor	2	PE	[4-5],[263-264]
9695	Alpha-glucosidase inhibitor	alpha-glucosidase inhibitor	2	AD	[186-187],[517-518]
3169	dipeptidyl peptidase IV inhibitor (DPP IV inhibitor)	dipeptidyl peptidase IV inhibitor	5	GP	[257-258],[315-316],[375-376],[414-415],[506-507]
3170	dipeptidyl peptidase IV inhibitor (DPP IV inhibitor)	dipeptidyl peptidase IV inhibitor	1	PP	[354-355]
3172	dipeptidyl peptidase IV inhibitor (DPP IV inhibitor)	dipeptidyl peptidase IV inhibitor	2	VA	[172-173],[441-442]
3174	dipeptidyl peptidase IV inhibitor (DPP IV inhibitor)	dipeptidyl peptidase IV inhibitor	1	KA	[300-301]
3175	dipeptidyl peptidase IV inhibitor (DPP IV inhibitor)	dipeptidyl peptidase IV inhibitor	3	LA	[168-169],[465-466],[482-483]
3179	dipeptidyl peptidase IV inhibitor (DPP IV inhibitor)	dipeptidyl peptidase IV inhibitor	1	PA	[433-434]
3180	dipeptidyl peptidase IV inhibitor (DPP IV inhibitor)	dipeptidyl peptidase IV inhibitor	2	LP	[150-151],[248-249]
3181	dipeptidyl peptidase IV inhibitor (DPP IV inhibitor)	dipeptidyl peptidase IV inhibitor	2	VP	[180-181],[432-433]
3182	dipeptidyl peptidase IV inhibitor (DPP IV inhibitor)	dipeptidyl peptidase IV inhibitor	2	LL	[159-160],[242-243]
3183	dipeptidyl peptidase IV inhibitor (DPP IV inhibitor)	dipeptidyl peptidase IV inhibitor	5	VV	[147-148],[148-149],[179-180],[388-389],[431-432]
8503	Dipeptidyl peptidase IV inhibitor (DPP IV inhibitor)	dipeptidyl peptidase IV inhibitor	2	TP	[224-225],[281-282]
8504	Dipeptidyl peptidase IV inhibitor (DPP IV inhibitor)	dipeptidyl peptidase IV inhibitor	1	WP	[319-320]
8520	dipeptidyl peptidase IV inhibitor (DPP IV inhibitor)	dipeptidyl peptidase IV inhibitor	1	HP	[436-437]
8521	dipeptidyl peptidase IV inhibitor (DPP IV inhibitor)	dipeptidyl peptidase IV inhibitor	1	YP	[353-354]
8524	dipeptidyl peptidase IV inhibitor (DPP IV inhibitor)	dipeptidyl peptidase IV inhibitor	1	GA	[296-297]
8525	dipeptidyl peptidase IV inhibitor (DPP IV inhibitor)	dipeptidyl peptidase IV inhibitor	2	IA	[385-386],[397-398]
8526	dipeptidyl peptidase IV inhibitor (DPP IV inhibitor)	dipeptidyl peptidase IV inhibitor	5	RA	[221-222],[268-269],[307-308],[381-382],[516-517]
8529	dipeptidyl peptidase IV inhibitor (DPP IV inhibitor)	dipeptidyl peptidase IV inhibitor	1	EP	[65-66]
8530	dipeptidyl peptidase IV inhibitor (DPP IV inhibitor)	dipeptidyl peptidase IV inhibitor	3	NP	[127-128],[174-175],[262-263]
8532	dipeptidyl peptidase IV inhibitor (DPP IV inhibitor)	dipeptidyl peptidase IV inhibitor	1	QP	[244-245]
8555	dipeptidyl peptidase IV inhibitor (DPP IV inhibitor)	dipeptidyl peptidase IV inhibitor	1	FL	[170-171]
8557	dipeptidyl peptidase IV inhibitor (DPP IV inhibitor)	dipeptidyl peptidase IV inhibitor	1	HL	[401-402]
8558	dipeptidyl peptidase IV inhibitor (DPP IV inhibitor)	dipeptidyl peptidase IV inhibitor	10	EK	[32-33],[37-38],[68-69],[96-97],[164-165],[235-236],[287-288],[291-292],[411-412],[476-477]
8559	dipeptidyl peptidase IV inhibitor (DPP IV inhibitor)	dipeptidyl peptidase IV inhibitor	1	AL	[278-279]
8561	dipeptidyl peptidase IV inhibitor (DPP IV inhibitor)	dipeptidyl peptidase IV inhibitor	1	GL	[162-163]
8609	dipeptidyl peptidase IV inhibitor (DPP IV inhibitor)	dipeptidyl peptidase IV inhibitor	1	WRQ	[509-511]

8637	dipeptidyl peptidase IV inhibitor (DPP IV inhibitor)	dipeptidyl peptidase IV inhibitor	1	AA	[277-278]
8638	dipeptidyl peptidase IV inhibitor (DPP IV inhibitor)	dipeptidyl peptidase IV inhibitor	1	PL	[355-356]
8652	dipeptidyl peptidase IV inhibitor (DPP IV inhibitor)	dipeptidyl peptidase IV inhibitor	1	PPL	[354-356]
8675	dipeptidyl peptidase IV inhibitor (DPP IV inhibitor)	dipeptidyl peptidase IV inhibitor	1	WR	[509-510]
8689	dipeptidyl peptidase IV inhibitor (DPP IV inhibitor)	dipeptidyl peptidase IV inhibitor	1	LQP	[243-245]
8696	dipeptidyl peptidase IV inhibitor (DPP IV inhibitor)	dipeptidyl peptidase IV inhibitor	1	YT	[463-464]
8697	dipeptidyl peptidase IV inhibitor (DPP IV inhibitor)	dipeptidyl peptidase IV inhibitor	2	WG	[51-52],[194-195]
8757	dipeptidyl peptidase IV inhibitor (DPP IV inhibitor)	dipeptidyl peptidase IV inhibitor	2	AD	[186-187],[517-518]
8758	dipeptidyl peptidase IV inhibitor (DPP IV inhibitor)	dipeptidyl peptidase IV inhibitor	1	AE	[457-458]
8759	dipeptidyl peptidase IV inhibitor (DPP IV inhibitor)	dipeptidyl peptidase IV inhibitor	4	AF	[169-170],[177-178],[269-270],[483-484]
8760	dipeptidyl peptidase IV inhibitor (DPP IV inhibitor)	dipeptidyl peptidase IV inhibitor	3	AG	[222-223],[434-435],[466-467]
8762	dipeptidyl peptidase IV inhibitor (DPP IV inhibitor)	dipeptidyl peptidase IV inhibitor	2	AS	[301-302],[442-443]
8763	dipeptidyl peptidase IV inhibitor (DPP IV inhibitor)	dipeptidyl peptidase IV inhibitor	1	AT	[382-383]
8767	dipeptidyl peptidase IV inhibitor (DPP IV inhibitor)	dipeptidyl peptidase IV inhibitor	2	DP	[3-4],[335-336]
8770	dipeptidyl peptidase IV inhibitor (DPP IV inhibitor)	dipeptidyl peptidase IV inhibitor	5	EG	[116-117],[295-296],[314-315],[392-393],[458-459]
8771	dipeptidyl peptidase IV inhibitor (DPP IV inhibitor)	dipeptidyl peptidase IV inhibitor	2	EH	[104-105],[134-135]
8772	dipeptidyl peptidase IV inhibitor (DPP IV inhibitor)	dipeptidyl peptidase IV inhibitor	2	EI	[396-397],[449-450]
8773	dipeptidyl peptidase IV inhibitor (DPP IV inhibitor)	dipeptidyl peptidase IV inhibitor	3	ES	[208-209],[264-265],[347-348]
8775	dipeptidyl peptidase IV inhibitor (DPP IV inhibitor)	dipeptidyl peptidase IV inhibitor	5	EV	[253-254],[273-274],[454-455],[473-474],[490-491]
8776	dipeptidyl peptidase IV inhibitor (DPP IV inhibitor)	dipeptidyl peptidase IV inhibitor	1	EW	[50-51]
8778	dipeptidyl peptidase IV inhibitor (DPP IV inhibitor)	dipeptidyl peptidase IV inhibitor	1	FN	[329-330]
8779	dipeptidyl peptidase IV inhibitor (DPP IV inhibitor)	dipeptidyl peptidase IV inhibitor	1	FQ	[504-505]
8780	dipeptidyl peptidase IV inhibitor (DPP IV inhibitor)	dipeptidyl peptidase IV inhibitor	1	FR	[92-93]
8781	dipeptidyl peptidase IV inhibitor (DPP IV inhibitor)	dipeptidyl peptidase IV inhibitor	5	GE	[67-68],[260-261],[313-314],[391-392],[488-489]
8783	dipeptidyl peptidase IV inhibitor (DPP IV inhibitor)	dipeptidyl peptidase IV inhibitor	3	GG	[83-84],[259-260],[371-372]
8784	dipeptidyl peptidase IV inhibitor (DPP IV inhibitor)	dipeptidyl peptidase IV inhibitor	2	GH	[250-251],[435-436]
8790	dipeptidyl peptidase IV inhibitor (DPP IV inhibitor)	dipeptidyl peptidase IV inhibitor	1	HE	[64-65]
8791	dipeptidyl peptidase IV inhibitor (DPP IV inhibitor)	dipeptidyl peptidase IV inhibitor	1	HF	[135-136]
8795	dipeptidyl peptidase IV inhibitor (DPP IV inhibitor)	dipeptidyl peptidase IV inhibitor	1	HS	[105-106]
8799	dipeptidyl peptidase IV inhibitor (DPP IV inhibitor)	dipeptidyl peptidase IV inhibitor	1	HY	[251-252]
8801	dipeptidyl peptidase IV inhibitor (DPP IV inhibitor)	dipeptidyl peptidase IV inhibitor	2	II	[217-218],[471-472]
8802	dipeptidyl peptidase IV inhibitor (DPP IV inhibitor)	dipeptidyl peptidase IV inhibitor	1	IL	[450-451]

8803	dipeptidyl peptidase IV inhibitor (DPP IV inhibitor)	dipeptidyl peptidase IV inhibitor	1	IM	[361-362]
8806	dipeptidyl peptidase IV inhibitor (DPP IV inhibitor)	dipeptidyl peptidase IV inhibitor	6	IR	[202-203],[218-219],[220-221],[306-307],[424-425],[461-462]
8807	dipeptidyl peptidase IV inhibitor (DPP IV inhibitor)	dipeptidyl peptidase IV inhibitor	1	IW	[318-319]
8808	dipeptidyl peptidase IV inhibitor (DPP IV inhibitor)	dipeptidyl peptidase IV inhibitor	6	KE	[24-25],[36-37],[100-101],[303-304],[477-478],[480-481]
8809	dipeptidyl peptidase IV inhibitor (DPP IV inhibitor)	dipeptidyl peptidase IV inhibitor	1	KF	[152-153]
8810	dipeptidyl peptidase IV inhibitor (DPP IV inhibitor)	dipeptidyl peptidase IV inhibitor	4	KG	[29-30],[39-40],[370-371],[487-488]
8811	dipeptidyl peptidase IV inhibitor (DPP IV inhibitor)	dipeptidyl peptidase IV inhibitor	1	KH	[10-11]
8812	dipeptidyl peptidase IV inhibitor (DPP IV inhibitor)	dipeptidyl peptidase IV inhibitor	2	KI	[201-202],[384-385]
8813	dipeptidyl peptidase IV inhibitor (DPP IV inhibitor)	dipeptidyl peptidase IV inhibitor	5	KK	[38-39],[99-100],[333-334],[418-419],[468-469]
8814	dipeptidyl peptidase IV inhibitor (DPP IV inhibitor)	dipeptidyl peptidase IV inhibitor	3	KR	[69-70],[155-156],[206-207]
8815	dipeptidyl peptidase IV inhibitor (DPP IV inhibitor)	dipeptidyl peptidase IV inhibitor	2	KS	[324-325],[412-413]
8816	dipeptidyl peptidase IV inhibitor (DPP IV inhibitor)	dipeptidyl peptidase IV inhibitor	4	KT	[1-2],[141-142],[280-281],[485-486]
8817	dipeptidyl peptidase IV inhibitor (DPP IV inhibitor)	dipeptidyl peptidase IV inhibitor	3	KV	[14-15],[146-147],[493-494]
8818	dipeptidyl peptidase IV inhibitor (DPP IV inhibitor)	dipeptidyl peptidase IV inhibitor	1	KW	[508-509]
8819	dipeptidyl peptidase IV inhibitor (DPP IV inhibitor)	dipeptidyl peptidase IV inhibitor	3	KY	[33-34],[97-98],[165-166]
8825	dipeptidyl peptidase IV inhibitor (DPP IV inhibitor)	dipeptidyl peptidase IV inhibitor	1	LV	[171-172]
8826	dipeptidyl peptidase IV inhibitor (DPP IV inhibitor)	dipeptidyl peptidase IV inhibitor	1	ME	[475-476]
8831	dipeptidyl peptidase IV inhibitor (DPP IV inhibitor)	dipeptidyl peptidase IV inhibitor	1	MK	[75-76]
8834	dipeptidyl peptidase IV inhibitor (DPP IV inhibitor)	dipeptidyl peptidase IV inhibitor	1	MN	[210-211]
8837	dipeptidyl peptidase IV inhibitor (DPP IV inhibitor)	dipeptidyl peptidase IV inhibitor	1	MV	[362-363]
8839	dipeptidyl peptidase IV inhibitor (DPP IV inhibitor)	dipeptidyl peptidase IV inhibitor	1	NA	[456-457]
8841	dipeptidyl peptidase IV inhibitor (DPP IV inhibitor)	dipeptidyl peptidase IV inhibitor	1	NE	[234-235]
8844	dipeptidyl peptidase IV inhibitor (DPP IV inhibitor)	dipeptidyl peptidase IV inhibitor	1	NH	[182-183]
8845	dipeptidyl peptidase IV inhibitor (DPP IV inhibitor)	dipeptidyl peptidase IV inhibitor	3	NL	[247-248],[330-331],[447-448]
8847	dipeptidyl peptidase IV inhibitor (DPP IV inhibitor)	dipeptidyl peptidase IV inhibitor	1	NN	[340-341]
8848	dipeptidyl peptidase IV inhibitor (DPP IV inhibitor)	dipeptidyl peptidase IV inhibitor	1	NQ	[445-446]
8850	dipeptidyl peptidase IV inhibitor (DPP IV inhibitor)	dipeptidyl peptidase IV inhibitor	1	NT	[230-231]
8851	dipeptidyl peptidase IV inhibitor (DPP IV inhibitor)	dipeptidyl peptidase IV inhibitor	1	NV	[211-212]
8853	dipeptidyl peptidase IV inhibitor (DPP IV inhibitor)	dipeptidyl peptidase IV inhibitor	1	NY	[341-342]
8854	dipeptidyl peptidase IV inhibitor (DPP IV inhibitor)	dipeptidyl peptidase IV inhibitor	4	PF	[225-226],[320-321],[376-377],[437-438]
8855	dipeptidyl peptidase IV inhibitor (DPP IV inhibitor)	dipeptidyl peptidase IV inhibitor	3	PG	[66-67],[249-250],[258-259]
8856	dipeptidyl peptidase IV inhibitor (DPP IV inhibitor)	dipeptidyl peptidase IV inhibitor	1	PH	[400-401]

8858	dipeptidyl peptidase IV inhibitor (DPP IV inhibitor)	dipeptidyl peptidase IV inhibitor	2	PK	[151-152],[507-508]
8860	dipeptidyl peptidase IV inhibitor (DPP IV inhibitor)	dipeptidyl peptidase IV inhibitor	1	PN	[181-182]
8861	dipeptidyl peptidase IV inhibitor (DPP IV inhibitor)	dipeptidyl peptidase IV inhibitor	1	PQ	[175-176]
8862	dipeptidyl peptidase IV inhibitor (DPP IV inhibitor)	dipeptidyl peptidase IV inhibitor	3	PS	[316-317],[336-337],[415-416]
8864	dipeptidyl peptidase IV inhibitor (DPP IV inhibitor)	dipeptidyl peptidase IV inhibitor	1	PV	[245-246]
8866	dipeptidyl peptidase IV inhibitor (DPP IV inhibitor)	dipeptidyl peptidase IV inhibitor	1	PY	[128-129]
8867	dipeptidyl peptidase IV inhibitor (DPP IV inhibitor)	dipeptidyl peptidase IV inhibitor	1	QA	[176-177]
8868	dipeptidyl peptidase IV inhibitor (DPP IV inhibitor)	dipeptidyl peptidase IV inhibitor	3	QD	[81-82],[293-294],[498-499]
8869	dipeptidyl peptidase IV inhibitor (DPP IV inhibitor)	dipeptidyl peptidase IV inhibitor	5	QE	[43-44],[95-96],[121-122],[357-358],[410-411]
8871	dipeptidyl peptidase IV inhibitor (DPP IV inhibitor)	dipeptidyl peptidase IV inhibitor	4	QG	[144-145],[214-215],[505-506],[514-515]
8872	dipeptidyl peptidase IV inhibitor (DPP IV inhibitor)	dipeptidyl peptidase IV inhibitor	1	QH	[511-512]
8873	dipeptidyl peptidase IV inhibitor (DPP IV inhibitor)	dipeptidyl peptidase IV inhibitor	1	QI	[305-306]
8874	dipeptidyl peptidase IV inhibitor (DPP IV inhibitor)	dipeptidyl peptidase IV inhibitor	2	QL	[88-89],[344-345]
8875	dipeptidyl peptidase IV inhibitor (DPP IV inhibitor)	dipeptidyl peptidase IV inhibitor	1	QN	[446-447]
8876	dipeptidyl peptidase IV inhibitor (DPP IV inhibitor)	dipeptidyl peptidase IV inhibitor	2	QQ	[85-86],[513-514]
8877	dipeptidyl peptidase IV inhibitor (DPP IV inhibitor)	dipeptidyl peptidase IV inhibitor	1	QS	[338-339]
8881	dipeptidyl peptidase IV inhibitor (DPP IV inhibitor)	dipeptidyl peptidase IV inhibitor	1	QY	[18-19]
8882	dipeptidyl peptidase IV inhibitor (DPP IV inhibitor)	dipeptidyl peptidase IV inhibitor	5	RG	[55-56],[57-58],[161-162],[196-197],[312-313]
8884	dipeptidyl peptidase IV inhibitor (DPP IV inhibitor)	dipeptidyl peptidase IV inhibitor	1	RI	[219-220]
8886	dipeptidyl peptidase IV inhibitor (DPP IV inhibitor)	dipeptidyl peptidase IV inhibitor	3	RL	[70-71],[167-168],[394-395]
8889	dipeptidyl peptidase IV inhibitor (DPP IV inhibitor)	dipeptidyl peptidase IV inhibitor	2	RR	[102-103],[311-312]
8891	dipeptidyl peptidase IV inhibitor (DPP IV inhibitor)	dipeptidyl peptidase IV inhibitor	1	SF	[265-266]
8893	dipeptidyl peptidase IV inhibitor (DPP IV inhibitor)	dipeptidyl peptidase IV inhibitor	3	SI	[188-189],[317-318],[423-424]
8894	dipeptidyl peptidase IV inhibitor (DPP IV inhibitor)	dipeptidyl peptidase IV inhibitor	3	SK	[157-158],[302-303],[405-406]
8895	dipeptidyl peptidase IV inhibitor (DPP IV inhibitor)	dipeptidyl peptidase IV inhibitor	1	SV	[428-429]
8896	dipeptidyl peptidase IV inhibitor (DPP IV inhibitor)	dipeptidyl peptidase IV inhibitor	1	SW	[193-194]
8897	dipeptidyl peptidase IV inhibitor (DPP IV inhibitor)	dipeptidyl peptidase IV inhibitor	3	SY	[106-107],[364-365],[416-417]
8898	dipeptidyl peptidase IV inhibitor (DPP IV inhibitor)	dipeptidyl peptidase IV inhibitor	3	TD	[2-3],[231-232],[426-427]
8899	dipeptidyl peptidase IV inhibitor (DPP IV inhibitor)	dipeptidyl peptidase IV inhibitor	1	TE	[142-143]
8900	dipeptidyl peptidase IV inhibitor (DPP IV inhibitor)	dipeptidyl peptidase IV inhibitor	1	TF	[328-329]
8901	dipeptidyl peptidase IV inhibitor (DPP IV inhibitor)	dipeptidyl peptidase IV inhibitor	2	TG	[322-323],[326-327]
8902	dipeptidyl peptidase IV inhibitor (DPP IV inhibitor)	dipeptidyl peptidase IV inhibitor	1	TH	[63-64]

8903	dipeptidyl peptidase IV inhibitor (DPP IV inhibitor)	dipeptidyl peptidase IV inhibitor	1	TI	[198-199]
8904	dipeptidyl peptidase IV inhibitor (DPP IV inhibitor)	dipeptidyl peptidase IV inhibitor	5	TK	[154-155],[200-201],[369-370],[383-384],[486-487]
8905	dipeptidyl peptidase IV inhibitor (DPP IV inhibitor)	dipeptidyl peptidase IV inhibitor	1	TL	[464-465]
8909	dipeptidyl peptidase IV inhibitor (DPP IV inhibitor)	dipeptidyl peptidase IV inhibitor	1	TR	[138-139]
8911	dipeptidyl peptidase IV inhibitor (DPP IV inhibitor)	dipeptidyl peptidase IV inhibitor	1	TT	[137-138]

Table S21. Profile of potential biological activity of fragments of protein Pis v 4.0101.

ID	Name of peptide	Activity	Number	Sequence	Location
3166	Diprotin B	antiamnestic	1	VPL	[185-187]
3461	Prolyl endopeptidase inhibitor	antiamnestic	1	GP	[181-182]
3257	beta-lactokinin	ACE inhibitor	1	RL	[17-18]
3381	ACE inhibitor	ACE inhibitor	1	LY	[224-225]
3404	ACE inhibitor	ACE inhibitor	1	VRP	[204-206]
3486	ACE inhibitor from sake lees	ACE inhibitor	2	VW	[155-156],[192-193]
3553	ACE inhibitor	ACE inhibitor	1	YG	[39-40]
3563	ACE inhibitor	ACE inhibitor	1	AY	[196-197]
7512	ACE inhibitor from Alaskan pollack skin	ACE inhibitor	1	GP	[181-182]
7513	ACE inhibitor from Alaskan pollack skin	ACE inhibitor	2	PL	[176-177],[186-187]
7542	ACE inhibitor	ACE inhibitor	1	DLP	[34-36]
7544	ACE inhibitor	ACE inhibitor	1	IW	[212-213]
7554	ACE inhibitor from Tricholoma giganteum	ACE inhibitor	1	GEP	[117-119]
7558	ACE inhibitor from buckwheat	ACE inhibitor	1	VK	[86-87]
7579	ACE inhibitor	ACE inhibitor	2	GW	[125-126],[153-154]
7582	ACE inhibitor	ACE inhibitor	1	RP	[205-206]
7587	ACE inhibitor	ACE inhibitor	1	VP	[185-186]
7589	ACE inhibitor	ACE inhibitor	1	YA	[220-221]
7590	ACE inhibitor	ACE inhibitor	1	AA	[147-148]
7593	ACE inhibitor	ACE inhibitor	1	IF	[104-105]
7596	ACE inhibitor	ACE inhibitor	1	GI	[189-190]
7598	ACE inhibitor	ACE inhibitor	2	GA	[40-41],[146-147]
7599	ACE inhibitor	ACE inhibitor	3	GL	[19-20],[26-27],[158-159]
7600	ACE inhibitor	ACE inhibitor	1	AG	[221-222]
7601	ACE inhibitor	ACE inhibitor	1	GH	[98-99]
7604	ACE inhibitor	ACE inhibitor	2	KG	[79-80],[180-181]
7605	ACE inhibitor	ACE inhibitor	1	FG	[132-133]
7606	ACE inhibitor	ACE inhibitor	1	DA	[81-82]
7607	ACE inhibitor	ACE inhibitor	3	GS	[122-123],[133-134],[151-152]

7614	ACE inhibitor	ACE inhibitor	1	HG	[121-122]
7615	ACE inhibitor	ACE inhibitor	2	GE	[117-118],[222-223]
7616	ACE inhibitor	ACE inhibitor	4	GG	[96-97],[97-98],[115-116],[116-117]
7617	ACE inhibitor	ACE inhibitor	1	QG	[150-151]
7618	ACE inhibitor	ACE inhibitor	1	SG	[152-153]
7619	ACE inhibitor	ACE inhibitor	4	LG	[18-19],[124-125],[157-158],[188-189]
7620	ACE inhibitor	ACE inhibitor	1	GD	[80-81]
7622	ACE inhibitor	ACE inhibitor	2	EG	[114-115],[145-146]
7623	ACE inhibitor	ACE inhibitor	1	EA	[136-137]
7624	ACE inhibitor	ACE inhibitor	1	NG	[95-96]
7628	ACE inhibitor from k-CN (fr. 67-68)	ACE inhibitor	2	VR	[24-25],[204-205]
7655	ACE inhibitor from wakame	ACE inhibitor	1	YNK	[66-68]
7680	ACE inhibitor from pea vicilin	ACE inhibitor	2	QK	[56-57],[226-227]
7682	ACE inhibitor from garlic	ACE inhibitor	1	NY	[65-66]
7683	ACE inhibitor from garlic	ACE inhibitor	1	NF	[131-132]
7684	ACE inhibitor from garlic	ACE inhibitor	1	SY	[5-6]
7691	ACE inhibitor from wakame	ACE inhibitor	1	KY	[219-220]
7692	ACE inhibitor	ACE inhibitor	1	KF	[93-94]
7693	ACE inhibitor from wakame	ACE inhibitor	2	KL	[87-88],[165-166]
7697	ACE inhibitor from wakame	ACE inhibitor	1	YK	[201-202]
7698	ACE inhibitor from wakame	ACE inhibitor	2	NK	[67-68],[78-79]
7743	ACE inhibitor	ACE inhibitor	1	KA	[68-69]
7752	ACE inhibitor from shark meat hydrolysate	ACE inhibitor	1	EY	[38-39]
7826	ACE inhibitor	ACE inhibitor	1	EI	[49-50]
7829	ACE inhibitor	ACE inhibitor	1	VE	[168-169]
7830	ACE inhibitor	ACE inhibitor	1	TE	[13-14]
7831	ACE inhibitor	ACE inhibitor	4	LQ	[27-28],[88-89],[149-150],[199-200]
7836	ACE inhibitor	ACE inhibitor	1	PP	[119-120]
7839	ACE inhibitor	ACE inhibitor	1	ME	[135-136]
7841	ACE inhibitor	ACE inhibitor	2	KE	[161-162],[227-228]
7843	ACE inhibitor	ACE inhibitor	1	PH	[120-121]
8182	ACE Inhibitor	ACE inhibitor	1	ALEP	[41-44]

8185	ACE inhibitor	ACE inhibitor	1	TF	[29-30]
8193	ACE inhibitor	ACE inhibitor	4	AI	[45-46],[76-77],[91-92],[127-128]
8951	ACE inhibitor	ACE inhibitor	1	AV	[84-85]
9061	ACE inhibitor	ACE inhibitor	1	LGI	[188-190]
9068	ACE inhibitor	ACE inhibitor	1	VTR	[7-9]
9072	ACE inhibitor	ACE inhibitor	1	DY	[207-208]
9073	ACE inhibitor	ACE inhibitor	1	TP	[110-111]
9077	ACE inhibitor	ACE inhibitor	1	YV	[6-7]
9078	ACE inhibitor	ACE inhibitor	1	YE	[37-38]
9089	ACE inhibitor	ACE inhibitor	1	WA	[126-127]
9107	ACE inhibitor	ACE inhibitor	1	WL	[156-157]
9148	ACE inhibitor	ACE inhibitor	1	KGP	[180-182]
9173	ACE inhibitor	ACE inhibitor	1	RG	[25-26]
9185	ACE inhibitor	ACE inhibitor	1	YN	[66-67]
9196	ACE inhibitor	ACE inhibitor	1	AVV	[84-86]
9213	ACE inhibitor	ACE inhibitor	1	LR	[16-17]
3283	Antithrombotic peptide	antithrombotic	1	GP	[181-182]
2882	Immunostimulating peptide	immunomodulating	1	YG	[39-40]
3350	Stimulating vasoactive substance release	stimulating	1	VPL	[185-187]
8321	Glucose uptake stimulating peptide	stimulating	3	LV	[166-167],[177-178],[184-185]
8324	Glucose uptake stimulating peptide	stimulating	1	LI	[138-139]
8326	Glucose uptake stimulating peptide	stimulating	2	LL	[3-4],[187-188]
8330	Stimulating vasoactive substance release	stimulating	2	SE	[48-49],[113-114]
8310	Anxiolytic peptide	neuropeptide	2	YL	[198-199],[208-209]
2749	peptide regulating ion flow	regulating	1	DY	[207-208]
2753	peptide regulating the stomach mucosal membrane activity	regulating	1	GP	[181-182]
3302		antioxidative	1	LHH	[53-55]
3305		antioxidative	1	LH	[53-54]
3319		antioxidative	2	HH	[54-55],[58-59]
7866	peptide from Okara protein	antioxidative	1	AY	[196-197]
7872	peptide from soybean protein isolates: beta-conglycinin and glycinin	antioxidative	1	LY	[224-225]
7888	antioxidative peptide	antioxidative	1	EL	[223-224]

7928	synthetic peptide	antioxidative	1	YEY	[37-39]
7941	synthetic peptide	antioxidative	1	YYL	[197-199]
7953	synthetic peptide	antioxidative	1	AYY	[196-198]
7986	synthetic peptide	antioxidative	1	KHH	[57-59]
8026	synthetic peptide	antioxidative	1	PHG	[120-122]
8103	peptide derived from dried bonito	antioxidative	1	VKL	[86-88]
8104	peptide derived from dried bonito	antioxidative	1	VVKL	[85-88]
8114	peptide derived from sardinelle by-products proteins (Sardinella aurita)	antioxidative	1	GGE	[116-118]
8133	peptide derived from dried bonito	antioxidative	1	KVI	[214-216]
8217	Antioxidative peptide	antioxidative	2	LK	[20-21],[209-210]
8219	antioxidative peptide	antioxidative	1	TY	[61-62]
8461	Antioxidant peptide from marine bivalve (Mactra veneriformis)	antioxidative	2	VW	[155-156],[192-193]
8983	Antioxidative peptide	antioxidative	1	GAA	[146-148]
3751		bacterial permease ligand	1	KK	[164-165]
4007	Peptide activating ubiquitin-mediated proteolysis	activating ubiquitin-mediated proteolysis	1	WA	[126-127]
9387	Alpha-glucosidase inhibitor	alpha-glucosidase inhibitor	2	VW	[155-156],[192-193]
9650	Alpha-glucosidase inhibitor	alpha-glucosidase inhibitor	1	EA	[136-137]
9651	Alpha-glucosidase inhibitor	alpha-glucosidase inhibitor	1	PP	[119-120]
9693	Alpha-glucosidase inhibitor	alpha-glucosidase inhibitor	1	VE	[168-169]
3169	dipeptidyl peptidase IV inhibitor (DPP IV inhibitor)	dipeptidyl peptidase IV inhibitor	1	GP	[181-182]
3170	dipeptidyl peptidase IV inhibitor (DPP IV inhibitor)	dipeptidyl peptidase IV inhibitor	1	PP	[119-120]
3173	dipeptidyl peptidase IV inhibitor (DPP IV inhibitor)	dipeptidyl peptidase IV inhibitor	1	MA	[1-2]
3174	dipeptidyl peptidase IV inhibitor (DPP IV inhibitor)	dipeptidyl peptidase IV inhibitor	1	KA	[68-69]
3179	dipeptidyl peptidase IV inhibitor (DPP IV inhibitor)	dipeptidyl peptidase IV inhibitor	1	PA	[44-45]
3180	dipeptidyl peptidase IV inhibitor (DPP IV inhibitor)	dipeptidyl peptidase IV inhibitor	2	LP	[32-33],[35-36]
3181	dipeptidyl peptidase IV inhibitor (DPP IV inhibitor)	dipeptidyl peptidase IV inhibitor	1	VP	[185-186]
3182	dipeptidyl peptidase IV inhibitor (DPP IV inhibitor)	dipeptidyl peptidase IV inhibitor	2	LL	[3-4],[187-188]
3183	dipeptidyl peptidase IV inhibitor (DPP IV inhibitor)	dipeptidyl peptidase IV inhibitor	2	VV	[85-86],[167-168]
3184	dipeptidyl peptidase IV inhibitor (DPP IV inhibitor)	dipeptidyl peptidase IV inhibitor	1	HA	[195-196]
8347	dipeptidyl peptidase IV inhibitor (DPP-IV inhibitor)	dipeptidyl peptidase IV inhibitor	1	VPL	[185-187]
8503	Dipeptidyl peptidase IV inhibitor (DPP IV inhibitor)	dipeptidyl peptidase IV inhibitor	1	TP	[110-111]
8518	dipeptidyl peptidase IV inhibitor (DPP IV inhibitor)	dipeptidyl peptidase IV inhibitor	1	RP	[205-206]

8524	dipeptidyl peptidase IV inhibitor (DPP IV inhibitor)	dipeptidyl peptidase IV inhibitor	2	GA	[40-41],[146-147]
8528	dipeptidyl peptidase IV inhibitor (DPP IV inhibitor)	dipeptidyl peptidase IV inhibitor	1	WA	[126-127]
8529	dipeptidyl peptidase IV inhibitor (DPP IV inhibitor)	dipeptidyl peptidase IV inhibitor	2	EP	[43-44],[118-119]
8531	dipeptidyl peptidase IV inhibitor (DPP IV inhibitor)	dipeptidyl peptidase IV inhibitor	1	TA	[171-172]
8556	dipeptidyl peptidase IV inhibitor (DPP IV inhibitor)	dipeptidyl peptidase IV inhibitor	1	WV	[154-155]
8559	dipeptidyl peptidase IV inhibitor (DPP IV inhibitor)	dipeptidyl peptidase IV inhibitor	5	AL	[2-3],[41-42],[69-70],[137-138],[148-149]
8560	dipeptidyl peptidase IV inhibitor (DPP IV inhibitor)	dipeptidyl peptidase IV inhibitor	3	SL	[15-16],[123-124],[183-184]
8561	dipeptidyl peptidase IV inhibitor (DPP IV inhibitor)	dipeptidyl peptidase IV inhibitor	3	GL	[19-20],[26-27],[158-159]
8594	dipeptidyl peptidase IV inhibitor (DPP IV inhibitor)	dipeptidyl peptidase IV inhibitor	2	VR	[24-25],[204-205]
8637	dipeptidyl peptidase IV inhibitor (DPP IV inhibitor)	dipeptidyl peptidase IV inhibitor	1	AA	[147-148]
8638	dipeptidyl peptidase IV inhibitor (DPP IV inhibitor)	dipeptidyl peptidase IV inhibitor	2	PL	[176-177],[186-187]
8676	dipeptidyl peptidase IV inhibitor (DPP IV inhibitor)	dipeptidyl peptidase IV inhibitor	3	WK	[106-107],[213-214],[218-219]
8677	dipeptidyl peptidase IV inhibitor (DPP IV inhibitor)	dipeptidyl peptidase IV inhibitor	1	WL	[156-157]
8691	dipeptidyl peptidase IV inhibitor (DPP IV inhibitor)	dipeptidyl peptidase IV inhibitor	1	WE	[193-194]
8758	dipeptidyl peptidase IV inhibitor (DPP IV inhibitor)	dipeptidyl peptidase IV inhibitor	1	AE	[144-145]
8760	dipeptidyl peptidase IV inhibitor (DPP IV inhibitor)	dipeptidyl peptidase IV inhibitor	1	AG	[221-222]
8762	dipeptidyl peptidase IV inhibitor (DPP IV inhibitor)	dipeptidyl peptidase IV inhibitor	1	AS	[82-83]
8764	dipeptidyl peptidase IV inhibitor (DPP IV inhibitor)	dipeptidyl peptidase IV inhibitor	1	AV	[84-85]
8765	dipeptidyl peptidase IV inhibitor (DPP IV inhibitor)	dipeptidyl peptidase IV inhibitor	1	AY	[196-197]
8767	dipeptidyl peptidase IV inhibitor (DPP IV inhibitor)	dipeptidyl peptidase IV inhibitor	1	DP	[175-176]
8768	dipeptidyl peptidase IV inhibitor (DPP IV inhibitor)	dipeptidyl peptidase IV inhibitor	1	DQ	[74-75]
8770	dipeptidyl peptidase IV inhibitor (DPP IV inhibitor)	dipeptidyl peptidase IV inhibitor	2	EG	[114-115],[145-146]
8771	dipeptidyl peptidase IV inhibitor (DPP IV inhibitor)	dipeptidyl peptidase IV inhibitor	1	EH	[194-195]
8772	dipeptidyl peptidase IV inhibitor (DPP IV inhibitor)	dipeptidyl peptidase IV inhibitor	1	EI	[49-50]
8773	dipeptidyl peptidase IV inhibitor (DPP IV inhibitor)	dipeptidyl peptidase IV inhibitor	2	ES	[14-15],[162-163]
8774	dipeptidyl peptidase IV inhibitor (DPP IV inhibitor)	dipeptidyl peptidase IV inhibitor	1	ET	[169-170]
8777	dipeptidyl peptidase IV inhibitor (DPP IV inhibitor)	dipeptidyl peptidase IV inhibitor	1	EY	[38-39]
8778	dipeptidyl peptidase IV inhibitor (DPP IV inhibitor)	dipeptidyl peptidase IV inhibitor	1	FN	[94-95]
8781	dipeptidyl peptidase IV inhibitor (DPP IV inhibitor)	dipeptidyl peptidase IV inhibitor	2	GE	[117-118],[222-223]
8783	dipeptidyl peptidase IV inhibitor (DPP IV inhibitor)	dipeptidyl peptidase IV inhibitor	4	GG	[96-97],[97-98],[115-116],[116-117]
8784	dipeptidyl peptidase IV inhibitor (DPP IV inhibitor)	dipeptidyl peptidase IV inhibitor	1	GH	[98-99]
8785	dipeptidyl peptidase IV inhibitor (DPP IV inhibitor)	dipeptidyl peptidase IV inhibitor	1	GI	[189-190]

8787	dipeptidyl peptidase IV inhibitor (DPP IV inhibitor)	dipeptidyl peptidase IV inhibitor	2	GW	[125-126],[153-154]
8792	dipeptidyl peptidase IV inhibitor (DPP IV inhibitor)	dipeptidyl peptidase IV inhibitor	2	HH	[54-55],[58-59]
8793	dipeptidyl peptidase IV inhibitor (DPP IV inhibitor)	dipeptidyl peptidase IV inhibitor	1	HI	[99-100]
8795	dipeptidyl peptidase IV inhibitor (DPP IV inhibitor)	dipeptidyl peptidase IV inhibitor	1	HS	[102-103]
8797	dipeptidyl peptidase IV inhibitor (DPP IV inhibitor)	dipeptidyl peptidase IV inhibitor	1	HV	[23-24]
8803	dipeptidyl peptidase IV inhibitor (DPP IV inhibitor)	dipeptidyl peptidase IV inhibitor	1	IM	[50-51]
8804	dipeptidyl peptidase IV inhibitor (DPP IV inhibitor)	dipeptidyl peptidase IV inhibitor	3	IN	[77-78],[100-101],[216-217]
8805	dipeptidyl peptidase IV inhibitor (DPP IV inhibitor)	dipeptidyl peptidase IV inhibitor	1	IQ	[139-140]
8807	dipeptidyl peptidase IV inhibitor (DPP IV inhibitor)	dipeptidyl peptidase IV inhibitor	1	IW	[212-213]
8808	dipeptidyl peptidase IV inhibitor (DPP IV inhibitor)	dipeptidyl peptidase IV inhibitor	2	KE	[161-162],[227-228]
8809	dipeptidyl peptidase IV inhibitor (DPP IV inhibitor)	dipeptidyl peptidase IV inhibitor	1	KF	[93-94]
8810	dipeptidyl peptidase IV inhibitor (DPP IV inhibitor)	dipeptidyl peptidase IV inhibitor	2	KG	[79-80],[180-181]
8811	dipeptidyl peptidase IV inhibitor (DPP IV inhibitor)	dipeptidyl peptidase IV inhibitor	1	KH	[57-58]
8813	dipeptidyl peptidase IV inhibitor (DPP IV inhibitor)	dipeptidyl peptidase IV inhibitor	1	KK	[164-165]
8815	dipeptidyl peptidase IV inhibitor (DPP IV inhibitor)	dipeptidyl peptidase IV inhibitor	1	KS	[21-22]
8816	dipeptidyl peptidase IV inhibitor (DPP IV inhibitor)	dipeptidyl peptidase IV inhibitor	1	KT	[10-11]
8817	dipeptidyl peptidase IV inhibitor (DPP IV inhibitor)	dipeptidyl peptidase IV inhibitor	1	KV	[214-215]
8819	dipeptidyl peptidase IV inhibitor (DPP IV inhibitor)	dipeptidyl peptidase IV inhibitor	1	KY	[219-220]
8820	dipeptidyl peptidase IV inhibitor (DPP IV inhibitor)	dipeptidyl peptidase IV inhibitor	1	LH	[53-54]
8821	dipeptidyl peptidase IV inhibitor (DPP IV inhibitor)	dipeptidyl peptidase IV inhibitor	1	LI	[138-139]
8824	dipeptidyl peptidase IV inhibitor (DPP IV inhibitor)	dipeptidyl peptidase IV inhibitor	2	LT	[12-13],[109-110]
8825	dipeptidyl peptidase IV inhibitor (DPP IV inhibitor)	dipeptidyl peptidase IV inhibitor	3	LV	[166-167],[177-178],[184-185]
8826	dipeptidyl peptidase IV inhibitor (DPP IV inhibitor)	dipeptidyl peptidase IV inhibitor	1	ME	[135-136]
8834	dipeptidyl peptidase IV inhibitor (DPP IV inhibitor)	dipeptidyl peptidase IV inhibitor	1	MN	[142-143]
8835	dipeptidyl peptidase IV inhibitor (DPP IV inhibitor)	dipeptidyl peptidase IV inhibitor	1	MQ	[51-52]
8839	dipeptidyl peptidase IV inhibitor (DPP IV inhibitor)	dipeptidyl peptidase IV inhibitor	1	NA	[143-144]
8842	dipeptidyl peptidase IV inhibitor (DPP IV inhibitor)	dipeptidyl peptidase IV inhibitor	1	NF	[131-132]
8843	dipeptidyl peptidase IV inhibitor (DPP IV inhibitor)	dipeptidyl peptidase IV inhibitor	1	NG	[95-96]
8844	dipeptidyl peptidase IV inhibitor (DPP IV inhibitor)	dipeptidyl peptidase IV inhibitor	1	NH	[101-102]
8845	dipeptidyl peptidase IV inhibitor (DPP IV inhibitor)	dipeptidyl peptidase IV inhibitor	1	NL	[108-109]
8848	dipeptidyl peptidase IV inhibitor (DPP IV inhibitor)	dipeptidyl peptidase IV inhibitor	1	NQ	[173-174]
8851	dipeptidyl peptidase IV inhibitor (DPP IV inhibitor)	dipeptidyl peptidase IV inhibitor	1	NV	[203-204]

8852	dipeptidyl peptidase IV inhibitor (DPP IV inhibitor)	dipeptidyl peptidase IV inhibitor	1	NW	[217-218]
8853	dipeptidyl peptidase IV inhibitor (DPP IV inhibitor)	dipeptidyl peptidase IV inhibitor	1	NY	[65-66]
8856	dipeptidyl peptidase IV inhibitor (DPP IV inhibitor)	dipeptidyl peptidase IV inhibitor	1	PH	[120-121]
8862	dipeptidyl peptidase IV inhibitor (DPP IV inhibitor)	dipeptidyl peptidase IV inhibitor	1	PS	[182-183]
8864	dipeptidyl peptidase IV inhibitor (DPP IV inhibitor)	dipeptidyl peptidase IV inhibitor	1	PV	[111-112]
8866	dipeptidyl peptidase IV inhibitor (DPP IV inhibitor)	dipeptidyl peptidase IV inhibitor	1	PY	[36-37]
8867	dipeptidyl peptidase IV inhibitor (DPP IV inhibitor)	dipeptidyl peptidase IV inhibitor	1	QA	[75-76]
8868	dipeptidyl peptidase IV inhibitor (DPP IV inhibitor)	dipeptidyl peptidase IV inhibitor	1	QD	[174-175]
8871	dipeptidyl peptidase IV inhibitor (DPP IV inhibitor)	dipeptidyl peptidase IV inhibitor	1	QG	[150-151]
8874	dipeptidyl peptidase IV inhibitor (DPP IV inhibitor)	dipeptidyl peptidase IV inhibitor	2	QL	[52-53],[72-73]
8877	dipeptidyl peptidase IV inhibitor (DPP IV inhibitor)	dipeptidyl peptidase IV inhibitor	1	QS	[89-90]
8878	dipeptidyl peptidase IV inhibitor (DPP IV inhibitor)	dipeptidyl peptidase IV inhibitor	2	QT	[28-29],[60-61]
8881	dipeptidyl peptidase IV inhibitor (DPP IV inhibitor)	dipeptidyl peptidase IV inhibitor	1	QY	[200-201]
8882	dipeptidyl peptidase IV inhibitor (DPP IV inhibitor)	dipeptidyl peptidase IV inhibitor	1	RG	[25-26]
8885	dipeptidyl peptidase IV inhibitor (DPP IV inhibitor)	dipeptidyl peptidase IV inhibitor	1	RK	[9-10]
8886	dipeptidyl peptidase IV inhibitor (DPP IV inhibitor)	dipeptidyl peptidase IV inhibitor	1	RL	[17-18]
8887	dipeptidyl peptidase IV inhibitor (DPP IV inhibitor)	dipeptidyl peptidase IV inhibitor	1	RM	[141-142]
8892	dipeptidyl peptidase IV inhibitor (DPP IV inhibitor)	dipeptidyl peptidase IV inhibitor	1	SH	[22-23]
8893	dipeptidyl peptidase IV inhibitor (DPP IV inhibitor)	dipeptidyl peptidase IV inhibitor	1	SI	[103-104]
8894	dipeptidyl peptidase IV inhibitor (DPP IV inhibitor)	dipeptidyl peptidase IV inhibitor	1	SK	[163-164]
8897	dipeptidyl peptidase IV inhibitor (DPP IV inhibitor)	dipeptidyl peptidase IV inhibitor	1	SY	[5-6]
8899	dipeptidyl peptidase IV inhibitor (DPP IV inhibitor)	dipeptidyl peptidase IV inhibitor	1	TE	[13-14]
8900	dipeptidyl peptidase IV inhibitor (DPP IV inhibitor)	dipeptidyl peptidase IV inhibitor	1	TF	[29-30]
8904	dipeptidyl peptidase IV inhibitor (DPP IV inhibitor)	dipeptidyl peptidase IV inhibitor	1	TK	[179-180]
8905	dipeptidyl peptidase IV inhibitor (DPP IV inhibitor)	dipeptidyl peptidase IV inhibitor	2	TL	[11-12],[31-32]
8907	dipeptidyl peptidase IV inhibitor (DPP IV inhibitor)	dipeptidyl peptidase IV inhibitor	2	TN	[64-65],[130-131]
8909	dipeptidyl peptidase IV inhibitor (DPP IV inhibitor)	dipeptidyl peptidase IV inhibitor	1	TR	[8-9]
8911	dipeptidyl peptidase IV inhibitor (DPP IV inhibitor)	dipeptidyl peptidase IV inhibitor	1	TT	[170-171]
8914	dipeptidyl peptidase IV inhibitor (DPP IV inhibitor)	dipeptidyl peptidase IV inhibitor	1	TY	[61-62]
8916	dipeptidyl peptidase IV inhibitor (DPP IV inhibitor)	dipeptidyl peptidase IV inhibitor	1	VE	[168-169]
8920	dipeptidyl peptidase IV inhibitor (DPP IV inhibitor)	dipeptidyl peptidase IV inhibitor	1	VI	[215-216]
8921	dipeptidyl peptidase IV inhibitor (DPP IV inhibitor)	dipeptidyl peptidase IV inhibitor	1	VK	[86-87]

8926	dipeptidyl peptidase IV inhibitor (DPP IV inhibitor)	dipeptidyl peptidase IV inhibitor	1	VS	[112-113]
8927	dipeptidyl peptidase IV inhibitor (DPP IV inhibitor)	dipeptidyl peptidase IV inhibitor	2	VT	[7-8],[178-179]
8928	dipeptidyl peptidase IV inhibitor (DPP IV inhibitor)	dipeptidyl peptidase IV inhibitor	2	VW	[155-156],[192-193]
8932	dipeptidyl peptidase IV inhibitor (DPP IV inhibitor)	dipeptidyl peptidase IV inhibitor	1	YA	[220-221]
8934	dipeptidyl peptidase IV inhibitor (DPP IV inhibitor)	dipeptidyl peptidase IV inhibitor	1	YE	[37-38]
8936	dipeptidyl peptidase IV inhibitor (DPP IV inhibitor)	dipeptidyl peptidase IV inhibitor	1	YG	[39-40]
8938	dipeptidyl peptidase IV inhibitor (DPP IV inhibitor)	dipeptidyl peptidase IV inhibitor	1	YI	[62-63]
8939	dipeptidyl peptidase IV inhibitor (DPP IV inhibitor)	dipeptidyl peptidase IV inhibitor	1	YK	[201-202]
8940	dipeptidyl peptidase IV inhibitor (DPP IV inhibitor)	dipeptidyl peptidase IV inhibitor	2	YL	[198-199],[208-209]
8942	dipeptidyl peptidase IV inhibitor (DPP IV inhibitor)	dipeptidyl peptidase IV inhibitor	1	YN	[66-67]
8943	dipeptidyl peptidase IV inhibitor (DPP IV inhibitor)	dipeptidyl peptidase IV inhibitor	1	YQ	[225-226]
8946	dipeptidyl peptidase IV inhibitor (DPP IV inhibitor)	dipeptidyl peptidase IV inhibitor	1	YV	[6-7]
8948	dipeptidyl peptidase IV inhibitor (DPP IV inhibitor)	dipeptidyl peptidase IV inhibitor	1	YY	[197-198]
9476	DPP-III inhibitor	dipeptidyl peptidase III inhibitor	1	YY	[197-198]
9478	DPP-III inhibitor	dipeptidyl peptidase III inhibitor	1	LR	[16-17]
9482	DPP-III inhibitor	dipeptidyl peptidase III inhibitor	2	YL	[198-199],[208-209]
9483	DPP-III inhibitor	dipeptidyl peptidase III inhibitor	1	YK	[201-202]
9486	DPP-III inhibitor	dipeptidyl peptidase III inhibitor	1	TF	[29-30]
9487	DPP-III inhibitor	dipeptidyl peptidase III inhibitor	2	GE	[117-118],[222-223]
9492	DPP-III inhibitor	dipeptidyl peptidase III inhibitor	1	DA	[81-82]
9507	DPP-III inhibitor	dipeptidyl peptidase III inhibitor	1	SM	[134-135]
9508	DPP-III inhibitor	dipeptidyl peptidase III inhibitor	1	YG	[39-40]
9510	DPP-III inhibitor	dipeptidyl peptidase III inhibitor	1	YI	[62-63]
9511	DPP-III inhibitor	dipeptidyl peptidase III inhibitor	1	KA	[68-69]
8249	CaMPDE inhibitor	CaMPDE inhibitor	1	KF	[93-94]
2835	Renin inhibitor	renin inhibitor	1	FT	[30-31]
2842	Renin inhibitor	renin inhibitor	1	LR	[16-17]
8248	Renin inhibitor	renin inhibitor	1	KF	[93-94]
9433	Renin inhibitor	renin inhibitor	1	YA	[220-221]
9470	Renin inhibitor	renin inhibitor	1	LY	[224-225]
9471	Renin inhibitor	renin inhibitor	1	TF	[29-30]

Table S22. Profile of potential biological activity of fragments of protein Pis v 5.0101.

ID	Name of peptide	Activity	Number	Sequence	Location
3460	Prolyl endopeptidase inhibitor	antiamnestic	1	PG	[107-108]
3257	beta-lactokinin	ACE inhibitor	1	RL	[82-83]
3258	beta-lactokinin	ACE inhibitor	1	IR	[259-260]
3383	ACE inhibitor	ACE inhibitor	1	IY	[310-311]
3384	ACE inhibitor	ACE inhibitor	2	VF	[228-229],[378-379]
3386	ACE inhibitor	ACE inhibitor	1	KW	[330-331]
3388	ACE inhibitor	ACE inhibitor	2	MY	[93-94],[179-180]
3489	ACE inhibitor from sake lees	ACE inhibitor	3	RF	[127-128],[137-138],[405-406]
3492	ACE inhibitor from sake	ACE inhibitor	1	VY	[359-360]
3535	ACE inhibitor	ACE inhibitor	1	YPR	[180-182]
3537	ACE inhibitor	ACE inhibitor	2	PR	[181-182],[262-263]
3551	ACE inhibitor (from bovine beta-Lg)	ACE inhibitor	1	LF	[16-17]
3553	ACE inhibitor	ACE inhibitor	1	YG	[360-361]
3666	ACE inhibitor	ACE inhibitor	1	YP	[180-181]
7513	ACE inhibitor from Alaskan pollack skin	ACE inhibitor	1	PL	[421-422]
7541	ACE inhibitor from wheat germ hydrolysate	ACE inhibitor	1	IVY	[358-360]
7547	ACE inhibitor	ACE inhibitor	1	IRP	[259-261]
7558	ACE inhibitor from buckwheat	ACE inhibitor	4	VK	[236-237],[250-251],[252-253],[398-399]
7562	ACE inhibitor from soy hydrolysate	ACE inhibitor	1	IA	[144-145]
7581	ACE inhibitor	ACE inhibitor	1	IP	[106-107]
7582	ACE inhibitor	ACE inhibitor	1	RP	[260-261]
7583	ACE inhibitor	ACE inhibitor	1	AF	[440-441]
7584	ACE inhibitor	ACE inhibitor	2	AP	[89-90],[115-116]
7585	ACE inhibitor	ACE inhibitor	5	LA	[21-22],[186-187],[225-226],[422-423],[437-438]
7586	ACE inhibitor	ACE inhibitor	1	KR	[399-400]
7587	ACE inhibitor	ACE inhibitor	2	VP	[348-349],[391-392]
7588	ACE inhibitor	ACE inhibitor	3	RA	[400-401],[416-417],[430-431]
7591	ACE inhibitor	ACE inhibitor	2	GF	[219-220],[269-270]
7592	ACE inhibitor	ACE inhibitor	2	FR	[64-65],[138-139]
7593	ACE inhibitor	ACE inhibitor	2	IF	[216-217],[388-389]
7594	ACE inhibitor	ACE inhibitor	1	VG	[315-316]
7597	ACE inhibitor	ACE inhibitor	2	GM	[52-53],[100-101]
7598	ACE inhibitor	ACE inhibitor	1	GA	[247-248]

7599	ACE inhibitor	ACE inhibitor	2	GL	[80-81],[288-289]
7600	ACE inhibitor	ACE inhibitor	5	AG	[51-52],[67-68],[148-149],[187-188],[423-424]
7603	ACE inhibitor	ACE inhibitor	4	GR	[204-205],[281-282],[284-285],[316-317]
7604	ACE inhibitor	ACE inhibitor	3	KG	[140-141],[253-254],[363-364]
7605	ACE inhibitor	ACE inhibitor	1	FG	[374-375]
7606	ACE inhibitor	ACE inhibitor	3	DA	[39-40],[447-448],[472-473]
7607	ACE inhibitor	ACE inhibitor	1	GS	[424-425]
7608	ACE inhibitor	ACE inhibitor	3	GV	[68-69],[149-150],[339-340]
7610	ACE inhibitor	ACE inhibitor	6	GQ	[27-28],[119-120],[123-124],[386-387],[403-404],[463-464]
7612	ACE inhibitor	ACE inhibitor	1	GT	[103-104]
7614	ACE inhibitor	ACE inhibitor	2	HG	[18-19],[122-123]
7615	ACE inhibitor	ACE inhibitor	2	GE	[98-99],[381-382]
7617	ACE inhibitor	ACE inhibitor	2	QG	[26-27],[118-119]
7618	ACE inhibitor	ACE inhibitor	1	SG	[462-463]
7620	ACE inhibitor	ACE inhibitor	2	GD	[141-142],[254-255]
7621	ACE inhibitor	ACE inhibitor	1	TG	[102-103]
7622	ACE inhibitor	ACE inhibitor	4	EG	[97-98],[99-100],[157-158],[385-386]
7623	ACE inhibitor	ACE inhibitor	1	EA	[50-51]
7624	ACE inhibitor	ACE inhibitor	2	NG	[79-80],[287-288]
7625	ACE inhibitor	ACE inhibitor	1	PG	[107-108]
7635	ACE inhibitor from k-CN (fr. 51-53)	ACE inhibitor	1	VAV	[69-71]
7680	ACE inhibitor from pea vicilin	ACE inhibitor	2	QK	[133-134],[210-211]
7681	ACE inhibitor from soy	ACE inhibitor	1	DG	[380-381]
7683	ACE inhibitor from garlic	ACE inhibitor	3	NF	[352-353],[373-374],[394-395]
7685	ACE inhibitor from garlic	ACE inhibitor	1	SF	[410-411]
7692	ACE inhibitor	ACE inhibitor	2	KF	[183-184],[452-453]
7693	ACE inhibitor from wakame	ACE inhibitor	1	KL	[297-298]
7741	ACE inhibitor	ACE inhibitor	3	RR	[205-206],[263-264],[282-283]
7742	ACE inhibitor	ACE inhibitor	3	AR	[72-73],[401-402],[448-449]
7752	ACE inhibitor from shark meat hydrolysate	ACE inhibitor	3	EY	[48-49],[85-86],[277-278]
7828	ACE inhibitor	ACE inhibitor	4	EV	[227-228],[314-315],[382-383],[435-436]
7829	ACE inhibitor	ACE inhibitor	4	VE	[47-48],[54-55],[96-97],[231-232]
7831	ACE inhibitor	ACE inhibitor	4	LQ	[239-240],[256-257],[332-333],[341-342]
7832	ACE inhibitor	ACE inhibitor	2	LN	[321-322],[324-325]
7833	ACE inhibitor	ACE inhibitor	2	PT	[90-91],[458-459]
7835	ACE inhibitor	ACE inhibitor	1	AH	[355-356]

7836	ACE inhibitor	ACE inhibitor	1	PP	[261-262]
7837	ACE inhibitor	ACE inhibitor	2	PQ	[116-117],[392-393]
7838	ACE inhibitor	ACE inhibitor	1	EW	[407-408]
7840	ACE inhibitor	ACE inhibitor	1	EK	[242-243]
7841	ACE inhibitor	ACE inhibitor	1	KE	[299-300]
7843	ACE inhibitor	ACE inhibitor	1	PH	[349-350]
8182	ACE Inhibitor	ACE inhibitor	1	ALEP	[40-43]
8184	ACE Inhibitor	ACE inhibitor	1	IQP	[76-78]
8193	ACE inhibitor	ACE inhibitor	1	AI	[248-249]
8951	ACE inhibitor	ACE inhibitor	2	AV	[70-71],[396-397]
9029	ACE inhibitor	ACE inhibitor	1	ALP	[145-147]
9031	ACE inhibitor	ACE inhibitor	1	LEE	[289-291]
9046	ACE inhibitor	ACE inhibitor	1	VQV	[368-370]
9051	ACE inhibitor	ACE inhibitor	1	RPP	[260-262]
9073	ACE inhibitor	ACE inhibitor	1	TP	[312-313]
9075	ACE inhibitor	ACE inhibitor	1	DM	[178-179]
9076	ACE inhibitor	ACE inhibitor	4	FQ	[128-129],[194-195],[229-230],[441-442]
9078	ACE inhibitor	ACE inhibitor	2	YE	[49-50],[278-279]
9079	ACE inhibitor	ACE inhibitor	3	IL	[15-16],[224-225],[328-329]
9107	ACE inhibitor	ACE inhibitor	1	WL	[331-332]
9160	ACE inhibitor	ACE inhibitor	1	TLS	[459-461]
9173	ACE inhibitor	ACE inhibitor	7	RG	[203-204],[246-247],[268-269],[280-281],[283-284],[338-339],[402-403]
9184	ACE inhibitor	ACE inhibitor	1	ST	[425-426]
9185	ACE inhibitor	ACE inhibitor	1	YN	[155-156]
9192	ACE inhibitor	ACE inhibitor	1	AGS	[423-425]
9196	ACE inhibitor	ACE inhibitor	1	AVV	[396-398]
9213	ACE inhibitor	ACE inhibitor	2	LR	[81-82],[429-430]
9265	ACE inhibitor	ACE inhibitor	1	TLS	[459-461]
9566	ACE inhibitor	ACE inhibitor	2	QP	[77-78],[457-458]
3285	Antithrombotic peptide	antithrombotic	1	PG	[107-108]
2882	Immunostimulating peptide	immunomodulating	1	YG	[360-361]
3351	Stimulating vasoactive substance release	stimulating	1	EEE	[273-275]
8320	Glucose uptake stimulating peptide	stimulating	3	VL	[340-341],[428-429],[436-437]
8321	Glucose uptake stimulating peptide	stimulating	1	LV	[235-236]
8322	Glucose uptake stimulating peptide	stimulating	3	IV	[95-96],[249-250],[358-359]
8323	Glucose uptake stimulating peptide	stimulating	3	IL	[15-16],[224-225],[328-329]

8324	Glucose uptake stimulating peptide	stimulating	2	LI	[14-15],[105-106]
8325	Glucose uptake stimulating peptide	stimulating	1	II	[143-144]
8326	Glucose uptake stimulating peptide	stimulating	3	LL	[6-7],[13-14],[167-168]
8329	Stimulating vasoactive substance release	stimulating	4	EE	[273-274],[274-275],[290-291],[434-435]
8330	Stimulating vasoactive substance release	stimulating	4	SE	[266-267],[272-273],[276-277],[384-385]
2890	neuropeptide	neuropeptide	6	GQ	[27-28],[119-120],[123-124],[386-387],[403-404],[463-464]
2754	peptide regulating the stomach mucosal membrane activity	regulating	1	PG	[107-108]
7873	peptide from soybean protein isolates: beta-conglycinin and glycinin	antioxidative	1	IY	[310-311]
7886	peptide derived from egg white albumin	antioxidative	1	AH	[355-356]
8037	synthetic peptide	antioxidative	1	PHW	[349-351]
8067	synthetic peptide	antioxidative	1	RHT	[73-75]
8090	peptide derived from sardine muscle	antioxidative	2	MY	[93-94],[179-180]
8105	peptide derived from dried bonito	antioxidative	1	VKV	[250-252]
8134	peptide derived from dried bonito	antioxidative	1	KD	[243-244]
8215	Antioxidative peptide	antioxidative	1	IR	[259-260]
8217	Antioxidative peptide	antioxidative	2	LK	[298-299],[329-330]
8219	antioxidative peptide	antioxidative	1	TY	[112-113]
8224	antioxidative peptide	antioxidative	1	VY	[359-360]
8459	Antioxidant peptide from marine bivalve (Mactra veneriformis)	antioxidative	1	TW	[56-57]
9342	Antioxidative peptide	antioxidative	1	FC	[217-218]
4005		activating ubiquitin-mediated proteolysis	3	RA	[400-401],[416-417],[430-431]
4006	Ubiqitin-mediated proteolysis activating peptide	activating ubiquitin-mediated proteolysis	5	LA	[21-22],[186-187],[225-226],[422-423],[437-438]
9580	Hypolipidemic peptide	hypolipidemic	1	EF	[193-194]
9548	Alpha-glucosidase inhibitor	alpha-glucosidase inhibitor	1	YP	[180-181]
9650	Alpha-glucosidase inhibitor	alpha-glucosidase inhibitor	1	EA	[50-51]
9651	Alpha-glucosidase inhibitor	alpha-glucosidase inhibitor	1	PP	[261-262]
9693	Alpha-glucosidase inhibitor	alpha-glucosidase inhibitor	4	VE	[47-48],[54-55],[96-97],[231-232]
9694	Alpha-glucosidase inhibitor	alpha-glucosidase inhibitor	5	PE	[84-85],[110-111],[190-191],[313-314],[433-434]
3170	dipeptidyl peptidase IV inhibitor (DPP IV inhibitor)	dipeptidyl peptidase IV inhibitor	1	PP	[261-262]
3171	dipeptidyl peptidase IV inhibitor (DPP IV inhibitor)	dipeptidyl peptidase IV inhibitor	1	MP	[432-433]
3172	dipeptidyl peptidase IV inhibitor (DPP IV inhibitor)	dipeptidyl peptidase IV inhibitor	3	VA	[69-70],[71-72],[150-151]
3173	dipeptidyl peptidase IV inhibitor (DPP IV inhibitor)	dipeptidyl peptidase IV inhibitor	1	MA	[1-2]

3175	dipeptidyl peptidase IV inhibitor (DPP IV inhibitor)	dipeptidyl peptidase IV inhibitor	5	LA	[21-22],[186-187],[225-226],[422-423],[437-438]
3176	dipeptidyl peptidase IV inhibitor (DPP IV inhibitor)	dipeptidyl peptidase IV inhibitor	1	FA	[395-396]
3177	dipeptidyl peptidase IV inhibitor (DPP IV inhibitor)	dipeptidyl peptidase IV inhibitor	2	AP	[89-90],[115-116]
3179	dipeptidyl peptidase IV inhibitor (DPP IV inhibitor)	dipeptidyl peptidase IV inhibitor	1	PA	[147-148]
3180	dipeptidyl peptidase IV inhibitor (DPP IV inhibitor)	dipeptidyl peptidase IV inhibitor	3	LP	[83-84],[146-147],[326-327]
3181	dipeptidyl peptidase IV inhibitor (DPP IV inhibitor)	dipeptidyl peptidase IV inhibitor	2	VP	[348-349],[391-392]
3182	dipeptidyl peptidase IV inhibitor (DPP IV inhibitor)	dipeptidyl peptidase IV inhibitor	3	LL	[6-7],[13-14],[167-168]
3183	dipeptidyl peptidase IV inhibitor (DPP IV inhibitor)	dipeptidyl peptidase IV inhibitor	4	VV	[162-163],[370-371],[390-391],[397-398]
8501	Dipeptidyl peptidase IV inhibitor (DPP IV inhibitor)	dipeptidyl peptidase IV inhibitor	1	IP	[106-107]
8503	Dipeptidyl peptidase IV inhibitor (DPP IV inhibitor)	dipeptidyl peptidase IV inhibitor	1	TP	[312-313]
8505	Dipeptidyl peptidase IV inhibitor (DPP IV inhibitor)	dipeptidyl peptidase IV inhibitor	2	SP	[160-161],[420-421]
8518	dipeptidyl peptidase IV inhibitor (DPP IV inhibitor)	dipeptidyl peptidase IV inhibitor	1	RP	[260-261]
8521	dipeptidyl peptidase IV inhibitor (DPP IV inhibitor)	dipeptidyl peptidase IV inhibitor	1	YP	[180-181]
8524	dipeptidyl peptidase IV inhibitor (DPP IV inhibitor)	dipeptidyl peptidase IV inhibitor	1	GA	[247-248]
8525	dipeptidyl peptidase IV inhibitor (DPP IV inhibitor)	dipeptidyl peptidase IV inhibitor	1	IA	[144-145]
8526	dipeptidyl peptidase IV inhibitor (DPP IV inhibitor)	dipeptidyl peptidase IV inhibitor	3	RA	[400-401],[416-417],[430-431]
8529	dipeptidyl peptidase IV inhibitor (DPP IV inhibitor)	dipeptidyl peptidase IV inhibitor	1	EP	[42-43]
8530	dipeptidyl peptidase IV inhibitor (DPP IV inhibitor)	dipeptidyl peptidase IV inhibitor	2	NP	[3-4],[189-190]
8532	dipeptidyl peptidase IV inhibitor (DPP IV inhibitor)	dipeptidyl peptidase IV inhibitor	2	QP	[77-78],[457-458]
8558	dipeptidyl peptidase IV inhibitor (DPP IV inhibitor)	dipeptidyl peptidase IV inhibitor	1	EK	[242-243]
8559	dipeptidyl peptidase IV inhibitor (DPP IV inhibitor)	dipeptidyl peptidase IV inhibitor	3	AL	[40-41],[145-146],[345-346]
8560	dipeptidyl peptidase IV inhibitor (DPP IV inhibitor)	dipeptidyl peptidase IV inhibitor	5	SL	[5-6],[8-9],[234-235],[320-321],[323-324]
8561	dipeptidyl peptidase IV inhibitor (DPP IV inhibitor)	dipeptidyl peptidase IV inhibitor	2	GL	[80-81],[288-289]
8638	dipeptidyl peptidase IV inhibitor (DPP IV inhibitor)	dipeptidyl peptidase IV inhibitor	1	PL	[421-422]
8677	dipeptidyl peptidase IV inhibitor (DPP IV inhibitor)	dipeptidyl peptidase IV inhibitor	1	WL	[331-332]
8679	dipeptidyl peptidase IV inhibitor (DPP IV inhibitor)	dipeptidyl peptidase IV inhibitor	1	WI	[408-409]
8680	dipeptidyl peptidase IV inhibitor (DPP IV inhibitor)	dipeptidyl peptidase IV inhibitor	1	WN	[351-352]
8684	dipeptidyl peptidase IV inhibitor (DPP IV inhibitor)	dipeptidyl peptidase IV inhibitor	1	WC	[153-154]
8693	dipeptidyl peptidase IV inhibitor (DPP IV inhibitor)	dipeptidyl peptidase IV inhibitor	1	IQP	[76-78]
8696	dipeptidyl peptidase IV inhibitor (DPP IV inhibitor)	dipeptidyl peptidase IV inhibitor	1	YT	[311-312]
8758	dipeptidyl peptidase IV inhibitor (DPP IV inhibitor)	dipeptidyl peptidase IV inhibitor	2	AE	[226-227],[336-337]
8759	dipeptidyl peptidase IV inhibitor (DPP IV inhibitor)	dipeptidyl peptidase IV inhibitor	1	AF	[440-441]
8760	dipeptidyl peptidase IV inhibitor (DPP IV inhibitor)	dipeptidyl peptidase IV inhibitor	5	AG	[51-52],[67-68],[148-149],[187-188],[423-424]
8761	dipeptidyl peptidase IV inhibitor (DPP IV inhibitor)	dipeptidyl peptidase IV inhibitor	1	AH	[355-356]
8762	dipeptidyl peptidase IV inhibitor (DPP IV inhibitor)	dipeptidyl peptidase IV inhibitor	1	AS	[22-23]
8764	dipeptidyl peptidase IV inhibitor (DPP IV inhibitor)	dipeptidyl peptidase IV inhibitor	2	AV	[70-71],[396-397]

8766	dipeptidyl peptidase IV inhibitor (DPP IV inhibitor)	dipeptidyl peptidase IV inhibitor	4	DN	[44-45],[244-245],[286-287],[372-373]
8767	dipeptidyl peptidase IV inhibitor (DPP IV inhibitor)	dipeptidyl peptidase IV inhibitor	2	DP	[58-59],[304-305]
8768	dipeptidyl peptidase IV inhibitor (DPP IV inhibitor)	dipeptidyl peptidase IV inhibitor	1	DQ	[36-37]
8769	dipeptidyl peptidase IV inhibitor (DPP IV inhibitor)	dipeptidyl peptidase IV inhibitor	1	DR	[415-416]
8770	dipeptidyl peptidase IV inhibitor (DPP IV inhibitor)	dipeptidyl peptidase IV inhibitor	4	EG	[97-98],[99-100],[157-158],[385-386]
8773	dipeptidyl peptidase IV inhibitor (DPP IV inhibitor)	dipeptidyl peptidase IV inhibitor	2	ES	[271-272],[275-276]
8774	dipeptidyl peptidase IV inhibitor (DPP IV inhibitor)	dipeptidyl peptidase IV inhibitor	3	ET	[55-56],[111-112],[291-292]
8775	dipeptidyl peptidase IV inhibitor (DPP IV inhibitor)	dipeptidyl peptidase IV inhibitor	4	EV	[227-228],[314-315],[382-383],[435-436]
8776	dipeptidyl peptidase IV inhibitor (DPP IV inhibitor)	dipeptidyl peptidase IV inhibitor	1	EW	[407-408]
8777	dipeptidyl peptidase IV inhibitor (DPP IV inhibitor)	dipeptidyl peptidase IV inhibitor	3	EY	[48-49],[85-86],[277-278]
8778	dipeptidyl peptidase IV inhibitor (DPP IV inhibitor)	dipeptidyl peptidase IV inhibitor	3	FN	[184-185],[353-354],[453-454]
8779	dipeptidyl peptidase IV inhibitor (DPP IV inhibitor)	dipeptidyl peptidase IV inhibitor	4	FQ	[128-129],[194-195],[229-230],[441-442]
8780	dipeptidyl peptidase IV inhibitor (DPP IV inhibitor)	dipeptidyl peptidase IV inhibitor	2	FR	[64-65],[138-139]
8781	dipeptidyl peptidase IV inhibitor (DPP IV inhibitor)	dipeptidyl peptidase IV inhibitor	2	GE	[98-99],[381-382]
8782	dipeptidyl peptidase IV inhibitor (DPP IV inhibitor)	dipeptidyl peptidase IV inhibitor	2	GF	[219-220],[269-270]
8786	dipeptidyl peptidase IV inhibitor (DPP IV inhibitor)	dipeptidyl peptidase IV inhibitor	3	GV	[68-69],[149-150],[339-340]
8789	dipeptidyl peptidase IV inhibitor (DPP IV inhibitor)	dipeptidyl peptidase IV inhibitor	1	HD	[303-304]
8790	dipeptidyl peptidase IV inhibitor (DPP IV inhibitor)	dipeptidyl peptidase IV inhibitor	1	HE	[61-62]
8795	dipeptidyl peptidase IV inhibitor (DPP IV inhibitor)	dipeptidyl peptidase IV inhibitor	1	HS	[356-357]
8796	dipeptidyl peptidase IV inhibitor (DPP IV inhibitor)	dipeptidyl peptidase IV inhibitor	1	HT	[74-75]
8798	dipeptidyl peptidase IV inhibitor (DPP IV inhibitor)	dipeptidyl peptidase IV inhibitor	1	HW	[350-351]
8800	dipeptidyl peptidase IV inhibitor (DPP IV inhibitor)	dipeptidyl peptidase IV inhibitor	1	IH	[302-303]
8801	dipeptidyl peptidase IV inhibitor (DPP IV inhibitor)	dipeptidyl peptidase IV inhibitor	1	II	[143-144]
8802	dipeptidyl peptidase IV inhibitor (DPP IV inhibitor)	dipeptidyl peptidase IV inhibitor	3	IL	[15-16],[224-225],[328-329]
8805	dipeptidyl peptidase IV inhibitor (DPP IV inhibitor)	dipeptidyl peptidase IV inhibitor	2	IQ	[76-77],[135-136]
8806	dipeptidyl peptidase IV inhibitor (DPP IV inhibitor)	dipeptidyl peptidase IV inhibitor	1	IR	[259-260]
8808	dipeptidyl peptidase IV inhibitor (DPP IV inhibitor)	dipeptidyl peptidase IV inhibitor	1	KE	[299-300]
8809	dipeptidyl peptidase IV inhibitor (DPP IV inhibitor)	dipeptidyl peptidase IV inhibitor	2	KF	[183-184],[452-453]
8810	dipeptidyl peptidase IV inhibitor (DPP IV inhibitor)	dipeptidyl peptidase IV inhibitor	3	KG	[140-141],[253-254],[363-364]
8811	dipeptidyl peptidase IV inhibitor (DPP IV inhibitor)	dipeptidyl peptidase IV inhibitor	1	KH	[131-132]
8812	dipeptidyl peptidase IV inhibitor (DPP IV inhibitor)	dipeptidyl peptidase IV inhibitor	3	KI	[134-135],[223-224],[450-451]
8814	dipeptidyl peptidase IV inhibitor (DPP IV inhibitor)	dipeptidyl peptidase IV inhibitor	1	KR	[399-400]
8815	dipeptidyl peptidase IV inhibitor (DPP IV inhibitor)	dipeptidyl peptidase IV inhibitor	1	KS	[211-212]
8816	dipeptidyl peptidase IV inhibitor (DPP IV inhibitor)	dipeptidyl peptidase IV inhibitor	1	KT	[412-413]
8817	dipeptidyl peptidase IV inhibitor (DPP IV inhibitor)	dipeptidyl peptidase IV inhibitor	1	KV	[251-252]
8818	dipeptidyl peptidase IV inhibitor (DPP IV inhibitor)	dipeptidyl peptidase IV inhibitor	1	KW	[330-331]

8821	dipeptidyl peptidase IV inhibitor (DPP IV inhibitor)	dipeptidyl peptidase IV inhibitor	2	LI	[14-15],[105-106]
8822	dipeptidyl peptidase IV inhibitor (DPP IV inhibitor)	dipeptidyl peptidase IV inhibitor	2	LM	[92-93],[346-347]
8823	dipeptidyl peptidase IV inhibitor (DPP IV inhibitor)	dipeptidyl peptidase IV inhibitor	2	LN	[321-322],[324-325]
8825	dipeptidyl peptidase IV inhibitor (DPP IV inhibitor)	dipeptidyl peptidase IV inhibitor	1	LV	[235-236]
8830	dipeptidyl peptidase IV inhibitor (DPP IV inhibitor)	dipeptidyl peptidase IV inhibitor	1	MI	[418-419]
8831	dipeptidyl peptidase IV inhibitor (DPP IV inhibitor)	dipeptidyl peptidase IV inhibitor	1	MK	[296-297]
8836	dipeptidyl peptidase IV inhibitor (DPP IV inhibitor)	dipeptidyl peptidase IV inhibitor	1	MR	[469-470]
8837	dipeptidyl peptidase IV inhibitor (DPP IV inhibitor)	dipeptidyl peptidase IV inhibitor	2	MV	[53-54],[347-348]
8838	dipeptidyl peptidase IV inhibitor (DPP IV inhibitor)	dipeptidyl peptidase IV inhibitor	2	MY	[93-94],[179-180]
8839	dipeptidyl peptidase IV inhibitor (DPP IV inhibitor)	dipeptidyl peptidase IV inhibitor	5	NA	[88-89],[344-345],[354-355],[365-366],[439-440]
8840	dipeptidyl peptidase IV inhibitor (DPP IV inhibitor)	dipeptidyl peptidase IV inhibitor	1	ND	[414-415]
8841	dipeptidyl peptidase IV inhibitor (DPP IV inhibitor)	dipeptidyl peptidase IV inhibitor	4	NE	[31-32],[156-157],[241-242],[455-456]
8842	dipeptidyl peptidase IV inhibitor (DPP IV inhibitor)	dipeptidyl peptidase IV inhibitor	3	NF	[352-353],[373-374],[394-395]
8843	dipeptidyl peptidase IV inhibitor (DPP IV inhibitor)	dipeptidyl peptidase IV inhibitor	2	NG	[79-80],[287-288]
8844	dipeptidyl peptidase IV inhibitor (DPP IV inhibitor)	dipeptidyl peptidase IV inhibitor	1	NH	[60-61]
8845	dipeptidyl peptidase IV inhibitor (DPP IV inhibitor)	dipeptidyl peptidase IV inhibitor	2	NL	[185-186],[325-326]
8847	dipeptidyl peptidase IV inhibitor (DPP IV inhibitor)	dipeptidyl peptidase IV inhibitor	3	NN	[214-215],[343-344],[454-455]
8848	dipeptidyl peptidase IV inhibitor (DPP IV inhibitor)	dipeptidyl peptidase IV inhibitor	1	NQ	[175-176]
8849	dipeptidyl peptidase IV inhibitor (DPP IV inhibitor)	dipeptidyl peptidase IV inhibitor	2	NR	[45-46],[245-246]
8850	dipeptidyl peptidase IV inhibitor (DPP IV inhibitor)	dipeptidyl peptidase IV inhibitor	1	NT	[376-377]
8852	dipeptidyl peptidase IV inhibitor (DPP IV inhibitor)	dipeptidyl peptidase IV inhibitor	1	NW	[152-153]
8855	dipeptidyl peptidase IV inhibitor (DPP IV inhibitor)	dipeptidyl peptidase IV inhibitor	1	PG	[107-108]
8856	dipeptidyl peptidase IV inhibitor (DPP IV inhibitor)	dipeptidyl peptidase IV inhibitor	1	PH	[349-350]
8857	dipeptidyl peptidase IV inhibitor (DPP IV inhibitor)	dipeptidyl peptidase IV inhibitor	1	PI	[327-328]
8860	dipeptidyl peptidase IV inhibitor (DPP IV inhibitor)	dipeptidyl peptidase IV inhibitor	2	PN	[59-60],[78-79]
8861	dipeptidyl peptidase IV inhibitor (DPP IV inhibitor)	dipeptidyl peptidase IV inhibitor	2	PQ	[116-117],[392-393]
8862	dipeptidyl peptidase IV inhibitor (DPP IV inhibitor)	dipeptidyl peptidase IV inhibitor	2	PS	[4-5],[305-306]
8863	dipeptidyl peptidase IV inhibitor (DPP IV inhibitor)	dipeptidyl peptidase IV inhibitor	2	PT	[90-91],[458-459]
8864	dipeptidyl peptidase IV inhibitor (DPP IV inhibitor)	dipeptidyl peptidase IV inhibitor	1	PV	[161-162]
8867	dipeptidyl peptidase IV inhibitor (DPP IV inhibitor)	dipeptidyl peptidase IV inhibitor	1	QA	[114-115]
8868	dipeptidyl peptidase IV inhibitor (DPP IV inhibitor)	dipeptidyl peptidase IV inhibitor	1	QD	[129-130]
8870	dipeptidyl peptidase IV inhibitor (DPP IV inhibitor)	dipeptidyl peptidase IV inhibitor	1	QF	[63-64]
8871	dipeptidyl peptidase IV inhibitor (DPP IV inhibitor)	dipeptidyl peptidase IV inhibitor	2	QG	[26-27],[118-119]
8872	dipeptidyl peptidase IV inhibitor (DPP IV inhibitor)	dipeptidyl peptidase IV inhibitor	1	QH	[121-122]
8873	dipeptidyl peptidase IV inhibitor (DPP IV inhibitor)	dipeptidyl peptidase IV inhibitor	3	QI	[34-35],[387-388],[442-443]
8874	dipeptidyl peptidase IV inhibitor (DPP IV inhibitor)	dipeptidyl peptidase IV inhibitor	4	QL	[37-38],[176-177],[238-239],[333-334]

8875	dipeptidyl peptidase IV inhibitor (DPP IV inhibitor)	dipeptidyl peptidase IV inhibitor	5	QN	[30-31],[174-175],[240-241],[342-343],[393-394]
8876	dipeptidyl peptidase IV inhibitor (DPP IV inhibitor)	dipeptidyl peptidase IV inhibitor	13	QQ	[25-26],[28-29],[29-30],[117-118],[120-121],[195-196],[196-197],[197-198],[198-199],[199-200],[200-201],[209-210],[467-468]
8877	dipeptidyl peptidase IV inhibitor (DPP IV inhibitor)	dipeptidyl peptidase IV inhibitor	6	QS	[124-125],[201-202],[207-208],[233-234],[265-266],[464-465]
8879	dipeptidyl peptidase IV inhibitor (DPP IV inhibitor)	dipeptidyl peptidase IV inhibitor	4	QV	[230-231],[257-258],[367-368],[369-370]
8882	dipeptidyl peptidase IV inhibitor (DPP IV inhibitor)	dipeptidyl peptidase IV inhibitor	7	RG	[203-204],[246-247],[268-269],[280-281],[283-284],[338-339],[402-403]
8883	dipeptidyl peptidase IV inhibitor (DPP IV inhibitor)	dipeptidyl peptidase IV inhibitor	1	RH	[73-74]
8884	dipeptidyl peptidase IV inhibitor (DPP IV inhibitor)	dipeptidyl peptidase IV inhibitor	1	RI	[317-318]
8885	dipeptidyl peptidase IV inhibitor (DPP IV inhibitor)	dipeptidyl peptidase IV inhibitor	3	RK	[139-140],[182-183],[449-450]
8886	dipeptidyl peptidase IV inhibitor (DPP IV inhibitor)	dipeptidyl peptidase IV inhibitor	1	RL	[82-83]
8889	dipeptidyl peptidase IV inhibitor (DPP IV inhibitor)	dipeptidyl peptidase IV inhibitor	3	RR	[205-206],[263-264],[282-283]
8891	dipeptidyl peptidase IV inhibitor (DPP IV inhibitor)	dipeptidyl peptidase IV inhibitor	1	SF	[410-411]
8893	dipeptidyl peptidase IV inhibitor (DPP IV inhibitor)	dipeptidyl peptidase IV inhibitor	1	SI	[357-358]
8895	dipeptidyl peptidase IV inhibitor (DPP IV inhibitor)	dipeptidyl peptidase IV inhibitor	2	SV	[10-11],[427-428]
8901	dipeptidyl peptidase IV inhibitor (DPP IV inhibitor)	dipeptidyl peptidase IV inhibitor	1	TG	[102-103]
8903	dipeptidyl peptidase IV inhibitor (DPP IV inhibitor)	dipeptidyl peptidase IV inhibitor	2	TI	[75-76],[292-293]
8904	dipeptidyl peptidase IV inhibitor (DPP IV inhibitor)	dipeptidyl peptidase IV inhibitor	1	TK	[222-223]
8905	dipeptidyl peptidase IV inhibitor (DPP IV inhibitor)	dipeptidyl peptidase IV inhibitor	4	TL	[91-92],[104-105],[166-167],[459-460]
8906	dipeptidyl peptidase IV inhibitor (DPP IV inhibitor)	dipeptidyl peptidase IV inhibitor	1	TM	[295-296]
8907	dipeptidyl peptidase IV inhibitor (DPP IV inhibitor)	dipeptidyl peptidase IV inhibitor	1	TN	[413-414]
8910	dipeptidyl peptidase IV inhibitor (DPP IV inhibitor)	dipeptidyl peptidase IV inhibitor	2	TS	[319-320],[426-427]
8912	dipeptidyl peptidase IV inhibitor (DPP IV inhibitor)	dipeptidyl peptidase IV inhibitor	2	TV	[164-165],[377-378]
8913	dipeptidyl peptidase IV inhibitor (DPP IV inhibitor)	dipeptidyl peptidase IV inhibitor	1	TW	[56-57]
8914	dipeptidyl peptidase IV inhibitor (DPP IV inhibitor)	dipeptidyl peptidase IV inhibitor	1	TY	[112-113]
8915	dipeptidyl peptidase IV inhibitor (DPP IV inhibitor)	dipeptidyl peptidase IV inhibitor	1	VD	[371-372]
8916	dipeptidyl peptidase IV inhibitor (DPP IV inhibitor)	dipeptidyl peptidase IV inhibitor	4	VE	[47-48],[54-55],[96-97],[231-232]
8917	dipeptidyl peptidase IV inhibitor (DPP IV inhibitor)	dipeptidyl peptidase IV inhibitor	2	VF	[228-229],[378-379]
8918	dipeptidyl peptidase IV inhibitor (DPP IV inhibitor)	dipeptidyl peptidase IV inhibitor	1	VG	[315-316]
8920	dipeptidyl peptidase IV inhibitor (DPP IV inhibitor)	dipeptidyl peptidase IV inhibitor	1	VI	[258-259]
8921	dipeptidyl peptidase IV inhibitor (DPP IV inhibitor)	dipeptidyl peptidase IV inhibitor	4	VK	[236-237],[250-251],[252-253],[398-399]
8922	dipeptidyl peptidase IV inhibitor (DPP IV inhibitor)	dipeptidyl peptidase IV inhibitor	3	VL	[340-341],[428-429],[436-437]
8925	dipeptidyl peptidase IV inhibitor (DPP IV inhibitor)	dipeptidyl peptidase IV inhibitor	1	VQ	[368-369]
8926	dipeptidyl peptidase IV inhibitor (DPP IV inhibitor)	dipeptidyl peptidase IV inhibitor	2	VS	[170-171],[383-384]
8927	dipeptidyl peptidase IV inhibitor (DPP IV inhibitor)	dipeptidyl peptidase IV inhibitor	2	VT	[163-164],[165-166]
8929	dipeptidyl peptidase IV inhibitor (DPP IV inhibitor)	dipeptidyl peptidase IV inhibitor	1	VY	[359-360]
8930	dipeptidyl peptidase IV inhibitor (DPP IV inhibitor)	dipeptidyl peptidase IV inhibitor	1	WD	[57-58]

8934	dipeptidyl peptidase IV inhibitor (DPP IV inhibitor)	dipeptidyl peptidase IV inhibitor	2	YE	[49-50],[278-279]
8936	dipeptidyl peptidase IV inhibitor (DPP IV inhibitor)	dipeptidyl peptidase IV inhibitor	1	YG	[360-361]
8938	dipeptidyl peptidase IV inhibitor (DPP IV inhibitor)	dipeptidyl peptidase IV inhibitor	1	YI	[94-95]
8942	dipeptidyl peptidase IV inhibitor (DPP IV inhibitor)	dipeptidyl peptidase IV inhibitor	1	YN	[155-156]
8943	dipeptidyl peptidase IV inhibitor (DPP IV inhibitor)	dipeptidyl peptidase IV inhibitor	1	YQ	[113-114]
8945	dipeptidyl peptidase IV inhibitor (DPP IV inhibitor)	dipeptidyl peptidase IV inhibitor	1	YS	[86-87]
9478	DPP-III inhibitor	dipeptidyl peptidase III inhibitor	2	LR	[81-82],[429-430]
9479	DPP-III inhibitor	dipeptidyl peptidase III inhibitor	1	MR	[469-470]
9485	DPP-III inhibitor	dipeptidyl peptidase III inhibitor	3	RR	[205-206],[263-264],[282-283]
9487	DPP-III inhibitor	dipeptidyl peptidase III inhibitor	2	GE	[98-99],[381-382]
9488	DPP-III inhibitor	dipeptidyl peptidase III inhibitor	2	GF	[219-220],[269-270]
9489	DPP-III inhibitor	dipeptidyl peptidase III inhibitor	2	PR	[181-182],[262-263]
9490	DPP-III inhibitor	dipeptidyl peptidase III inhibitor	3	RF	[127-128],[137-138],[405-406]
9491	DPP-III inhibitor	dipeptidyl peptidase III inhibitor	1	RV	[46-47]
9492	DPP-III inhibitor	dipeptidyl peptidase III inhibitor	3	DA	[39-40],[447-448],[472-473]
9497	DPP-III inhibitor	dipeptidyl peptidase III inhibitor	1	IH	[302-303]
9499	DPP-III inhibitor	dipeptidyl peptidase III inhibitor	5	LA	[21-22],[186-187],[225-226],[422-423],[437-438]
9500	DPP-III inhibitor	dipeptidyl peptidase III inhibitor	1	FA	[395-396]
9501	DPP-III inhibitor	dipeptidyl peptidase III inhibitor	2	FR	[64-65],[138-139]
9504	DPP-III inhibitor	dipeptidyl peptidase III inhibitor	5	PE	[84-85],[110-111],[190-191],[313-314],[433-434]
9508	DPP-III inhibitor	dipeptidyl peptidase III inhibitor	1	YG	[360-361]
9509	DPP-III inhibitor	dipeptidyl peptidase III inhibitor	1	VY	[359-360]
9510	DPP-III inhibitor	dipeptidyl peptidase III inhibitor	1	YI	[94-95]
8247	CaMPDE inhibitor	CaMPDE inhibitor	1	IR	[259-260]
8249	CaMPDE inhibitor	CaMPDE inhibitor	2	KF	[183-184],[452-453]
8250	CaMPDE inhibitor	CaMPDE inhibitor	1	EF	[193-194]
2842	Renin inhibitor	renin inhibitor	2	LR	[81-82],[429-430]
8246	renin inhibitor	renin inhibitor	1	IR	[259-260]
8248	Renin inhibitor	renin inhibitor	2	KF	[183-184],[452-453]
8251	Renin inhibitor	renin inhibitor	1	EF	[193-194]
9430	Renin inhibitor	renin inhibitor	2	NR	[45-46],[245-246]
9431	Renin inhibitor	renin inhibitor	1	QF	[63-64]
9432	Renin inhibitor	renin inhibitor	1	SF	[410-411]

Table S23. Predicted products of proteolysis of the proteins from tree nuts by pepsin, trypsin and chymotrypsin.

No	Allergen	Products of proteolysis
1	Ana o 1.0101	M - G - PPT - K - F - SF - SL - F - L - VS - VL - VL - CL - G - F - A - L - A - K - ID - PE - L - K - Q - CK - H - Q - CK - VQ - R - Q - Y - D - E - Q - Q - K - E - Q - C - VK - E - CE - K - Y - Y - K - E - K - K - G - R - E - R - E - H - E - E - E - E - E - E - W - G - T - G - G - VD - E - PST - H - E - PA - E - K - H - L - SQ - CM - R - Q - CE - R - Q - E - G - G - Q - Q - K - Q - L - CR - F - R - CQ - E - R - Y - K - K - E - R - G - Q - H - N - Y - K - R - E - D - D - E - D - E - D - E - D - E - A - E - E - E - D - E - N - PY - VF - E - D - E - D - F - T - T - K - VK - T - E - Q - G - K - V - VL - L - PK - F - T - Q - K - SK - L - L - H - A - L - E - K - Y - R - L - A - VL - VA - N - PQ - A - F - V - VPSH - M - D - A - D - S - IF - F - VSW - G - R - G - T - IT - K - IL - E - N - K - R - E - S - IN - VR - Q - G - D - I - VS - ISSG - T - PF - Y - IA - N - N - D - E - N - E - K - L - Y - L - VQ - F - L - R - P - VN - L - PG - H - F - E - VF - H - G - PG - G - E - N - PE - SF - Y - R - A - F - SW - E - IL - E - A - A - L - K - T - SK - D - T - L - E - K - L - F - E - K - Q - D - Q - G - T - IM - K - A - SK - E - Q - IR - A - M - SR - R - G - E - G - PK - IW - PF - T - E - E - ST - G - SF - K - L - F - K - K - D - PSQ - SN - K - Y - G - Q - L - F - E - A - E - R - ID - Y - PPL - E - K - L - D - M - V - VSY - A - N - IT - K - G - G - M - S - VPF - Y - N - SR - A - T - K - IA - I - V - VSG - E - G - C - VE - IA - CPH - L - SSSK - SSH - PSY - K - K - L - R - A - R - IR - K - D - T - VF - I - VPA - G - H - PF - A - T - VA - SG - N - E - N - L - E - I - VCF - E - VN - A - E - G - N - IR - Y - T - L - A - G - K - K - N - I - IK - VM - E - K - E - A - K - E - L - A - F - K - M - E - G - E - E - VD - K - VF - G - K - Q - D - E - E - F - F - F - Q - G - PE - W - R - K - E - K - E - G - R - A - D - E
2	Ana o 1.0102	PPT - K - F - SF - SL - F - L - VS - VL - VL - CL - G - F - A - L - A - K - ID - PE - L - K - Q - CK - H - Q - CK - VQ - R - Q - Y - D - E - Q - Q - K - E - Q - C - VK - E - CE - K - Y - Y - K - E - K - K - G - R - E - R - E - H - E - E - E - E - E - E - W - G - T - G - G - VD - E - PST - H - E - PA - E - K - H - L - SQ - CM - R - Q - CE - R - Q - E - G - G - Q - Q - K - Q - L - CR - F - R - CQ - E - R - Y - K - K - E - R - G - Q - H - N - Y - K - R - E - D - D - E - D - E - D - E - D - E - A - E - E - E - D - E - N - PY - VF - E - D - E - D - F - T - T - K - VK - T - E - Q - G - K - V - VL - L - PK - F - T - Q - K - SK - L - L - H - A - L - E - K - Y - R - L - A - VL - VA - N - PQ - A - F - V - VPSH - M - D - A - D - S - IF - F - VSW - G - R - G - T - IT - K - IL - E - N - K - R - E - S - IN - VR - Q - G - D - I - VS - ISSG - T - PF - Y - IA - N - N - D - E - N - E - K - L - Y - L - VQ - F - L - R - P - VN - L - PG - H - F - E - VF - H - G - PG - G - E - N - PE - SF - Y - R - A - F - SW - E - IL - E - A - A - L - K - T - SK - D - T - L - E - K - L - F - E - K - Q - D - Q - G - T - IM - K - A - SK - E - Q - VR - A - M - SR - R - G - E - G - PK - IW - PF - T - E - E - ST - G - SF - K - L - F - K - K - D - PSQ - SN - K - Y - G - Q - L - F - E - A - E - R - ID - Y - PPL - E - K - L - D - M - V - VSY - A - N - IT - K - G - G - M - S - VPF - Y - N - SR - A - T - K - IA - I - V - VSG - E - G - C - VE - IA - CPH - L - SSSK - SSH - PSY - K - K - L - R - A - R - IR - K - D - T - VF - I - VPA - G - H - PF - A - T - VA - SG - N - E - N - L - E - I - VCF - E - VN - A - E - G - N - IR - Y - T - L - A - G - K - K - N - I - IK - VM - E - K - E - A - K - E - L - A - F - K - M - E - G - E - E - VD - K - VF - G - K - Q - D - E - E - F - F - F - Q - G - PE - W - R - K - E - K - E - G - R - A - D - E
3	Ana o 2.0101	L - S - VCF - L - IL - F - H - G - CL - A - SR - Q - E - W - Q - Q - Q - D - E - CQ - ID - R - L - D - A - L - E - PD - N - R - VE - Y - E - A - G - T - VE - A - W - D - PN - H - E - Q - F - R - CA - G - VA - L - VR - H - T - IQ - PN - G - L - L - L - PQ - Y - SN - A - PQ - L - IY - V - VQ - G - E - G - M - T - G - ISY - PG - CPE - T - Y - Q - A - PQ - Q - G - R - Q - Q - G - Q - SG - R - F - Q - D - R - H - Q - K - IR - R - F - R - R - G - D - I - IA - IPA - G - VA - H - W - CY - N - E - G - N - SP - V - VT - VT - L - L - D - VSN - SQ - N - Q - L - D - R - T - PR - K - F - H - L - A - G - N - PK - D - VF - Q - Q - Q - Q - Q - H - Q - SR - G - R - N - L - F - SG - F - D - T - E - L - L - A - E - A - F - Q - VD - E - R - L - IK - Q - L - K - SE - D - N - R - G - G - I - VK - VK - D - D - E - L - R - V - IR - PSR - SQ - SE - R - G - SE - SE - E - E - SE - D - E - K - R - R - W - G - Q - R - D - N - G - IE - E - T - ICT - M - R - L - K - E - N - IN - D - PA - R - A - D - IY - T - PE - VG - R - L - T - T - L - N - SL - N - L - P - IL - K - W - L - Q - L - S - VE - K - G - VL - Y - K - N - A - L - VL - PH - W - N - L - N - SH - S - I - IY - G - CK - G - K - G - Q - VQ - V - VD - N - F - G - N - R - VF - D - G - E - VR - E - G - Q - M - L - V - VPQ - N - F - A - V - VK - R - A - R - E - E - R - F - E - W - ISF - K - T - N - D - R - A - M - T - SPL - A - G - R - T - S - VL - G - G - M - PE - E - VL - A - N - A - F - Q - ISR - E - D - A - R - K - IK - F - N - N - Q - Q - T - T - L - T - SG - E - SSH - H - M - R - D - D - A
4	Ana o 3.0101	M - A - K - F - L - L - L - L - SA - F - A - VL - L - L - VA - N - A - S - IY - R - A - I - VE - VE - E - D - SG - R - E - Q - SCQ - R - Q - F - E - E - Q - Q - R - F - R - N - CQ - R - Y - VK - Q - E - VQ - R - G - G - R - Y - N - Q - R - Q - E - SL - R - E - CCQ - E - L - Q - E - VD - R - R - CR - CQ - N - L - E - Q - M - VR - Q - L - Q - Q - Q - E - Q - IK - G - E - E - VR - E - L - Y - E - T - A - SE - L - PR - ICS - ISPSQ - G - CQ - F - Q - SSY

5	Car i 1.0101	M - A - R - VA - A - L - L - VA - L - L - F - VA - N - A - A - A - F - R - T - T - IT - T - M - E - ID - E - D - ID - N - PR - R - R - G - E - SCR - E - Q - IQ - R - Q - Q - Y - L - N - R - CQ - D - Y - L - R - Q - Q - CR - SG - G - Y - D - E - D - N - Q - R - Q - H - F - R - Q - CCQ - Q - L - SQ - M - E - E - Q - CQ - CE - G - L - R - Q - A - VR - Q - Q - Q - Q - E - E - G - IR - G - E - E - M - E - E - M - VQ - CA - SD - L - PK - E - CG - ISSR - SCE - IR - R - SW - F
6	Car i 2.0101	M - VT - K - A - K - IPL - F - L - F - L - SA - L - F - L - A - L - VCSSL - A - L - E - T - E - D - L - SN - E - L - N - PH - H - D - PE - SH - R - W - E - F - Q - Q - CQ - E - R - CQ - H - E - E - R - G - Q - R - Q - A - Q - Q - CQ - R - R - CE - E - Q - L - R - E - R - E - R - E - R - E - R - E - E - I - VD - PR - E - PR - K - Q - Y - E - Q - CR - E - T - CE - K - Q - D - PR - Q - Q - PQ - CE - R - R - CE - R - Q - F - Q - E - Q - Q - E - R - E - R - R - E - R - R - R - G - R - D - D - D - D - K - E - N - PR - D - PR - E - Q - Y - R - Q - CE - E - H - CR - R - Q - G - Q - G - Q - R - Q - Q - Q - Q - CQ - SR - CE - E - R - F - E - E - E - Q - R - R - Q - E - E - R - E - R - R - R - G - R - D - N - D - D - E - E - N - PR - D - PR - E - Q - Y - R - Q - CQ - E - H - CR - R - Q - G - Q - G - Q - R - Q - Q - Q - Q - CQ - SR - CE - E - R - L - E - E - E - Q - R - K - Q - E - E - R - E - R - R - R - G - R - D - E - D - D - Q - N - PR - D - PE - Q - R - Y - E - Q - CQ - Q - Q - CE - R - Q - R - R - G - Q - E - Q - Q - L - CR - R - R - CE - Q - Q - R - Q - Q - E - E - R - E - R - Q - R - G - R - D - R - Q - D - PQ - Q - Q - Y - H - R - CQ - R - R - CQ - T - Q - E - Q - SPE - R - Q - R - Q - CQ - Q - R - CE - R - Q - Y - K - E - Q - Q - G - R - E - W - G - PD - Q - A - SPR - R - E - SR - G - R - E - E - E - Q - Q - R - H - N - PY - Y - F - H - SQ - G - L - R - SR - H - E - SG - E - G - E - VK - Y - L - E - R - F - T - E - R - T - E - L - L - R - G - IE - N - Y - R - V - V - IL - E - A - N - PN - T - F - VL - PY - H - K - D - A - E - S - V - I - V - VT - R - G - R - A - T - L - T - F - VSQ - E - R - R - E - SF - N - L - E - Y - G - D - V - IR - VPA - G - A - T - E - Y - V - IN - Q - D - SN - E - R - L - E - M - VK - L - L - Q - P - VN - N - PG - Q - F - R - E - Y - Y - A - A - G - A - Q - ST - E - SY - L - R - VF - SN - D - IL - VA - A - L - N - T - PR - D - R - L - E - R - F - F - D - Q - Q - E - Q - R - E - G - V - I - IR - A - SQ - E - K - L - R - A - L - SQ - H - A - M - SA - G - Q - R - PW - G - R - R - SSG - G - P - ISL - K - SQ - R - SSY - SN - Q - F - G - Q - F - F - E - A - CPE - E - H - R - Q - L - Q - E - M - D - VL - VN - Y - A - E - IK - R - G - A - M - M - VPH - Y - N - SK - A - T - V - V - VY - V - VE - G - T - G - R - F - E - M - A - CPH - D - VSSQ - SY - E - Y - K - G - R - R - E - Q - E - E - E - E - SST - G - Q - F - Q - K - VT - A - R - L - A - R - G - D - IF - V - IPA - G - H - P - IA - IT - A - SQ - N - E - N - L - R - L - VG - F - G - IN - G - K - N - N - Q - R - N - F - L - A - G - Q - N - N - I - IN - Q - L - E - R - E - A - K - E - L - SF - N - M - PR - E - E - IE - E - IF - E - R - Q - VE - SY - F - VPM - E - R - Q - SR - R - G - Q - G - R - D - H - PL - A - S - IL - D - F - A - G - F - F
7	Car i 4.0101	M - A - K - P - IL - L - S - IY - L - CL - I - I - VA - L - F - N - G - CL - A - Q - SG - G - R - Q - Q - H - K - F - G - Q - CQ - L - N - R - L - D - A - L - E - PT - N - R - IE - A - E - A - G - V - IE - SW - D - PN - H - Q - Q - L - Q - CA - G - VA - V - VR - R - T - IE - PN - G - L - L - L - PH - Y - SN - A - PQ - L - VY - IA - R - G - R - G - IT - G - VL - F - PG - CPE - T - F - E - E - SQ - R - Q - SQ - Q - G - Q - R - R - E - F - Q - Q - D - R - H - Q - K - IR - H - F - R - E - G - D - I - IA - F - PA - G - VA - H - W - CY - N - D - G - SSP - V - VA - IF - L - L - D - T - H - N - N - A - N - Q - L - D - Q - N - PR - N - F - Y - L - A - G - N - PD - D - E - F - R - PQ - G - Q - Q - E - Y - E - Q - H - R - R - Q - Q - Q - H - Q - Q - R - R - G - E - H - G - E - Q - Q - R - D - L - G - N - N - VF - SG - F - D - A - E - F - L - A - D - A - F - N - VD - T - E - T - A - R - R - L - Q - SE - N - D - H - R - G - S - I - VR - VE - G - R - Q - L - Q - V - IR - PR - W - SR - E - E - Q - E - H - E - E - R - K - E - R - E - R - E - R - E - SE - SE - R - R - Q - SR - R - G - G - R - D - D - N - G - L - E - E - T - ICT - L - SL - R - E - N - IG - D - PSR - A - D - IY - T - E - E - A - G - R - IST - VN - SH - N - L - P - IL - R - W - L - Q - L - SA - E - R - G - A - L - Y - SD - A - L - Y - VPH - W - N - L - N - A - H - S - V - VY - A - L - R - G - R - A - E - VQ - V - VD - N - F - G - Q - T - VF - D - D - E - L - R - E - G - Q - L - L - T - IPQ - N - F - A - V - VK - R - A - R - D - E - G - F - E - W - VSF - K - T - N - E - N - A - M - VSPL - A - G - R - T - SA - IR - A - L - PE - E - VL - VN - A - F - Q - IPR - E - D - A - R - R - L - K - F - N - R - Q - E - ST - L - VR - SR - SR - SSR - SE - R - R - A - E - V
8	Jug r 1.0101	A - A - L - L - VA - L - L - F - VA - N - A - A - A - F - R - T - T - IT - T - M - E - ID - E - D - ID - N - PR - R - R - G - E - G - CR - E - Q - IQ - R - Q - Q - N - L - N - H - CQ - Y - Y - L - R - Q - Q - SR - SG - G - Y - D - E - D - N - Q - R - Q - H - F - R - Q - CCQ - Q - L - SQ - M - D - E - Q - CQ - CE - G - L - R - Q - V - VR - R - Q - Q - Q - Q - Q - G - L - R - G - E - E - M - E - E - M - VQ - SA - R - D - L - PN - E - CG - ISSQ - R - CE - IR - R - SW - F

9	Jug r 2.0101	R - G - R - D - D - D - D - E - E - N - PR - D - PR - E - Q - Y - R - Q - CQ - E - Y - CR - R - Q - G - Q - G - Q - R - Q - Q - Q - Q - CQ - IR - CE - E - R - L - E - E - D - Q - R - SQ - E - E - R - E - R - R - R - G - R - D - VD - D - Q - N - PR - D - PE - Q - R - Y - E - Q - CQ - Q - Q - CE - R - Q - R - R - G - Q - E - Q - T - L - CR - R - R - CE - Q - R - R - Q - Q - E - E - R - E - R - Q - R - G - R - D - R - Q - D - PQ - Q - Q - Y - H - R - CQ - R - R - CQ - IQ - E - Q - SPE - R - Q - R - Q - CQ - Q - R - CE - R - Q - Y - K - E - Q - Q - G - R - E - R - G - PE - A - SPR - R - E - SR - G - R - E - E - E - Q - Q - R - H - N - PY - Y - F - H - SQ - S - IR - SR - H - E - SE - E - G - E - VK - Y - L - E - R - F - T - E - R - T - E - L - L - R - G - IE - N - Y - R - V - V - IL - D - A - N - PN - T - SM - L - PH - H - K - D - A - E - S - VA - V - VT - R - G - R - A - T - L - T - L - VSQ - E - T - R - E - SF - N - L - E - CG - D - V - IR - VPA - G - A - T - VY - V - IN - Q - D - SN - E - R - L - E - M - VK - L - L - Q - P - VN - N - PG - Q - F - R - E - Y - Y - A - A - G - A - K - SPD - Q - SY - L - R - VF - SN - D - IL - VA - A - L - N - T - PR - D - R - L - E - R - F - F - D - Q - Q - E - Q - R - E - G - V - I - IR - A - SQ - E - K - L - R - A - L - SQ - H - A - M - SA - G - Q - R - PW - G - R - R - SSG - G - P - ISL - K - SE - SPSY - SN - Q - F - G - Q - F - F - E - A - CPE - E - H - R - Q - L - Q - E - M - D - VL - VN - Y - A - E - IK - R - G - A - M - M - VPH - Y - N - SK - A - T - V - V - VY - V - VE - G - T - G - R - Y - E - M - A - CPH - VSSQ - SY - E - G - Q - G - R - R - E - Q - E - E - E - E - ST - G - R - F - Q - K - VT - A - R - L - A - R - G - D - IF - V - IPA - G - H - P - IA - IT - A - SQ - N - E - N - L - R - L - L - G - F - D - IN - G - E - N - N - Q - R - D - F - L - A - G - Q - N - N - I - IN - Q - L - E - R - E - A - K - E - L - SF - N - M - PR - E - E - IE - E - IF - E - SQ - M - E - SY - F - VPT - E - R - Q - SR - R - G - Q - G - R - D - H - PL - A - S - IL - D - F - A - F - F
10	Jug r 3	M - T - G - SL - VL - K - L - SG - M - VL - L - CM - V - VA - A - P - VA - E - A - V - IT - CG - Q - VA - SS - VG - SC - IG - Y - L - R - G - T - VPT - VPPSCCN - G - VK - SL - N - K - A - A - A - T - T - A - D - R - Q - A - A - CE - CL - K - K - T - SG - S - IPG - L - N - PG - L - A - A - G - L - PG - K - CG - VS - VPY - K - IST - ST - N - CK - A - VK
11	Jug r 4.0101	M - A - K - P - IL - L - S - IY - L - F - L - I - VA - L - F - N - G - CL - A - Q - SG - G - R - Q - Q - Q - Q - F - G - Q - CQ - L - N - R - L - D - A - L - E - PT - N - R - IE - A - E - A - G - V - IE - SW - D - PN - N - Q - Q - F - Q - CA - G - VA - V - VR - R - T - IE - PN - G - L - L - L - PQ - Y - SN - A - PQ - L - VY - IA - R - G - R - G - IT - G - VL - F - PG - CPE - T - F - E - E - SQ - R - Q - SQ - Q - G - Q - SR - E - F - Q - Q - D - R - H - Q - K - IR - H - F - R - E - G - D - I - IA - F - PA - G - VA - H - W - SY - N - D - G - SN - P - V - VA - ISL - L - D - T - N - N - N - A - N - Q - L - D - Q - N - PR - N - F - Y - L - A - G - N - PD - D - E - F - R - PQ - G - Q - Q - E - Y - E - Q - H - R - R - Q - Q - Q - R - Q - Q - R - PG - E - H - G - Q - Q - Q - R - G - L - G - N - N - VF - SG - F - D - A - D - F - L - A - D - A - F - N - VD - T - E - T - A - R - R - L - Q - SE - N - D - H - R - R - S - I - VR - VE - G - R - Q - L - Q - V - IR - PR - W - SR - E - E - Q - E - R - E - E - R - K - E - R - E - R - E - R - E - SE - SE - R - R - Q - SR - R - G - G - R - D - D - N - G - L - E - E - T - ICT - L - R - L - R - E - N - IG - D - PSR - A - D - IY - T - E - E - A - G - R - IST - VN - SH - T - L - P - VL - R - W - L - Q - L - SA - E - R - G - A - L - Y - SD - A - L - Y - VPH - W - N - L - N - A - H - S - V - VY - A - L - R - G - R - A - E - VQ - V - VD - N - F - G - Q - T - VF - D - D - E - L - R - E - G - Q - L - L - T - IPQ - N - F - A - V - VK - R - A - R - N - E - G - F - E - W - VSF - K - T - N - E - N - A - M - VSPL - A - G - R - T - SA - IR - A - L - PE - E - VL - A - T - A - F - Q - IPR - E - D - A - R - R - L - K - F - N - R - Q - E - ST - L - VR - SR - PSR - SR - SSR - SE - R - R - A - E - V
12	Pis v 1.0101	M - A - K - L - VL - L - L - SA - F - A - F - L - IL - A - A - N - A - S - IY - R - A - T - VE - VE - G - E - N - L - SSG - Q - SCQ - K - Q - F - E - E - Q - Q - K - F - K - H - CQ - M - Y - VQ - Q - E - VQ - K - SQ - D - G - H - SL - T - A - R - IN - Q - R - Q - Q - CF - K - Q - CCQ - E - L - Q - E - VD - K - K - CR - CQ - N - L - E - Q - M - VK - R - Q - Q - Q - Q - G - Q - F - R - G - E - K - L - Q - E - L - Y - E - T - A - SE - L - PR - M - CN - ISPSQ - G - CQ - F - SSPY - W - SY
13	Pis v 2.0101	M - G - Y - SSL - L - SF - SL - G - F - L - L - L - F - H - CSF - A - Q - IE - Q - V - VN - SQ - Q - R - Q - Q - Q - Q - R - F - Q - T - Q - CQ - IQ - N - L - N - A - L - E - PK - R - R - IE - SE - A - G - VT - E - F - W - D - Q - N - E - E - Q - L - Q - CA - N - VA - VF - R - H - T - IQ - SR - G - L - L - VPSY - D - N - A - PE - L - VY - V - VQ - G - SG - IH - G - A - VF - PG - CPE - T - F - Q - E - E - SQ - SR - SR - SE - R - SQ - Q - SG - E - Q - H - Q - K - VR - P - IQ - E - G - D - V - IA - L - PA - G - VA - H - W - IY - N - N - G - Q - SK - L - VL - VA - L - A - D - VG - N - SE - N - Q - L - D - Q - Y - L - R - K - F - VL - G - G - SPQ - Q - E - IQ - G - SG - Q - SR - SR - SQ - SQ - SSR - R - G - Q - Q - G - Q - Q - SN - N - IL - SA - F - D - E - E - IL - A - Q - SF - N - ID - T - Q - L - A - R - R - L - Q - K - E - K - R - Q - R - G - I - I - VR - VQ - E - D - L - E - VL - SPH - R - Q - E - Q - E - Q - E - Y - E - E - E - R - E - R - R - Q - R - N - G - L - E - E - T - F - CT - M - T - L - K - Y - N - IN - D - PSR - A - D - VY - N - PR - G - G - R - VSS - VN - A - L - N - L - P - IL - R - F - L - Q - L - SA - K - K - G - VL - H - R - D - A - IL - A - PH - W - N - VN - A - H - S - I - VY - IT - R - G - N - G - R - IQ - I - VSE - N - G - E - S - VF - D - E - E - IR - E - G - Q - L - V - V - VPQ - N - F - A - V - VK - R - A - SSD - K - F - E - W - VSF - K - T - N - G - L - SQ - T - SQ - L - A - G - R - VS - VF - R - A - L - PL - D - V - IK - N - SF - D - ISR - E - D - A - R - R - L - K - E - SR - SE - T - T - IF - A - PG - SSSQ - R - K - SQ - SE - R - E - R - Q - R - E - R - E - E - R - E - IH

14	Pis v 2.0201	M - G - Y - SSL - L - SF - SL - G - F - L - L - L - F - H - CSF - A - Q - IE - Q - V - VN - SQ - Q - R - Q - Q - Q - Q - R - F - Q - T - Q - CQ - IQ - N - L - N - A - L - E - PK - R - R - IE - SE - A - G - VT - E - F - W - D - Q - N - E - E - Q - L - Q - CA - N - VA - VF - R - H - T - IQ - SR - G - L - L - VPSY - N - N - A - PE - L - VY - V - VQ - G - SG - IH - G - A - VF - PG - CPE - T - F - Q - E - E - SQ - SQ - SR - SQ - H - SR - SE - R - SQ - Q - SG - E - Q - H - Q - K - VR - H - IR - E - G - D - I - IA - L - PA - G - VA - H - W - IY - N - N - G - Q - SK - L - VL - VA - L - A - D - VG - N - SE - N - Q - L - D - Q - Y - L - R - K - F - VL - G - G - SPQ - Q - E - IQ - G - G - G - Q - SW - SQ - SR - SSR - K - G - Q - Q - SN - N - IL - SA - F - D - E - E - IL - A - Q - SF - N - ID - T - Q - L - VK - K - L - Q - R - E - E - K - Q - R - G - I - I - VR - VK - E - D - L - Q - VL - SPQ - R - Q - E - K - E - Y - SD - N - G - L - E - E - T - F - CT - M - T - L - K - L - N - IN - D - PSR - A - D - VY - N - PR - G - G - R - VT - S - IN - A - L - N - L - P - IL - R - F - L - Q - L - S - VE - K - G - VL - Y - Q - N - A - IM - A - PH - W - N - M - N - A - H - S - I - VY - IT - R - G - N - G - R - M - Q - I - VSE - N - G - E - S - VF - D - E - E - IR - E - G - Q - L - V - V - VPQ - N - F - A - V - VK - R - A - SSD - G - F - E - W - VSF - K - T - N - G - L - A - K - ISQ - L - A - G - R - IS - VM - R - G - L - PL - D - V - IQ - N - SF - D - ISR - E - D - A - W - N - L - K - E - SR - SE - M - T - IF - A - PG - SR - SQ - R - Q - R - N
15	Pis v 3.0101	K - T - D - PE - L - K - Q - CK - H - Q - CK - VQ - R - Q - Y - D - E - E - Q - K - E - Q - CA - K - G - CE - K - Y - Y - K - E - K - K - G - R - E - Q - E - E - E - E - E - E - E - W - G - SG - R - G - R - G - D - E - F - ST - H - E - PG - E - K - R - L - SQ - CM - K - Q - CE - R - Q - D - G - G - Q - Q - K - Q - L - CR - F - R - CQ - E - K - Y - K - K - E - R - R - E - H - SY - SR - D - E - E - E - E - E - E - G - D - E - E - Q - E - E - E - D - E - N - PY - VF - E - D - E - H - F - T - T - R - VK - T - E - Q - G - K - V - V - VL - PK - F - T - K - R - SK - L - L - R - G - L - E - K - Y - R - L - A - F - L - VA - N - PQ - A - F - V - VPN - H - M - D - A - D - S - IF - F - VSW - G - R - G - T - IT - K - IR - E - N - K - R - E - SM - N - VK - Q - G - D - I - IR - IR - A - G - T - PF - Y - I - VN - T - D - E - N - E - K - L - Y - I - VK - L - L - Q - P - VN - L - PG - H - Y - E - VF - H - G - PG - G - E - N - PE - SF - Y - R - A - F - SR - E - VL - E - A - A - L - K - T - PR - D - K - L - E - K - L - F - E - K - Q - D - E - G - A - I - VK - A - SK - E - Q - IR - A - M - SR - R - G - E - G - PS - IW - PF - T - G - K - ST - G - T - F - N - L - F - K - K - D - PSQ - SN - N - Y - G - Q - L - F - E - SE - F - K - D - Y - PPL - Q - E - L - D - IM - VSY - VN - IT - K - G - G - M - SG - PF - Y - N - SR - A - T - K - IA - I - V - VSG - E - G - R - L - E - IA - CPH - L - SSSK - N - SG - Q - E - K - SG - PSY - K - K - L - SSS - IR - T - D - S - VF - V - VPA - G - H - PF - VT - VA - SG - N - Q - N - L - E - IL - CF - E - VN - A - E - G - N - IR - Y - T - L - A - G - K - K - N - I - IE - VM - E - K - E - A - K - E - L - A - F - K - T - K - G - E - E - VD - K - VF - G - K - Q - D - E - E - F - F - F - Q - G - PK - W - R - Q - H - Q - Q - G - R - A - D - E
16	Pis v 4.0101	M - A - L - L - SY - VT - R - K - T - L - T - E - SL - R - L - G - L - K - SH - VR - G - L - Q - T - F - T - L - PD - L - PY - E - Y - G - A - L - E - PA - ISSE - IM - Q - L - H - H - Q - K - H - H - Q - T - Y - IT - N - Y - N - K - A - L - E - Q - L - D - Q - A - IN - K - G - D - A - SA - V - VK - L - Q - SA - IK - F - N - G - G - G - H - IN - H - S - IF - W - K - N - L - T - P - VSE - G - G - G - E - PPH - G - SL - G - W - A - ID - T - N - F - G - SM - E - A - L - IQ - R - M - N - A - E - G - A - A - L - Q - G - SG - W - VW - L - G - L - D - K - E - SK - K - L - V - VE - T - T - A - N - Q - D - PL - VT - K - G - PSL - VPL - L - G - ID - VW - E - H - A - Y - Y - L - Q - Y - K - N - VR - PD - Y - L - K - N - IW - K - V - IN - W - K - Y - A - G - E - L - Y - Q - K - E - CP
17	Pis v 5.0101	M - A - N - PSL - L - SL - S - VCL - L - IL - F - H - G - CL - A - SR - Q - Q - G - Q - Q - Q - N - E - CQ - ID - Q - L - D - A - L - E - PD - N - R - VE - Y - E - A - G - M - VE - T - W - D - PN - H - E - Q - F - R - CA - G - VA - VA - R - H - T - IQ - PN - G - L - R - L - PE - Y - SN - A - PT - L - M - Y - I - VE - G - E - G - M - T - G - T - L - IPG - CPE - T - Y - Q - A - PQ - Q - G - Q - Q - H - G - Q - SSR - F - Q - D - K - H - Q - K - IQ - R - F - R - K - G - D - I - IA - L - PA - G - VA - N - W - CY - N - E - G - N - SP - V - VT - VT - L - L - D - VSN - SQ - N - Q - L - D - M - Y - PR - K - F - N - L - A - G - N - PE - D - E - F - Q - Q - Q - Q - Q - Q - Q - SR - G - R - R - Q - SQ - Q - K - SCN - N - IF - CG - F - D - T - K - IL - A - E - VF - Q - VE - Q - SL - VK - Q - L - Q - N - E - K - D - N - R - G - A - I - VK - VK - G - D - L - Q - V - IR - PPR - R - Q - SE - R - G - F - E - SE - E - E - SE - Y - E - R - G - R - R - G - R - D - N - G - L - E - E - T - ICT - M - K - L - K - E - N - IH - D - PSR - SD - IY - T - PE - VG - R - IT - SL - N - SL - N - L - P - IL - K - W - L - Q - L - SA - E - R - G - VL - Q - N - N - A - L - M - VPH - W - N - F - N - A - H - S - I - VY - G - CK - G - N - A - Q - VQ - V - VD - N - F - G - N - T - VF - D - G - E - VSE - G - Q - IF - V - VPQ - N - F - A - V - VK - R - A - R - G - Q - R - F - E - W - ISF - K - T - N - D - R - A - M - ISPL - A - G - ST - S - VL - R - A - M - PE - E - VL - A - N - A - F - Q - ISR - E - D - A - R - K - IK - F - N - N - E - Q - PT - L - SSG - Q - SSQ - Q - M - R - D - D - A

“-“ – Bond predicted to be susceptible to proteolysis by pepsin, trypsin and chymotrypsin.
